# Impact and effect mechanisms of mass campaigns in resource-constrained health systems: quasi-experimental evidence from polio eradication in Nigeria

**DOI:** 10.1136/bmjgh-2020-004248

**Published:** 2021-03-08

**Authors:** Marco J Haenssgen, Svea Closser, Olakunle Alonge

**Affiliations:** 1 Department of Global Sustainable Development, School of Cross-Faculty Studies, University of Warwick, Coventry, UK; 2 Institute of Advanced Study, Milburn House, University of Warwick, Coventry, UK; 3 Bloomberg School of Public Health, Johns Hopkins University, Baltimore, Maryland, USA

**Keywords:** poliomyelitis, health systems, immunisation

## Abstract

**Background:**

Mass campaigns are a key strategy for delivering life-saving interventions under Global Health Initiatives, especially in weak health system contexts. They are frequently designed parallel to the health system to rapidly achieve programme targets such as vaccination coverage, but we lack quantitative evidence demonstrating their impact and effect mechanisms on health system performance at sub-/national level. This longitudinal study responds to this gap through an analysis of polio eradication campaigns in Nigeria.

**Methods:**

Using four rounds of Demographic and Health Surveys in Nigeria between October 2000 and December 2017, we created a longitudinal dataset containing 88 881 under-5 children/pregnancies. We estimated the relationships between individuals’ campaign exposure and health system performance indices (full RI schedule attainment, maternal healthcare services utilisation and child survival) using multilevel, mixed-effects regression models applied nationally and stratified by the six geopolitical zones in Nigeria.

**Results:**

Nationally, high-frequency mass campaigns had detrimental health systems effects that potentially left 3.6 million children deprived of full immunisation. The frequency of campaigns was most concentrated in regions with weak health systems, where the operations of RI were disrupted, alongside negative effects on child survival and institutional delivery. In contrast, regions with relatively strong health systems and few campaigns experienced beneficial effects on maternal healthcare service utilisation.

**Conclusions:**

As we provide evidence that well-functioning health systems can benefit from mass campaigns under Global Health Initiatives, our work also challenges the established wisdom to intensify mass campaigns in weaker health systems to bypass service provision bottlenecks. Mass campaigns do not inherently benefit or damage a health system, but frequent campaigns in weak health system contexts can impede service provision. We call for an additional burden of proof and active efforts to integrate mass campaigns into routine health services by harmonising implementation plans and service delivery in weak health system contexts.

**Key questions**

**What is already known?**

- Mass campaigns are a key strategy to deliver interventions under Global Health Initiatives, for example, supplementary immunisation activities (SIAs) and are often most heavily used in weaker health systems to bypass service provision bottlenecks.
- The health system effects of mass campaigns are disputed: Commonly cited positive impacts include the strengthening of surveillance, management, and logistical systems and health service demand generation, but resource and workforce requirements of mass campaigns can also drain capacity to provide routine services, and frequent campaigns may lead to public resistance.
- Studies indicate that context matters for mass campaigns: the impact of large numbers of SIAs is likely to be more disruptive in weak health systems, but conclusive quantitative evidence is lacking.

**What are the new findings?**

- Regions of Nigeria with relatively strong health systems had less frequent campaigns and also experienced beneficial effects of campaigns on maternal healthcare service utilisation. In contrast, regions with weak health systems had more frequent mass campaigns, which were more likely to disrupt routine immunisation and to compromise child survival and institutional delivery.
- On the national level, the health system effects of more frequent mass campaigns potentially left 3.6 million Nigerian children between 2000 and 2017 unvaccinated for the full immunisation schedule.
- Our methodologically innovative study clarifies how both positive and negative impacts of mass campaign programmes (such as SIAs) on health systems may be derived within the same country setting—thus confirming patterns that have been laid out in the qualitative literature.

**What do the new findings imply?**

- While SIAs can complement strong health systems and may be the only viable option for providing services in fragile and emergency settings, the conventional logic of targeting mass campaigns to overcome health system weaknesses can also be detrimental for weak health systems.
- Given the significant risk of disrupting weak health systems, mass campaigns should be purposively designed and implemented in ways that facilitate routine health services provision.
- Global health initiatives should work with local stakeholders to strengthen health systems, share resources and harmonise implementation plans for mass campaigns and service delivery.

## Introduction

Mass campaigns deliver essential health interventions to billions of people across the world each year. Cost-effective and proven to achieve coverage goals, campaigns have become a mainstay of global health initiatives (GHIs) aiming to reduce population incidence of specific diseases.^1 2^ Using the case of polio eradication in Nigeria, we analyse supplementary immunisation activities (SIAs), a key type of mass campaigns in global health, to understand whether and how they may support or damage country health system functions.

The Global Polio Eradication Initiative (GPEI) is a global multibillion dollar effort to eradicate polio, originating from a World Health Assembly declaration in 1988.^3^ Estimates suggest that 125 countries were polio-endemic at that time, with an estimated 350 000 cases per year worldwide.^4^ While the original eradication target of the year 2000 was not met, significant progress has been made, with now only two countries (Afghanistan and Pakistan) in which the wild poliovirus is endemic. Nigeria was the most recent country to be declared polio-free—registering the last wild poliovirus case in September 2016—but its path to eliminating polio continues to be challenging, as outbreaks of circulating vaccine-derived poliovirus type 2 have continued into 2020.^5^

A central strategy of the GPEI is the delivery of polio vaccine both through routine immunisation (RI) and through mass vaccination campaigns (SIAs). Official GPEI policy has, since the inception of the programme, advocated for RI strengthening alongside SIAs. However, the GPEI globally and within Nigeria de facto prioritised SIAs from the mid-1990s until relatively recently.^6 7^ Much debate has therefore surrounded the question of whether the resource requirements and institutional arrangements of polio SIAs support^8–11^ or undermine^10 12 13^ broader health system operations including RI. Despite indicative evidence that weak health systems and conflict can militate against the system strengthening effects of campaign inputs,^14–16^ quantitative studies have not been able to establish conclusively a general direction of the impacts of campaigns, or the conditions on which those impacts depend (see Study Design section for further discussion).^10 17–19^

Nigeria is an excellent case study for examining this question because, as one of the world’s last polioendemic countries, it has until recently continued frequent SIAs. In Nigeria, SIAs are multiday events where oral polio vaccine (OPV) is delivered both at fixed sites and door to door, with the aim of achieving high coverage among children under the age of 5. Some of these campaigns are nationwide, but areas with polio transmission are targeted with many additional sub-national campaigns.

Based on a nationwide, repeated cross-sectional and multilevel assessment of individual exposure to SIAs since birth and during pregnancy, we provide systematic evidence that resource-intensive and labour-intensive SIAs in Nigeria are concentrated in areas with relatively weaker health systems, and increased frequency of SIAs can have significantly disruptive effects in these areas. Conversely, SIAs are conducted with less frequency in areas with relatively stronger health systems, and associated with beneficial outcomes in these areas. Hence, the health systems context ultimately shapes the effect of SIAs.

## Methods

### Study design

Our study design responds to the methodological challenges in assessing the heterogeneous impacts of mass campaigns on country health systems; these challenges have resulted in mixed and inconclusive results in previous quantitative research on the topic. Much of this work consists of descriptive studies. Aylward *et al*^20^ interpreted the association between SIA and vaccine coverage trends as evidence of synergies between polio eradication and RI performance. A more recent descriptive study, a cross-sectional survey of GPEI-related staff in 10 countries by van den Ent *et al,*^14^ found that polio staff spent time strengthening RI-related activities but also mentioned that this was easier to do in settings with fewer campaigns. Other descriptive research has involved observational studies of routine service delivery in primary care settings during SIAs. Mounier-Jack *et al*^15^ in Cameroon documented disruption of antenatal care and vaccination services as a result of frequent health campaigns including SIAs, and Omoleke *et al*^16^ indicated a lack of integration between RI, that is, facility-based Expanded Programme Immunisation (EPI) activities, and SIAs in Nigeria that could potentially be detrimental to immunisation services delivery. Although insightful, conclusions about impacts often remain speculative in this descriptive work.

Quasi-experimental analyses have produced similarly mixed results. Using individual-level data from the Demographic and Health Surveys (DHSs), Bonu *et al*^17^ studied RI uptake before and after the introduction of polio eradication efforts between 1990 and 2001 in 15 countries, finding heterogeneous developments of RI coverage over time and even declines of routine polio vaccination in some countries including Nigeria despite the introduction of SIAs. Focusing specifically on routine OPV, Helleringer *et al*^21^ concluded from an analysis of DHS data covering 20 countries that self-reported SIA participation was linked to higher vaccine uptake among poorer economic strata of the population. In contrast, Closser *et al*^10^ found that ‘to the degree that polio eradication campaigns have an effect on outcomes in RI and maternal healthcare, (the modestly positive yet overall mixed) effects are small relative to other factors and are inconsistent from place to place’—based on cross-national panel data (regressing national-level health system indicators against SIA onset and average number of SIAs per year) and individual DHS data from seven countries (relating individual-level and district-level outcomes to the aggregate incidence of SIAs).

A main challenge in these studies is the direct attribution of changes in health system performance to the operation of SIAs. More recent research has therefore made use of unique variation in children’s direct exposure to SIAs. For instance, a study by Helleringer *et al*^22^ in Bangladesh analysed the random timing of births before and after a national mass immunisation campaign. Limiting their focus on children aged 4 months, the authors argue that one-time exposure to the SIA (comparing children born before and after the campaign) significantly increased uptake of the diphtheria, pertussis and tetanus vaccine (DPT) vaccine as well as routine childhood immunisation more broadly. A related study by Chakrabarti *et al*^18^ expanded the analysis to the broader target group of children under 5 years of age. Using the time when a child was first exposed to one SIA as an instrumental variable for total SIA exposure, the authors found an overall negative relationship between SIA and RI, which was statistically significant in three out of five countries for the period 1992–2013. Haenssgen^19^ further incorporated subnationally varying SIAs (ie, subnational polio immunisation days and mop-up campaigns) into quasi-experimental exposure assessments, based on geo-coded child-level data from the District Level Household and Facility Survey in northern India between 2002 and 2008. This study detected an overall negative association between SIA exposure and RI attainment in Uttar Pradesh, whereby age-specific results suggested that older children may be deprived of the opportunity to catch up on missed routine childhood vaccines. However, the analysis also demonstrated a positive association between SIAs and RI in neighbouring Bihar, which had similarly intense SIAs but placed comparatively more policy emphasis on synergies between polio eradication and RI.

Overall, quantitative assessments of SIA impacts on country health systems have undergone substantial evolution, involving a move towards precise quasi-experimental methods, fine-grained analyses of SIA exposure on the individual level, and an expansion of health service assessments beyond RI. We built on the quasi-experimental design by Haenssgen^19^ to assess the health system impact of SIAs at the individual (child) level. Given that the nationwide implementation of the Polio Eradication Initiative limits the study of like-for-like counterfactual settings, we studied different degrees of exposure to various numbers of recurring SIAs on individual (child/pregnancy) outcomes. Our study design exploited exogenous variation in children’s birth dates and survey implementation at an unprecedented level of precision (based on exact implementation dates of SIAs and birth dates of children), while controlling for individual, maternal, household and locational determinants of health service utilisation in a multilevel regression design that helps to take within-country variation of health system performance into account.

### Country setting

Nigeria has a population of around 200 million people—and over 40% of this population is under the age of 18.^23^ Hence, maternal and child health issues including immunisation play an outsized role in shaping the health system. The country is comprised of 36 states and 774 local government areas (LGAs). The health system is decentralised along three tiers: federal, state and LGA levels. The states are often grouped into six geopolitical regions: North-Central, North-East, North-West, South-East, South-West and South-South. Significant variations exist in health system performance across these regions, with generally better indicators of service delivery in the southern regions.^24^

### Patient and public involvement

It was not appropriate or possible to involve patients or the public in the design, or conduct, or reporting, or dissemination plans of our research.

### Data

This analysis used four rounds of geo-coded DHSs (DHSs; https://dhsprogram.com/) from Nigeria, from 2003, 2008, 2013 and 2018. DHSs are standardised and nationally representative surveys of health and education measures. We harmonised the datasets across the survey rounds to ensure consistent measurement of key indicators such as household wealth or ethnic group. We subsequently matched the DHS data with a calendar of national and subnational SIAs in Nigeria from October 2000 until December 2017. Geospatial analysis using ArcGIS V.10.5.1 with administrative boundary data from United Nations Office for the Coordination of Humanitarian Affairs Nigeria^25^ enabled us to identify the LGAs in which the 3533 DHS clusters were located.

Because SIAs aim at full coverage of all children in a target area, we assumed that all children in a targeted LGA would have been exposed to the SIA. There were 312 campaigns recorded in the calendar from October 2000 until December 2017, and 145 of these were SIAs mobilised for polio eradication using OPV and included 25 national immunisation days (which also include, eg, vitamin A administration), 16 subnational immunisation days, 17 immunisation-plus days (which also provide, eg, oral rehydration solutions, anthelmintics and paracetamol), 57 subnational immunisation-plus days, 27 mop-up campaigns or ‘outbreak response’ activities and three maternal/neonatal/child health weeks (a visual summary of these SIAs is presented in online supplemental figure 1.^26^ Over the period from October 2000 to December 2017, the total number of registered SIAs ranged from 45 to 120 per LGA—which yielded a total of 59 127 LGA-SIA pairs (covering the 774 LGAs in Nigeria). The overall accuracy of matching SIAs to LGAs was 96.55%.

10.1136/bmjgh-2020-004248.supp1 Supplementary data

From the combined data set, we excluded women who were not usual residents of the households in which they were interviewed, all children born before the first recorded SIA (October 2000), all survey interviews after the last SIA in our register (December 2017), observations that could not be matched to an LGA, and households who had migrated to the survey sites after the child was born. The remaining sample contained 88 881 observations, spanning 690 LGAs and including 8681 pregnancies with a child that did not live to its fifth birthday. Using the information on SIA dates, we were able to calculate the number of campaign rounds to which children and pregnancies were exposed. We defined three relevant periods: pregnancy period; RI period, that is, birth until age 10 months (when children are expected to receive facility-provided EPI schedule of childhood vaccines, including DPT) and follow-up period, that is, after age 10 months (when children are expected to catch-up on missed vaccines according to the schedule).

### Analysis

To assess the impact of SIAs on RI and other health system functions, we estimated multilevel logistic and linear regression models. In broad terms, these models predict healthcare utilisation/outcomes with SIA exposure (‘EXP’) as key independent variable alongside a range of standard control variables such as socioeconomic status that are commonly identified in the childhood immunisation literature (see online supplemental table 1 for variable summaries and online supplemental table 2 for an overview of the models reported here).^27–29^

10.1136/bmjgh-2020-004248.supp2 Supplementary data

10.1136/bmjgh-2020-004248.supp3 Supplementary data

The three outcomes in our models were full childhood immunisation, maternal healthcare utilisation and child survival. Access to full immunisation was assessed using a binary index (‘1’ if a child had attained three doses of DPT vaccine, one dose of Bacillus Calmette-Guerin (BCG) vaccine, and one dose of measles vaccine). Contrary to the Nigerian RI schedule,^30^ the DHSs did not cover hepatitis B vaccination. We excluded OPV from the indicator for assessing full immunisation owing to potential endogeneity (given that our main exposure variable, SIAs, involves the administration of OPV via campaigns) and recall biases (given that high exposure to SIAs and social mobilisation activities that often accompany SIAs may positively influence a parent’s recall of his or her child’s OPV uptake as part of the full immunisation schedule).^31 32^ Because older children may be differentially affected by SIAs (eg, missed follow-up vaccines), we also disaggregated the relative contribution of exposure during the RI and follow-up periods and analysed interactions between exposure and child age (‘EXPxAGE’).^19^ Maternal healthcare utilisation indicators included the number of antenatal care visits and tetanus toxoid injections before birth as well as the place where the child was delivered (at home, private facility, public facility). Lastly, we studied child survival as a function of SIA exposure during pregnancy and after the birth of the child. As exact birth date information was not always available for children who did not survive (ie, the day of birth is missing), we used monthwise birth date approximations for the SIA exposure measurement.

These analyses were carried out at the individual child/pregnancy level. However, poor health system development at the LGA level may be associated with poor coverage of health services, including full immunisation for children, and this may necessitate more frequent SIAs. Hence, we estimated two-level models with an LGA random effect (level 2) to account for variations in health system development comparing LGAs. To support the development of policy recommendations in light of health system differences, we also stratified the analysis across the six regions in Nigeria; the Northeast and Northwest contain the poorest performing health systems. Furthermore, because potential disruptions may be better managed over time as health systems adapt to SIAs, we also analysed two-way and three-way interactions between the survey year variable, SIA exposure, and child age to detect such dynamic effects.

As part of our robustness checks, we carried out three-level analyses with an additional state random effect (level 3), noting, however, that states face considerable within-state variation in SIA frequency and intensity, owing to which we limit the reporting of our main results to the two-level model specifications (level 2: LGA, level 1: individual child/pregnancy). Other robustness checks included: excluding the DHS 2018 survey round (because most of the SIAs during that year were mop-up activities), stratifying the dataset by DHS-round as an alternative approach for assessing time trends, regressing on a full immunisation outcome that excludes measles, analysing decomposed SIA exposure (with exposure for national immunisation days, immunisation plus days, mop-up campaigns and maternal/neonatal/child health weeks as separate variables), and estimating the main models with LGA-level fixed effects. These robustness checks are reported in (online supplemental tables 3b–5b for the 3-level models, (online supplemental table 6a) for the analysis excluding DHS 2018, (online supplemental table 6b) for the time trends analysis, (online supplemental table 6) for the full immunisation outcome excluding measles, (online supplemental table 6) for the analysis with decomposed SIA exposure and online online supplemental table 6 for the fixed LGA effects models). Overall, the results based on these robustness checks were consistent with our main results. Although the Hausman tests suggested that the fixed effects models may be preferable for the maternal healthcare services utilisation and child survival outcomes, the inclusion of 774 LGA dummy variables in the stratified analyses impacted model fit (complete separation), and the model failed to converge. Moreover, the Hausman tests may be sensitive to large samples as was the case in our analyses.^33 34^ Hence, we report random effects results for consistency. All statistical analyses were carried out in Stata V.16.

10.1136/bmjgh-2020-004248.supp4 Supplementary data

10.1136/bmjgh-2020-004248.supp5 Supplementary data

10.1136/bmjgh-2020-004248.supp6 Supplementary data

10.1136/bmjgh-2020-004248.supp7 Supplementary data

## Results

### Aggregate results

#### Routine immunisation

Table 1 depicts the main results on the link between SIA exposure and non-polio full immunisation (see online supplemental table 3 for the detailed results). The results indicate a negative and statistically significant association between SIA exposure since birth and children’s attainment of non-polio full immunisation. Model 2 shows that the coefficients for SIA exposure during the RI and follow-up periods had the same direction and point estimates, but only the latter was statistically significant at the 1% level. The interaction model (model 3) suggests that the association between SIA exposure and full vaccination also becomes increasingly negative with child age. Two-way and three-way interaction terms involving the survey round dummy were not statistically significant (see online supplemental table 3), indicating that the relationship between SIA exposure and RI attainment did not change across the period 2000–2017.

**Table 1:** Main results: link between SIA exposure and routine childhood immunisation uptake

Dependent variable: Non-polio full immunisation	Full model	Exposure decomposition by period	Interaction model (EXPxAGE)
Model no	(1)	(2)	(3)
EXP_CHI	−0.024†		0.012
	(−0.036 to –0.012)		(−0.009 to 0.034)
EXP_CHI_RI		−0.024*	
		(−0.048 to 0.000)	
EXP_CHI_FU		−0.024†	
		(−0.037 to –0.010)	
EXPxAGE			−0.001†
			(−0.001 to –0.001)
CHI_AGE	0.078†	0.078†	0.038†
	(0.063 to 0.093)	(0.063 to 0.093)	(0.031 to 0.044)
CHI_AGE2	−0.001†	−0.001†	
	(−0.001 to –0.001)	(−0.001 to –0.001)	
Level 1 observations (child)	24 381	24 381	24 381
Level 2 observations (LGA)	684	684	684
Akaike information criterion	18 030.298	18 032.469	18 055.959
Prob.>Χ^2^	<0.001	<0.001	<0.001

Notes. Two-level logistic regression with LGA random effect. Sample include children aged 10–60 months who were alive at the time of the survey and born between Octber 2000 and December 2017. Dependent variable: full immunisation, defined as at least one dose of BCG, three doses of DPT and one dose of measles vaccine as recalled by mother and/or reported by child’s health card. Main results only, omitting child, parental and household determinants of immunisation, survey round dummy, constant and multilevel variance parameter. Interaction terms between SIA exposure and survey round dummy were not statistically significant and the associated models have been omitted from the main results. Coefficients reported, 95% CIs in brackets.

*P<0.10.

†P<0.01.

BCG, Bacillus Calmette-Guerin; DPT, diphtheria, pertussis and tetanus; LGA, local government area; SIA, supplementary immunisation activity.

**Figure 1:**
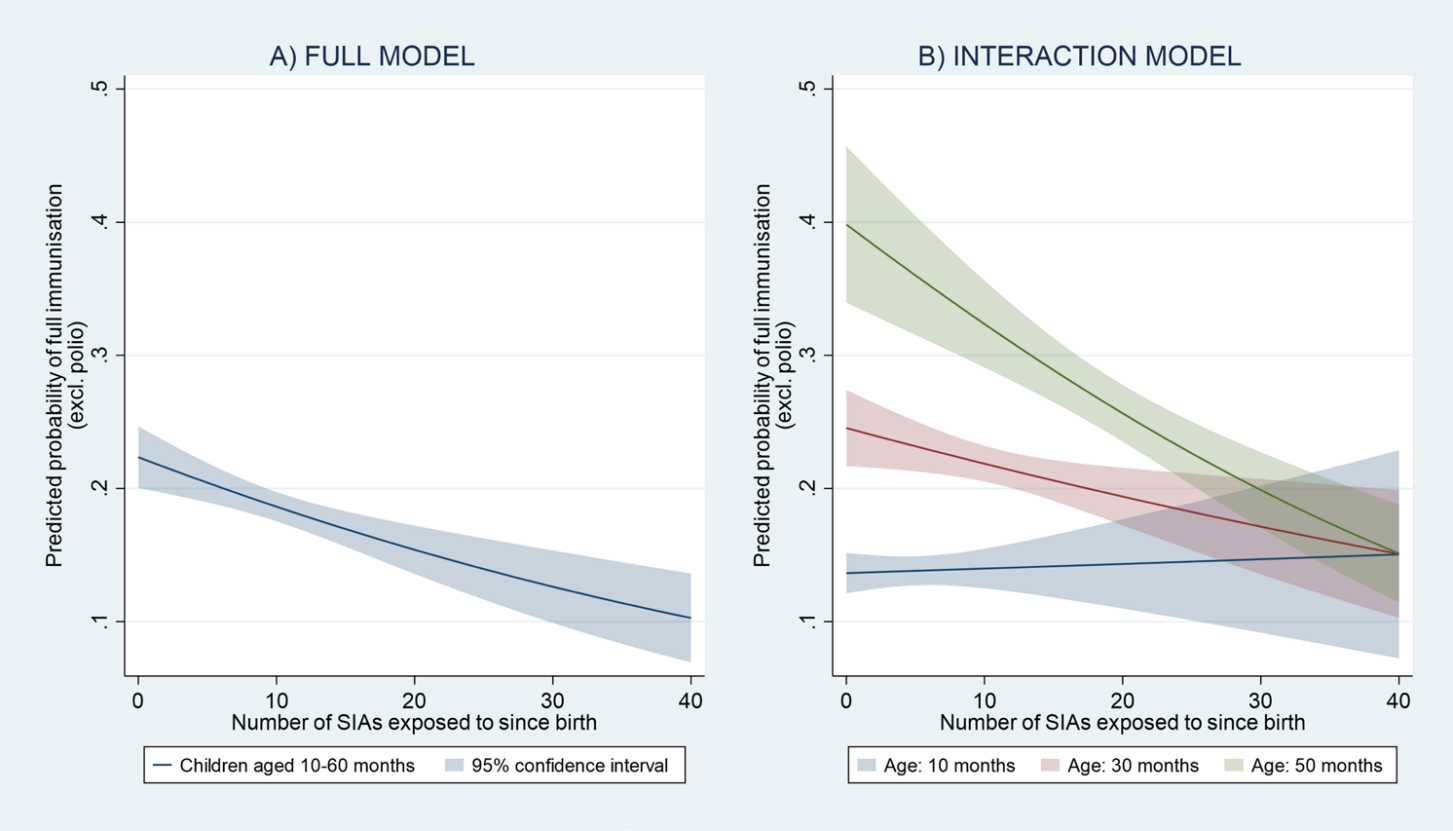
Predicted probability of (non-polio) full immunisation as a function of SIAs to which a child had been exposed since birth (A) and as function of child age (B). Source: authors. Notes. prediction based on models 1 and 3 in table 1 for children aged 10–60 months (n=24 381), controlling for child, parental and household determinants of immunisation with survey round dummy and LGA random effect. Full immunisation defined here as at least one dose of BCG, three doses of DPT and one dose of measles vaccine as recalled by mother and/or reported by child’s health card. DPT, diphtheria, pertussis and tetanus; LGA, local government area; SIAs, supplementary immunisation activities; BCG, Bacillus Calmetter-Guerin.

Based on these results, the full model predicts that children not exposed to SIAs had an average probability of non-polio full immunisation of 22.4% (95% CI 20.0% to 24.7%), whereas children with 10, 20 and 40 rounds of exposure had a probability of 18.6% (95% CI 17.5% to 19.7%), 15.4% (95% CI 13.6% to 17.2%) and 10.3% (95% CI 6.9% to 13.6%), respectively (figure 1A). The age-specific results further suggest that, whereas exposure to 10 SIAs is predicted to yield a 0.3% point higher probability of non-polio full immunisation for a 10-month-old child compared with an unexposed child, the same exposure for a 30-month-old child would entail a 2.7% point lower predicted probability, and a 7.5% point lower probability for a child aged 50 months (figure 1B).

### Maternal health service utilisation

The results about maternal health service uptake are more mixed. Table 2 presents the main findings for SIA exposure during pregnancy separated by place of delivery and antenatal care (see online supplemental table 4 for the detailed results). Only the coefficient for delivery in private facilities was statistically significant (at the 1% level) yet negative. Year interactions did not yield statistically significant results for any of the maternal health service indicators considered here, suggesting again that the overall associations did not change over the study period.

**Table 2:** Main results: link between SIA exposure and maternal health service uptake

Dependent variables: maternal healthcare access	Delivery‡			Antenatal care§	
	At home	At private facility	At public facility	No of antenatal care visits	No of tetanus toxoid injections
Model no	(1)	(2)	(3)	(4)	(5)
EXP_PREG	0.007	−0.065†	0.010	−0.025*	0.001
	(−0.013 to 0.027)	(−0.093 to –0.036)	(−0.009 to 0.029)	(−0.054 to 0.003)	(−0.008 to 0.009)
Level 1 observations (child)	34 713	34 713	34 713	35 019	36 585
Level 2 observations (LGA)	686	686	686	686	687
Akaike information criterion	26 422.845	17 601.384	30 318.818	193 640.288	111 227.001
Prob.>χ^2^	<0.001	<0.001	<0.001	<0.001	<0.001

Notes. Two-level regressions with LGA random effect (see notes a and b for model specification). Sample include pregnancies for children born between October 2000 and December 2017 (the available data for the covariates means that the sample is limited to live births). Dependent variables as defined in table header. Main results only, omitting maternal and household determinants of healthcare access, survey round dummy, constant and multilevel variance parameters. Interaction terms between SIA exposure and survey round dummy were not statistically significant and the associated models have been omitted from the main results. Coefficients reported, 95% CIs in brackets.

*P<0.10.

†P<0.01.

‡Two-level logistic regression models with binary outcome indicators of facility access for delivery.

§Two-level linear regression models of number of antenatal care visits and tetanus toxoid injections prior to delivery. Two-level Poisson regression models yielded similar results and have been omitted from reporting.

LGA, local government area; SIA, supplementary Immunisation activity.

### Child survival

Table 3 presents the main results for child survival suggesting that SIA exposure may be negatively associated with child survival (see online supplemental table 5 for the detailed results). The results of model 1 in table 3 suggest that prebirth SIA exposure (EXP_PREG_nod) did not have a statistically significant association with child survival, whereas postbirth exposure (EXP_CHI_nod) was negatively and statistically significantly linked to child survival (at the 1% level). The combined exposure in model 2 (EXP_TOT_nod) exhibited a similar trend. As before, the year interaction with total SIA exposure was not statistically significant. For illustration, the predicted probability of survival as a function of SIA exposure as per model 1 (controlling for all covariates including child age, predictions based on postbirth exposure only) suggest that a child with zero SIA exposure since birth had a 95.2% probability of survival (95% CI 94.8% to 95.6%), whereas children exposed to ten SIAs had a 0.9% point lower probability of 94.3% (95% CI:94.0% to 94.5%).

**Table 3:** Main results: link between SIA exposure and child survival

Dependent variable: child survival	Exposure decomposition	Total exposure
Model no	(1)	(2)
EXP_PREG_nod (date approximation)	−0.012	
	(−0.034 to 0.010)	
EXP_CHI_nod (date approximation)	−0.019†	
	(−0.029 to –0.009)	
EXP_TOT_nod (total exposure, date approximation)		−0.018†
		(−0.027 to –0.009)
Level 1 observations (child)	52 431	52 431
Level 2 observations (LGA)	689	689
Akaike information criterion	23 684.534	23 682.853
Prob. >Χ^2^	<0.001	<0.001

Notes. Two-level logistic regression with LGA random effect. Sample includes children up to 60 months of age (or aged 60 months at time of death) born between October 2000 and December 2017. Dependent variable: child is alive. Main results only, omitting child, parental and household determinants of immunisation, survey round dummy, constant and multilevel variance parameter. Using date approximations (year-month) as exact birth date information not available for children who did not survive. Total SIA exposure (EXP_TOT_nod) combines exposure during pregnancy and after birth and is used for interaction models (interaction terms were not statistically significant and were omitted from main results). Coefficients reported, 95% CIs in brackets.

*P<0.10.

†P<0.01.

LGA, local government area; SIA, supplementary immunisation activity.

### Disaggregated results

Considering the argument that weak health systems are particularly vulnerable to disruptions from mass campaigns, we demonstrate in online supplemental table 6 that SIAs in Nigeria were concentrated in regions with relatively poorer health system performance and lower wealth index (Northwest, Northeast). For example, compared with the Southwest region, LGAs in the Northwest region had more than twice the number of SIAs during the study period (114 vs 47), while the full immunisation rate of children in the complete survey sample was only one-fifth compared with children in the Southeast region (8.3% vs 40.6%). As a result of the subnational variations in SIA frequency as a result health system challenges, we analysed the relationship between SIA exposure and health service utilisation stratified by the six regions of Nigeria.

The main results of the stratified analysis are presented in table 4, which shows the regression coefficients for SIA exposure across regions and the various outcome variables (see online supplemental table 7 for the detailed results). The stratified analysis has a lower statistical power due to smaller samples. However, the overall negative and significant association between SIA exposure and non-polio full immunisation persisted in the two regions with the poorest health system performance (Northwest, Northeast). The exposure–child age interaction term was furthermore statistically significant and negative in the Northcentral, Southeast and South-South, suggesting that the only region without a detectable relationship between a negative full immunisation outcome and SIAs was the Southwest. The statistical associations suggest that, for instance, children in the Northwest with zero SIA exposure had an average predicted probability of 9.4% of full immunisation, whereas the predicted probabilities for children with 10 and 20 rounds of exposure were 4.8% and 2.3%, respectively. The negative interaction terms in the better performing South of Nigeria suggest that younger children may indeed be more likely to receive their scheduled shots if exposed to SIAs, while further exposure of older children to additional SIAs may not improve their likelihood of catching up on previously missed scheduled vaccines.

10.1136/bmjgh-2020-004248.supp8 Supplementary data

**Table 4:** Main results: link between SIA exposure and child/maternal health outcomes, stratified by region

Model	Main dependent variables	Region					
		North central	North east	North west	South east	South South	South west
Non-polio full immunisation status (full model)	EXP_CHI	0.026*	−0.074‡	−0.076†	0.092	0.065	−0.060
		(−0.004, 0.056)	(−0.106 to –0.042)	(−0.151 to –0.002)	(−0.035, 0.220)	(−0.042, 0.171)	(−0.144, 0.024)
Non-polio full immunisation status (EXPxAGE interaction model)	EXP_CHI	0.054†	−0.058†	−0.034	0.238†	0.171†	−0.019
		(0.009, 0.100)	(−0.104 to –0.013)	(−0.110, 0.042)	(0.056, 0.420)	(0.001, 0.341)	(−0.138, 0.101)
	EXPxAGE	−0.001†	0.000	−0.001*	−0.005‡	−0.003‡	−0.002*
		(−0.002 to –0.000)	(−0.001, 0.001)	(−0.002, 0.000)	(−0.008 to –0.002)	(−0.006 to –0.001)	(−0.004, 0.000)
Delivery: at home	EXP_PREG	0.003	−0.025	−0.015	0.036	−0.023	0.055
		(−0.036, 0.042)	(−0.067, 0.017)	(−0.067, 0.037)	(−0.062, 0.135)	(−0.102, 0.056)	(−0.013, 0.122)
Delivery: at private facility	EXP_PREG	−0.015	0.024	−0.059	0.020	0.038	−0.003
		(−0.061, 0.031)	(−0.103, 0.151)	(−0.197, 0.080)	(−0.060, 0.099)	(−0.074, 0.150)	(−0.059, 0.052)
Delivery: at public facility	EXP_PREG	0.002	0.018	0.025	−0.049	0.014	−0.033
		(−0.034, 0.038)	(−0.024, 0.060)	(−0.028, 0.078)	(−0.129, 0.030)	(−0.060, 0.088)	(−0.087, 0.022)
Antenatal care: no of antenatal care visits	EXP_PREG	0.034	0.015	0.025	0.155†	0.072	−0.069
		(−0.030, 0.097)	(−0.026, 0.055)	(−0.005, 0.055)	(0.001, 0.308)	(−0.055, 0.198)	(−0.206, 0.069)
Antenatal care: no of tetanus injections	EXP_PREG	0.003	0.001	0.002	0.010	0.037*	0.034‡
		(−0.015, 0.021)	(−0.017, 0.019)	(−0.013, 0.017)	(−0.021, 0.042)	(−0.000, 0.074)	(0.009, 0.058)
Child survival (exposure decomposition)	EXP_PREG_nod (date approximation)	−0.019	0.019	−0.008	0.036	−0.056	−0.021
		(−0.076, 0.037)	(−0.022, 0.060)	(−0.051, 0.035)	(−0.116, 0.189)	(−0.206, 0.094)	(−0.145, 0.102)
	EXP_CHI_nod (date approximation)	0.030*	0.005	0.025	−0.043	−0.005	0.018
		(−0.001, 0.061)	(−0.020, 0.029)	(−0.011, 0.060)	(−0.171, 0.085)	(−0.139, 0.128)	(−0.103, 0.139)
Child survival (total exposure)	EXP_TOT_nod (total exposure, date approximation)	0.018	0.008	0.013	−0.013	−0.026	−0.001
		(−0.008, 0.044)	(−0.013, 0.030)	(−0.019, 0.045)	(−0.126, 0.099)	(−0.142, 0.091)	(−0.103, 0.101)

Notes. Two-level logistic regression with LGA random effect. Sample includes children up to 60 months of age (or aged 60 months at time of death) born between October 2000 and December 2017. Dependent variables as indicated in column ‘model,’ corresponding to aggregate results presented above. Main results only, omitting child, parental and household determinants of immunisation, survey round dummy, constant and multilevel variance parameter. Coefficients reported, 95% CIs in brackets.

*P<0.10.

†P<0.05.

‡P<0.01.

LGA, local government area; SIA, supplementary immunisation activity.

For maternal healthcare utilisation, a positive and statistically significant relationship between antenatal care and SIA exposure during the pregnancy emerged in regions with relatively better health system performance. These associations suggest, for instance, that a mother in the Southeast region exposed to five rounds of SIAs was predicted to make 8.7 antenatal care visits compared with 7.9 visits for unexposed mothers. In contrast to healthcare utilisation, the child mortality results did not persist in the stratified analysis, suggesting that this association may be relatively weak overall (aggregate fixed effects regression results presented in online supplemental table 6 suggest that an effect may nonetheless be present, rather than being a product of reverse causality).

In support of the subregional heterogeneity described here, the intraclass correlations of the explanatory variables included in our random effects models ranged from 1.2% (child survival) to 21.8% (antenatal care visits) (see online supplemental table 8). This suggests that health system factors mapped to the LGA level (level 2) explain a substantial proportion of the variance in our models.

10.1136/bmjgh-2020-004248.supp9 Supplementary data

## Discussion

Our analysis showed a negative overall relationship between mass campaign exposure and routine health services performance, especially for full immunisation catch-up among older children, and to a lesser extent for delivery and child survival. Campaigns were more frequent in areas with weaker health systems—a finding reflective of unofficial global GPEI strategy—which appears to have driven the overall negative relationship between full immunisation and SIAs. SIAs may in fact be associated with increased maternal health services utilisation in regions with relatively stronger health systems (Southeast, Southwest). The type of SIA (eg, national immunisation days, immunisation plus days, mop-up campaigns and maternal/neonatal/child health weeks) was not differentially associated with the outcomes (online supplemental table 6), which suggests that the shared characteristic as ‘campaigns’ dominates in the observed relationship, rather than the specific combination of activities within the different types of SIA.

While other studies have come to similar conclusions about the relationship between mass campaigns and health system performance,^10 17–19 35^ our study is one of the first to quantify the magnitude and subnational variation of their impacts on health systems. Thus, we address a major methodological challenge of directly attributing changes in health system performance to the operation of SIAs using quantitative models. Our study is supported by the quality of the underlying DHS data and their nationally representative samples from multiple survey years.

Our analysis is limited by our inability to evaluate systemic changes arising from the introduction of SIAs as part of other broader GHIs apart from GPEI. Cross-national before-and-after comparisons are better suited to assess such impacts, which might well extend beyond the indicators assessed here, including, for instance, Ebola or helminths control.^8 36^ In addition, our analysis may be susceptible to omitted variable bias (eg, specific LGA factors that influence service delivery outcomes)—but our wide range of evidence-based explanatory variables, the use of random effects models, and our robustness checks as reported in online supplemental file 1, at least partly offset this risk.

10.1136/bmjgh-2020-004248.supp10 Supplementary data

As we focus analytically on the impacts of SIA exposure in the presence of sub-national health system variation, this study also does not directly examine the underlying drivers that explain people’s relationship to government health services or vertical interventions. This point, however, has been addressed in related work.^37–39^ What for instance materialises as ‘distrust’ towards the government and vaccination campaigns in Nigeria’s northern regions (also illustrated in box 1 below) is arguably rooted in deprivation, discrimination, colonial histories and the local epidemiological context. Globally, culture and religion are also often mentioned as key factors shaping people’s relationship to government health services, but extensive research has linked these patterns to underlying historical trauma that population subgroups have experienced—rather than, for example, the practice of religion itself—alongside issues of power in global health policy-making.^37–39^ These underlying factors determining people’s health service utilisation and their relationship to the government are related to our findings. Ethnographic work shows that in northern Nigeria, vaccine hesitancy is fuelled by heavy government focus on frequent, well-funded polio campaigns in the context of crumbling health systems—the discrepancy makes already distrustful people nervous.^38 39^

Box 1 **Case studies of polio eradication in Nigeria**

**Polio campaigns and health systems in the Northwest region: Kano state, 2012^10 38^**

The polio campaign in Kano state in January 2012 was kicked off in a pavilion with cushioned chairs for assembled dignitaries. Soap, whistles, biscuits and infant clothes were prominently displayed on large tables to entice mothers to bring their children in for vaccination.

At the same time, teams of health workers went door to door with polio vaccine. Refusals were common in some areas. Workers commented that there was a stark contrast between poor-quality health services and frequent, well-funded polio campaigns, which made people nervous. A community health worker said, ‘People keep asking, why is it that polio vaccine is to be taken house to house and free of charge, while if you go to the hospital other drugs are never free. This makes people raise a question mark even on the other routine vaccines given at the hospital free of charge.’^38^

Health centres in the area had cracked walls and broken refrigerators, were frequently out of basic supplies, and were often short of staff. These staff shortages were exacerbated during polio campaigns. A health worker commented, ‘Once the polio campaign is flagged up, every other activity is halt in the primary health centre, because every high official’s attention will focus on the polio campaigns.’

‘Eighty per cent of the healthcare activity will stop until the polio campaign days are over,’ another worker added.^10^

**Polio campaigns and health systems in the Southwest region: Ondo state, 2013^44 45^**

For the year of 2012, Bill Gates, along with President Goodluck Jonathan, announced the ‘Governor’s Immunisation Leadership Challenge’—a competition for Nigerian states to increase their polio campaign and immunisation coverage. The idea was that by providing large monetary prizes of up to US$1 million, states would be incentivised to increase immunisation coverage enough to finally eliminate polio from the country.

States competed to improve their polio campaign performance data, and in 2013, Ondo state was announced the winner. Energised by this win, state officials poured the US$500 000 in prize money into scaling up its fledgling Healthy Mothers Healthy Babies programme, expanding it into more facilities, and creating and aggressively tracking new indicators on maternal mortality. The governor said, ‘Winning the Bill and Melinda Gates Polio Challenge was an added impetus to pursue more aggressive healthcare projects in the state.’^44^

Thus, policy-makers and managers of a state with relatively strong health system in the Southwest region used polio assets to strengthen broader health system goals. Kano state, the case study in the Northwest region described above, also entered the Immunisation Leadership Challenge. However, their immunisation coverage was too low to receive any prize money.^45^

We take our results therefore as evidence that SIAs do not inherently benefit or damage a health system, but that large numbers of campaigns in weak health system contexts may strain limited capacity, and create systemic issues leading to adverse outcomes (see box 1).^39^ In contrast, stronger health systems are more able to take advantage of added resources that mass campaigns provide to produce beneficial outcomes.^10 40^ Hence, the programmatic and health system effects of mass campaigns are mainly determined by the critical interaction between mass campaigns and health systems in context.

In areas with weak health systems and low full immunisation coverage across the world, the GPEI made a strategic decision to increase the number of campaigns, rather than using funding to support RI. This correlation holds in Nigeria as it does globally.^10^ Thus, the health systems most in need of support were targeted with high numbers of SIAs that this analysis shows can cause further damage to the health system. The scale of such damage can be considerable. Based on our results (and given average numbers of SIAs and cohort sizes of the under-5 population), we estimate that, between 2000 and 2017, every additional SIA was associated with between 41 700 and 127 500 Nigerian children deprived from being fully immunised. Over our entire study period, this translates into lost opportunities to fully immunise more than 3.6 million children (these effects primarily pertain to young children rather than infants given the age-specific non-linear relationship between SIA exposure and full immunisation). These numbers do not represent an exact accounting: the relationship between a heavy campaign burden and a very weak health system is complex. But our analysis shows that repeated campaigns (on a near-monthly basis in some areas) can further interfere with already weak health services provision.

Our study shows that these disruptive effects do not improve or change over time, suggesting that health systems do not evolve passively to mitigate disruptive impacts of SIAs. Nigeria and the African region were recently certified polio-free (although vaccine-derived polio persists),^41^ and plans are underway to repurpose the SIAs for delivery of other health interventions. It is important for policy-makers and health system managers in Nigeria and elsewhere to actively harmonise implementation plans and service delivery between mass campaigns and routine health services. This can be achieved by focusing on support for mid-level district health officers and front-line health workers, who are often responsible for both routine services and SIAs. Given the worse outcomes for children older than 10 months associated with high-intensity SIAs, it is important that any integrated plan includes activities for providing services to children above the age of 10 months who missed their routine shots, especially in the Northwest and Northeast of Nigeria.

We do not call for abandoning mass campaigns as a strategic approach since they may be the only viable option to providing services and saving lives in fragile and emergency settings. Rather, as mass campaigns can leave a weak health system worse-off, implementing such programmes should require an additional burden of proof that the programmes’ design and intensity will at least not disrupt and ideally facilitate the operation of routine services. Prior system readiness assessments should contextualise and adapt mass campaigns to their contexts, nationally and subnationally. Such adaptation requires adopting and implementing explicit goals to strengthen weak health systems as part of the campaign programme (rather than divorced from them and delivered by another entity). The ensuing health system strengthening strategies should be locally owned and could involve, for example, production of human resources for health and other health services inputs to accompany mass campaigns (or in fact be adopted in lieu of repeated campaigns) in weaker health systems. While such an approach is likely to yield the twin goals of achieving the specific programmatic outcomes of a campaign (eg, raise immunisation coverage for a specific antigen) and support of the health system, it is likely to be more cost-intensive and time-intensive and will require cooperation and coordination of processes among multiple stakeholders.

The health system impacts of mass campaigns are a critical subject in light of persistent aspirations in global public health to control infectious diseases (eg, malaria and measles) through mass campaign programmes, and in light of likely future mass immunisation campaigns for emerging infectious diseases such as the COVID-19. Our study speaks not only to SIAs for polio eradication but to the broader use of mass campaigns.^42 43^ We are not making a general argument for or against mass campaigns; the health system context (including actors, institutions, infrastructure, processes and overall capacity) drives the result of these campaigns,^35^ and there are trade-offs. For example, policy-makers may need to weigh the benefits of averting a crippling infectious disease for thousands of children against the costs of depriving them of other vital routine services, and potentially contributing to higher mortality—from conditions addressed by those routine services—in areas with weak health systems where frequent SIAs are targeted. Proponents of SIAs as a major tool should be open and more circumspect about their potential for health system disruptions. The design and implementation of such campaigns should be approached with an understanding of the health system context—and should include strategies to strengthen routine health services and not work around them.
