# Supplementary material for: Impact and effect mechanisms of mass campaigns in resource-constrained health systems: quasi-experimental evidence from polio eradication in Nigeria
Source: BMJ Glob Health. 2021 Mar 8;6(3):e004248. doi: 10.1136/bmjgh-2020-004248 (PMC7942242; doi:10.1136/bmjgh-2020-004248)
Supplement: Supplementary data [file bmjgh-2020-004248supp001.pdf]

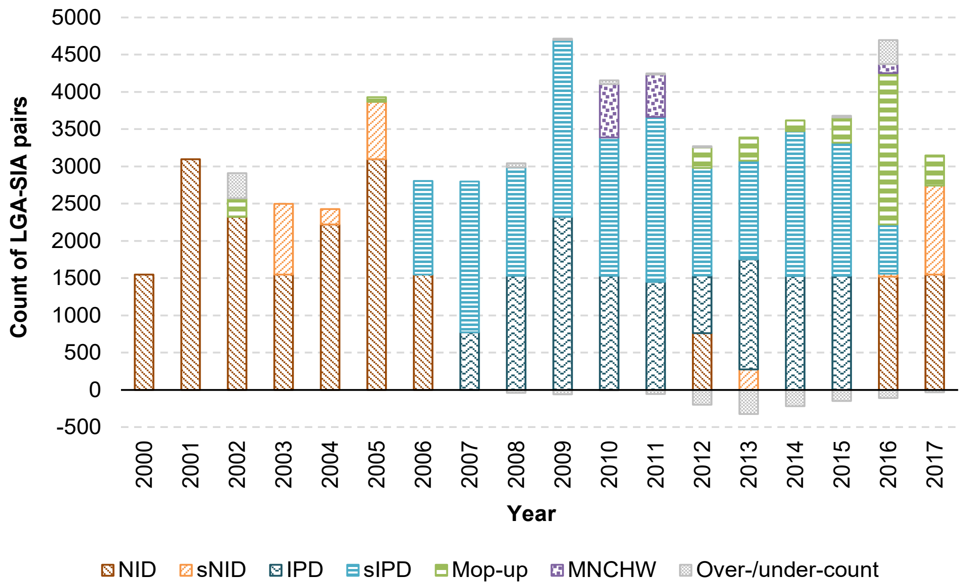

Figure 1. Overview of SIA coverage of Nigerian LGAs, 2000-2017

Source: Authors.

Notes: n = 59,127 LGA-SIA pairs (a total of 145 SIAs and 744 LGAs, with 45-120 SIAs per LGA between 2000 and 2017). NID = National Immunisation Day; sNID = sub-National Immunsiation Day; IPD = Immunisation-Plus Day; sIPD = sub-national Immunisation-Plus Day; MNCHW = Maternal, Neonatal, and Child Health Week; SIA = Supplementary Immunisation Activity; LGA = Local Government Area. "Mop-up" campaigns include revaccination and outbreak response campaigns. Over-/under-count refers to ability to match LGAs in accordance with SIA register of the World Health Organization, with over-count being displayed as negative values (i.e. representing excess LGA count).
