# Supplementary material for: Impact and effect mechanisms of mass campaigns in resource-constrained health systems: quasi-experimental evidence from polio eradication in Nigeria
Source: BMJ Glob Health. 2021 Mar 8;6(3):e004248. doi: 10.1136/bmjgh-2020-004248 (PMC7942242; doi:10.1136/bmjgh-2020-004248)
Supplement: Supplementary data [file bmjgh-2020-004248supp002.pdf]

Table 1: Variable Summary

Variable Name	Variable Description		N	Mean	Std. Dev.	Min	Max	Unique Values
**Outcome Variables: Childhood vaccination**								
**VAC**	Full vaccination (excl. polio & hepatitis B) [1 = yes]		69,376	0.27	0.44	0	1	2
**VAC_norep**	Full vaccination (excl. polio & hepatitis B, health card only) [1		18,792	0.44	0.5	0	1	2
**Outcome Variables: Pregnancy health service utilisation**								
**MOT_ANC**	Number of antenatal visits during pregnancy		55,790	4.57	5.07	0	29	30
**MOT_TET**	Number of tetanus toxoid injections before birth		57,962	1.31	1.32	0	9	10
**MOT_DEL_home**	Place of delivery: home [1 = yes]		87,532	0.65	0.48	0	1	2
**MOT_DEL_priv**	Place of delivery: private healthcare provider [1 = yes]		87,532	0.12	0.32	0	1	2
**MOT_DEL_pub**	Place of delivery: public healthcare provider [1 = yes]		87,532	0.24	0.42	0	1	2
**Outcome Variables: Child survival**								
**CHI_ALIVE**	Child is alive [1 = alive]		88,881	0.9	0.3	0	1	2
**Key Independent Variables: SIA Exposure**								
**EXP_CHI**	SIA exposure since birth (exact date)		58,453	11.11	9.71	0	42	43
**EXP_CHI_nod**	SIA exposure since birth (approx. date)		88,881	11.34	9.85	0	42	43
**EXP_CHI_RI**	SIA exposure since birth (exact date, RI period)		58,453	3.89	2.33	0	11	13
**EXP_CHI_RI_nod**	SIA exposure since birth (approx. date, RI period)		88,881	3.73	2.44	0	10	13
**EXP_CHI_FU**	SIA exposure since birth (exact date, follow-up period)		58,453	7.5	8.42	0	37	38
**EXP_CHI_FU_nod**	SIA exposure since birth (approx. date, follow-up period)		88,881	7.77	8.53	0	37	38
**EXP_PREG**	SIA exposure during pregnancy (exact date)		58,453	3.67	2.01	0	9	12
**EXP_PREG_nod**	SIA exposure during pregnancy (approximate date)		88,881	3.83	2.12	0	9	12
**EXP_TOT_nod**	Total SIA exposure pre- & post-birth (approximate date)		88,881	15.2	10.76	0	48	49
**Other Control Variables**								
**CHI_AGE**	Age of child (months)		88,881	28.29	17.35	0	60	61
**CHI_ORD**	Birth order of child		88,881	4.01	2.6	1	18	18
**CHI_SEX**	Sex of child [1 = female]		88,881	0.49	0.5	0	1	2
**MOT_AWE**	Child has health card [1 = yes]		68,957	0.52	0.5	0	1	2
**MOT_AGE**	Age of mother at birth (years)		88,881	27.1	6.9	12	49	38
**MOT_EDM**	Mother's highest year of education		88,842	2.43	2.64	0	10	10
**MOT_EDF**	Husband/partner's highest year of education		84,741	3.01	2.7	0	9	10
	Mother's religion	Catholic		0.08	0.28	0	1	
	(categorical variable)	Other Christian		0.3	0.46	0	1	
**HH_REL**		Islam	88,565	0.6	0.49	0	1	5
		Traditionalist		0.01	0.1	0	1	
		Other		0	0.05	0	1	
	Mother's ethnicity	Ekoi		0.01	0.08	0	1	
	(categorical variable)	Fulani		0.09	0.29	0	1	
		Hausa		0.32	0.47	0	1	
		Ibibio		0.01	0.12	0	1	
		Igala		0.01	0.1	0	1	
**HH_ETH**		Igbo	88,646	0.11	0.31	0	1	11
		Ijaw / Izon		0.03	0.17	0	1	
		Kanuri / Beriberi		0.02	0.15	0	1	
		Tiv		0.02	0.14	0	1	
		Yoruba		0.1	0.3	0	1	
		Other		0.27	0.44	0	1	
**HH_SIZ**	Number of household members		88,881	7.13	3.68	1	43	37
**HH_WEA**	Household asset wealth (out of 12 assets)		88,881	3.62	2.5	0	12	13
**INF_RUR**	Rural/urban residence [1 = rural]		88,881	0.69	0.46	0	1	2
	Survey year	2003		0.02	0.15	0	1	
	(categorical variable)	2008		0.29	0.45	0	1	
**YEAR**		2013	88,881	0.35	0.48	0	1	4
		2018		0.34	0.47	0	1	
**SIA intensity**	LGA above [2] or below [1] median total no. of SIAs 2000-2017		88,881	1.66	0.47	1	2	2
