# Supplementary material for: Impact and effect mechanisms of mass campaigns in resource-constrained health systems: quasi-experimental evidence from polio eradication in Nigeria
Source: BMJ Glob Health. 2021 Mar 8;6(3):e004248. doi: 10.1136/bmjgh-2020-004248 (PMC7942242; doi:10.1136/bmjgh-2020-004248)
Supplement: Supplementary data [file bmjgh-2020-004248supp003.pdf]

Table 2: Model overview

		Childhood vaccination (Table 1)		Pregnancy health service utilisation (Table 2)				Child survival (Table 3)	
	Outcome variables:	VAC		MOT_ANC<br>MOT_TET		MOT_DEL_home<br>MOT_DEL_priv<br>MOT_DEL_pub		CHI_ALIVE	
	Functional form:	Logistic regression		Linear regression		Logistic regression		Logistic regression	
	Analysis:	Main results	Robust-ness checks	Main results	Robust-ness checks	Main results	Robust-ness checks	Main results	Robust-ness checks
Key independent variables	EXP_CHI								
	EXP_CHI_RI								
	EXP_CHI_FU								
	EXP_CHI_nod								
	EXP_CHI_RI_nod								
	EXP_CHI_FU_nod								
	EXP_PREG								
	EXP_PREG_nod								
	EXP_TOT_nod								
	EXPxYR								
	EXPxAGE								
	EXPxAGExYEAR								
Other control variables	CHI_AGE								
	CHI_AGE^2								
	CHI_ORD								
	CHI_SEX								
	MOT_ANC								
	MOT_AWE								
	MOT_AGE								
	MOT_EDM								
	MOT_EDF								
	HH_REL								
	HH_ETH								
	HH_SIZ								
	HH_WEA								
	INF_RUR								
	YEAR								
	LGA random effect								
	State random effect								
	Fixed effects								
	Decomposition of SIAs								
	Stratification by region								
	Stratification by survey round								
	Exclusion of 2018 round								
	Non-polio/mealses RI outcome								
	Alternative reporting (health card only)								
