# Supplementary material for: Impact and effect mechanisms of mass campaigns in resource-constrained health systems: quasi-experimental evidence from polio eradication in Nigeria
Source: BMJ Glob Health. 2021 Mar 8;6(3):e004248. doi: 10.1136/bmjgh-2020-004248 (PMC7942242; doi:10.1136/bmjgh-2020-004248)
Supplement: Supplementary data [file bmjgh-2020-004248supp004.pdf]

Table 3: Main results: Link between SIA exposure and routine childhood immunisation uptake

Dependent variable: non-polio full immunisation status	Full model	Interaction model (EXPxAGE)	Exposure decomposition
EXP_CHI	-0.024*** [-0.036, -0.012]	0.012 [-0.009, 0.034]	
EXP_CHI_RI			-0.024* [-0.048, 0.000]
EXP_CHI_FU			-0.024*** [-0.037, -0.010]
EXPxAGE		-0.001*** [-0.001, -0.001]	
EXPxYR [yr = 2008]			
EXPxYR [yr = 2013]			
EXPxYR [yr = 2018]			
AGExYR [yr = 2008]			
AGExYR [yr = 2013]			
AGExYR [yr = 2018]			
EXPxAGExYR [yr = 2008]			
EXPxAGExYR [yr = 2013]			
EXPxAGExYR [yr = 2018]			
CHI_AGE	0.078*** [0.063, 0.093]	0.038*** [0.031, 0.044]	0.078*** [0.063, 0.093]
CHI_AGE2	-0.001*** [-0.001, -0.001]		-0.001*** [-0.001, -0.001]
Level 1 Observations (child)	24381	24381	24381
Level 2 Observations (LGA)	684	684	684
Akaike Information Criterion	18030.298	18055.959	18032.469
Prob. > X^2	<0.001	<0.001	<0.001

95% confidence intervals in brackets
* p < 0.10, ** p < 0.05, *** p < 0.01

Table 3a: Detailed results: Link between SIA exposure and routine childhood immunisation uptake

	Main results					Robustness checks: reporting			
				Year-Interaction models		Full model		Interaction model (EXPxAGE)	
Dependent variable: non-polio full immunisation status	Full model	Interaction model (EXPxAGE)	Exposure decomposition	(EXPxYR)	(EXPxAGExYR)	Only health card reported vaccines (VAC_norep)	Date approximation (EXP_CHI_nod)	Only health card reported vaccines (VAC_norep)	Date approximation (EXP_CHI_nod)
EXP_CHI	-0.024***	0.012		-0.053	-0.551	-0.002	-0.025***	0.036**	0.004
	[-0.036, -0.012]	[-0.009, 0.034]		[-0.217, 0.112]	[-1.460, 0.359]	[-0.021, 0.018]	[-0.036, -0.015]	[0.002, 0.070]	[-0.014, 0.022]
EXP_CHI_RI			-0.024*						
			[-0.048, 0.000]						
EXP_CHI_FU			-0.024***						
			[-0.037, -0.010]						
EXPxAGE		-0.001***			0.020			-0.001***	-0.001***
		[-0.001, -0.001]			[-0.032, 0.072]			[-0.002, -0.000]	[-0.001, -0.000]
EXPxYR [yr = 2008]				0.010	0.531				
				[-0.154, 0.174]	[-0.379, 1.440]				
EXPxYR [yr = 2013]				0.027	0.570				
				[-0.137, 0.190]	[-0.339, 1.479]				
EXPxYR [yr = 2018]				0.041	0.711				
				[-0.124, 0.205]	[-0.203, 1.626]				
AGExYR [yr = 2008]					-0.062				
					[-0.251, 0.126]				
AGExYR [yr = 2013]					-0.058				
					[-0.246, 0.130]				
AGExYR [yr = 2018]					-0.060				
					[-0.249, 0.129]				
EXPxAGExYR [yr = 2008]					-0.020				
					[-0.072, 0.032]				
EXPxAGExYR [yr = 2013]					-0.021				
					[-0.073, 0.031]				
EXPxAGExYR [yr = 2018]					-0.026				
					[-0.078, 0.026]				
CHI_AGE	0.078***	0.038***	0.078***	0.033***	0.098	0.069***	0.070***	0.033***	0.036***
	[0.063, 0.093]	[0.031, 0.044]	[0.063, 0.093]	[0.027, 0.039]	[-0.090, 0.286]	[0.046, 0.091]	[0.057, 0.083]	[0.023, 0.043]	[0.030, 0.042]
CHI_AGE2	-0.001***		-0.001***			-0.001***	-0.001***		
	[-0.001, -0.001]		[-0.001, -0.001]			[-0.001, -0.000]	[-0.001, -0.000]		
CHI_ORD	-0.060***	-0.061***	-0.061***	-0.061***	-0.062***	-0.083***	-0.076***	-0.083***	-0.077***
	[-0.085, -0.036]	[-0.086, -0.037]	[-0.085, -0.036]	[-0.085, -0.036]	[-0.086, -0.037]	[-0.121, -0.046]	[-0.098, -0.055]	[-0.121, -0.046]	[-0.099, -0.055]
CHI_SEX	-0.003	-0.004	-0.003	-0.007	-0.006	-0.036	0.014	-0.037	0.013
	[-0.078, 0.071]	[-0.079, 0.070]	[-0.078, 0.071]	[-0.081, 0.068]	[-0.081, 0.069]	[-0.146, 0.074]	[-0.050, 0.079]	[-0.147, 0.073]	[-0.051, 0.078]
MOT_ANC	0.055***	0.056***	0.055***	0.055***	0.055***	0.037***	0.059***	0.037***	0.059***
	[0.046, 0.064]	[0.047, 0.064]	[0.046, 0.064]	[0.046, 0.064]	[0.047, 0.064]	[0.025, 0.049]	[0.051, 0.067]	[0.025, 0.050]	[0.051, 0.067]
MOT_EDM	0.061***	0.061***	0.061***	0.061***	0.062***	0.050***	0.057***	0.049***	0.057***
	[0.043, 0.079]	[0.044, 0.079]	[0.043, 0.079]	[0.043, 0.079]	[0.044, 0.080]	[0.023, 0.077]	[0.041, 0.072]	[0.022, 0.076]	[0.041, 0.072]
MOT_EDF	0.010	0.010	0.010	0.009	0.010	-0.021	0.008	-0.021	0.008
	[-0.008, 0.028]	[-0.008, 0.028]	[-0.008, 0.028]	[-0.009, 0.028]	[-0.009, 0.028]	[-0.049, 0.007]	[-0.008, 0.024]	[-0.049, 0.008]	[-0.008, 0.024]
MOT_AWE	2.972***	2.969***	2.972***	2.957***	2.965***	0.000	2.990***	0.000	2.986***
	[2.848, 3.096]	[2.846, 3.093]	[2.848, 3.096]	[2.833, 3.080]	[2.841, 3.089]	[0.000, 0.000]	[2.882, 3.099]	[0.000, 0.000]	[2.878, 3.095]
MOT_AGE	0.026***	0.026***	0.026***	0.026***	0.027***	0.037***	0.031***	0.037***	0.032***
	[0.018, 0.035]	[0.018, 0.035]	[0.018, 0.035]	[0.017, 0.035]	[0.018, 0.035]	[0.024, 0.050]	[0.024, 0.039]	[0.024, 0.050]	[0.024, 0.039]
HH_RUR	-0.087*	-0.090*	-0.087*	-0.093*	-0.098*	-0.182**	-0.094**	-0.184**	-0.097**
	[-0.190, 0.016]	[-0.193, 0.013]	[-0.190, 0.016]	[-0.195, 0.010]	[-0.201, 0.005]	[-0.324, -0.039]	[-0.186, -0.002]	[-0.326, -0.042]	[-0.189, -0.005]
HH_REL (ref: Catholic): Other Christian	-0.126*	-0.128*	-0.126*	-0.130*	-0.124*	-0.131	-0.105*	-0.133	-0.107*
	[-0.267, 0.015]	[-0.269, 0.013]	[-0.267, 0.015]	[-0.271, 0.011]	[-0.265, 0.017]	[-0.318, 0.056]	[-0.230, 0.020]	[-0.320, 0.054]	[-0.232, 0.018]
HH_REL (ref: Catholic): Islam	-0.472***	-0.481***	-0.472***	-0.467***	-0.476***	-0.460***	-0.427***	-0.472***	-0.434***
	[-0.644, -0.300]	[-0.653, -0.308]	[-0.645, -0.300]	[-0.639, -0.295]	[-0.649, -0.304]	[-0.697, -0.222]	[-0.580, -0.275]	[-0.709, -0.234]	[-0.586, -0.281]
HH_REL (ref: Catholic): Traditionalist	-0.546***	-0.543***	-0.546***	-0.552***	-0.559***	-0.470	-0.513***	-0.474	-0.508***
	[-0.954, -0.139]	[-0.950, -0.137]	[-0.954, -0.139]	[-0.959, -0.146]	[-0.967, -0.152]	[-1.121, 0.182]	[-0.892, -0.135]	[-1.126, 0.178]	[-0.886, -0.129]
HH_REL (ref: Catholic): Other	-0.228	-0.222	-0.229	-0.207	-0.185	-0.956	-0.158	-0.957	-0.160
	[-1.246, 0.789]	[-1.236, 0.792]	[-1.246, 0.789]	[-1.220, 0.805]	[-1.198, 0.827]	[-2.528, 0.616]	[-0.927, 0.611]	[-2.528, 0.614]	[-0.927, 0.608]
HH_ETH (ref: Ekoi): Fulani	-0.930***	-0.958***	-0.931***	-0.866***	-0.900***	-0.782**	-0.961***	-0.799**	-0.983***
	[-1.438, -0.422]	[-1.467, -0.450]	[-1.438, -0.423]	[-1.374, -0.358]	[-1.411, -0.390]	[-1.430, -0.133]	[-1.423, -0.499]	[-1.448, -0.149]	[-1.445, -0.521]
HH_ETH (ref: Ekoi): Hausa	-0.851***	-0.892***	-0.852***	-0.777***	-0.828***	-0.983***	-0.771***	-1.018***	-0.798***
	[-1.337, -0.365]	[-1.379, -0.404]	[-1.339, -0.365]	[-1.263, -0.290]	[-1.318, -0.339]	[-1.585, -0.382]	[-1.217, -0.325]	[-1.621, -0.414]	[-1.245, -0.352]
HH_ETH (ref: Ekoi): Ibibio	-0.636**	-0.629**	-0.636**	-0.589**	-0.579**	-0.704**	-0.622**	-0.696**	-0.620**
	[-1.171, -0.101]	[-1.165, -0.094]	[-1.171, -0.101]	[-1.123, -0.055]	[-1.115, -0.044]	[-1.360, -0.047]	[-1.117, -0.126]	[-1.354, -0.038]	[-1.115, -0.124]
HH_ETH (ref: Ekoi): Igala	-0.660**	-0.659**	-0.660**	-0.610**	-0.600**	-0.540	-0.519*	-0.532	-0.521*
	[-1.245, -0.075]	[-1.245, -0.073]	[-1.246, -0.075]	[-1.194, -0.026]	[-1.186, -0.014]	[-1.326, 0.246]	[-1.058, 0.020]	[-1.320, 0.255]	[-1.061, 0.018]
HH_ETH (ref: Ekoi): Igbo	-0.453*	-0.449*	-0.453*	-0.409*	-0.404*	-0.804***	-0.428*	-0.804***	-0.427*
	[-0.922, 0.016]	[-0.919, 0.020]	[-0.922, 0.016]	[-0.876, 0.059]	[-0.874, 0.065]	[-1.362, -0.245]	[-0.864, 0.009]	[-1.364, -0.245]	[-0.864, 0.009]
HH_ETH (ref: Ekoi): Ijaw / Izon	-0.489*	-0.487*	-0.489*	-0.456*	-0.443	-1.058***	-0.358	-1.051***	-0.357
	[-1.029, 0.052]	[-1.029, 0.054]	[-1.029, 0.052]	[-0.995, 0.083]	[-0.984, 0.098]	[-1.737, -0.378]	[-0.859, 0.143]	[-1.731, -0.370]	[-0.858, 0.145]
HH_ETH (ref: Ekoi): Kanuri / Beriberi	-0.994***	-1.025***	-0.994***	-0.900***	-0.966***	-1.482***	-0.949***	-1.518***	-0.972***
	[-1.598, -0.389]	[-1.630, -0.420]	[-1.599, -0.390]	[-1.504, -0.295]	[-1.574, -0.359]	[-2.320, -0.643]	[-1.492, -0.405]	[-2.357, -0.679]	[-1.515, -0.428]
HH_ETH (ref: Ekoi): Tiv	-0.601**	-0.618**	-0.602**	-0.566**	-0.565**	-1.137***	-0.497*	-1.145***	-0.515**
	[-1.158, -0.045]	[-1.175, -0.060]	[-1.158, -0.045]	[-1.121, -0.010]	[-1.123, -0.007]	[-1.828, -0.445]	[-1.010, 0.017]	[-1.838, -0.452]	[-1.028, -0.001]
HH_ETH (ref: Ekoi): Yoruba	-0.574**	-0.573**	-0.574**	-0.526**	-0.527**	-0.775***	-0.479**	-0.773***	-0.480**
	[-1.051, -0.096]	[-1.051, -0.095]	[-1.051, -0.096]	[-1.002, -0.050]	[-1.006, -0.049]	[-1.351, -0.198]	[-0.923, -0.036]	[-1.351, -0.195]	[-0.924, -0.036]
HH_ETH (ref: Ekoi): Other	-0.620***	-0.632***	-0.620***	-0.558**	-0.577**	-0.802***	-0.542**	-0.810***	-0.553**
	[-1.081, -0.158]	[-1.094, -0.170]	[-1.082, -0.159]	[-1.019, -0.097]	[-1.040, -0.113]	[-1.356, -0.248]	[-0.970, -0.114]	[-1.365, -0.254]	[-0.981, -0.125]
HH_SIZ	-0.013**	-0.013**	-0.013**	-0.013**	-0.013**	-0.023**	-0.006	-0.023**	-0.006
	[-0.026, -0.000]	[-0.026, -0.001]	[-0.026, -0.000]	[-0.026, -0.001]	[-0.026, -0.001]	[-0.042, -0.003]	[-0.016, 0.005]	[-0.042, -0.003]	[-0.016, 0.005]
HH_WEA	0.142***	0.141***	0.142***	0.141***	0.142***	0.119***	0.119***	0.118***	0.119***
	[0.122, 0.162]	[0.121, 0.161]	[0.122, 0.162]	[0.121, 0.161]	[0.123, 0.162]	[0.090, 0.147]	[0.102, 0.136]	[0.089, 0.146]	[0.102, 0.135]
YEAR (ref: 2003): 2008	0.606***	0.604***	0.606***	0.693**	0.944	0.753***	0.712***	0.745***	0.710***
	[0.346, 0.866]	[0.343, 0.865]	[0.346, 0.866]	[0.102, 1.283]	[-1.902, 3.790]	[0.392, 1.113]	[0.470, 0.954]	[0.384, 1.107]	[0.467, 0.953]
YEAR (ref: 2003): 2013	0.749***	0.740***	0.748***	0.703**	0.779	0.873***	0.902***	0.864***	0.894***
	[0.491, 1.006]	[0.482, 0.999]	[0.491, 1.006]	[0.117, 1.289]	[-2.061, 3.618]	[0.518, 1.228]	[0.662, 1.142]	[0.509, 1.220]	[0.653, 1.135]
YEAR (ref: 2003): 2018	0.843***	0.919***	0.844***	0.771**	0.977	1.161***	1.056***	1.222***	1.115***
	[0.578, 1.108]	[0.654, 1.183]	[0.578, 1.109]	[0.177, 1.366]	[-1.879, 3.834]	[0.796, 1.526]	[0.817, 1.295]	[0.858, 1.586]	[0.877, 1.354]
Constant	-5.247***	-4.888***	-5.247***	-4.684***	-5.085***	-1.906***	-5.438***	-1.598***	-5.122***
	[-5.854, -4.640]	[-5.479, -4.296]	[-5.854, -4.640]	[-5.467, -3.902]	[-7.967, -2.202]	[-2.682, -1.130]	[-5.995, -4.880]	[-2.348, -0.849]	[-5.666, -4.578]
Multilevel variance parameter: Level 1	0.285***	0.286***	0.285***	0.280***	0.283***	0.254***	0.337***	0.255***	0.336***
	[0.218, 0.352]	[0.219, 0.353]	[0.218, 0.352]	[0.214, 0.346]	[0.216, 0.349]	[0.161, 0.347]	[0.269, 0.405]	[0.162, 0.348]	[0.268, 0.403]
Level 1 Observations (child)	24381	24381	24381	24381	24381	6514	31805	6514	31805
Level 2 Observations (LGA)	684	684	684	684	684	622	687	622	687
Akaike Information Criterion	18030.298	18055.959	18032.469	18070.207	18050.327	7972.642	23901.729	7980.84	23926.462
Prob. > X^2	<0.001	<0.001	<0.001	<0.001	<0.001	<0.001	<0.001	<0.001	<0.001

95% confidence intervals in brackets
* p < 0.10, ** p < 0.05, *** p < 0.01

Table 3b: Robustness Check (3-Level models): Link between SIA exposure and routine childhood immunisation uptake

Dependent variable: non-polio full immunisation status	Main results					Robustness checks: reporting			
				Year-Interaction models		Full model		Interaction model (EXPxAGE)	
	Full model	Exposure decomposition	Interaction model (EXPxAGE)	(EXPxYR)	(EXPxAGExYR)	Only health card reported vaccines (VAC_norep)	Date approximation (EXP_CHI_nod)	Only health card reported vaccines (VAC_norep)	Date approximation (EXP_CHI_nod)
EXP_CHI	-0.020***<br>[-0.033, -0.006]		0.023*<br>[-0.001, 0.047]	-0.067<br>[-0.231, 0.098]	-0.453<br>[-1.359, 0.453]	0.001<br>[-0.021, 0.022]	-0.022***<br>[-0.034, -0.011]	0.043**<br>[0.007, 0.079]	0.010<br>[-0.009, 0.030]
EXP_CHI_RI		-0.020<br>[-0.046, 0.005]							
EXP_CHI_FU		-0.020***<br>[-0.034, -0.005]							
EXPxAGE			-0.001***<br>[-0.001, -0.001]		0.015<br>[-0.037, 0.066]			-0.001***<br>[-0.002, -0.000]	-0.001***<br>[-0.001, -0.000]
EXPxYR [yr = 2008]				0.028<br>[-0.135, 0.191]	0.449<br>[-0.456, 1.354]				
EXPxYR [yr = 2013]				0.043<br>[-0.120, 0.206]	0.481<br>[-0.424, 1.386]				
EXPxYR [yr = 2018]				0.057<br>[-0.107, 0.220]	0.624<br>[-0.286, 1.534]				
AGExYR [yr = 2008]					-0.071<br>[-0.259, 0.117]				
AGExYR [yr = 2013]					-0.067<br>[-0.255, 0.121]				
AGExYR [yr = 2018]					-0.070<br>[-0.259, 0.118]				
EXPxAGExYR [yr = 2008]					-0.015<br>[-0.067, 0.037]				
EXPxAGExYR [yr = 2013]					-0.016<br>[-0.068, 0.036]				
EXPxAGExYR [yr = 2018]					-0.021<br>[-0.073, 0.032]				
CHI_AGE	0.076***<br>[0.061, 0.091]	0.076***<br>[0.061, 0.091]	0.036***<br>[0.029, 0.042]	0.032***<br>[0.025, 0.038]	0.106<br>[-0.082, 0.294]	0.068***<br>[0.045, 0.091]	0.069***<br>[0.055, 0.082]	0.032***<br>[0.022, 0.043]	0.034***<br>[0.028, 0.040]
CHI_AGE2	-0.001***<br>[-0.001, -0.001]	-0.001***<br>[-0.001, -0.001]				-0.001***<br>[-0.001, -0.000]	-0.001***<br>[-0.001, -0.000]		
CHI_ORD	-0.060***<br>[-0.085, -0.036]	-0.060***<br>[-0.085, -0.036]	-0.061***<br>[-0.086, -0.036]	-0.061***<br>[-0.085, -0.036]	-0.061***<br>[-0.086, -0.037]	-0.082***<br>[-0.120, -0.045]	-0.075***<br>[-0.097, -0.054]	-0.082***<br>[-0.120, -0.045]	-0.076***<br>[-0.098, -0.054]
CHI_SEX	-0.002<br>[-0.076, 0.073]	-0.002<br>[-0.076, 0.073]	-0.003<br>[-0.077, 0.072]	-0.004<br>[-0.079, 0.070]	-0.004<br>[-0.078, 0.070]	-0.040<br>[-0.150, 0.069]	0.014<br>[-0.050, 0.079]	-0.041<br>[-0.151, 0.069]	0.013<br>[-0.052, 0.078]
MOT_ANC	0.057***<br>[0.048, 0.066]	0.057***<br>[0.048, 0.066]	0.057***<br>[0.048, 0.066]	0.056***<br>[0.048, 0.065]	0.057***<br>[0.048, 0.066]	0.038***<br>[0.025, 0.051]	0.060***<br>[0.052, 0.068]	0.038***<br>[0.025, 0.051]	0.060***<br>[0.052, 0.068]
MOT_EDM	0.060***<br>[0.042, 0.078]	0.060***<br>[0.042, 0.078]	0.060***<br>[0.042, 0.078]	0.060***<br>[0.042, 0.078]	0.061***<br>[0.043, 0.079]	0.049***<br>[0.022, 0.076]	0.056***<br>[0.040, 0.071]	0.048***<br>[0.021, 0.075]	0.056***<br>[0.040, 0.071]
MOT_EDF	0.009<br>[-0.010, 0.027]	0.009<br>[-0.010, 0.027]	0.009<br>[-0.010, 0.027]	0.008<br>[-0.010, 0.027]	0.008<br>[-0.010, 0.027]	-0.023<br>[-0.051, 0.005]	0.007<br>[-0.008, 0.023]	-0.023<br>[-0.051, 0.005]	0.007<br>[-0.009, 0.023]
MOT_AWE	2.962***<br>[2.838, 3.086]	2.962***<br>[2.838, 3.086]	2.960***<br>[2.836, 3.084]	2.946***<br>[2.822, 3.070]	2.955***<br>[2.831, 3.079]	0.000<br>[0.000, 0.000]	2.991***<br>[2.882, 3.100]	0.000<br>[0.000, 0.000]	2.987***<br>[2.878, 3.096]
MOT_AGE	0.027***<br>[0.018, 0.035]	0.027***<br>[0.018, 0.035]	0.027***<br>[0.018, 0.036]	0.027***<br>[0.018, 0.035]	0.027***<br>[0.019, 0.036]	0.037***<br>[0.024, 0.050]	0.032***<br>[0.024, 0.039]	0.037***<br>[0.024, 0.050]	0.032***<br>[0.024, 0.039]
HH_RUR	-0.062<br>[-0.165, 0.040]	-0.062<br>[-0.165, 0.040]	-0.065<br>[-0.168, 0.038]	-0.068<br>[-0.170, 0.035]	-0.072<br>[-0.175, 0.031]	-0.176**<br>[-0.320, -0.033]	-0.066<br>[-0.159, 0.026]	-0.180**<br>[-0.323, -0.037]	-0.068<br>[-0.161, 0.024]
HH_REL (ref: Catholic): Other Christian	-0.112<br>[-0.254, 0.030]	-0.112<br>[-0.253, 0.030]	-0.114<br>[-0.256, 0.028]	-0.116<br>[-0.257, 0.025]	-0.109<br>[-0.251, 0.033]	-0.160*<br>[-0.349, 0.029]	-0.093<br>[-0.218, 0.033]	-0.163*<br>[-0.352, 0.026]	-0.094<br>[-0.220, 0.031]
HH_REL (ref: Catholic): Islam	-0.429***<br>[-0.605, -0.254]	-0.429***<br>[-0.605, -0.254]	-0.433***<br>[-0.608, -0.257]	-0.430***<br>[-0.605, -0.255]	-0.432***<br>[-0.607, -0.256]	-0.437***<br>[-0.680, -0.194]	-0.392***<br>[-0.548, -0.237]	-0.447***<br>[-0.690, -0.204]	-0.394***<br>[-0.550, -0.239]
HH_REL (ref: Catholic): Traditionalist	-0.498**<br>[-0.906, -0.091]	-0.498**<br>[-0.906, -0.090]	-0.493**<br>[-0.901, -0.085]	-0.503**<br>[-0.910, -0.096]	-0.509**<br>[-0.918, -0.101]	-0.476<br>[-1.127, 0.176]	-0.459**<br>[-0.840, -0.079]	-0.480<br>[-1.131, 0.172]	-0.453**<br>[-0.833, -0.073]
HH_REL (ref: Catholic): Other	-0.318<br>[-1.344, 0.709]	-0.318<br>[-1.345, 0.708]	-0.316<br>[-1.339, 0.708]	-0.292<br>[-1.312, 0.729]	-0.280<br>[-1.302, 0.742]	-1.100<br>[-2.685, 0.485]	-0.212<br>[-0.986, 0.562]	-1.106<br>[-2.689, 0.478]	-0.215<br>[-0.989, 0.558]
HH_ETH (ref: Ekoi): Fulani	-0.802***<br>[-1.341, -0.262]	-0.802***<br>[-1.342, -0.263]	-0.816***<br>[-1.357, -0.275]	-0.751***<br>[-1.290, -0.212]	-0.772***<br>[-1.314, -0.229]	-0.833**<br>[-1.522, -0.145]	-0.888***<br>[-1.378, -0.399]	-0.845**<br>[-1.535, -0.155]	-0.901***<br>[-1.391, -0.411]
HH_ETH (ref: Ekoi): Hausa	-0.703***<br>[-1.227, -0.179]	-0.703***<br>[-1.227, -0.179]	-0.722***<br>[-1.248, -0.196]	-0.649**<br>[-1.172, -0.125]	-0.677**<br>[-1.205, -0.150]	-1.106***<br>[-1.756, -0.455]	-0.673***<br>[-1.152, -0.195]	-1.131***<br>[-1.783, -0.479]	-0.686***<br>[-1.165, -0.207]
HH_ETH (ref: Ekoi): Ibibio	-0.468<br>[-1.030, 0.095]	-0.468<br>[-1.030, 0.095]	-0.458<br>[-1.022, 0.106]	-0.418<br>[-0.980, 0.144]	-0.419<br>[-0.983, 0.145]	-0.550<br>[-1.258, 0.159]	-0.492*<br>[-1.009, 0.026]	-0.537<br>[-1.247, 0.173]	-0.489*<br>[-1.007, 0.029]
HH_ETH (ref: Ekoi): Igala	-0.637**<br>[-1.266, -0.008]	-0.637**<br>[-1.266, -0.009]	-0.639**<br>[-1.268, -0.009]	-0.595*<br>[-1.222, 0.032]	-0.594*<br>[-1.224, 0.036]	-0.651<br>[-1.495, 0.194]	-0.505*<br>[-1.080, 0.069]	-0.646<br>[-1.492, 0.200]	-0.510*<br>[-1.085, 0.065]
HH_ETH (ref: Ekoi): Igbo	-0.332<br>[-0.849, 0.185]	-0.332<br>[-0.849, 0.185]	-0.324<br>[-0.842, 0.195]	-0.291<br>[-0.806, 0.225]	-0.294<br>[-0.812, 0.225]	-0.737**<br>[-1.358, -0.116]	-0.382<br>[-0.859, 0.095]	-0.732**<br>[-1.354, -0.109]	-0.382<br>[-0.859, 0.095]
HH_ETH (ref: Ekoi): Ijaw / Izon	-0.545*<br>[-1.142, 0.053]	-0.545*<br>[-1.142, 0.053]	-0.548*<br>[-1.147, 0.052]	-0.512*<br>[-1.108, 0.084]	-0.507*<br>[-1.106, 0.092]	-1.211***<br>[-1.974, -0.448]	-0.417<br>[-0.964, 0.130]	-1.202***<br>[-1.966, -0.437]	-0.418<br>[-0.966, 0.129]
HH_ETH (ref: Ekoi): Kanuri / Beriberi	-0.751**<br>[-1.386, -0.116]	-0.751**<br>[-1.387, -0.116]	-0.765**<br>[-1.401, -0.128]	-0.678**<br>[-1.312, -0.043]	-0.729**<br>[-1.367, -0.091]	-1.423***<br>[-2.294, -0.552]	-0.809***<br>[-1.379, -0.240]	-1.450***<br>[-2.322, -0.578]	-0.821***<br>[-1.391, -0.251]
HH_ETH (ref: Ekoi): Tiv	-0.556*<br>[-1.178, 0.067]	-0.556*<br>[-1.178, 0.067]	-0.571*<br>[-1.195, 0.053]	-0.530*<br>[-1.151, 0.091]	-0.526*<br>[-1.150, 0.099]	-0.969**<br>[-1.750, -0.188]	-0.482*<br>[-1.052, 0.088]	-0.974**<br>[-1.757, -0.191]	-0.497*<br>[-1.067, 0.074]
HH_ETH (ref: Ekoi): Yoruba	-0.428<br>[-0.950, 0.094]	-0.428<br>[-0.950, 0.094]	-0.427<br>[-0.950, 0.097]	-0.386<br>[-0.907, 0.135]	-0.394<br>[-0.918, 0.130]	-0.630*<br>[-1.269, 0.009]	-0.371<br>[-0.853, 0.111]	-0.624*<br>[-1.265, 0.016]	-0.373<br>[-0.855, 0.110]
HH_ETH (ref: Ekoi): Other	-0.526**<br>[-1.020, -0.031]	-0.526**<br>[-1.021, -0.032]	-0.530**<br>[-1.026, -0.034]	-0.475*<br>[-0.969, 0.019]	-0.487*<br>[-0.984, 0.010]	-0.828***<br>[-1.426, -0.230]	-0.496**<br>[-0.952, -0.039]	-0.833***<br>[-1.432, -0.233]	-0.501**<br>[-0.958, -0.044]
HH_SIZ	-0.014**<br>[-0.027, -0.001]	-0.014**<br>[-0.027, -0.001]	-0.014**<br>[-0.027, -0.001]	-0.014**<br>[-0.027, -0.001]	-0.014**<br>[-0.027, -0.001]	-0.023**<br>[-0.043, -0.004]	-0.007<br>[-0.017, 0.004]	-0.024**<br>[-0.043, -0.004]	-0.007<br>[-0.017, 0.004]
HH_WEA	0.140***<br>[0.121, 0.160]	0.140***<br>[0.121, 0.160]	0.139***<br>[0.119, 0.159]	0.140***<br>[0.120, 0.159]	0.141***<br>[0.121, 0.161]	0.116***<br>[0.087, 0.145]	0.119***<br>[0.102, 0.136]	0.115***<br>[0.086, 0.144]	0.118***<br>[0.101, 0.135]
YEAR (ref: 2003): 2008	0.636***<br>[0.377, 0.894]	0.636***<br>[0.377, 0.894]	0.625***<br>[0.366, 0.885]	0.662**<br>[0.076, 1.248]	1.088<br>[-1.748, 3.924]	0.789***<br>[0.429, 1.149]	0.729***<br>[0.487, 0.971]	0.774***<br>[0.414, 1.135]	0.723***<br>[0.480, 0.965]
YEAR (ref: 2003): 2013	0.768***<br>[0.511, 1.025]	0.768***<br>[0.511, 1.025]	0.746***<br>[0.487, 1.005]	0.679**<br>[0.097, 1.261]	0.943<br>[-1.887, 3.773]	0.894***<br>[0.538, 1.250]	0.908***<br>[0.667, 1.148]	0.875***<br>[0.518, 1.233]	0.893***<br>[0.651, 1.134]
YEAR (ref: 2003): 2018	0.899***<br>[0.635, 1.163]	0.900***<br>[0.635, 1.164]	0.980***<br>[0.717, 1.243]	0.774**<br>[0.182, 1.367]	1.222<br>[-1.626, 4.069]	1.202***<br>[0.838, 1.567]	1.094***<br>[0.855, 1.333]	1.266***<br>[0.902, 1.629]	1.155***<br>[0.917, 1.393]
Constant	-5.446***<br>[-6.091, -4.801]	-5.445***<br>[-6.090, -4.800]	-5.111***<br>[-5.743, -4.479]	-4.829***<br>[-5.638, -4.021]	-5.451***<br>[-8.332, -2.571]	-1.929***<br>[-2.749, -1.108]	-5.572***<br>[-6.166, -4.979]	-1.635***<br>[-2.430, -0.839]	-5.271***<br>[-5.853, -4.690]
Multilevel variance parameter: Level 1	0.133***<br>[0.054, 0.211]	0.133***<br>[0.054, 0.212]	0.144***<br>[0.058, 0.231]	0.127***<br>[0.052, 0.202]	0.140***<br>[0.056, 0.223]	0.114***<br>[0.036, 0.192]	0.146***<br>[0.064, 0.227]	0.118***<br>[0.038, 0.197]	0.152***<br>[0.067, 0.237]
Multilevel variance parameter: Level 2	0.177***<br>[0.122, 0.233]	0.177***<br>[0.122, 0.233]	0.176***<br>[0.120, 0.231]	0.174***<br>[0.119, 0.229]	0.174***<br>[0.119, 0.229]	0.165***<br>[0.086, 0.244]	0.225***<br>[0.168, 0.283]	0.164***<br>[0.085, 0.243]	0.222***<br>[0.166, 0.279]
Level 1 Observations (child)	24381	24381	24381	24381	24381	6514	31805	6514	31805
Level 2 Observations (LGA)	684	684	684	684	684	622	687	622	687
Level 3 Observations (state)	37	37	37	37	37	37	37	37	37
Akaike Information Criterion	17947.458	17949.558	17969.372	17988.064	17966.35	7940.803	23768.277	7947.817	23790.225
Prob. > X^2	<0.001	<0.001	<0.001	<0.001	<0.001	<0.001	<0.001	<0.001	<0.001

95% confidence intervals in brackets

* p < 0.10, ** p < 0.05, *** p < 0.01
