# Supplementary material for: Impact and effect mechanisms of mass campaigns in resource-constrained health systems: quasi-experimental evidence from polio eradication in Nigeria
Source: BMJ Glob Health. 2021 Mar 8;6(3):e004248. doi: 10.1136/bmjgh-2020-004248 (PMC7942242; doi:10.1136/bmjgh-2020-004248)
Supplement: Supplementary data [file bmjgh-2020-004248supp005.pdf]

Table 4: Main results: Link between SIA exposure and maternal health service uptake

Dependent variables: various indicators of maternal healthcare access (see right)	Delivery			Antenatal care	
	At home	At private facility	At public facility	No. of antenatal care visits	No. of tetanus injections
EXP_PREG	0.007<br>[-0.013, 0.027]	-0.065***<br>[-0.093, -0.036]	0.010<br>[-0.009, 0.029]	-0.025*<br>[-0.054, 0.003]	0.001<br>[-0.008, 0.009]
Level 1 Observations (child)	34713	34713	34713	35019	36585
Level 2 Observations (LGA)	686	686	686	686	687
Akaike Information Criterion	26422.845	17601.384	30318.818	193640.288	111227.001
Prob. > X^2	<0.001	<0.001	<0.001	<0.001	<0.001

95% confidence intervals in brackets

* p < 0.10, ** p < 0.05, *** p < 0.01

Table 4a: Detailed results: Link between SIA exposure and maternal health service uptake

Dependent variables: various indicators of maternal healthcare access (see right)	Main results					Year-Interaction models					Robustness checks: reporting (date approximation)				
	Delivery			Antenatal care		Delivery			Antenatal care		Delivery			Antenatal care	
	At home	At private facility	At public facility	No. of antenatal care visits	No. of tetanus injections	At home	At private facility	At public facility	No. of antenatal care visits	No. of tetanus injections	At home	At private facility	At public facility	No. of antenatal care visits	No. of tetanus injections
EXP_PREG	0.007 [-0.013, 0.027]	-0.065*** [-0.093, -0.036]	0.010 [-0.009, 0.029]	-0.025* [-0.054, 0.003]	0.001 [-0.008, 0.009]	0.102 [-0.086, 0.289]	-0.094 [-0.332, 0.144]	0.005 [-0.177, 0.187]	0.027 [-0.239, 0.292]	-0.040 [-0.107, 0.028]	-0.004 [-0.019, 0.012]	-0.060*** [-0.084, -0.036]	0.016** [0.001, 0.031]	-0.013 [-0.033, 0.007]	0.001 [-0.005, 0.008]
EXPxYR [yr = 2008]						-0.064 [-0.253, 0.126]	0.013 [-0.230, 0.256]	-0.027 [-0.212, 0.157]	-0.065 [-0.337, 0.208]	0.009 [-0.060, 0.077]					
EXPxYR [yr = 2013]						-0.110 [-0.298, 0.078]	0.028 [-0.213, 0.268]	0.023 [-0.159, 0.206]	-0.130 [-0.401, 0.141]	0.037 [-0.032, 0.106]					
EXPxYR [yr = 2018]						-0.097 [-0.286, 0.092]	0.047 [-0.195, 0.289]	0.002 [-0.181, 0.185]	0.034 [-0.237, 0.304]	0.064* [-0.005, 0.133]					
MOT_ANC	-0.137*** [-0.146, -0.129]	0.057*** [0.049, 0.065]	0.062*** [0.055, 0.068]			-0.137*** [-0.146, -0.129]	0.057*** [0.049, 0.064]	0.062*** [0.055, 0.068]			-0.144*** [-0.151, -0.136]	0.060*** [0.053, 0.068]	0.063*** [0.057, 0.069]		
MOT_EDM	-0.065*** [-0.079, -0.050]	0.052*** [0.032, 0.073]	0.057*** [0.043, 0.071]	0.097*** [0.074, 0.120]	0.031*** [0.024, 0.038]	-0.065*** [-0.079, -0.050]	0.052*** [0.032, 0.073]	0.057*** [0.043, 0.071]	0.097*** [0.074, 0.120]	0.031*** [0.024, 0.038]	-0.066*** [-0.078, -0.053]	0.051*** [0.033, 0.069]	0.061*** [0.049, 0.073]	0.095*** [0.075, 0.115]	0.031*** [0.025, 0.037]
MOT_EDF	-0.030*** [-0.045, -0.016]	0.033*** [0.012, 0.054]	0.035*** [0.020, 0.049]	0.082*** [0.063, 0.102]	0.023*** [0.017, 0.029]	-0.030*** [-0.045, -0.016]	0.033*** [0.011, 0.054]	0.035*** [0.020, 0.049]	0.082*** [0.063, 0.101]	0.023*** [0.017, 0.029]	-0.028*** [-0.040, -0.015]	0.027*** [0.007, 0.046]	0.038*** [0.026, 0.050]	0.076*** [0.059, 0.092]	0.025*** [0.020, 0.031]
MOT_AWE	-0.913*** [-0.988, -0.838]	0.284*** [0.172, 0.397]	0.978*** [0.901, 1.055]	1.946*** [1.813, 2.078]	0.643*** [0.603, 0.683]	-0.911*** [-0.986, -0.835]	0.282*** [0.169, 0.394]	0.977*** [0.900, 1.054]	1.928*** [1.796, 2.060]	0.637*** [0.596, 0.677]	-0.903*** [-0.968, -0.837]	0.282*** [0.181, 0.383]	0.957*** [0.890, 1.023]	1.905*** [1.791, 2.019]	0.646*** [0.611, 0.681]
MOT_AGE	-0.002 [-0.007, 0.003]	0.001 [-0.005, 0.008]	0.002 [-0.002, 0.007]	0.011*** [0.005, 0.017]	0.000 [-0.001, 0.002]	-0.003 [-0.008, 0.002]	0.001 [-0.005, 0.008]	0.002 [-0.002, 0.007]	0.011*** [0.005, 0.017]	0.001 [-0.001, 0.002]	0.000 [-0.005, 0.004]	0.001 [-0.005, 0.007]	0.001 [-0.003, 0.005]	0.007** [0.001, 0.012]	0.000 [-0.002, 0.001]
HH_RUR	0.538*** [0.448, 0.628]	-0.374*** [-0.484, -0.264]	-0.325*** [-0.408, -0.241]	-0.588*** [-0.816, -0.360]	-0.156*** [-0.206, -0.105]	0.542*** [0.452, 0.632]	-0.376*** [-0.486, -0.266]	-0.329*** [-0.412, -0.245]	-0.585*** [-0.812, -0.359]	-0.159*** [-0.209, -0.108]	0.487*** [0.407, 0.567]	-0.341*** [-0.441, -0.241]	-0.300*** [-0.373, -0.226]	-0.579*** [-0.779, -0.380]	-0.160*** [-0.209, -0.112]
HH_REL (ref: Catholic): Other Christian	0.094 [-0.030, 0.217]	-0.097 [-0.219, 0.026]	0.012 [-0.097, 0.121]	-0.055 [-0.277, 0.167]	-0.046* [-0.099, 0.006]	0.092 [-0.031, 0.215]	-0.097 [-0.219, 0.026]	0.013 [-0.096, 0.122]	-0.060 [-0.283, 0.163]	-0.047* [-0.100, 0.006]	0.103* [-0.007, 0.213]	-0.150*** [-0.259, -0.040]	0.055 [-0.042, 0.152]	-0.108 [-0.310, 0.094]	-0.040 [-0.087, 0.008]
HH_REL (ref: Catholic): Islam	0.225*** [0.076, 0.375]	-0.242*** [-0.403, -0.081]	-0.025 [-0.159, 0.110]	-0.168 [-0.467, 0.130]	-0.101*** [-0.174, -0.028]	0.225*** [0.075, 0.374]	-0.242*** [-0.403, -0.081]	-0.026 [-0.161, 0.108]	-0.170 [-0.469, 0.130]	-0.100*** [-0.173, -0.027]	0.231*** [0.098, 0.365]	-0.289*** [-0.435, -0.144]	0.022 [-0.098, 0.142]	-0.234* [-0.501, 0.034]	-0.104*** [-0.171, -0.036]
HH_REL (ref: Catholic): Traditionalist	1.004*** [0.646, 1.362]	-0.911*** [-1.380, -0.442]	-0.475** [-0.838, -0.111]	-0.653*** [-1.130, -0.176]	-0.350*** [-0.480, -0.219]	1.004*** [0.646, 1.362]	-0.913*** [-1.382, -0.444]	-0.474** [-0.837, -0.111]	-0.665*** [-1.143, -0.187]	-0.354*** [-0.485, -0.223]	0.900*** [0.569, 1.231]	-0.866*** [-1.301, -0.430]	-0.406** [-0.741, -0.070]	-0.619*** [-1.087, -0.151]	-0.282*** [-0.407, -0.158]
HH_REL (ref: Catholic): Other	-0.257 [-1.016, 0.503]	0.019 [-1.254, 1.292]	0.387 [-0.373, 1.148]	-0.084 [-1.535, 1.366]	-0.142 [-0.681, 0.397]	-0.265 [-1.024, 0.495]	0.028 [-1.246, 1.302]	0.388 [-0.373, 1.149]	0.020 [-1.431, 1.471]	-0.115 [-0.655, 0.424]	-0.189 [-0.779, 0.401]	0.115 [-0.877, 1.107]	0.286 [-0.301, 0.874]	-0.783 [-1.829, 0.262]	-0.287 [-0.662, 0.087]
HH_ETH (ref: Ekoi): Fulani	0.672*** [0.239, 1.104]	-0.084 [-0.988, 0.821]	-0.765*** [-1.190, -0.340]	-0.741 [-2.013, 0.531]	-0.349*** [-0.591, -0.107]	0.639*** [0.206, 1.072]	-0.080 [-0.985, 0.825]	-0.731*** [-1.156, -0.306]	-0.813 [-2.103, 0.477]	-0.324*** [-0.566, -0.081]	0.710*** [0.314, 1.106]	-0.065 [-0.816, 0.687]	-0.808*** [-1.192, -0.425]	-0.646 [-1.867, 0.576]	-0.331*** [-0.565, -0.097]
HH_ETH (ref: Ekoi): Hausa	0.941*** [0.521, 1.360]	-0.596 [-1.459, 0.266]	-0.820*** [-1.231, -0.408]	-0.436 [-1.703, 0.830]	-0.254** [-0.493, -0.015]	0.908*** [0.488, 1.328]	-0.591 [-1.453, 0.271]	-0.787*** [-1.199, -0.374]	-0.506 [-1.790, 0.778]	-0.228* [-0.468, 0.012]	0.971*** [0.584, 1.358]	-0.811** [-1.537, -0.086]	-0.843*** [-1.216, -0.469]	-0.319 [-1.535, 0.896]	-0.228* [-0.459, 0.003]
HH_ETH (ref: Ekoi): Ibibio	0.378 [-0.094, 0.850]	0.562 [-0.336, 1.459]	-0.310 [-0.771, 0.151]	-0.197 [-1.463, 1.070]	-0.102 [-0.385, 0.182]	0.350 [-0.122, 0.823]	0.565 [-0.332, 1.463]	-0.281 [-0.742, 0.180]	-0.254 [-1.535, 1.027]	-0.081 [-0.366, 0.204]	0.538** [0.099, 0.977]	0.290 [-0.478, 1.058]	-0.449** [-0.871, -0.027]	-0.011 [-1.219, 1.196]	-0.113 [-0.385, 0.160]
HH_ETH (ref: Ekoi): Igala	-0.510* [-1.034, 0.015]	1.297*** [0.404, 2.190]	0.034 [-0.454, 0.522]	0.013 [-1.304, 1.329]	0.277* [-0.017, 0.571]	-0.545** [-1.070, -0.020]	1.301*** [0.408, 2.195]	0.069 [-0.419, 0.557]	-0.066 [-1.400, 1.268]	0.301** [0.006, 0.595]	-0.417* [-0.895, 0.061]	0.951** [0.195, 1.707]	0.037 [-0.405, 0.478]	0.266 [-1.004, 1.535]	0.297** [0.002, 0.591]
HH_ETH (ref: Ekoi): Igbo	-0.788*** [-1.203, -0.372]	1.877*** [1.046, 2.708]	-0.575*** [-0.979, -0.171]	1.258* [-0.025, 2.540]	0.120 [-0.113, 0.353]	-0.813*** [-1.229, -0.397]	1.881*** [1.050, 2.712]	-0.547*** [-0.952, -0.143]	1.201* [-0.099, 2.501]	0.141 [-0.092, 0.374]	-0.742*** [-1.129, -0.354]	1.545*** [0.846, 2.244]	-0.556*** [-0.925, -0.186]	1.273** [0.037, 2.509]	0.122 [-0.104, 0.348]
HH_ETH (ref: Ekoi): Ijaw / Izon	0.887*** [0.401, 1.372]	0.122 [-0.804, 1.047]	-0.812*** [-1.287, -0.337]	-0.683 [-2.054, 0.688]	-0.133 [-0.397, 0.131]	0.863*** [0.377, 1.348]	0.122 [-0.803, 1.048]	-0.786*** [-1.261, -0.311]	-0.749 [-2.134, 0.636]	-0.120 [-0.383, 0.143]	0.841*** [0.395, 1.287]	-0.004 [-0.791, 0.783]	-0.828*** [-1.258, -0.397]	-0.583 [-1.910, 0.743]	-0.122 [-0.377, 0.133]
HH_ETH (ref: Ekoi): Kanuri / Beriberi	0.996*** [0.506, 1.487]	-0.539 [-1.680, 0.602]	-0.949*** [-1.430, -0.469]	-0.503 [-1.794, 0.787]	-0.239* [-0.504, 0.026]	0.961*** [0.469, 1.453]	-0.533 [-1.674, 0.609]	-0.912*** [-1.393, -0.430]	-0.591 [-1.896, 0.715]	-0.211 [-0.477, 0.054]	0.869*** [0.427, 1.312]	-0.450 [-1.377, 0.477]	-0.859*** [-1.288, -0.430]	-0.396 [-1.636, 0.843]	-0.241* [-0.492, 0.010]
HH_ETH (ref: Ekoi): Tiv	-0.776*** [-1.248, -0.304]	1.720*** [0.838, 2.601]	0.133 [-0.333, 0.600]	-0.896 [-2.238, 0.445]	-0.344** [-0.612, -0.075]	-0.809*** [-1.281, -0.337]	1.722*** [0.841, 2.604]	0.164 [-0.303, 0.631]	-0.968 [-2.326, 0.389]	-0.321** [-0.588, -0.054]	-0.651*** [-1.089, -0.213]	1.405*** [0.657, 2.152]	0.111 [-0.313, 0.535]	-0.698 [-1.982, 0.586]	-0.290** [-0.541, -0.038]
HH_ETH (ref: Ekoi): Yoruba	-0.160 [-0.582, 0.261]	1.295*** [0.459, 2.132]	-0.418** [-0.827, -0.009]	2.233*** [0.948, 3.518]	0.060 [-0.175, 0.295]	-0.189 [-0.611, 0.233]	1.300*** [0.463, 2.136]	-0.388* [-0.797, 0.022]	2.177*** [0.874, 3.480]	0.078 [-0.157, 0.313]	-0.116 [-0.508, 0.276]	0.960*** [0.256, 1.664]	-0.374** [-0.748, -0.000]	2.208*** [0.976, 3.439]	0.052 [-0.177, 0.281]
HH_ETH (ref: Ekoi): Other	0.206 [-0.196, 0.607]	0.905** [0.076, 1.734]	-0.350* [-0.744, 0.044]	0.035 [-1.215, 1.284]	-0.018 [-0.251, 0.214]	0.174 [-0.229, 0.576]	0.909** [0.080, 1.738]	-0.318 [-0.713, 0.077]	-0.035 [-1.303, 1.232]	0.005 [-0.228, 0.238]	0.263 [-0.110, 0.636]	0.642* [-0.054, 1.338]	-0.371** [-0.731, -0.011]	0.132 [-1.070, 1.333]	-0.006 [-0.232, 0.219]
HH_SIZ	0.032*** [0.023, 0.042]	-0.017** [-0.032, -0.003]	-0.020*** [-0.030, -0.011]	-0.043*** [-0.056, -0.030]	-0.006*** [-0.010, -0.002]	0.033*** [0.023, 0.042]	-0.017** [-0.032, -0.002]	-0.020*** [-0.030, -0.011]	-0.043*** [-0.057, -0.030]	-0.006*** [-0.010, -0.002]	0.035*** [0.027, 0.044]	-0.021*** [-0.034, -0.008]	-0.023*** [-0.031, -0.015]	-0.038*** [-0.049, -0.027]	-0.005*** [-0.009, -0.002]
HH_WEA	-0.207*** [-0.223, -0.190]	0.120*** [0.099, 0.141]	0.130*** [0.114, 0.145]	0.286*** [0.256, 0.317]	0.058*** [0.050, 0.067]	-0.207*** [-0.224, -0.190]	0.120*** [0.099, 0.141]	0.129*** [0.114, 0.145]	0.288*** [0.258, 0.319]	0.059*** [0.051, 0.067]	-0.207*** [-0.222, -0.193]	0.128*** [0.110, 0.147]	0.129*** [0.116, 0.142]	0.261*** [0.235, 0.288]	0.053*** [0.046, 0.060]
YEAR (ref: 2003): 2008	0.013 [-0.168, 0.193]	-0.240** [-0.450, -0.031]	0.079 [-0.094, 0.251]	-0.634*** [-1.029, -0.238]	-0.100** [-0.188, -0.013]	0.146 [-0.348, 0.640]	-0.266 [-0.840, 0.309]	0.162 [-0.312, 0.636]	-0.459 [-1.284, 0.366]	-0.094 [-0.286, 0.098]	0.035 [-0.130, 0.200]	-0.236** [-0.434, -0.038]	0.064 [-0.094, 0.223]	-0.542*** [-0.912, -0.172]	-0.089* [-0.180, 0.001]
YEAR (ref: 2003): 2013	-0.025 [-0.204, 0.155]	-0.388*** [-0.594, -0.183]	0.143* [-0.027, 0.314]	-0.258 [-0.668, 0.153]	-0.024 [-0.113, 0.065]	0.263 [-0.228, 0.755]	-0.449 [-1.018, 0.121]	0.064 [-0.405, 0.534]	0.186 [-0.638, 1.011]	-0.112 [-0.303, 0.078]	-0.021 [-0.185, 0.142]	-0.355*** [-0.549, -0.161]	0.116 [-0.039, 0.272]	-0.141 [-0.528, 0.246]	-0.001 [-0.092, 0.090]
YEAR (ref: 2003): 2018	-0.247*** [-0.433, -0.061]	-0.401*** [-0.620, -0.182]	0.354*** [0.178, 0.531]	-1.242*** [-1.651, -0.834]	-0.046 [-0.139, 0.048]	-0.011 [-0.505, 0.482]	-0.498* [-1.071, 0.074]	0.350 [-0.122, 0.821]	-1.370*** [-2.184, -0.556]	-0.217** [-0.408, -0.027]	-0.282*** [-0.443, -0.120]	-0.412*** [-0.608, -0.216]	0.379*** [0.225, 0.534]	-0.998*** [-1.377, -0.620]	-0.009 [-0.103, 0.084]
Constant	2.049*** [1.575, 2.524]	-3.635*** [-4.523, -2.747]	-2.439*** [-2.903, -1.975]	3.562*** [2.191, 4.933]	0.987*** [0.723, 1.251]	1.851*** [1.200, 2.503]	-3.572*** [-4.602, -2.542]	-2.455*** [-3.088, -1.821]	3.498*** [1.962, 5.033]	1.065*** [0.745, 1.386]	2.011*** [1.575, 2.448]	-3.405*** [-4.160, -2.650]	-2.453*** [-2.874, -2.032]	3.596*** [2.289, 4.904]	0.976*** [0.724, 1.228]
Multilevel variance parameter: Level 1	0.449*** [0.362, 0.536]	0.726*** [0.565, 0.888]	0.462*** [0.383, 0.542]	0.683*** [0.597, 0.769]	-1.428*** [-1.525, -1.330]	0.445*** [0.359, 0.531]	0.723*** [0.562, 0.884]	0.460*** [0.381, 0.539]	0.672*** [0.585, 0.759]	-1.456*** [-1.554, -1.359]	0.501*** [0.414, 0.589]	0.767*** [0.603, 0.931]	0.460*** [0.387, 0.533]	0.675*** [0.595, 0.756]	-1.405*** [-1.498, -1.313]
Multilevel variance parameter: Residual				1.322*** [1.282, 1.361]	0.090*** [0.064, 0.116]				1.321*** [1.282, 1.361]	0.090*** [0.064, 0.115]				1.281*** [1.243, 1.319]	0.085*** [0.063, 0.107]
Level 1 Observations (child)	34713	34713	34713	35019	36585	34713	34713	34713	35019	36585	45437	45437	45437	45839	47644
Level 2 Observations (LGA)	686	686	686	686	687	686	686	686	686	687	689	689	689	689	690
Akaike Information Criterion	26422.845	17601.384	30318.818	193640.288	111227.001	26423.667	17606.418	30319.207	193610.157	111190.932	34655.448	21928.17	39661.692	249499.916	144291.105
Prob. > X^2	<0.001	<0.001	<0.001	<0.001	<0.001	<0.001	<0.001	<0.001	<0.001	<0.001	<0.001	<0.001	<0.001	<0.001	<0.001

95% confidence intervals in brackets

* p < 0.10, ** p < 0.05, *** p < 0.01

Table 4b: Robustness check (3-level models): Link between SIA exposure and maternal health service uptake

Dependent variables: various indicators of maternal care and child survival (see right)	Main results					Year-Interaction models					Robustness checks: reporting				
	Delivery			Antenatal care		Delivery			Antenatal care		Delivery			Antenatal care	
	At home	At private facility	At public facility	No. of antenatal care visits	No. of tetanus injections	At home	At private facility	At public facility	No. of antenatal care visits	No. of tetanus injections	At home	At private facility	At public facility	No. of antenatal care visits	No. of tetanus injections
EXP_PREG	-0.014	0.001	0.012	0.025	0.012**	0.056	0.035	0.014	0.076	-0.020	-0.018**	-0.002	0.017**	0.017	0.008*
	[-0.036, 0.007]	[-0.029, 0.030]	[-0.008, 0.031]	[-0.012, 0.063]	[0.003, 0.021]	[-0.130, 0.243]	[-0.207, 0.276]	[-0.169, 0.197]	[-0.178, 0.330]	[-0.096, 0.055]	[-0.035, -0.002]	[-0.026, 0.023]	[0.001, 0.032]	[-0.013, 0.047]	[-0.000, 0.016]
EXPxYR [yr = 2008]						-0.044	-0.033	-0.036	-0.064	0.000					
						[-0.232, 0.145]	[-0.278, 0.212]	[-0.221, 0.149]	[-0.337, 0.209]	[-0.076, 0.076]					
EXPxYR [yr = 2013]						-0.087	-0.040	0.018	-0.128	0.030					
						[-0.274, 0.100]	[-0.282, 0.203]	[-0.165, 0.201]	[-0.388, 0.133]	[-0.047, 0.107]					
EXPxYR [yr = 2018]						-0.068	-0.026	-0.008	0.034	0.052					
						[-0.255, 0.120]	[-0.271, 0.219]	[-0.192, 0.176]	[-0.223, 0.292]	[-0.027, 0.132]					
MOT_ANC	-0.133***	0.048***	0.063***			-0.133***	0.048***	0.063***			-0.139***	0.052***	0.064***		
	[-0.141, -0.124]	[0.040, 0.056]	[0.056, 0.069]			[-0.141, -0.125]	[0.040, 0.056]	[0.056, 0.069]			[-0.147, -0.132]	[0.045, 0.059]	[0.058, 0.070]		
MOT_EDM	-0.062***	0.041***	0.057***	0.091***	0.029***	-0.062***	0.041***	0.057***	0.091***	0.029***	-0.063***	0.040***	0.061***	0.090***	0.029***
	[-0.077, -0.047]	[0.020, 0.061]	[0.043, 0.071]	[0.068, 0.113]	[0.020, 0.038]	[-0.077, -0.047]	[0.020, 0.061]	[0.043, 0.071]	[0.069, 0.113]	[0.020, 0.038]	[-0.076, -0.051]	[0.021, 0.058]	[0.049, 0.073]	[0.070, 0.109]	[0.021, 0.038]
MOT_EDF	-0.029***	0.024**	0.034***	0.081***	0.022***	-0.029***	0.024**	0.034***	0.081***	0.022***	-0.026***	0.017*	0.037***	0.075***	0.024***
	[-0.044, -0.014]	[0.003, 0.046]	[0.020, 0.048]	[0.062, 0.100]	[0.013, 0.030]	[-0.044, -0.014]	[0.003, 0.045]	[0.020, 0.048]	[0.062, 0.100]	[0.013, 0.030]	[-0.039, -0.014]	[-0.003, 0.036]	[0.025, 0.050]	[0.054, 0.096]	[0.017, 0.032]
MOT_AWE	-0.910***	0.232***	0.972***	1.927***	0.633***	-0.909***	0.232***	0.971***	1.908***	0.628***	-0.900***	0.236***	0.948***	1.894***	0.637***
	[-0.986, -0.835]	[0.120, 0.344]	[0.895, 1.049]	[1.682, 2.172]	[0.572, 0.694]	[-0.985, -0.834]	[0.120, 0.344]	[0.894, 1.049]	[1.662, 2.153]	[0.565, 0.690]	[-0.966, -0.834]	[0.136, 0.337]	[0.882, 1.015]	[1.665, 2.123]	[0.586, 0.688]
MOT_AGE	-0.003	-0.001	0.003	0.009***	0.000	-0.003	-0.001	0.003	0.009***	0.000	-0.001	-0.001	0.001	0.005*	0.000
	[-0.008, 0.002]	[-0.008, 0.006]	[-0.002, 0.008]	[0.003, 0.015]	[-0.002, 0.002]	[-0.008, 0.002]	[-0.008, 0.006]	[-0.002, 0.008]	[0.004, 0.015]	[-0.002, 0.002]	[-0.005, 0.004]	[-0.007, 0.005]	[-0.003, 0.005]	[-0.000, 0.011]	[-0.003, 0.002]
HH_RUR	0.528***	-0.345***	-0.335***	-0.514***	-0.142***	0.532***	-0.345***	-0.340***	-0.510***	-0.145***	0.474***	-0.309***	-0.313***	-0.511***	-0.149***
	[0.439, 0.618]	[-0.453, -0.238]	[-0.419, -0.251]	[-0.778, -0.249]	[-0.205, -0.080]	[0.442, 0.622]	[-0.453, -0.237]	[-0.424, -0.256]	[-0.776, -0.244]	[-0.209, -0.082]	[0.395, 0.554]	[-0.405, -0.213]	[-0.387, -0.239]	[-0.730, -0.293]	[-0.207, -0.091]
HH_REL (ref: Catholic): Other Christian	0.031	-0.023	0.014	-0.071	-0.031	0.030	-0.023	0.015	-0.076	-0.031	0.053	-0.085	0.055	-0.116	-0.028
	[-0.093, 0.156]	[-0.145, 0.099]	[-0.095, 0.124]	[-0.288, 0.146]	[-0.084, 0.022]	[-0.095, 0.154]	[-0.146, 0.099]	[-0.094, 0.125]	[-0.295, 0.143]	[-0.084, 0.021]	[-0.059, 0.164]	[-0.194, 0.023]	[-0.043, 0.153]	[-0.336, 0.104]	[-0.075, 0.019]
HH_REL (ref: Catholic): Islam	0.148*	-0.079	-0.021	-0.042	-0.040	0.147*	-0.079	-0.023	-0.045	-0.042	0.161**	-0.129*	0.021	-0.134	-0.054
	[-0.006, 0.301]	[-0.242, 0.084]	[-0.158, 0.116]	[-0.433, 0.349]	[-0.107, 0.027]	[-0.006, 0.301]	[-0.242, 0.084]	[-0.160, 0.114]	[-0.439, 0.348]	[-0.109, 0.025]	[0.024, 0.297]	[-0.275, 0.018]	[-0.101, 0.143]	[-0.491, 0.224]	[-0.126, 0.019]
HH_REL (ref: Catholic): Traditionalist	0.951***	-0.902***	-0.456**	-0.555**	-0.308***	0.951***	-0.904***	-0.455**	-0.567**	-0.313***	0.855***	-0.852***	-0.394**	-0.543**	-0.247***
	[0.590, 1.312]	[-1.367, -0.437]	[-0.819, -0.094]	[-1.011, -0.098]	[-0.423, -0.193]	[0.590, 1.311]	[-1.369, -0.438]	[-0.817, -0.093]	[-1.024, -0.109]	[-0.428, -0.197]	[0.523, 1.187]	[-1.284, -0.420]	[-0.729, -0.059]	[-1.031, -0.054]	[-0.375, -0.119]
HH_REL (ref: Catholic): Other	-0.389	0.218	0.409	0.037	-0.087	-0.389	0.220	0.407	0.149	-0.063	-0.306	0.306	0.305	-0.590	-0.231*
	[-1.143, 0.365]	[-1.057, 1.493]	[-0.350, 1.169]	[-1.052, 1.125]	[-0.512, 0.338]	[-1.143, 0.365]	[-1.056, 1.496]	[-0.353, 1.166]	[-0.923, 1.220]	[-0.476, 0.350]	[-0.894, 0.282]	[-0.687, 1.299]	[-0.284, 0.893]	[-1.351, 0.171]	[-0.486, 0.023]
HH_ETH (ref: Ekoi): Fulani	0.824***	-0.200	-0.744***	-0.608**	-0.280***	0.788***	-0.205	-0.704***	-0.687**	-0.256***	0.841***	-0.160	-0.785***	-0.576**	-0.282***
	[0.369, 1.279]	[-1.163, 0.764]	[-1.189, -0.300]	[-1.110, -0.106]	[-0.405, -0.155]	[0.332, 1.244]	[-1.168, 0.759]	[-1.149, -0.258]	[-1.223, -0.151]	[-0.388, -0.125]	[0.426, 1.256]	[-0.962, 0.642]	[-1.186, -0.384]	[-1.075, -0.078]	[-0.405, -0.158]
HH_ETH (ref: Ekoi): Hausa	0.979***	-0.399	-0.748***	-0.134	-0.115**	0.945***	-0.403	-0.708***	-0.211	-0.092*	0.995***	-0.610	-0.763***	-0.124	-0.124**
	[0.532, 1.426]	[-1.328, 0.531]	[-1.183, -0.313]	[-0.634, 0.366]	[-0.217, -0.013]	[0.497, 1.393]	[-1.332, 0.526]	[-1.144, -0.272]	[-0.731, 0.310]	[-0.198, 0.014]	[0.586, 1.404]	[-1.393, 0.172]	[-1.157, -0.369]	[-0.601, 0.354]	[-0.231, -0.017]
HH_ETH (ref: Ekoi): Ibibio	0.427*	0.030	-0.243	-0.170	-0.099	0.397	0.028	-0.209	-0.228	-0.075	0.596***	-0.198	-0.381*	-0.025	-0.135
	[-0.063, 0.917]	[-0.920, 0.980]	[-0.721, 0.234]	[-0.746, 0.405]	[-0.352, 0.153]	[-0.093, 0.888]	[-0.922, 0.979]	[-0.687, 0.269]	[-0.835, 0.379]	[-0.327, 0.176]	[0.144, 1.047]	[-1.009, 0.612]	[-0.817, 0.055]	[-0.417, 0.367]	[-0.329, 0.058]
HH_ETH (ref: Ekoi): Igala	0.105	0.377	-0.091	-0.114	0.288**	0.068	0.373	-0.048	-0.199	0.312**	0.086	0.108	-0.018	0.101	0.304**
	[-0.446, 0.657]	[-0.572, 1.327]	[-0.604, 0.423]	[-0.505, 0.278]	[0.044, 0.532]	[-0.484, 0.621]	[-0.577, 1.323]	[-0.562, 0.465]	[-0.610, 0.212]	[0.071, 0.554]	[-0.411, 0.582]	[-0.694, 0.909]	[-0.480, 0.444]	[-0.276, 0.477]	[0.033, 0.576]
HH_ETH (ref: Ekoi): Igbo	-0.358	0.818*	-0.431*	0.755***	0.020	-0.388*	0.816*	-0.395*	0.694***	0.044	-0.355*	0.595	-0.390*	0.772***	0.016
	[-0.813, 0.097]	[-0.078, 1.713]	[-0.862, 0.001]	[0.284, 1.225]	[-0.080, 0.120]	[-0.843, 0.068]	[-0.080, 1.711]	[-0.828, 0.037]	[0.188, 1.199]	[-0.052, 0.140]	[-0.774, 0.065]	[-0.159, 1.350]	[-0.783, 0.003]	[0.399, 1.145]	[-0.077, 0.109]
HH_ETH (ref: Ekoi): Ijaw / Izon	0.857***	-0.265	-0.705***	-0.772*	-0.083	0.829***	-0.266	-0.673***	-0.839**	-0.066	0.802***	-0.416	-0.684***	-0.731*	-0.080
	[0.336, 1.378]	[-1.253, 0.723]	[-1.212, -0.199]	[-1.550, 0.007]	[-0.259, 0.093]	[0.308, 1.351]	[-1.254, 0.722]	[-1.180, -0.167]	[-1.640, -0.038]	[-0.248, 0.115]	[0.329, 1.275]	[-1.254, 0.422]	[-1.141, -0.227]	[-1.484, 0.023]	[-0.247, 0.087]
HH_ETH (ref: Ekoi): Kanuri / Beriberi	1.104***	-0.411	-0.925***	-0.316	-0.153**	1.068***	-0.413	-0.882***	-0.410	-0.128*	0.944***	-0.240	-0.813***	-0.276	-0.169***
	[0.593, 1.616]	[-1.629, 0.808]	[-1.424, -0.426]	[-0.771, 0.138]	[-0.276, -0.030]	[0.555, 1.581]	[-1.632, 0.806]	[-1.382, -0.382]	[-0.915, 0.095]	[-0.257, 0.002]	[0.484, 1.404]	[-1.230, 0.751]	[-1.258, -0.367]	[-0.708, 0.156]	[-0.286, -0.052]
HH_ETH (ref: Ekoi): Tiv	-0.140	0.642	-0.010	-0.676***	-0.421***	-0.173	0.639	0.025	-0.750***	-0.399***	-0.082	0.460	-0.040	-0.570**	-0.358***
	[-0.647, 0.367]	[-0.304, 1.588]	[-0.511, 0.491]	[-1.181, -0.171]	[-0.581, -0.260]	[-0.681, 0.334]	[-0.307, 1.586]	[-0.477, 0.526]	[-1.281, -0.219]	[-0.564, -0.234]	[-0.549, 0.384]	[-0.343, 1.262]	[-0.493, 0.413]	[-1.059, -0.080]	[-0.492, -0.224]
HH_ETH (ref: Ekoi): Yoruba	0.331	0.348	-0.448**	1.415***	0.097*	0.298	0.346	-0.411*	1.348***	0.120**	0.327	0.103	-0.394*	1.495***	0.082
	[-0.123, 0.784]	[-0.551, 1.247]	[-0.883, -0.013]	[0.906, 1.924]	[-0.018, 0.212]	[-0.157, 0.752]	[-0.553, 1.245]	[-0.847, 0.025]	[0.827, 1.869]	[0.007, 0.232]	[-0.091, 0.745]	[-0.654, 0.860]	[-0.790, 0.003]	[0.975, 2.014]	[-0.029, 0.192]
HH_ETH (ref: Ekoi): Other	0.424*	0.273	-0.350*	0.085	0.029	0.390*	0.271	-0.312	0.011	0.053	0.434**	0.077	-0.349*	0.138	0.032
	[-0.000, 0.849]	[-0.614, 1.161]	[-0.765, 0.064]	[-0.250, 0.421]	[-0.053, 0.111]	[-0.036, 0.816]	[-0.617, 1.159]	[-0.727, 0.103]	[-0.370, 0.392]	[-0.032, 0.138]	[0.042, 0.826]	[-0.670, 0.823]	[-0.726, 0.028]	[-0.170, 0.445]	[-0.041, 0.105]
HH_SIZ	0.033***	-0.013*	-0.022***	-0.038***	-0.006***	0.033***	-0.013*	-0.022***	-0.039***	-0.006***	0.036***	-0.018***	-0.024***	-0.035***	-0.005***
	[0.023, 0.043]	[-0.028, 0.001]	[-0.031, -0.013]	[-0.054, -0.023]	[-0.010, -0.002]	[0.023, 0.043]	[-0.028, 0.001]	[-0.031, -0.012]	[-0.054, -0.023]	[-0.010, -0.002]	[0.027, 0.044]	[-0.031, -0.005]	[-0.032, -0.016]	[-0.046, -0.023]	[-0.009, -0.001]
HH_WEA	-0.210***	0.120***	0.133***	0.278***	0.057***	-0.210***	0.120***	0.132***	0.280***	0.057***	-0.211***	0.129***	0.132***	0.256***	0.051***
	[-0.227, -0.193]	[0.099, 0.141]	[0.117, 0.148]	[0.241, 0.314]	[0.047, 0.066]	[-0.227, -0.193]	[0.099, 0.141]	[0.117, 0.148]	[0.243, 0.317]	[0.048, 0.067]	[-0.225, -0.196]	[0.110, 0.147]	[0.119, 0.145]	[0.220, 0.291]	[0.044, 0.059]
YEAR (ref: 2003): 2008	0.064	-0.308***	0.077	-0.715***	-0.110**	0.153	-0.236	0.181	-0.538	-0.082	0.081	-0.304***	0.057	-0.625***	-0.099*
	[-0.114, 0.243]	[-0.514, -0.102]	[-0.096, 0.250]	[-1.142, -0.287]	[-0.213, -0.006]	[-0.338, 0.644]	[-0.812, 0.341]	[-0.295, 0.657]	[-1.384, 0.307]	[-0.295, 0.130]	[-0.083, 0.245]	[-0.499, -0.109]	[-0.102, 0.215]	[-1.057, -0.192]	[-0.207, 0.009]
YEAR (ref: 2003): 2013	0.026	-0.475***	0.136	-0.389*	-0.037	0.260	-0.384	0.064	0.051	-0.109	0.017	-0.437***	0.109	-0.254	-0.011
	[-0.152, 0.204]	[-0.678, -0.272]	[-0.035, 0.307]	[-0.827, 0.050]	[-0.150, 0.076]	[-0.228, 0.748]	[-0.956, 0.187]	[-0.408, 0.536]	[-0.779, 0.880]	[-0.306, 0.088]	[-0.146, 0.180]	[-0.629, -0.246]	[-0.047, 0.265]	[-0.686, 0.179]	[-0.130, 0.107]
YEAR (ref: 2003): 2018	-0.189**	-0.468***	0.342***	-1.296***	-0.046	-0.027	-0.408	0.362	-1.422***	-0.186*	-0.232***	-0.456***	0.363***	-1.065***	-0.012
	[-0.374, -0.005]	[-0.684, -0.252]	[0.165, 0.519]	[-1.916, -0.676]	[-0.207, 0.115]	[-0.518, 0.464]	[-0.983, 0.167]	[-0.112, 0.836]	[-2.332, -0.511]	[-0.402, 0.029]	[-0.393, -0.072]	[-0.650, -0.263]	[0.207, 0.518]	[-1.660, -0.471]	[-0.174, 0.150]
Constant	1.852***	-3.405***	-2.482***	3.574***	0.910***	1.715***	-3.476***	-2.522***	3.511***	0.966***	1.834***	-3.202***	-2.497***	3.689***	0.929***
	[1.322, 2.382]	[-4.464, -2.346]	[-2.980, -1.984]	[2.722, 4.426]	[0.764, 1.055]	[1.024, 2.407]	[-4.657, -2.294]	[-3.182, -1.861]	[2.422, 4.599]	[0.730, 1.201]	[1.340, 2.328]	[-4.134, -2.269]	[-2.953, -2.040]	[2.816, 4.561]	[0.774, 1.085]
Multilevel variance parameter: Level 1	0.353***	2.194***	0.151***	0.731***	-1.488***	0.351***	2.191***	0.151***	0.722***	-1.522***	0.380***	2.175***	0.172***	0.698***	-1.534***
	[0.170, 0.536]	[1.041, 3.348]	[0.067, 0.235]	[0.524, 0.938]	[-1.757, -1.219]	[0.169, 0.534]	[1.038, 3.343]	[0.067, 0.236]	[0.515, 0.929]	[-1.797, -1.246]	[0.188, 0.571]	[1.070, 3.279]	[0.080, 0.264]	[0.491, 0.905]	[-1.791, -1.277]
Multilevel variance parameter: Level 2	0.256***	0.255***	0.337***	-0.047	-1.653***	0.254***	0.255***	0.334***	-0.057	-1.679***	0.266***	0.222***	0.321***	-0.073	-1.615***
	[0.197, 0.316]	[0.183, 0.328]	[0.271, 0.403]	[-0.276, 0.181]	[-1.789, -1.517]	[0.195, 0.314]	[0.183, 0.328]	[0.269, 0.400]	[-0.287, 0.173]	[-1.815, -1.542]	[0.211, 0.322]	[0.162, 0.282]	[0.263, 0.378]	[-0.297, 0.151]	[-1.740, -1.490]
Multilevel variance parameter: Residual				1.320***	0.088***				1.319***	0.088***				1.279***	0.083***
				[1.190, 1.449]	[0.040, 0.137]				[1.190, 1.448]	[0.040, 0.137]				[1.150, 1.409]	[0.038, 0.129]
Level 1 Observations (child)	34713	34713	34713	35019	36585	34713	34713	34713	35019	36585	45437	45437	45437	45839	47644
Level 2 Observations (LGA)	757	757	757	757	758	757	757	757	757	758	761	761	761	761	762
Level 3 Observations (state)	37	37	37	37	37	37	37	37	37	37	37	37	37	37	37
Akaike Information Criterion	26186.547	17119.846	30165.878	192900.905	111038.932	26188.211	17125.583	30165.157	192869.798	111007.527	34345.877	21317.031	39462.966	248646.722	144047.955
Prob. > X^2	<0.001	<0.001	<0.001	<0.001	<0.001	<0.001	<0.001	<0.001	<0.001	<0.001	<0.001	<0.001	<0.001	<0.001	<0.001

95% confidence intervals in brackets

* $p < 0.10$, ** $p < 0.05$, *** $p < 0.01$
