# Supplementary material for: Impact and effect mechanisms of mass campaigns in resource-constrained health systems: quasi-experimental evidence from polio eradication in Nigeria
Source: BMJ Glob Health. 2021 Mar 8;6(3):e004248. doi: 10.1136/bmjgh-2020-004248 (PMC7942242; doi:10.1136/bmjgh-2020-004248)
Supplement: Supplementary data [file bmjgh-2020-004248supp006.pdf]

Table 5: Main results: Link between SIA exposure and child survival

Dependent variable: Child survival	Exposure decomposition	Total exposure
EXP_PREG_nod (date approximation)	-0.012<br>[-0.034, 0.010]	
EXP_CHI_nod (date approximation)	-0.019***<br>[-0.029, -0.009]	
EXP_TOT_nod (total exposure, date approximation)		-0.018***<br>[-0.027, -0.009]
Level 1 Observations (child)	52431	52431
Level 2 Observations (LGA)	689	689
Akaike Information Criterion	23684.534	23682.853
Prob. > X^2	<0.001	<0.001

95% confidence intervals in brackets

* p < 0.10, ** p < 0.05, *** p < 0.01

Table 5a: Detailed results: Link between SIA exposure and child survival

Dependent variable: Child survival	Main results		
	Full model		Year-Interaction
	Exposure decomposition	Total exposure	Child survival (total exposure)
EXP_PREG_nod (date approximation)	-0.012<br>[-0.034, 0.010]		
EXP_CHI_nod (date approximation)	-0.019***<br>[-0.029, -0.009]		
EXP_TOT_nod (total exposure, date approximation)		-0.018***<br>[-0.027, -0.009]	-0.058<br>[-0.134, 0.019]
EXPxYR [yr = 2008]			0.031<br>[-0.044, 0.107]
EXPxYR [yr = 2013]			0.046<br>[-0.030, 0.121]
EXPxYR [yr = 2018]			0.031<br>[-0.045, 0.106]
CHI_AGE	-0.005<br>[-0.014, 0.005]	-0.005<br>[-0.014, 0.005]	-0.002<br>[-0.012, 0.008]
CHI_AGE2	0.000<br>[-0.000, 0.000]	0.000<br>[-0.000, 0.000]	0.000<br>[-0.000, 0.000]
CHI_ORD	-0.154***<br>[-0.176, -0.131]	-0.154***<br>[-0.176, -0.131]	-0.153***<br>[-0.176, -0.131]
CHI_SEX	0.213***<br>[0.141, 0.285]	0.213***<br>[0.141, 0.285]	0.213***<br>[0.141, 0.285]
MOT_ANC	-0.001<br>[-0.011, 0.009]	-0.001<br>[-0.011, 0.009]	-0.001<br>[-0.011, 0.009]
MOT_EDM	0.013<br>[-0.006, 0.032]	0.013<br>[-0.006, 0.032]	0.013<br>[-0.006, 0.032]
MOT_EDF	0.028***<br>[0.011, 0.045]	0.028***<br>[0.011, 0.045]	0.028***<br>[0.010, 0.045]
MOT_AGE	0.010**<br>[0.002, 0.018]	0.010**<br>[0.002, 0.018]	0.010**<br>[0.001, 0.018]
HH_RUR	-0.182***<br>[-0.284, -0.079]	-0.181***<br>[-0.284, -0.079]	-0.184***<br>[-0.286, -0.081]
HH_REL (ref: Catholic): Other Christian	-0.137<br>[-0.303, 0.028]	-0.138<br>[-0.303, 0.028]	-0.136<br>[-0.302, 0.029]
HH_REL (ref: Catholic): Islam	-0.072<br>[-0.267, 0.123]	-0.070<br>[-0.265, 0.124]	-0.065<br>[-0.260, 0.130]
HH_REL (ref: Catholic): Traditionalist	-0.092<br>[-0.464, 0.280]	-0.091<br>[-0.463, 0.281]	-0.099<br>[-0.471, 0.273]
HH_REL (ref: Catholic): Other	0.584<br>[-0.590, 1.757]	0.581<br>[-0.592, 1.755]	0.558<br>[-0.616, 1.732]
HH_ETH (ref: Ekoi): Fulani	-0.182<br>[-0.808, 0.444]	-0.178<br>[-0.803, 0.448]	-0.118<br>[-0.745, 0.510]
HH_ETH (ref: Ekoi): Hausa	-0.502<br>[-1.120, 0.116]	-0.497<br>[-1.114, 0.121]	-0.436<br>[-1.056, 0.183]
HH_ETH (ref: Ekoi): Ibibio	-0.457<br>[-1.138, 0.225]	-0.458<br>[-1.140, 0.223]	-0.422<br>[-1.104, 0.259]
HH_ETH (ref: Ekoi): Igala	-0.390<br>[-1.102, 0.322]	-0.389<br>[-1.101, 0.323]	-0.357<br>[-1.070, 0.355]
HH_ETH (ref: Ekoi): Igbo	-0.500<br>[-1.109, 0.109]	-0.501<br>[-1.110, 0.108]	-0.471<br>[-1.081, 0.139]
HH_ETH (ref: Ekoi): Ijaw / Izon	-0.242<br>[-0.896, 0.413]	-0.243<br>[-0.898, 0.412]	-0.204<br>[-0.859, 0.451]
HH_ETH (ref: Ekoi): Kanuri / Beriberi	-0.269<br>[-0.928, 0.389]	-0.264<br>[-0.922, 0.395]	-0.189<br>[-0.850, 0.472]
HH_ETH (ref: Ekoi): Tiv	-0.362<br>[-1.013, 0.290]	-0.359<br>[-1.010, 0.293]	-0.312<br>[-0.964, 0.341]
HH_ETH (ref: Ekoi): Yoruba	-0.021<br>[-0.642, 0.601]	-0.021<br>[-0.643, 0.600]	0.011<br>[-0.611, 0.633]
HH_ETH (ref: Ekoi): Other	-0.354<br>[-0.959, 0.251]	-0.351<br>[-0.956, 0.254]	-0.298<br>[-0.905, 0.308]
HH_SIZ	0.132***<br>[0.117, 0.146]	0.132***<br>[0.117, 0.146]	0.132***<br>[0.118, 0.146]
HH_WEA	0.017*<br>[-0.003, 0.037]	0.017<br>[-0.003, 0.037]	0.016<br>[-0.004, 0.036]
YEAR (ref: 2003): 2008	0.330***<br>[0.136, 0.524]	0.334***<br>[0.141, 0.528]	0.208<br>[-0.263, 0.680]
YEAR (ref: 2003): 2013	0.489***<br>[0.290, 0.689]	0.493***<br>[0.295, 0.692]	0.161<br>[-0.308, 0.629]
YEAR (ref: 2003): 2018	0.302***<br>[0.110, 0.494]	0.309***<br>[0.119, 0.500]	0.164<br>[-0.304, 0.633]
Constant	2.494***<br>[1.819, 3.169]	2.502***<br>[1.827, 3.177]	2.644***<br>[1.859, 3.428]
Multilevel variance parameter: Level 1	0.041***<br>[0.014, 0.068]	0.041***<br>[0.014, 0.068]	0.040***<br>[0.013, 0.067]
Level 1 Observations (child)	52431	52431	52431
Level 2 Observations (LGA)	689	689	689
Akaike Information Criterion	23684.534	23682.853	23676.199
Prob. > X^2	<0.001	<0.001	<0.001

95% confidence intervals in brackets

* p < 0.10, ** p < 0.05, *** p < 0.01

Table 5b: Robustness check (3-level models): Link between SIA exposure and child survival

Dependent variables: various indicators of maternal care and child survival (see right)	Main results		
	Full model		Year-Interaction
	Exposure decomposition	Total exposure	Child survival (total exposure)
EXP_PREG_nod (date approximation)	-0.013		
	[-0.035, 0.010]		
EXP_CHI_nod (date approximation)	-0.020***		
	[-0.031, -0.010]		
EXP_TOT_nod (total exposure, date approximation)		-0.019***	-0.062
		[-0.029, -0.009]	[-0.140, 0.015]
EXPxYR [yr = 2008]			0.033
			[-0.042, 0.109]
EXPxYR [yr = 2013]			0.048
			[-0.027, 0.124]
EXPxYR [yr = 2018]			0.034
			[-0.042, 0.110]
CHI_AGE	-0.004	-0.004	-0.001
	[-0.014, 0.006]	[-0.013, 0.006]	[-0.011, 0.009]
CHI_AGE2	0.000	0.000	0.000
	[-0.000, 0.000]	[-0.000, 0.000]	[-0.000, 0.000]
CHI_ORD	-0.154***	-0.154***	-0.154***
	[-0.177, -0.132]	[-0.177, -0.132]	[-0.176, -0.131]
CHI_SEX	0.212***	0.212***	0.212***
	[0.140, 0.284]	[0.140, 0.284]	[0.140, 0.284]
MOT_ANC	0.000	0.000	0.000
	[-0.010, 0.010]	[-0.010, 0.010]	[-0.010, 0.010]
MOT_EDM	0.014	0.014	0.014
	[-0.005, 0.033]	[-0.005, 0.033]	[-0.005, 0.033]
MOT_EDF	0.029***	0.029***	0.029***
	[0.012, 0.046]	[0.012, 0.046]	[0.012, 0.046]
MOT_AGE	0.010**	0.010**	0.010**
	[0.002, 0.019]	[0.002, 0.019]	[0.002, 0.019]
HH_RUR	-0.176***	-0.176***	-0.178***
	[-0.279, -0.073]	[-0.279, -0.073]	[-0.281, -0.075]
HH_REL (ref: Catholic): Other Christian	-0.128	-0.129	-0.127
	[-0.296, 0.039]	[-0.296, 0.038]	[-0.295, 0.040]
HH_REL (ref: Catholic): Islam	-0.089	-0.088	-0.083
	[-0.288, 0.109]	[-0.286, 0.110]	[-0.282, 0.115]
HH_REL (ref: Catholic): Traditionalist	-0.055	-0.054	-0.060
	[-0.428, 0.319]	[-0.428, 0.320]	[-0.434, 0.314]
HH_REL (ref: Catholic): Other	0.639	0.636	0.613
	[-0.535, 1.813]	[-0.538, 1.809]	[-0.561, 1.788]
HH_ETH (ref: Ekoi): Fulani	-0.001	0.003	0.071
	[-0.665, 0.663]	[-0.662, 0.667]	[-0.595, 0.737]
HH_ETH (ref: Ekoi): Hausa	-0.349	-0.345	-0.276
	[-1.006, 0.308]	[-1.002, 0.312]	[-0.935, 0.384]
HH_ETH (ref: Ekoi): Ibibio	-0.347	-0.349	-0.304
	[-1.064, 0.370]	[-1.066, 0.368]	[-1.022, 0.415]
HH_ETH (ref: Ekoi): Igala	-0.203	-0.202	-0.153
	[-0.969, 0.563]	[-0.968, 0.564]	[-0.921, 0.614]
HH_ETH (ref: Ekoi): Igbo	-0.353	-0.355	-0.316
	[-1.010, 0.305]	[-1.012, 0.303]	[-0.975, 0.342]
HH_ETH (ref: Ekoi): Ijaw / Izon	-0.056	-0.058	-0.011
	[-0.773, 0.660]	[-0.775, 0.658]	[-0.728, 0.707]
HH_ETH (ref: Ekoi): Kanuri / Beriberi	-0.230	-0.225	-0.146
	[-0.925, 0.464]	[-0.920, 0.469]	[-0.843, 0.551]
HH_ETH (ref: Ekoi): Tiv	-0.200	-0.198	-0.142
	[-0.911, 0.511]	[-0.909, 0.513]	[-0.855, 0.571]
HH_ETH (ref: Ekoi): Yoruba	0.047	0.046	0.087
	[-0.616, 0.709]	[-0.617, 0.709]	[-0.577, 0.751]
HH_ETH (ref: Ekoi): Other	-0.217	-0.215	-0.153
	[-0.861, 0.426]	[-0.859, 0.428]	[-0.798, 0.493]
HH_SIZ	0.132***	0.132***	0.133***
	[0.118, 0.146]	[0.118, 0.146]	[0.118, 0.147]
HH_WEA	0.014	0.014	0.014
	[-0.006, 0.035]	[-0.006, 0.034]	[-0.007, 0.034]
YEAR (ref: 2003): 2008	0.326***	0.331***	0.204
	[0.133, 0.519]	[0.139, 0.523]	[-0.266, 0.674]
YEAR (ref: 2003): 2013	0.495***	0.500***	0.155
	[0.295, 0.694]	[0.301, 0.699]	[-0.312, 0.623]
YEAR (ref: 2003): 2018	0.297***	0.305***	0.142
	[0.106, 0.488]	[0.115, 0.494]	[-0.326, 0.611]
Constant	2.353***	2.362***	2.505***
	[1.641, 3.065]	[1.650, 3.074]	[1.689, 3.322]
Multilevel variance parameter: Level 1	0.026**	0.026**	0.027**
	[0.005, 0.047]	[0.005, 0.047]	[0.005, 0.048]
Multilevel variance parameter: Level 2	0.020*	0.020	0.019
	[-0.004, 0.044]	[-0.004, 0.044]	[-0.005, 0.042]
Level 1 Observations (child)	52431	52431	52431
Level 2 Observations (LGA)	761	761	761
Level 3 Observations (state)	37	37	37
Akaike Information Criterion	23665.091	23663.461	23656.424
Prob. > X^2	<0.001	<0.001	<0.001

95% confidence intervals in brackets

* p < 0.10, ** p < 0.05, *** p < 0.01
