# Supplementary material for: Impact and effect mechanisms of mass campaigns in resource-constrained health systems: quasi-experimental evidence from polio eradication in Nigeria
Source: BMJ Glob Health. 2021 Mar 8;6(3):e004248. doi: 10.1136/bmjgh-2020-004248 (PMC7942242; doi:10.1136/bmjgh-2020-004248)
Supplement: Supplementary data [file bmjgh-2020-004248supp007.pdf]

Table 6: Relationship between health system performance and SIA frequency across regions in survey sample.

Indicator	Region					
	North Central	North East	North West	South East	South South	South West
Average total number of SIAs at LGA level	75.81	100.17	113.94	47.07	47.38	50.61
Full immunisation status (incl. polio)[a]	21.9%	11.7%	8.3%	40.6%	31.5%	33.4%
Child exposure to SIAs	9.11	13.01	15.06	4.78	4.71	5.34
Mother's exposure to SIAs in pregnancy	3.28	4.42	5.07	1.89	1.87	2.07
Mother's average no. of ANC visits	4.68	2.78	2.3	8.16	6.35	10.75
Average place of delivery: home	49.5%	80.1%	87.2%	17.8%	50.4%	23.2%
Average place of delivery: private facility	16.5%	1.5%	1.5%	45.5%	15.6%	29.3%
Average place of delivery: public facility	34.0%	18.4%	11.3%	36.8%	34.0%	47.4%
Average survival of children under 5 years	91.8%	89.6%	87.4%	91.9%	93.1%	94.1%
Mothers' average years of education	3.05	1.49	1.16	4.57	4.42	4.25
Average household wealth index	3.88	2.54	2.93	4.96	4.75	5.1

Notes. Derived from DHS data. Unweighted statistics aggregated firstly at LGA and secondly at regional level, comprising full sample of children born between Oct 2000 and Dec 2017. ANC = antenatal care; DHS = Demographic and Health Survey; LGA = local government area; SIA = Supplementary Immunisation Activity.

[a.] Full immunisation status for children aged between 10 and 60 months, including at least 1 dose of BCG, 3 doses of DPT, 1 dose of measles vaccine, and three doses of oral polio vaccine.

Table 6a: Additional robustness checks: exclusion of 2018 survey round to compensate for mop-up variability

Dependent variables: various indicators of routine immunisation, maternal care and child survival (see right)	**Non-polio full immunisation status**		**Delivery**			**Antenatal care**		**Child survival**
	Full model	Interaction model	At home	At private facility	At public facility	No. of antenatal care	No. of tetanus injections	Total exposure
EXP_CHI	-0.030***<br>[-0.044,-0.016]	0.011<br>[-0.015,0.036]						
EXPxAGE		-0.001***<br>[-0.001,-0.000]						
EXP_PREG			0.006<br>[-0.020,0.032]	-0.057***<br>[-0.091,-0.024]	0.004<br>[-0.020,0.029]	-0.020<br>[-0.060,0.021]	-0.003<br>[-0.014,0.007]	
EXP_TOT_nod (total exposure, date approximation)								-0.013**<br>[-0.024,-0.001]
CHI_AGE	0.033***<br>[0.026,0.039]	0.038***<br>[0.031,0.045]						-0.002<br>[-0.014,0.009]
CHI_AGE2								0.000<br>[-0.000,0.000]
CHI_ORD	-0.060***<br>[-0.088,-0.033]	-0.061***<br>[-0.088,-0.034]						-0.143***<br>[-0.170,-0.116]
CHI_SEX	-0.007<br>[-0.090,0.077]	-0.006<br>[-0.089,0.078]						0.245***<br>[0.157,0.334]
MOT_ANC	0.053***<br>[0.043,0.062]	0.053***<br>[0.044,0.062]	-0.133***<br>[-0.142,-0.125]	0.056***<br>[0.047,0.064]	0.061***<br>[0.054,0.068]			0.002<br>[-0.010,0.013]
MOT_EDM	0.069***<br>[0.049,0.089]	0.069***<br>[0.049,0.089]	-0.064***<br>[-0.081,-0.048]	0.057***<br>[0.035,0.080]	0.053***<br>[0.038,0.069]	0.105***<br>[0.079,0.130]	0.031***<br>[0.024,0.038]	0.018<br>[-0.006,0.041]
MOT_EDF	0.006<br>[-0.014,0.027]	0.007<br>[-0.013,0.028]	-0.028***<br>[-0.045,-0.012]	0.036***<br>[0.012,0.059]	0.031***<br>[0.015,0.047]	0.090***<br>[0.068,0.112]	0.022***<br>[0.015,0.029]	0.024**<br>[0.002,0.045]
MOT_AWE	2.955***<br>[2.821,3.089]	2.964***<br>[2.829,3.098]	-0.947***<br>[-1.029,-0.865]	0.289***<br>[0.166,0.411]	1.021***<br>[0.936,1.106]	1.900***<br>[1.754,2.047]	0.631***<br>[0.584,0.677]	
MOT_AGE	0.025***<br>[0.016,0.035]	0.026***<br>[0.016,0.035]	-0.002<br>[-0.007,0.004]	0.001<br>[-0.007,0.008]	0.002<br>[-0.003,0.007]	0.014***<br>[0.007,0.020]	0.002<br>[-0.000,0.004]	0.010**<br>[0.000,0.020]
HH_RUR	-0.074<br>[-0.191,0.044]	-0.076<br>[-0.194,0.042]	0.599***<br>[0.498,0.701]	-0.402***<br>[-0.526,-0.278]	-0.364***<br>[-0.458,-0.270]	-0.568***<br>[-0.836,-0.301]	-0.168***<br>[-0.226,-0.111]	-0.266***<br>[-0.395,-0.137]
HH_REL (ref: Catholic): Other Christian	-0.172**<br>[-0.329,-0.015]	-0.172**<br>[-0.330,-0.015]	0.106<br>[-0.029,0.242]	-0.042<br>[-0.180,0.096]	-0.050<br>[-0.172,0.071]	-0.102<br>[-0.348,0.144]	-0.037<br>[-0.096,0.022]	-0.050<br>[-0.245,0.144]
HH_REL (ref: Catholic): Islam	-0.504***<br>[-0.695,-0.313]	-0.522***<br>[-0.714,-0.330]	0.205**<br>[0.040,0.369]	-0.218**<br>[-0.397,-0.040]	-0.035<br>[-0.184,0.113]	-0.177<br>[-0.508,0.154]	-0.109***<br>[-0.189,-0.029]	0.025<br>[-0.204,0.254]
HH_REL (ref: Catholic): Traditionalist	-0.596***<br>[-1.025,-0.168]	-0.600***<br>[-1.029,-0.171]	1.053***<br>[0.680,1.426]	-0.863***<br>[-1.347,-0.380]	-0.553***<br>[-0.936,-0.171]	-0.589**<br>[-1.087,-0.091]	-0.360***<br>[-0.498,-0.221]	-0.069<br>[-0.457,0.318]
HH_REL (ref: Catholic): Other	-0.733<br>[-2.533,1.067]	-0.725<br>[-2.524,1.075]	-0.555<br>[-1.634,0.523]	0.570<br>[-0.794,1.933]	0.388<br>[-0.744,1.520]	0.096<br>[-2.104,2.295]	0.022<br>[-0.697,0.741]	-0.324<br>[-1.551,0.903]
HH_ETH (ref: Ekoi): Fulani	-1.040***<br>[-1.596,-0.484]	-1.092***<br>[-1.650,-0.535]	0.671***<br>[0.201,1.141]	-0.058<br>[-1.098,0.981]	-0.744***<br>[-1.207,-0.280]	-0.764<br>[-2.268,0.740]	-0.383***<br>[-0.655,-0.110]	-0.447<br>[-1.256,0.362]
HH_ETH (ref: Ekoi): Hausa	-0.926***<br>[-1.459,-0.393]	-1.012***<br>[-1.547,-0.477]	0.936***<br>[0.481,1.391]	-0.569<br>[-1.557,0.419]	-0.806***<br>[-1.254,-0.357]	-0.504<br>[-2.005,0.996]	-0.293**<br>[-0.563,-0.023]	-0.832**<br>[-1.632,-0.032]
HH_ETH (ref: Ekoi): Ibibio	-0.639**<br>[-1.224,-0.054]	-0.643**<br>[-1.229,-0.057]	0.296<br>[-0.220,0.811]	0.750<br>[-0.270,1.771]	-0.263<br>[-0.767,0.242]	-0.026<br>[-1.484,1.433]	-0.163<br>[-0.483,0.156]	-0.777*<br>[-1.650,0.095]
HH_ETH (ref: Ekoi): Igala	-0.699**<br>[-1.337,-0.061]	-0.704**<br>[-1.342,-0.065]	-0.532*<br>[-1.106,0.043]	1.440***<br>[0.423,2.456]	0.023<br>[-0.509,0.556]	-0.135<br>[-1.689,1.418]	0.284*<br>[-0.046,0.615]	-0.670<br>[-1.584,0.243]
HH_ETH (ref: Ekoi): Igbo	-0.542**<br>[-1.050,-0.035]	-0.546**<br>[-1.054,-0.037]	-0.852***<br>[-1.301,-0.403]	2.114***<br>[1.165,3.064]	-0.601***<br>[-1.039,-0.162]	1.096<br>[-0.409,2.602]	0.078<br>[-0.184,0.340]	-0.917**<br>[-1.704,-0.130]
HH_ETH (ref: Ekoi): Ijaw / Izon	-0.654**<br>[-1.242,-0.066]	-0.651**<br>[-1.240,-0.061]	0.819***<br>[0.289,1.350]	0.238<br>[-0.817,1.292]	-0.751***<br>[-1.271,-0.231]	-0.915<br>[-2.502,0.672]	-0.150<br>[-0.444,0.145]	-0.766*<br>[-1.592,0.059]
HH_ETH (ref: Ekoi): Kanuri / Beriberi	-1.146***<br>[-1.819,-0.474]	-1.232***<br>[-1.906,-0.557]	1.027***<br>[0.493,1.560]	-0.564<br>[-1.886,0.759]	-0.954***<br>[-1.477,-0.431]	-0.626<br>[-2.148,0.897]	-0.286*<br>[-0.579,0.007]	-0.628<br>[-1.471,0.215]
	-0.721**	-0.734**	-0.803***	1.933***	0.111	-1.066	-0.392**	-0.883**

HH_ETH (ref: Ekoi): Tiv	[-1.334,-0.108]	[-1.347,-0.120]	[-1.314,-0.292]	[0.928,2.937]	[-0.398,0.621]	[-2.636,0.504]	[-0.696,-0.088]	[-1.710,-0.056]
HH_ETH (ref: Ekoi): Yoruba	-0.650**	-0.660**	-0.172	1.441***	-0.422*	2.185***	0.061	-0.429
	[-1.168,-0.132]	[-1.179,-0.141]	[-0.628,0.283]	[0.485,2.397]	[-0.866,0.022]	[0.668,3.702]	[-0.204,0.327]	[-1.231,0.372]
HH_ETH (ref: Ekoi): Other	-0.727***	-0.759***	0.196	1.015**	-0.322	-0.039	-0.054	-0.723*
	[-1.226,-0.228]	[-1.260,-0.259]	[-0.238,0.630]	[0.066,1.963]	[-0.750,0.105]	[-1.520,1.442]	[-0.317,0.209]	[-1.506,0.060]
HH_SIZ	-0.015**	-0.015**	0.030***	-0.013	-0.019***	-0.049***	-0.008***	0.135***
	[-0.029,-0.001]	[-0.029,-0.001]	[0.019,0.041]	[-0.029,0.003]	[-0.030,-0.009]	[-0.064,-0.035]	[-0.013,-0.004]	[0.117,0.153]
HH_WEA	0.155***	0.156***	-0.213***	0.116***	0.138***	0.318***	0.066***	0.012
	[0.133,0.178]	[0.133,0.179]	[-0.232,-0.194]	[0.092,0.140]	[0.120,0.155]	[0.282,0.353]	[0.057,0.076]	[-0.013,0.038]
YEAR (ref: 2003): 2008	0.667***	0.601***	0.001	-0.246**	0.081	-0.658***	-0.098**	0.320***
	[0.402,0.932]	[0.333,0.869]	[-0.185,0.188]	[-0.462,-0.031]	[-0.097,0.259]	[-1.073,-0.242]	[-0.184,-0.013]	[0.123,0.517]
YEAR (ref: 2003): 2013	0.801***	0.720***	-0.030	-0.406***	0.146	-0.338	-0.036	0.464***
	[0.539,1.064]	[0.453,0.986]	[-0.216,0.156]	[-0.618,-0.194]	[-0.030,0.322]	[-0.769,0.094]	[-0.123,0.052]	[0.258,0.670]
Constant	-4.617***	-4.787***	2.050***	-3.869***	-2.399***	3.518***	1.007***	2.723***
	[-5.246,-3.989]	[-5.423,-4.151]	[1.540,2.561]	[-4.878,-2.861]	[-2.900,-1.897]	[1.918,5.118]	[0.716,1.299]	[1.871,3.576]
Multilevel variance parameter: Level 1	0.349***	0.348***	0.478***	0.789***	0.499***	0.793***	-1.384***	0.041**
	[0.265,0.434]	[0.264,0.432]	[0.381,0.576]	[0.603,0.975]	[0.407,0.590]	[0.696,0.890]	[-1.483,-1.285]	[0.007,0.075]
Multilevel variance parameter: Residual						1.333***	0.092***	
						[1.291,1.376]	[0.064,0.121]	
Level 1 Observations (child)	20752	20752	29480	29480	29480	29691	31177	34500
Level 2 Observations (LGA)	572	572	574	574	574	574	575	576
Akaike Information Criterion	14663.935	14651.865	21880.73	14443.993	24935.19	164998.875	94979.021	15648.893
Prob. > X^2	<0.001	<0.001	<0.001	<0.001	<0.001	<0.001	<0.001	<0.001

95% confidence intervals in brackets
* p < 0.10, ** p < 0.05, *** p < 0.01

Table 6b-1: Additional robustness checks: year-wise stratification to detect time trends - Polio

Dependent variable: non-polio full immunisation status	**Full model**				**Interaction model (EXPxAGE)**			
	2003	2008	2013	2018	2003	2008	2013	2018
EXP_CHI	-0.040	-0.051***	-0.026***	-0.027	-0.086	-0.042	0.027*	0.111*
	[-0.289,0.209]	[-0.079,-0.023]	[-0.043,-0.010]	[-0.065,0.010]	[-1.046,0.874]	[-0.093,0.009]	[-0.003,0.058]	[-0.012,0.233]
EXPxAGE					0.003	0.000	-0.001***	-0.005**
					[-0.051,0.057]	[-0.001,0.001]	[-0.002,-0.001]	[-0.009,-0.001]
CHI_AGE	0.117**	0.035***	0.035***	0.036***	0.109	0.036***	0.043***	0.042***
	[0.017,0.218]	[0.025,0.046]	[0.026,0.043]	[0.016,0.056]	[-0.087,0.305]	[0.024,0.049]	[0.033,0.052]	[0.021,0.062]
CHI_ORD	0.018	-0.037*	-0.076***	-0.069**	0.018	-0.037*	-0.076***	-0.070**
	[-0.153,0.188]	[-0.080,0.007]	[-0.113,-0.039]	[-0.129,-0.008]	[-0.153,0.188]	[-0.081,0.006]	[-0.113,-0.039]	[-0.131,-0.009]
CHI_SEX	0.031	0.010	-0.027	0.030	0.031	0.010	-0.025	0.029
	[-0.464,0.526]	[-0.126,0.145]	[-0.137,0.083]	[-0.146,0.206]	[-0.464,0.526]	[-0.126,0.145]	[-0.135,0.085]	[-0.147,0.205]
MOT_ANC	0.095***	0.061***	0.044***	0.074***	0.094***	0.061***	0.045***	0.075***
	[0.045,0.144]	[0.045,0.077]	[0.032,0.056]	[0.049,0.100]	[0.044,0.144]	[0.045,0.077]	[0.032,0.057]	[0.050,0.101]
MOT_EDM	0.074	0.064***	0.075***	0.041*	0.074	0.064***	0.076***	0.044*
	[-0.036,0.185]	[0.031,0.096]	[0.048,0.101]	[-0.003,0.085]	[-0.036,0.185]	[0.031,0.096]	[0.049,0.103]	[-0.000,0.088]
MOT_EDF	-0.035	0.026	-0.009	0.028	-0.035	0.026	-0.007	0.029
	[-0.159,0.088]	[-0.007,0.059]	[-0.037,0.018]	[-0.015,0.071]	[-0.159,0.088]	[-0.007,0.059]	[-0.035,0.020]	[-0.014,0.072]
MOT_AWE	2.968***	2.453***	3.411***	3.131***	2.971***	2.455***	3.422***	3.132***
	[1.880,4.056]	[2.255,2.650]	[3.217,3.605]	[2.779,3.484]	[1.881,4.061]	[2.257,2.652]	[3.228,3.615]	[2.779,3.484]
MOT_AGE	0.012	0.021***	0.027***	0.033***	0.012	0.021***	0.027***	0.034***
	[-0.046,0.070]	[0.006,0.037]	[0.014,0.040]	[0.012,0.054]	[-0.046,0.070]	[0.006,0.037]	[0.015,0.040]	[0.013,0.055]
HH_RUR	-0.056	-0.031	-0.168**	-0.232*	-0.054	-0.030	-0.176**	-0.235**
	[-0.680,0.569]	[-0.244,0.181]	[-0.321,-0.015]	[-0.467,0.002]	[-0.680,0.571]	[-0.243,0.182]	[-0.329,-0.022]	[-0.469,-0.001]
HH_REL (ref: Catholic): Other Christian	-0.189	-0.269**	-0.099	0.011	-0.188	-0.270**	-0.099	0.018
	[-0.895,0.517]	[-0.532,-0.007]	[-0.310,0.112]	[-0.320,0.342]	[-0.894,0.518]	[-0.532,-0.007]	[-0.311,0.112]	[-0.313,0.349]
HH_REL (ref: Catholic): Islam	-0.258	-0.540***	-0.474***	-0.432**	-0.257	-0.543***	-0.504***	-0.438**
	[-1.035,0.519]	[-0.864,-0.216]	[-0.729,-0.218]	[-0.848,-0.015]	[-1.034,0.520]	[-0.868,-0.219]	[-0.760,-0.248]	[-0.854,-0.022]
HH_REL (ref: Catholic): Traditionalist	-0.325	-0.481	-0.757**	-0.168	-0.322	-0.481	-0.774**	-0.105
	[-2.215,1.565]	[-1.089,0.126]	[-1.411,-0.104]	[-1.807,1.470]	[-2.212,1.568]	[-1.089,0.127]	[-1.428,-0.119]	[-1.755,1.545]
HH_REL (ref: Catholic): Other	0.000	-0.531	0.000	0.162	0.000	-0.526	0.000	0.191
	[0.000,0.000]	[-2.432,1.370]	[0.000,0.000]	[-1.319,1.642]	[0.000,0.000]	[-2.428,1.377]	[0.000,0.000]	[-1.290,1.671]
HH_ETH (ref: Ekoi): Fulani	0.000	-1.277***	0.058	-0.712	0.000	-1.289***	0.034	-0.771
	[0.000,0.000]	[-2.032,-0.522]	[-1.523,1.640]	[-2.074,0.650]	[0.000,0.000]	[-2.046,-0.531]	[-1.549,1.617]	[-2.133,0.591]
HH_ETH (ref: Ekoi): Hausa	-0.297	-1.227***	0.249	-0.635	-0.287	-1.245***	0.170	-0.691
	[-2.094,1.500]	[-1.956,-0.498]	[-1.314,1.812]	[-1.952,0.682]	[-2.098,1.524]	[-1.978,-0.511]	[-1.395,1.734]	[-2.008,0.626]
HH_ETH (ref: Ekoi): Ibibio	0.000	-0.898**	0.426	-0.712	0.000	-0.899**	0.469	-0.721
	[0.000,0.000]	[-1.695,-0.101]	[-1.174,2.027]	[-2.124,0.700]	[0.000,0.000]	[-1.697,-0.101]	[-1.134,2.071]	[-2.133,0.690]
HH_ETH (ref: Ekoi): Igala	-0.403	-1.279***	0.926	-1.062	-0.399	-1.284***	0.987	-1.073
	[-2.844,2.038]	[-2.159,-0.399]	[-0.719,2.571]	[-2.652,0.528]	[-2.844,2.046]	[-2.165,-0.404]	[-0.660,2.633]	[-2.664,0.519]
HH_ETH (ref: Ekoi): Igbo	0.510	-0.827**	0.569	-0.415	0.512	-0.828**	0.611	-0.433
	[-1.416,2.435]	[-1.481,-0.173]	[-0.981,2.120]	[-1.695,0.865]	[-1.418,2.442]	[-1.482,-0.174]	[-0.941,2.164]	[-1.712,0.847]
HH_ETH (ref: Ekoi): Ijaw / Izon	0.219	-1.426***	0.894	0.126	0.212	-1.425***	0.943	0.110
	[-2.879,3.317]	[-2.240,-0.611]	[-0.696,2.484]	[-1.326,1.578]	[-2.892,3.316]	[-2.239,-0.610]	[-0.649,2.535]	[-1.342,1.562]
HH_ETH (ref: Ekoi): Kanuri / Beriberi	0.000	-1.606***	0.072	-0.521	0.000	-1.622***	-0.019	-0.615
	[0.000,0.000]	[-2.589,-0.624]	[-1.584,1.727]	[-2.060,1.018]	[0.000,0.000]	[-2.607,-0.636]	[-1.677,1.640]	[-2.154,0.925]
HH_ETH (ref: Ekoi): Tiv	0.636	-0.966**	0.299	-0.453	0.640	-0.972**	0.347	-0.511
	[-1.631,2.903]	[-1.777,-0.155]	[-1.325,1.924]	[-1.861,0.955]	[-1.632,2.912]	[-1.784,-0.161]	[-1.280,1.973]	[-1.920,0.897]
HH_ETH (ref: Ekoi): Yoruba	1.185	-0.572*	0.193	-0.550	1.185	-0.576*	0.228	-0.566
	[-0.686,3.055]	[-1.252,0.108]	[-1.360,1.746]	[-1.852,0.753]	[-0.689,3.060]	[-1.256,0.105]	[-1.327,1.783]	[-1.868,0.736]
HH_ETH (ref: Ekoi): Other	0.094	-0.999***	0.430	-0.390	0.098	-1.008***	0.438	-0.448
	[-1.703,1.892]	[-1.651,-0.347]	[-1.113,1.973]	[-1.670,0.890]	[-1.705,1.902]	[-1.661,-0.354]	[-1.107,1.982]	[-1.728,0.832]
HH_SIZ	0.005	-0.026**	-0.009	-0.012	0.005	-0.026**	-0.008	-0.012
	[-0.080,0.090]	[-0.049,-0.004]	[-0.027,0.010]	[-0.043,0.019]	[-0.080,0.090]	[-0.049,-0.004]	[-0.027,0.011]	[-0.043,0.018]
HH_WEA	0.083	0.170***	0.156***	0.099***	0.083	0.170***	0.157***	0.101***
	[-0.035,0.201]	[0.130,0.209]	[0.127,0.185]	[0.056,0.142]	[-0.035,0.201]	[0.131,0.209]	[0.127,0.186]	[0.058,0.145]
Constant	-7.031***	-3.237***	-5.320***	-4.308***	-6.896***	-3.286***	-5.704***	-4.474***
	[-9.848,-4.214]	[-4.034,-2.439]	[-6.931,-3.709]	[-5.802,-2.814]	[-10.781,-3.011]	[-4.117,-2.454]	[-7.327,-4.081]	[-5.974,-2.973]
Multilevel variance parameter: Level 1	0.000	0.520***	0.295***	0.398***	0.000	0.520***	0.292***	0.396***
	[-0.000,0.000]	[0.347,0.692]	[0.198,0.392]	[0.200,0.596]	[-0.000,0.000]	[0.347,0.692]	[0.196,0.389]	[0.198,0.593]
Level 1 Observations (child)	648	8163	11924	3629	648	8163	11924	3629
Level 2 Observations (LGA)	138	369	370	504	138	369	370	504
Akaike Information Criterion	450.792	5772.355	8366.43	3393.899	452.782	5774.188	8351.201	3390.504
Prob. > X^2	<0.001	<0.001	<0.001	<0.001	<0.001	<0.001	<0.001	<0.001

95% confidence intervals in brackets
* p < 0.10, ** p < 0.05, *** p < 0.01

Table 6b-2: Additional robustness checks: year-wise stratification to detect time trends - delivery

Dependent variables: various indicators of delivery (see right)	At home 2003	At home 2008	At home 2013	At home 2018	At private facility 2003	At private facility 2008	At private facility 2013	At private facility 2018	At public facility 2003	At public facility 2008	At public facility 2013	At public facility 2018
EXP_PREG	0.104	0.004	0.019	0.023	-0.138	-0.065**	-0.060***	-0.108***	0.038	0.005	-0.005	0.014
	[-0.113,0.320]	[-0.042,0.050]	[-0.014,0.051]	[-0.012,0.058]	[-0.391,0.116]	[-0.123,-0.007]	[-0.103,-0.018]	[-0.160,-0.056]	[-0.176,0.252]	[-0.039,0.048]	[-0.035,0.026]	[-0.019,0.047]
MOT_ANC	-0.112***	-0.155***	-0.124***	-0.179***	0.029*	0.083***	0.049***	0.069***	0.071***	0.070***	0.054***	0.071***
	[-0.150,-0.075]	[-0.171,-0.140]	[-0.136,-0.113]	[-0.205,-0.152]	[-0.004,0.062]	[0.067,0.098]	[0.039,0.060]	[0.046,0.092]	[0.040,0.103]	[0.057,0.083]	[0.045,0.063]	[0.051,0.090]
MOT_EDM	-0.129***	-0.056***	-0.061***	-0.085***	0.076*	0.057***	0.056***	0.047*	0.102***	0.041***	0.055***	0.088***
	[-0.201,-0.058]	[-0.083,-0.028]	[-0.082,-0.040]	[-0.122,-0.047]	[-0.008,0.159]	[0.020,0.094]	[0.026,0.086]	[-0.004,0.098]	[0.030,0.174]	[0.014,0.068]	[0.034,0.075]	[0.053,0.124]
MOT_EDF	-0.012	-0.039***	-0.023**	-0.041**	0.029	0.027	0.046***	0.028	0.009	0.043***	0.024**	0.051***
	[-0.084,0.060]	[-0.066,-0.011]	[-0.045,-0.002]	[-0.078,-0.004]	[-0.061,0.119]	[-0.012,0.065]	[0.014,0.078]	[-0.027,0.082]	[-0.065,0.083]	[0.016,0.069]	[0.004,0.045]	[0.016,0.087]
MOT_AWE	-0.746***	-0.934***	-0.982***	-0.765***	0.229	0.327***	0.259***	0.238	0.848***	0.990***	1.075***	0.809***
	[-1.124,-0.368]	[-1.072,-0.795]	[-1.092,-0.872]	[-0.966,-0.564]	[-0.229,0.688]	[0.128,0.526]	[0.088,0.430]	[-0.061,0.537]	[0.446,1.249]	[0.846,1.133]	[0.962,1.188]	[0.609,1.010]
MOT_AGE	-0.013	-0.001	-0.001	-0.006	-0.005	0.002	-0.001	0.002	0.018	0.000	0.003	0.005
	[-0.039,0.013]	[-0.010,0.008]	[-0.009,0.006]	[-0.019,0.007]	[-0.035,0.026]	[-0.010,0.015]	[-0.010,0.009]	[-0.014,0.019]	[-0.008,0.044]	[-0.009,0.009]	[-0.004,0.009]	[-0.007,0.016]
HH_RUR	0.476**	0.641***	0.709***	0.450***	-0.086	-0.329***	-0.592***	-0.431***	-0.526**	-0.436***	-0.356***	-0.241**
	[0.034,0.919]	[0.448,0.835]	[0.572,0.846]	[0.227,0.672]	[-0.548,0.375]	[-0.570,-0.089]	[-0.758,-0.426]	[-0.686,-0.176]	[-0.960,-0.092]	[-0.623,-0.248]	[-0.479,-0.233]	[-0.443,-0.039]
HH_REL (ref: Catholic): Other Christian	-0.228	0.055	0.199**	0.176	0.514**	0.266**	-0.283***	-0.391***	-0.370	-0.278**	0.090	0.203
	[-0.756,0.299]	[-0.176,0.285]	[0.008,0.390]	[-0.132,0.485]	[0.018,1.010]	[0.019,0.512]	[-0.467,-0.099]	[-0.669,-0.113]	[-0.876,0.136]	[-0.493,-0.063]	[-0.073,0.254]	[-0.054,0.461]
HH_REL (ref: Catholic): Islam	0.347	-0.047	0.411***	0.416**	0.171	0.270*	-0.558***	-0.348*	-0.467*	-0.191	0.066	-0.052
	[-0.211,0.905]	[-0.333,0.239]	[0.182,0.640]	[0.030,0.802]	[-0.382,0.724]	[-0.049,0.589]	[-0.798,-0.319]	[-0.737,0.042]	[-1.002,0.068]	[-0.454,0.073]	[-0.134,0.267]	[-0.388,0.284]
HH_REL (ref: Catholic): Traditionalist	2.659**	1.275***	0.673**	0.535	0.000	-0.624*	-0.779**	-1.217	-1.795	-1.044***	-0.099	0.332
	[0.237,5.081]	[0.678,1.872]	[0.148,1.198]	[-0.915,1.985]	[0.000,0.000]	[-1.326,0.077]	[-1.488,-0.070]	[-3.410,0.976]	[-4.091,0.500]	[-1.706,-0.383]	[-0.608,0.410]	[-1.031,1.696]
HH_REL (ref: Catholic): Other	0.000	-1.175	0.003	-0.456	0.000	1.580**	0.000	0.000	0.000	0.330	0.489	1.074*
	[0.000,0.000]	[-2.618,0.269]	[-2.187,2.194]	[-1.750,0.838]	[0.000,0.000]	[0.067,3.094]	[0.000,0.000]	[0.000,0.000]	[0.000,0.000]	[-1.066,1.726]	[-1.708,2.685]	[-0.143,2.291]
HH_ETH (ref: Ekoi): Fulani	0.000	1.075***	0.369	0.357	0.000	-0.040	-0.898	-0.153	0.000	-1.106***	-0.403	-0.625
	[0.000,0.000]	[0.394,1.756]	[-0.914,1.651]	[-0.785,1.498]	[0.000,0.000]	[-1.341,1.262]	[-3.171,1.375]	[-1.943,1.638]	[0.000,0.000]	[-1.798,-0.414]	[-1.644,0.839]	[-1.722,0.471]
HH_ETH (ref: Ekoi): Hausa	0.924**	1.371***	0.590	0.759	-1.297	-1.110*	-0.953	-0.675	-0.617	-1.075***	-0.514	-0.770
	[0.152,1.696]	[0.719,2.023]	[-0.682,1.862]	[-0.351,1.869]	[-2.864,0.271]	[-2.374,0.154]	[-3.152,1.247]	[-2.384,1.035]	[-1.391,0.158]	[-1.736,-0.415]	[-1.745,0.717]	[-1.834,0.295]
HH_ETH (ref: Ekoi): Ibibio	-0.948	0.178	0.484	0.610	0.744	0.846	-0.114	-0.010	1.351	-0.198	-0.248	-0.492
	[-2.910,1.014]	[-0.575,0.931]	[-0.832,1.800]	[-0.601,1.821]	[-1.904,3.391]	[-0.451,2.142]	[-2.351,2.123]	[-1.848,1.828]	[-0.631,3.333]	[-0.943,0.546]	[-1.521,1.024]	[-1.656,0.673]
HH_ETH (ref: Ekoi): Igala	-1.208	-0.841*	-0.481	-0.705	1.997**	1.364**	0.878	1.585*	0.324	0.288	-0.001	-0.234
	[-2.662,0.247]	[-1.703,0.020]	[-1.845,0.882]	[-2.034,0.624]	[0.298,3.695]	[0.074,2.654]	[-1.350,3.106]	[-0.224,3.394]	[-1.053,1.702]	[-0.516,1.093]	[-1.297,1.296]	[-1.495,1.027]
HH_ETH (ref: Ekoi): Igbo	-0.910*	-1.046***	-0.853	-0.730	2.503***	2.415***	1.396	1.515*	-0.728	-0.660**	-0.459	-0.554
	[-1.907,0.088]	[-1.669,-0.424]	[-2.124,0.418]	[-1.824,0.364]	[1.109,3.896]	[1.271,3.559]	[-0.776,3.568]	[-0.123,3.153]	[-1.717,0.261]	[-1.289,-0.031]	[-1.685,0.767]	[-1.596,0.487]
HH_ETH (ref: Ekoi): Ijaw / Izon	0.012	0.996**	0.881	1.401**	0.257	0.102	-0.448	-0.270	0.377	-0.640	-0.825	-1.319**
	[-1.949,1.973]	[0.218,1.775]	[-0.435,2.196]	[0.175,2.628]	[-2.349,2.862]	[-1.229,1.434]	[-2.713,1.817]	[-2.136,1.597]	[-1.539,2.292]	[-1.437,0.157]	[-2.099,0.448]	[-2.502,-0.135]
HH_ETH (ref: Ekoi): Kanuri / Beriberi	0.656	1.330***	0.663	0.511	0.000	-0.880	-0.741	-0.448	0.131	-1.285***	-0.555	-0.644
	[-0.672,1.983]	[0.557,2.102]	[-0.674,1.999]	[-0.775,1.797]	[0.000,0.000]	[-2.734,0.975]	[-3.228,1.745]	[-2.686,1.790]	[-1.139,1.402]	[-2.060,-0.510]	[-1.848,0.738]	[-1.890,0.602]
HH_ETH (ref: Ekoi): Tiv	-1.588**	-0.853**	-0.951	-1.013*	2.493***	2.198***	1.302	1.892**	-0.041	-0.025	0.358	0.062
	[-3.085,-0.091]	[-1.559,-0.146]	[-2.279,0.378]	[-2.218,0.191]	[0.804,4.181]	[0.960,3.437]	[-0.924,3.527]	[0.164,3.621]	[-1.477,1.395]	[-0.756,0.706]	[-0.931,1.647]	[-1.090,1.215]
HH_ETH (ref: Ekoi): Yoruba	-0.659	-0.370	-0.137	-0.332	1.967***	1.501**	0.855	1.004	-0.462	-0.288	-0.408	-0.334
	[-1.615,0.297]	[-1.011,0.271]	[-1.409,1.134]	[-1.443,0.779]	[0.601,3.333]	[0.341,2.661]	[-1.318,3.029]	[-0.650,2.657]	[-1.400,0.476]	[-0.933,0.357]	[-1.636,0.820]	[-1.390,0.722]
HH_ETH (ref: Ekoi): Other	-0.001	0.307	0.015	-0.015	1.369**	0.946	0.373	0.781	-0.441	-0.414	-0.089	-0.337
	[-0.779,0.777]	[-0.299,0.913]	[-1.243,1.274]	[-1.092,1.062]	[0.075,2.663]	[-0.200,2.092]	[-1.795,2.541]	[-0.858,2.421]	[-1.240,0.359]	[-1.033,0.204]	[-1.308,1.129]	[-1.370,0.695]
HH_SIZ	0.030	0.022**	0.038***	0.044***	-0.043	-0.006	-0.021*	-0.035*	0.003	-0.014	-0.026***	-0.021*
	[-0.018,0.078]	[0.004,0.040]	[0.023,0.052]	[0.019,0.069]	[-0.104,0.018]	[-0.032,0.020]	[-0.044,0.002]	[-0.072,0.002]	[-0.045,0.051]	[-0.032,0.004]	[-0.039,-0.012]	[-0.045,0.002]
HH_WEA	-0.247***	-0.264***	-0.186***	-0.183***	0.177***	0.143***	0.106***	0.150***	0.082**	0.174***	0.118***	0.086***
	[-0.334,-0.160]	[-0.299,-0.230]	[-0.210,-0.161]	[-0.222,-0.145]	[0.090,0.264]	[0.101,0.186]	[0.075,0.137]	[0.103,0.196]	[0.003,0.160]	[0.142,0.206]	[0.096,0.140]	[0.051,0.121]
Constant	2.344***	2.386***	1.702**	1.973***	-3.864***	-4.914***	-3.104***	-3.415***	-2.637***	-2.205***	-2.397***	-2.225***
	[0.986,3.702]	[1.683,3.089]	[0.396,3.008]	[0.767,3.180]	[-5.696,-2.032]	[-6.157,-3.670]	[-5.323,-0.886]	[-5.192,-1.639]	[-3.998,-1.277]	[-2.916,-1.493]	[-3.658,-1.136]	[-3.372,-1.079]
Multilevel variance parameter: Level 1	0.733***	0.652***	0.490***	0.619***	0.439*	1.004***	0.814***	0.606***	0.846***	0.761***	0.473***	0.639***
	[0.226,1.240]	[0.466,0.839]	[0.368,0.612]	[0.402,0.835]	[-0.014,0.892]	[0.672,1.335]	[0.584,1.045]	[0.351,0.861]	[0.332,1.360]	[0.570,0.952]	[0.364,0.581]	[0.442,0.836]
Level 1 Observations (child)	1395	11609	16475	5233	1346	11609	16467	5211	1395	11609	16475	5233
Level 2 Observations (LGA)	153	370	370	510	153	370	370	510	153	370	370	510
Akaike Information Criterion	1147.831	7880.9	12731.565	4519.37	901.306	5217.905	8321.506	3169.365	1222.345	8804.345	14843.344	5370.273
Prob. > X^2	<0.001	<0.001	<0.001	<0.001	<0.001	<0.001	<0.001	<0.001	<0.001	<0.001	<0.001	<0.001

95% confidence intervals in brackets
* p < 0.10, ** p < 0.05, *** p < 0.01

BMJ Publishing Group Limited (BMJ) disclaims all liability and responsibility arising from any reliance placed on this supplemental material which has been supplied by the author(s)

Table 6b-3: Additional robustness checks: year-wise stratification to detect time trends: antenatal care and child survival

Dependent variables: various indicators of maternal care and child survival (see right)	**Antenatal care**								**Child survival**			
	**No. of antenatal care visits**				**No. of tetanus injections**				**Total exposure**			
	2003	2008	2013	2018	2003	2008	2013	2018	2003	2008	2013	2018
EXP_PREG	0.015 [-0.239,0.270]	-0.051 [-0.113,0.010]	-0.024 [-0.074,0.025]	-0.054*** [-0.086,-0.022]	0.014 [-0.056,0.084]	-0.012 [-0.030,0.006]	-0.005 [-0.018,0.008]	0.006 [-0.008,0.019]				
EXP_TOT_nod (total exposure, date approximation)									-0.087 [-0.267,0.092]	-0.038*** [-0.061,-0.015]	-0.006 [-0.021,0.009]	-0.037*** [-0.052,-0.021]
CHI_AGE									-0.122* [-0.249,0.004]	0.007 [-0.011,0.026]	0.001 [-0.016,0.017]	-0.003 [-0.019,0.014]
CHI_AGE2									0.005* [-0.001,0.011]	0.000 [-0.000,0.000]	0.000 [-0.000,0.000]	0.000* [-0.000,0.000]
CHI_ORD									-0.159*** [-0.276,-0.043]	-0.142*** [-0.183,-0.102]	-0.138*** [-0.178,-0.099]	-0.171*** [-0.212,-0.131]
CHI_SEX									0.182 [-0.173,0.537]	0.283*** [0.149,0.418]	0.224*** [0.098,0.350]	0.151** [0.027,0.275]
MOT_ANC									0.043* [-0.007,0.093]	-0.009 [-0.027,0.010]	0.006 [-0.010,0.022]	-0.005 [-0.025,0.015]
MOT_EDM	0.122** [0.000,0.244]	0.123*** [0.084,0.162]	0.088*** [0.055,0.121]	0.074*** [0.024,0.123]	0.047*** [0.016,0.078]	0.033*** [0.023,0.043]	0.028*** [0.017,0.038]	0.035*** [0.018,0.051]	0.066 [-0.026,0.158]	0.007 [-0.028,0.043]	0.023 [-0.010,0.057]	0.003 [-0.030,0.036]
MOT_EDF	0.094 [-0.028,0.216]	0.075*** [0.040,0.109]	0.093*** [0.064,0.121]	0.025 [-0.021,0.072]	0.010 [-0.018,0.038]	0.020*** [0.010,0.031]	0.026*** [0.016,0.036]	0.023*** [0.009,0.038]	0.092** [0.009,0.176]	0.035** [0.003,0.068]	0.003 [-0.028,0.034]	0.035** [0.006,0.064]
MOT_AWE	2.141*** [1.525,2.757]	1.721*** [1.483,1.959]	1.955*** [1.752,2.158]	1.553*** [1.328,1.779]	0.750*** [0.563,0.937]	0.582*** [0.511,0.654]	0.640*** [0.579,0.700]	0.576*** [0.505,0.647]				
MOT_AGE	0.035** [0.003,0.067]	0.005 [-0.005,0.015]	0.016*** [0.008,0.025]	0.006 [-0.009,0.022]	0.007 [-0.003,0.017]	0.002 [-0.001,0.005]	0.001 [-0.002,0.004]	-0.005** [-0.009,-0.000]	0.041* [-0.006,0.088]	0.013* [-0.002,0.028]	0.003 [-0.012,0.017]	0.007 [-0.008,0.022]
HH_RUR	-0.486 [-1.276,0.305]	-0.456* [-0.941,0.030]	-0.726*** [-1.070,-0.381]	-0.717*** [-1.048,-0.387]	-0.115 [-0.253,0.023]	-0.199*** [-0.300,-0.098]	-0.171*** [-0.248,-0.095]	-0.117*** [-0.205,-0.029]	-0.067 [-0.560,0.427]	-0.236** [-0.439,-0.033]	-0.365*** [-0.550,-0.181]	-0.049 [-0.219,0.121]
HH_REL (ref: Catholic): Other Christian	0.320 [-0.579,1.220]	-0.156 [-0.492,0.179]	-0.057 [-0.424,0.310]	0.309 [-0.086,0.704]	0.197 [-0.048,0.443]	-0.051 [-0.143,0.040]	-0.049 [-0.133,0.034]	-0.085 [-0.190,0.021]	0.083 [-0.578,0.745]	0.041 [-0.263,0.345]	-0.247 [-0.546,0.052]	-0.412** [-0.739,-0.086]
HH_REL (ref: Catholic): Islam	0.058 [-1.035,1.151]	-0.153 [-0.614,0.308]	-0.203 [-0.690,0.284]	-0.025 [-0.545,0.495]	0.004 [-0.227,0.236]	-0.135** [-0.259,-0.010]	-0.125** [-0.231,-0.019]	-0.083 [-0.228,0.061]	0.682** [0.021,1.344]	0.195 [-0.167,0.557]	-0.303* [-0.657,0.052]	-0.369* [-0.748,0.009]
HH_REL (ref: Catholic): Traditionalist	-1.539*** [-2.633,-0.445]	-0.418 [-1.022,0.186]	-0.582 [-1.401,0.238]	-1.368** [-2.474,-0.262]	0.075 [-0.669,0.820]	-0.440*** [-0.623,-0.257]	-0.357*** [-0.585,-0.128]	-0.213 [-0.621,0.194]	-0.160 [-1.352,1.031]	-0.059 [-0.591,0.472]	-0.187 [-0.876,0.501]	1.121 [-0.914,3.155]
HH_REL (ref: Catholic): Other	-4.444*** [-5.625,-3.264]	1.812 [-0.963,4.587]	-1.774*** [-2.603,-0.946]	0.433 [-1.236,2.102]	-0.814*** [-1.099,-0.529]	0.288 [-0.732,1.307]	-0.541 [-1.258,0.176]	-0.017 [-0.660,0.627]	0.000 [0.000,0.000]	-0.835 [-2.150,0.481]	0.000 [0.000,0.000]	0.000 [0.000,0.000]
HH_ETH (ref: Ekoi): Fulani	0.000 [0.000,0.000]	-0.658 [-2.000,0.683]	0.567 [-2.267,3.401]	-0.490 [-2.077,1.096]	0.000 [0.000,0.000]	-0.541*** [-0.881,-0.201]	-0.336*** [-0.521,-0.152]	-0.281** [-0.542,-0.021]	0.000 [0.000,0.000]	-0.356 [-1.217,0.505]	0.302* [-0.012,0.617]	0.472 [-0.516,1.461]
HH_ETH (ref: Ekoi): Hausa	0.074 [-0.470,0.619]	-0.617 [-1.948,0.714]	1.027 [-1.798,3.852]	0.037 [-1.542,1.616]	0.062 [-0.137,0.260]	-0.539*** [-0.875,-0.202]	-0.179** [-0.355,-0.003]	-0.164 [-0.419,0.090]	-0.577 [-1.324,0.169]	-0.598 [-1.448,0.252]	-0.163 [-0.389,0.064]	0.263 [-0.710,1.235]
HH_ETH (ref: Ekoi): Ibibio	1.244 [-1.726,4.213]	1.024 [-0.267,2.314]	0.716 [-2.289,3.721]	0.064 [-1.630,1.758]	0.364 [-0.397,1.126]	-0.255 [-0.619,0.110]	-0.199 [-0.444,0.047]	0.119 [-0.247,0.485]	-2.494*** [-4.324,-0.664]	-0.137 [-1.235,0.961]	-0.204 [-0.758,0.351]	0.185 [-0.906,1.275]
HH_ETH (ref: Ekoi): Igala	3.934*** [2.295,5.573]	0.215 [-1.301,1.731]	0.941 [-1.957,3.838]	0.854 [-0.935,2.642]	2.076*** [1.699,2.453]	0.024 [-0.375,0.424]	0.189 [-0.112,0.491]	0.193 [-0.241,0.628]	-1.161 [-2.970,0.648]	-0.713 [-1.709,0.282]	0.528 [-0.403,1.459]	0.114 [-1.020,1.247]
HH_ETH (ref: Ekoi): Igbo	2.564*** [1.360,3.768]	1.493** [0.169,2.818]	2.530* [-0.338,5.398]	2.459*** [0.844,4.073]	0.657*** [0.299,1.015]	-0.077 [-0.404,0.250]	0.103 [-0.043,0.249]	0.301*** [0.072,0.530]	-0.783 [-1.910,0.345]	-0.654 [-1.467,0.159]	-0.409*** [-0.695,-0.122]	0.289 [-0.675,1.252]
HH_ETH (ref: Ekoi): Ijaw / Izon	1.149 [-1.180,3.478]	-0.127 [-1.742,1.488]	0.263 [-2.656,3.183]	-0.052 [-2.106,2.002]	0.201 [-0.240,0.641]	-0.184 [-0.601,0.233]	-0.198* [-0.422,0.025]	-0.183 [-0.541,0.174]	-1.101 [-3.020,0.818]	-0.575 [-1.472,0.323]	-0.179 [-0.592,0.234]	1.357** [0.109,2.605]
HH_ETH (ref: Ekoi): Kanuri / Beriberi	1.003** [0.009,1.996]	-0.914 [-2.320,0.491]	1.163 [-1.701,4.028]	-0.290 [-1.924,1.345]	0.223 [-0.507,0.953]	-0.534*** [-0.911,-0.157]	-0.157 [-0.405,0.091]	-0.234 [-0.611,0.143]	-0.102 [-1.519,1.315]	-0.529 [-1.434,0.377]	0.231 [-0.391,0.854]	0.571 [-0.493,1.636]
HH_ETH (ref: Ekoi): Tiv	-0.068 [-1.797,1.661]	-0.978 [-2.399,0.443]	0.077 [-2.858,3.012]	0.280 [-1.475,2.036]	0.326 [-0.395,1.047]	-0.565*** [-0.929,-0.202]	-0.375** [-0.669,-0.082]	-0.197 [-0.461,0.068]	-0.327 [-1.976,1.322]	-0.847* [-1.710,0.016]	-0.142 [-0.698,0.415]	0.856 [-0.246,1.959]
HH_ETH (ref: Ekoi): Yoruba	5.942*** [4.397,7.488]	2.849*** [1.460,4.238]	3.553** [0.695,6.412]	3.056*** [1.428,4.685]	0.767*** [0.436,1.098]	-0.071 [-0.401,0.258]	0.049 [-0.110,0.208]	-0.027 [-0.263,0.208]	-1.234* [-2.477,0.009]	-0.103 [-0.951,0.745]	0.168 [-0.141,0.476]	0.808 [-0.178,1.794]
HH_ETH (ref: Ekoi): Other	1.491*** [0.544,2.437]	0.149 [-1.149,1.447]	1.326 [-1.484,4.137]	0.679 [-0.884,2.242]	0.556*** [0.259,0.852]	-0.211 [-0.536,0.114]	-0.040 [-0.186,0.106]	0.059 [-0.168,0.285]	-0.829* [-1.679,0.021]	-0.570 [-1.378,0.238]	0.000 [0.000,0.000]	0.466 [-0.489,1.421]
HH_SIZ	-0.070** [-0.140,-0.001]	-0.031*** [-0.052,-0.010]	-0.066*** [-0.086,-0.047]	-0.036** [-0.064,-0.008]	-0.009 [-0.024,0.006]	-0.003 [-0.009,0.002]	-0.014*** [-0.020,-0.007]	-0.001 [-0.011,0.009]	0.059* [-0.001,0.118]	0.134*** [0.107,0.161]	0.153*** [0.127,0.180]	0.133*** [0.108,0.157]
HH_WEA	0.348*** [0.212,0.484]	0.356*** [0.306,0.405]	0.297*** [0.250,0.345]	0.221*** [0.171,0.272]	0.076*** [0.045,0.108]	0.073*** [0.058,0.087]	0.063*** [0.050,0.075]	0.037*** [0.021,0.053]	0.109** [0.000,0.217]	-0.018 [-0.060,0.024]	0.018 [-0.016,0.053]	0.015 [-0.017,0.048]
Constant	0.814 [-0.905,2.534]	2.695*** [1.257,4.132]	2.145 [-0.805,5.095]	2.444*** [0.707,4.182]	0.054 [-0.379,0.487]	1.125*** [0.765,1.485]	1.010*** [0.806,1.215]	1.074*** [0.766,1.383]	2.191*** [0.564,3.818]	2.852*** [1.933,3.771]	2.839*** [2.295,3.383]	2.454*** [1.373,3.536]
Multilevel variance parameter: Level 1	0.446*** [0.139,0.754]	0.674*** [0.535,0.812]	0.881*** [0.773,0.988]	0.382*** [0.215,0.548]	-1.847*** [-2.590,-1.104]	-1.260*** [-1.389,-1.131]	-1.339*** [-1.463,-1.216]	-1.244*** [-1.420,-1.068]	0.269 [-0.119,0.658]	0.035 [-0.022,0.092]	0.079** [0.013,0.145]	0.066** [0.005,0.127]
Multilevel variance parameter: Residual	1.395*** [1.266,1.523]	1.270*** [1.213,1.327]	1.350*** [1.298,1.402]	1.173*** [1.122,1.225]	0.126*** [0.031,0.222]	0.041* [-0.005,0.086]	0.114*** [0.078,0.150]	0.036* [-0.006,0.079]				
Level 1 Observations (child)	1400	11781	16510	5328	1437	12654	17086	5408	1921	14275	18276	17857
Level 2 Observations (LGA)	153	370	370	511	154	370	370	512	158	370	370	515
Akaike Information Criterion	8042.517	64196.451	92468.14	28199.742	4511.606	37408.855	52854.597	16072.681	1014.205	6786.461	7863.049	8003.619
Prob. > X^2	<0.001	<0.001	<0.001	<0.001	<0.001	<0.001	<0.001	<0.001	<0.001	<0.001	<0.001	<0.001

95% confidence intervals in brackets

* p < 0.10, ** p < 0.05, *** p < 0.01

Table 6c: Additional robustness checks: exclusion of measles vaccine from full immunisation outcome

Dependent variable: non-polio/-measles full immunisation status	Full model	Interaction model (EXPxAGE)	**Full model (by region)** North Central	North East	North West	South East	South South	South West	**Interaction model (EXPxAGE) (by region)** North Central	North East	North West	South East	South South	South West
EXP_CHI	-0.020***	-0.032***	0.028*	-0.061***	-0.045	0.051	-0.019	-0.064	0.025	-0.065***	-0.041	0.057	-0.063	-0.085
	[-0.033,-0.008]	[-0.054,-0.010]	[-0.003,0.059]	[-0.094,-0.028]	[-0.118,0.029]	[-0.100,0.202]	[-0.131,0.093]	[-0.154,0.026]	[-0.022,0.072]	[-0.110,-0.019]	[-0.116,0.034]	[-0.157,0.271]	[-0.245,0.118]	[-0.213,0.043]
EXPxAGE		0.000							0.000	0.000	0.000	-0.001	0.001	0.000
		[-0.000,0.001]							[-0.001,0.001]	[-0.001,0.001]	[-0.001,0.001]	[-0.004,0.003]	[-0.002,0.003]	[-0.002,0.002]
CHI_AGE	0.026***	0.016***	0.009	0.049**	0.051	0.022	0.008	0.041**	-0.003	0.034***	0.033	0.007	0.011	0.032***
	[0.010,0.042]	[0.009,0.023]	[-0.023,0.041]	[0.008,0.091]	[-0.015,0.117]	[-0.029,0.072]	[-0.031,0.048]	[0.005,0.078]	[-0.019,0.014]	[0.011,0.056]	[-0.015,0.081]	[-0.028,0.042]	[-0.015,0.038]	[0.009,0.055]
CHI_AGE2	0.000		0.000	0.000	0.000	0.000	0.000	0.000						
	[-0.000,0.000]		[-0.001,0.000]	[-0.001,0.000]	[-0.001,0.000]	[-0.001,0.001]	[-0.001,0.001]	[-0.001,0.000]						
CHI_ORD	-0.046***	-0.046***	-0.018	-0.081***	-0.038	0.003	-0.071**	-0.056	-0.018	-0.082***	-0.038	0.004	-0.071**	-0.056
	[-0.071,-0.020]	[-0.071,-0.020]	[-0.072,0.036]	[-0.142,-0.020]	[-0.102,0.026]	[-0.072,0.078]	[-0.136,-0.005]	[-0.131,0.020]	[-0.072,0.037]	[-0.143,-0.021]	[-0.101,0.026]	[-0.071,0.079]	[-0.137,-0.005]	[-0.131,0.019]
CHI_SEX	-0.006	-0.006	-0.050	-0.038	0.048	-0.026	0.158	-0.073	-0.048	-0.039	0.045	-0.026	0.158	-0.072
	[-0.084,0.072]	[-0.084,0.072]	[-0.208,0.108]	[-0.222,0.146]	[-0.149,0.244]	[-0.279,0.228]	[-0.045,0.362]	[-0.266,0.121]	[-0.206,0.110]	[-0.223,0.144]	[-0.151,0.242]	[-0.280,0.228]	[-0.045,0.362]	[-0.266,0.122]
MOT_ANC	0.061***	0.061***	0.100***	0.135***	0.070***	0.027*	0.044***	0.041***	0.100***	0.135***	0.071***	0.027*	0.044***	0.041***
	[0.052,0.071]	[0.052,0.071]	[0.077,0.123]	[0.101,0.169]	[0.035,0.106]	[-0.000,0.055]	[0.022,0.066]	[0.025,0.057]	[0.077,0.123]	[0.101,0.169]	[0.035,0.106]	[-0.001,0.054]	[0.023,0.066]	[0.025,0.058]
MOT_EDM	0.056***	0.056***	0.054***	0.049**	0.073***	0.073**	0.046*	0.044*	0.054***	0.049**	0.073***	0.074**	0.045*	0.044*
	[0.037,0.074]	[0.037,0.074]	[0.019,0.090]	[0.008,0.091]	[0.027,0.119]	[0.005,0.142]	[-0.007,0.098]	[-0.006,0.094]	[0.019,0.089]	[0.007,0.091]	[0.027,0.118]	[0.006,0.143]	[-0.008,0.098]	[-0.006,0.094]
MOT_EDF	0.024**	0.024**	0.035*	0.014	0.044**	0.014	0.024	-0.033	0.035*	0.014	0.044**	0.013	0.024	-0.034
	[0.006,0.043]	[0.006,0.043]	[-0.002,0.072]	[-0.026,0.055]	[0.002,0.085]	[-0.057,0.085]	[-0.034,0.082]	[-0.086,0.020]	[-0.002,0.072]	[-0.026,0.055]	[0.003,0.085]	[-0.058,0.084]	[-0.034,0.082]	[-0.086,0.019]
MOT_AWE	3.124***	3.121***	2.731***	2.920***	3.684***	3.059***	2.965***	3.248***	2.730***	2.920***	3.683***	3.053***	2.964***	3.240***
	[3.008,3.240]	[3.005,3.237]	[2.505,2.956]	[2.644,3.196]	[3.415,3.953]	[2.682,3.436]	[2.649,3.281]	[2.892,3.604]	[2.505,2.955]	[2.644,3.196]	[3.414,3.952]	[2.677,3.430]	[2.648,3.280]	[2.885,3.595]
MOT_AGE	0.021***	0.021***	0.011	0.031***	0.033***	0.016	0.023**	0.015	0.011	0.031***	0.033***	0.015	0.023**	0.015
	[0.012,0.030]	[0.012,0.030]	[-0.007,0.029]	[0.008,0.054]	[0.008,0.058]	[-0.012,0.043]	[0.001,0.045]	[-0.007,0.036]	[-0.007,0.029]	[0.008,0.054]	[0.008,0.057]	[-0.012,0.043]	[0.001,0.046]	[-0.007,0.037]
HH_RUR	-0.150***	-0.150***	-0.045	-0.214	-0.172	-0.073	-0.037	-0.377***	-0.044	-0.214	-0.173	-0.072	-0.036	-0.376***
	[-0.260,-0.040]	[-0.259,-0.040]	[-0.279,0.188]	[-0.490,0.063]	[-0.484,0.140]	[-0.374,0.228]	[-0.299,0.226]	[-0.645,-0.109]	[-0.278,0.190]	[-0.491,0.062]	[-0.485,0.138]	[-0.374,0.229]	[-0.298,0.226]	[-0.644,-0.108]
HH_REL (ref: Catholic): Other Christian	-0.187**	-0.188**	-0.111	-0.611*	0.389	-0.263*	-0.345*	-0.132	-0.112	-0.616*	0.387	-0.261*	-0.350*	-0.132
	[-0.343,-0.031]	[-0.344,-0.032]	[-0.387,0.164]	[-1.272,0.051]	[-0.330,1.108]	[-0.542,0.016]	[-0.726,0.036]	[-0.710,0.447]	[-0.387,0.163]	[-1.276,0.044]	[-0.332,1.106]	[-0.540,0.018]	[-0.730,0.031]	[-0.710,0.447]
HH_REL (ref: Catholic): Islam	-0.575***	-0.571***	-0.448***	-1.070***	-0.348	-0.342	-0.101	-0.464	-0.446***	-1.080***	-0.348	-0.336	-0.103	-0.463
	[-0.761,-0.389]	[-0.757,-0.385]	[-0.759,-0.137]	[-1.744,-0.396]	[-1.166,0.470]	[-3.017,2.333]	[-0.916,0.714]	[-1.059,0.131]	[-0.758,-0.135]	[-1.752,-0.407]	[-1.166,0.469]	[-3.009,2.336]	[-0.917,0.712]	[-1.057,0.132]
HH_REL (ref: Catholic): Traditionalist	-0.757***	-0.756***	-0.964*	-1.116*	-0.812	-0.562	-0.866*	-0.025	-0.953*	-1.136*	-0.789	-0.557	-0.872*	-0.044
	[-1.166,-0.347]	[-1.165,-0.346]	[-2.082,0.155]	[-2.434,0.202]	[-2.396,0.773]	[-1.235,0.110]	[-1.896,0.163]	[-1.378,1.327]	[-2.070,0.163]	[-2.452,0.180]	[-2.370,0.792]	[-1.230,0.115]	[-1.903,0.158]	[-1.393,1.305]
HH_REL (ref: Catholic): Other	-0.098	-0.106	-0.138	0.000	0.000	0.000	-0.301	0.447	-0.137	0.000	0.000	0.000	-0.306	0.436
	[-1.149,0.952]	[-1.156,0.943]	[-2.341,2.065]	[0.000,0.000]	[0.000,0.000]	[0.000,0.000]	[-1.596,0.994]	[-3.479,4.372]	[-2.338,2.065]	[0.000,0.000]	[0.000,0.000]	[0.000,0.000]	[-1.600,0.989]	[-3.479,4.350]
HH_ETH (ref: Ekoi): Fulani	-0.847***	-0.833***	-0.261	-0.138	0.316	0.000		-0.881	-0.252	-0.142	0.319	0.000		-0.863
	[-1.388,-0.305]	[-1.375,-0.292]	[-0.817,0.295]	[-0.430,0.154]	[-0.343,0.975]	[0.000,0.000]		[-1.937,0.175]	[-0.807,0.303]	[-0.434,0.150]	[-0.339,0.978]	[0.000,0.000]		[-1.918,0.193]
HH_ETH (ref: Ekoi): Hausa	-0.804***	-0.784***	-0.461**	-0.137	0.410	-0.677	-0.163	-0.110	-0.459**	-0.137	0.412	-0.660	-0.164	-0.113
	[-1.328,-0.281]	[-1.307,-0.260]	[-0.849,-0.073]	[-0.433,0.158]	[-0.106,0.925]	[-3.000,1.646]	[-2.964,2.638]	[-1.047,0.828]	[-0.848,-0.071]	[-0.432,0.159]	[-0.103,0.928]	[-2.980,1.660]	[-2.970,2.643]	[-1.050,0.825]
HH_ETH (ref: Ekoi): Ibibio	-0.582**	-0.582**	-1.077	0.000	0.000	-0.764	-0.963***	0.727	-1.082	0.000	0.000	-0.748	-0.960***	0.734
	[-1.158,-0.006]	[-1.158,-0.006]	[-3.132,0.979]	[0.000,0.000]	[0.000,0.000]	[-3.466,1.937]	[-1.527,-0.399]	[-0.674,2.128]	[-3.140,0.977]	[0.000,0.000]	[0.000,0.000]	[-3.447,1.951]	[-1.522,-0.397]	[-0.667,2.134]
HH_ETH (ref: Ekoi): Igala	-0.733**	-0.731**	-0.423**	0.000	0.000	-1.131	-1.242	1.297	-0.428**	0.000	0.000	-1.134	-1.257	1.306
	[-1.359,-0.106]	[-1.358,-0.105]	[-0.832,-0.014]	[0.000,0.000]	[0.000,0.000]	[-3.896,1.635]	[-3.919,1.435]	[-0.300,2.894]	[-0.837,-0.019]	[0.000,0.000]	[0.000,0.000]	[-3.897,1.630]	[-3.956,1.441]	[-0.293,2.905]
HH_ETH (ref: Ekoi): Igbo	-0.031	-0.031	-0.312	0.195	0.011	-0.421	-0.199	0.848***	-0.305	0.206	0.013	-0.411	-0.198	0.850***
	[-0.543,0.480]	[-0.542,0.481]	[-0.873,0.248]	[-1.479,1.868]	[-1.068,1.090]	[-1.915,1.074]	[-0.831,0.433]	[0.231,1.465]	[-0.865,0.255]	[-1.466,1.877]	[-1.065,1.091]	[-1.904,1.081]	[-0.829,0.433]	[0.233,1.466]
HH_ETH (ref: Ekoi): Ijaw / Izon	-0.418	-0.419	0.000			0.000	-0.772***	-1.020	0.000			0.000	-0.767***	-1.030
	[-1.001,0.166]	[-1.002,0.165]	[0.000,0.000]			[0.000,0.000]	[-1.306,-0.238]	[-2.616,0.577]	[0.000,0.000]			[0.000,0.000]	[-1.300,-0.234]	[-2.626,0.567]
HH_ETH (ref: Ekoi): Kanuri / Beriberi	-0.940***	-0.915***	-0.903	-0.278	0.270		0.000	2.526*	-0.891	-0.272	0.279		0.000	2.558*
	[-1.569,-0.312]	[-1.544,-0.286]	[-2.583,0.778]	[-0.722,0.166]	[-0.982,1.522]		[0.000,0.000]	[-0.454,5.506]	[-2.572,0.791]	[-0.716,0.172]	[-0.973,1.530]		[0.000,0.000]	[-0.428,5.545]
HH_ETH (ref: Ekoi): Tiv	-0.529*	-0.525*	-0.187	-0.336	0.000	0.000	0.000	1.480	-0.189	-0.360	0.000	0.000	0.000	1.493
	[-1.122,0.064]	[-1.118,0.068]	[-0.574,0.200]	[-1.274,0.603]	[0.000,0.000]	[0.000,0.000]	[0.000,0.000]	[-0.382,3.342]	[-0.576,0.198]	[-1.298,0.579]	[0.000,0.000]	[0.000,0.000]	[0.000,0.000]	[-0.363,3.349]
HH_ETH (ref: Ekoi): Yoruba	-0.398	-0.395	0.162	-0.053	0.949	0.000	-2.229***	0.313*	0.163	-0.042	0.945	0.000	-2.222***	0.313*
	[-0.916,0.120]	[-0.913,0.123]	[-0.192,0.516]	[-1.510,1.403]	[-0.456,2.355]	[0.000,0.000]	[-3.204,-1.254]	[-0.007,0.633]	[-0.191,0.517]	[-1.498,1.414]	[-0.452,2.341]	[0.000,0.000]	[-3.196,-1.248]	[-0.007,0.633]
HH_ETH (ref: Ekoi): Other	-0.499*	-0.490*	0.000	0.000	0.000	0.000	-0.852***	0.000	0.000	0.000	0.000	0.000	-0.850***	0.000
	[-0.999,0.001]	[-0.989,0.010]	[0.000,0.000]	[0.000,0.000]	[0.000,0.000]	[0.000,0.000]	[-1.356,-0.347]	[0.000,0.000]	[0.000,0.000]	[0.000,0.000]	[0.000,0.000]	[0.000,0.000]	[-1.353,-0.346]	[0.000,0.000]
HH_SIZ	-0.014**	-0.014**	-0.012	-0.021*	-0.010	-0.037	-0.013	0.004	-0.013	-0.021*	-0.009	-0.037	-0.013	0.004
	[-0.027,-0.001]	[-0.027,-0.001]	[-0.038,0.014]	[-0.046,0.003]	[-0.038,0.019]	[-0.083,0.009]	[-0.059,0.033]	[-0.047,0.055]	[-0.038,0.013]	[-0.046,0.003]	[-0.037,0.019]	[-0.083,0.008]	[-0.059,0.033]	[-0.047,0.055]
HH_WEA	0.132***	0.132***	0.140***	0.112***	0.120***	0.152***	0.107***	0.121***	0.139***	0.112***	0.120***	0.152***	0.107***	0.122***
	[0.111,0.153]	[0.111,0.153]	[0.095,0.184]	[0.059,0.165]	[0.067,0.173]	[0.090,0.214]	[0.057,0.157]	[0.064,0.179]	[0.095,0.184]	[0.059,0.165]	[0.067,0.173]	[0.090,0.214]	[0.057,0.157]	[0.065,0.179]
YEAR (ref: 2003): 2008	0.784***	0.812***	1.026***	0.798**	1.350***	1.354***	0.961***	-0.273	1.044***	0.826**	1.352***	1.355***	0.952***	-0.237
	[0.524,1.044]	[0.551,1.073]	[0.528,1.525]	[0.030,1.567]	[0.502,2.198]	[0.679,2.029]	[0.237,1.685]	[-0.981,0.436]	[0.543,1.545]	[0.054,1.598]	[0.501,2.203]	[0.680,2.029]	[0.228,1.676]	[-0.948,0.473]
YEAR (ref: 2003): 2013	1.032***	1.064***	0.870***	1.389***	1.696***	1.767***	1.833***	-0.659*	0.894***	1.419***	1.673***	1.787***	1.859***	-0.617*
	[0.773,1.290]	[0.804,1.324]	[0.375,1.365]	[0.625,2.153]	[0.745,2.647]	[1.092,2.442]	[1.113,2.552]	[-1.375,0.056]	[0.397,1.391]	[0.652,2.186]	[0.725,2.621]	[1.111,2.463]	[1.137,2.582]	[-1.333,0.099]
YEAR (ref: 2003): 2018	1.237***	1.252***	1.389***	1.753***	1.447***	2.088***	1.779***	-0.572	1.410***	1.789***	1.490***	2.084***	1.711***	-0.585
	[0.970,1.505]	[0.985,1.518]	[0.874,1.904]	[0.978,2.528]	[0.609,2.285]	[1.281,2.896]	[0.959,2.599]	[-1.341,0.197]	[0.896,1.923]	[1.014,2.563]	[0.659,2.322]	[1.257,2.911]	[0.864,2.559]	[-1.356,0.186]
Constant	-4.445***	-4.303***	-4.563***	-5.058***	-7.592***	-4.376***	-3.903***	-3.203***	-4.425***	-4.860***	-7.380***	-4.239***	-3.851***	-3.074***
	[-5.084,-3.806]	[-4.926,-3.680]	[-5.376,-3.750]	[-6.190,-3.926]	[-8.962,-6.223]	[-6.286,-2.466]	[-5.111,-2.694]	[-4.375,-2.032]	[-5.190,-3.660]	[-5.941,-3.779]	[-8.692,-6.067]	[-6.113,-2.365]	[-5.017,-2.685]	[-4.216,-1.932]
Multilevel variance parameter: Level 1	0.354***	0.353***	0.213***	0.250***	0.695***	0.305***	0.045	0.296***	0.214***	0.252***	0.692***	0.304***	0.043	0.293***
	[0.274,0.434]	[0.273,0.433]	[0.085,0.342]	[0.097,0.402]	[0.394,0.996]	[0.085,0.525]	[-0.049,0.138]	[0.118,0.475]	[0.085,0.342]	[0.098,0.405]	[0.392,0.992]	[0.084,0.524]	[-0.050,0.136]	[0.116,0.471]
Level 1 Observations (child)	24367	24367	4346	5015	7248	2034	2511	3195	4346	5015	7248	2034	2511	3195
Level 2 Observations (LGA)	684	684	113	100	166	85	111	121	113	100	166	85	111	121
Akaike Information Criterion	17045.336	17044.81	4014.257	3048.336	2966.083	1700.836	2421.769	2826.662	4014.885	3048.749	2966.498	1701.332	2421.402	2826.912
Prob. > X^2	<0.001	<0.001	<0.001	<0.001	<0.001	<0.001	<0.001	<0.001	<0.001	<0.001	<0.001	<0.001	<0.001	<0.001

95% confidence intervals in brackets

* p < 0.10, ** p < 0.05, *** p < 0.01

Table 6d: Additional robustness checks: decomposition of SIA exposure into different campaign types

Dependent variables: various indicators of routine immunisation, maternal care and child survival (see right)	**Non-polio full immunisation (full model)**	**Delivery**			**Antenatal care**		**Child survival (total exposure)**	**Non-polio full immunisation status (by region)** **Full model**					
		At home	At private facility	At public facility	No. of antenatal care visits	No. of tetanus injections		North Central	North East	North West	South East	South South	South West
EXP_CHI (NIDs and sNIDs)	-0.037** [-0.070,-0.004]							0.008 [-0.063,0.080]	-0.151*** [-0.233,-0.069]	-0.056 [-0.191,0.079]	0.038 [-0.125,0.202]	0.118* [-0.021,0.256]	-0.103* [-0.216,0.011]
EXP_CHI (IPDs and sIPDs)	-0.025*** [-0.037,-0.012]							0.043** [0.006,0.080]	-0.093*** [-0.128,-0.058]	-0.078* [-0.167,0.010]	0.119 [-0.029,0.268]	0.189*** [0.061,0.317]	-0.072 [-0.165,0.021]
EXP_CHI (mop-up campaigns)	-0.007 [-0.048,0.035]							-0.011 [-0.103,0.082]	0.027 [-0.048,0.101]	-0.139** [-0.247,-0.030]	0.000 [0.000,0.000]	0.000 [0.000,0.000]	0.303 [-0.418,1.024]
EXP_CHI (MNCHW)	-0.047 [-0.146,0.052]							-0.175 [-0.393,0.044]	-0.102 [-0.359,0.155]	0.117 [-0.197,0.431]	-0.034 [-0.340,0.272]	-0.348*** [-0.610,-0.085]	-0.030 [-0.267,0.206]
EXP_PREG (NIDs and sNIDs)		0.014 [-0.018,0.046]	-0.063*** [-0.104,-0.021]	0.003 [-0.027,0.033]	-0.043* [-0.086,0.000]	0.002 [-0.010,0.013]							
EXP_PREG (IPDs and sIPDs)		0.004 [-0.021,0.029]	-0.093*** [-0.127,-0.058]	0.020* [-0.003,0.043]	-0.059*** [-0.095,-0.023]	-0.007 [-0.017,0.003]							
EXP_PREG (mop-up campaigns)		-0.012 [-0.049,0.025]	-0.021 [-0.093,0.051]	0.017 [-0.019,0.053]	0.022 [-0.015,0.058]	0.013* [-0.002,0.029]							
EXP_PREG (MNCHW)		0.088** [0.009,0.167]	0.024 [-0.066,0.114]	-0.075** [-0.146,-0.004]	0.111* [-0.009,0.230]	0.027* [-0.002,0.057]							
EXP_TOT_nod (NIDs and sNIDs)							-0.035*** [-0.057,-0.014]						
EXP_TOT_nod (IPDs and sIPDs)							-0.014*** [-0.024,-0.004]						
EXP_TOT_nod (mop-up campaigns)							-0.018 [-0.041,0.005]						
EXP_TOT_nod (MNCHW)							0.040 [-0.052,0.133]						
CHI_AGE	0.079*** [0.062,0.095]						-0.004 [-0.015,0.007]	0.072*** [0.038,0.107]	0.082*** [0.036,0.129]	0.109*** [0.034,0.183]	0.121*** [0.075,0.168]	0.100*** [0.059,0.142]	0.081*** [0.043,0.119]
CHI_AGE2	-0.001*** [-0.001,-0.001]						0.000 [-0.000,0.000]	-0.001*** [-0.002,-0.000]	0.000 [-0.001,0.000]	-0.001** [-0.002,-0.000]	-0.002*** [-0.003,-0.001]	-0.002*** [-0.002,-0.001]	-0.001** [-0.001,-0.000]
CHI_ORD	-0.060*** [-0.085,-0.036]						-0.154*** [-0.176,-0.131]	-0.034 [-0.088,0.019]	-0.071** [-0.133,-0.008]	-0.063* [-0.129,0.003]	-0.036 [-0.099,0.027]	-0.074** [-0.137,-0.011]	-0.087** [-0.157,-0.017]
CHI_SEX	-0.004 [-0.078,0.071]						0.212*** [0.140,0.284]	-0.075 [-0.229,0.079]	0.022 [-0.168,0.211]	0.045 [-0.155,0.245]	-0.147 [-0.359,0.066]	0.196** [0.003,0.390]	-0.006 [-0.183,0.170]
MOT_ANC	0.055*** [0.046,0.064]	-0.138*** [-0.146,-0.129]	0.057*** [0.049,0.064]	0.062*** [0.055,0.069]			-0.001 [-0.011,0.009]	0.094*** [0.072,0.115]	0.145*** [0.110,0.180]	0.064*** [0.028,0.101]	0.031*** [0.008,0.054]	0.041*** [0.020,0.061]	0.039*** [0.024,0.054]
MOT_EDM	0.061*** [0.043,0.079]	-0.065*** [-0.079,-0.050]	0.052*** [0.032,0.072]	0.057*** [0.043,0.071]	0.097*** [0.074,0.120]	0.031*** [0.024,0.038]	0.013 [-0.006,0.032]	0.070*** [0.035,0.105]	0.072*** [0.030,0.114]	0.067*** [0.021,0.114]	0.054* [-0.005,0.114]	0.053** [0.002,0.104]	0.043* [-0.003,0.089]
MOT_EDF	0.010 [-0.008,0.028]	-0.030*** [-0.045,-0.016]	0.033*** [0.011,0.054]	0.035*** [0.020,0.049]	0.083*** [0.063,0.102]	0.023*** [0.017,0.029]	0.028*** [0.010,0.045]	0.006 [-0.030,0.043]	0.006 [-0.036,0.048]	0.029 [-0.013,0.071]	0.016 [-0.046,0.079]	0.007 [-0.050,0.063]	-0.030 [-0.079,0.019]
MOT_AWE	2.972*** [2.848,3.096]	-0.916*** [-0.991,-0.840]	0.275*** [0.162,0.387]	0.984*** [0.907,1.061]	1.937*** [1.804,2.069]	0.640*** [0.600,0.680]		2.561*** [2.321,2.800]	2.882*** [2.574,3.189]	3.549*** [3.267,3.831]	2.629*** [2.241,3.018]	2.927*** [2.580,3.273]	2.959*** [2.586,3.333]
MOT_AGE	0.026*** [0.018,0.035]	-0.002 [-0.007,0.003]	0.001 [-0.005,0.008]	0.002 [-0.002,0.007]	0.011*** [0.005,0.018]	0.000 [-0.001,0.002]	0.010** [0.001,0.018]	0.017* [-0.001,0.034]	0.031** [0.007,0.054]	0.037*** [0.012,0.062]	0.013 [-0.011,0.036]	0.027** [0.006,0.048]	0.031*** [0.011,0.051]
HH_RUR	-0.087* [-0.190,0.016]	0.538*** [0.448,0.628]	-0.375*** [-0.485,-0.265]	-0.325*** [-0.408,-0.241]	-0.591*** [-0.818,-0.364]	-0.156*** [-0.206,-0.106]	-0.179*** [-0.282,-0.077]	-0.038 [-0.259,0.184]	-0.155 [-0.429,0.119]	-0.093 [-0.410,0.224]	-0.092 [-0.342,0.157]	-0.038 [-0.280,0.203]	-0.239* [-0.486,0.008]
HH_REL (ref: Catholic): Other Christian	-0.126* [-0.267,0.015]	0.095 [-0.029,0.218]	-0.095 [-0.217,0.027]	0.011 [-0.098,0.120]	-0.055 [-0.277,0.167]	-0.047* [-0.099,0.006]	-0.137 [-0.303,0.028]	-0.105 [-0.374,0.164]	-0.562* [-1.218,0.094]	0.465 [-0.245,1.175]	-0.109 [-0.337,0.119]	-0.409** [-0.760,-0.059]	-0.093 [-0.592,0.405]
HH_REL (ref: Catholic): Islam	-0.472*** [-0.644,-0.300]	0.229*** [0.079,0.379]	-0.238*** [-0.399,-0.077]	-0.028 [-0.163,0.106]	-0.167 [-0.465,0.132]	-0.100*** [-0.172,-0.027]	-0.069 [-0.263,0.126]	-0.379** [-0.682,-0.077]	-1.077*** [-1.745,-0.409]	-0.301 [-1.116,0.514]	-0.073 [-2.570,2.424]	0.020 [-0.754,0.793]	-0.403 [-0.920,0.114]
HH_REL (ref: Catholic): Traditionalist	-0.548*** [-0.955,-0.141]	1.008*** [0.650,1.366]	-0.914*** [-1.383,-0.445]	-0.478*** [-0.842,-0.115]	-0.650*** [-1.127,-0.173]	-0.349*** [-0.480,-0.218]	-0.091 [-0.463,0.281]	-0.697 [-1.815,0.421]	-1.628** [-3.137,-0.120]	-0.649 [-2.242,0.944]	-0.246 [-0.881,0.389]	-0.724 [-1.736,0.288]	-0.190 [-1.479,1.099]
HH_REL (ref: Catholic): Other	-0.224 [-1.244,0.795]	-0.259 [-1.018,0.500]	0.021 [-1.253,1.294]	0.383 [-0.377,1.144]	-0.057 [-1.495,1.382]	-0.135 [-0.672,0.401]	0.573 [-0.601,1.747]	0.000 [0.000,0.000]	0.000 [0.000,0.000]	0.000 [0.000,0.000]	0.000 [0.000,0.000]	-0.301 [-1.532,0.929]	0.946 [-3.331,5.223]
HH_ETH (ref: Ekoi): Fulani	-0.926*** [-1.433,-0.418]	0.673*** [0.240,1.105]	-0.070 [-0.974,0.834]	-0.768*** [-1.192,-0.343]	-0.745 [-2.016,0.527]	-0.347*** [-0.587,-0.107]	-0.186 [-0.813,0.440]	-0.085 [-0.631,0.460]	-0.060 [-0.361,0.242]	0.110 [-0.568,0.787]	0.000 [0.000,0.000]		-0.692 [-1.755,0.371]
HH_ETH (ref: Ekoi): Hausa	-0.847*** [-1.334,-0.361]	0.945*** [0.525,1.364]	-0.572 [-1.433,0.290]	-0.828*** [-1.239,-0.416]	-0.437 [-1.703,0.829]	-0.250** [-0.487,-0.013]	-0.504 [-1.122,0.115]	-0.483** [-0.872,-0.095]	-0.073 [-0.378,0.231]	0.361 [-0.160,0.882]	-1.194 [-3.028,0.639]	-1.657 [-3.919,0.605]	-0.229 [-1.129,0.670]
HH_ETH (ref: Ekoi): Ibibio	-0.633** [-1.168,-0.098]	0.372 [-0.101,0.844]	0.555 [-0.341,1.451]	-0.301 [-0.762,0.160]	-0.212 [-1.478,1.055]	-0.103 [-0.385,0.178]	-0.477 [-1.159,0.204]	-0.665 [-2.776,1.446]	0.000 [0.000,0.000]	0.000 [0.000,0.000]	-0.358 [-2.952,2.236]	-0.902*** [-1.429,-0.374]	1.125 [-0.229,2.479]
HH_ETH (ref: Ekoi): Igala	-0.657** [-1.243,-0.071]	-0.513* [-1.038,0.012]	1.298*** [0.405,2.190]	0.040 [-0.448,0.528]	0.004 [-1.313,1.322]	0.276* [-0.016,0.568]	-0.400 [-1.112,0.313]	-0.132 [-0.525,0.260]	0.000 [0.000,0.000]	0.000 [0.000,0.000]	-0.644 [-3.219,1.932]	-0.762 [-3.465,1.941]	0.698 [-0.640,2.036]
HH_ETH (ref: Ekoi): Igbo	-0.450* [-0.919,0.019]	-0.795*** [-1.211,-0.379]	1.881*** [1.051,2.711]	-0.568*** [-0.972,-0.164]	1.248* [-0.035,2.531]	0.120 [-0.111,0.351]	-0.528* [-1.138,0.081]	-0.163 [-0.690,0.364]	0.534 [-1.143,2.211]	0.274 [-0.817,1.364]	-0.896 [-2.200,0.408]	-0.502* [-1.066,0.062]	0.524** [0.017,1.032]
HH_ETH (ref: Ekoi): Ijaw / Izon	-0.486* [-1.027,0.055]	0.883*** [0.397,1.369]	0.118 [-0.807,1.042]	-0.806*** [-1.282,-0.331]	-0.696 [-2.066,0.675]	-0.134 [-0.396,0.127]	-0.260 [-0.915,0.395]	0.000 [0.000,0.000]			0.000 [0.000,0.000]	-0.656** [-1.158,-0.153]	-0.636 [-2.153,0.882]
HH_ETH (ref: Ekoi): Kanuri / Beriberi	-0.987*** [-1.591,-0.382]	1.001*** [0.511,1.492]	-0.518 [-1.659,0.622]	-0.956*** [-1.436,-0.476]	-0.512 [-1.801,0.777]	-0.237* [-0.500,0.026]	-0.271 [-0.930,0.388]	-0.661 [-2.271,0.948]	-0.178 [-0.644,0.287]	0.639 [-0.618,1.895]		0.000 [0.000,0.000]	2.778* [-0.171,5.727]
HH_ETH (ref: Ekoi): Tiv	-0.601** [-1.158,-0.045]	-0.775*** [-1.247,-0.303]	1.727*** [0.847,2.608]	0.134 [-0.333,0.601]	-0.904 [-2.245,0.437]	-0.343** [-0.609,-0.077]	-0.365 [-1.017,0.287]	-0.104 [-0.480,0.272]	-0.251 [-1.192,0.690]	0.000 [0.000,0.000]	0.000 [0.000,0.000]	0.000 [0.000,0.000]	1.786* [-0.016,3.588]
HH_ETH (ref: Ekoi): Yoruba	-0.570** [-1.048,-0.093]	-0.165 [-0.586,0.257]	1.300*** [0.465,2.136]	-0.413** [-0.822,-0.004]	2.235*** [0.950,3.519]	0.059 [-0.174,0.291]	-0.048 [-0.669,0.574]	0.187 [-0.142,0.516]	0.329 [-1.144,1.801]	0.649 [-0.692,1.989]	0.000 [0.000,0.000]	-2.187*** [-3.154,-1.221]	0.292* [-0.006,0.590]
HH_ETH (ref: Ekoi): Other	-0.616*** [-1.077,-0.154]	0.206 [-0.196,0.607]	0.912** [0.083,1.740]	-0.349* [-0.744,0.045]	0.030 [-1.220,1.279]	-0.017 [-0.247,0.213]	-0.361 [-0.967,0.245]	0.000 [0.000,0.000]	0.000 [0.000,0.000]	0.000 [0.000,0.000]	0.000 [0.000,0.000]	-0.887*** [-1.357,-0.417]	0.000 [0.000,0.000]
HH_SIZ	-0.013** [-0.026,-0.000]	0.033*** [0.023,0.042]	-0.017** [-0.032,-0.003]	-0.020*** [-0.030,-0.011]	-0.043*** [-0.056,-0.030]	-0.006*** [-0.010,-0.002]	0.132*** [0.118,0.146]	-0.011 [-0.036,0.015]	-0.021* [-0.047,0.004]	-0.008 [-0.036,0.021]	-0.011 [-0.051,0.029]	-0.019 [-0.063,0.025]	-0.011 [-0.058,0.036]
HH_WEA	0.142*** [0.122,0.162]	-0.206*** [-0.223,-0.189]	0.120*** [0.099,0.141]	0.129*** [0.114,0.145]	0.287*** [0.256,0.318]	0.059*** [0.050,0.067]	0.016 [-0.004,0.036]	0.149*** [0.106,0.192]	0.102*** [0.048,0.155]	0.132*** [0.078,0.185]	0.165*** [0.113,0.216]	0.121*** [0.074,0.168]	0.142*** [0.090,0.195]
YEAR (ref: 2003): 2008	0.578*** [0.302,0.854]	0.021 [-0.164,0.206]	-0.203* [-0.416,0.009]	0.056 [-0.120,0.232]	-0.569*** [-0.967,-0.170]	-0.080* [-0.169,0.008]	0.268** [0.062,0.473]	0.808*** [0.258,1.358]	0.797* [-0.064,1.659]	1.411*** [0.469,2.352]	0.666* [-0.076,1.407]	0.675* [-0.121,1.472]	-0.271 [-0.966,0.425]
YEAR (ref: 2003): 2013	0.726*** [0.457,0.996]	-0.033 [-0.226,0.159]	-0.354*** [-0.576,-0.132]	0.131 [-0.051,0.312]	-0.217 [-0.635,0.201]	-0.005 [-0.097,0.088]	0.330*** [0.110,0.550]	0.760*** [0.231,1.289]	1.279*** [0.434,2.125]	1.883*** [0.837,2.928]	0.724** [0.003,1.445]	1.410*** [0.626,2.195]	-0.820** [-1.492,-0.148]
YEAR (ref: 2003): 2018	0.808*** [0.532,1.083]	-0.228** [-0.417,-0.038]	-0.412*** [-0.637,-0.188]	0.342*** [0.162,0.522]	-1.275*** [-1.685,-0.866]	-0.051 [-0.145,0.043]	0.282*** [0.075,0.488]	1.141*** [0.583,1.699]	1.521*** [0.685,2.356]	1.159** [0.248,2.070]	1.034** [0.246,1.823]	1.843*** [0.981,2.705]	-0.801** [-1.517,-0.086]
Constant	-5.234*** [-5.846,-4.623]	2.038*** [1.561,2.515]	-3.645*** [-4.533,-2.756]	-2.427*** [-2.893,-1.961]	3.586*** [2.215,4.958]	0.980*** [0.718,1.243]	2.596*** [1.919,3.273]	-5.701*** [-6.558,-4.844]	-5.778*** [-6.960,-4.596]	-8.231*** [-9.639,-6.823]	-4.939*** [-6.647,-3.231]	-5.672*** [-6.916,-4.428]	-4.425*** [-5.521,-3.330]
Multilevel variance parameter: Level 1	0.285*** [0.218,0.352]	0.451*** [0.364,0.538]	0.710*** [0.552,0.868]	0.463*** [0.384,0.543]	0.672*** [0.585,0.759]	-1.437*** [-1.535,-1.340]	0.041*** [0.013,0.068]	0.171*** [0.065,0.277]	0.146** [0.032,0.261]	0.705*** [0.388,1.021]	0.168** [0.034,0.303]	0.029 [-0.038,0.097]	0.243*** [0.088,0.398]
Multilevel variance parameter: Residual					1.322*** [1.282,1.361]	0.090*** [0.064,0.116]							
Level 1 Observations (child)	24381	34713	34713	34713	35019	36585	52431	4342	5017	7249	2036	2514	3196
Level 2 Observations (LGA)	684	686	686	686	686	687	689	113	100	166	85	111	121
Akaike Information Criterion	18035.652	26423.512	17597.951	30317.164	193631.04	111221.63	23679.445	4154.682	2852.628	2871.344	2200.745	2604.453	3247.815
Prob. > X^2	<0.001	<0.001	<0.001	<0.001	<0.001	<0.001	<0.001	<0.001	<0.001	<0.001	<0.001	<0.001	<0.001

95% confidence intervals in brackets. NID = National Immunisation Day; sNID = sub-National Immunisation Day; IPD = Immunisation-Plus Day; sIPD = sub-national Immunisation-Plus Day; MNCHW = Maternal, Neonatal, and Child Health Week; SIA = Supplementary Immunisation Activity.

* p < 0.10, ** p < 0.05, *** p < 0.01

Table 6e: Additional robustness checks: fixed effects models

Dependent variables: various indicators of routine immunisation, maternal care and child survival (see right)	**Non-polio full immunisation**		**Delivery**			**Antenatal care**		**Child**
	Full model	Interaction model (EXPxAGE)	At home	At private facility	At public facility	No. of antenatal care visits	No. of tetanus injections	Total exposure
EXP_CHI	-0.020**	0.034**						
	[-0.036,-0.004]	[0.006,0.062]						
EXPxAGE		-0.001***						
		[-0.002,-0.001]						
EXP_PREG			-0.016	0.007	0.011	0.023	0.012***	
			[-0.038,0.006]	[-0.023,0.037]	[-0.009,0.032]	[-0.005,0.052]	[0.004,0.021]	
EXP_TOT_nod (total exposure, date approximation)								-0.029***
								[-0.041,-0.017]
CHI_AGE	0.080***	0.036***						0.001
	[0.064,0.095]	[0.028,0.043]						[-0.010,0.011]
CHI_AGE2	-0.001***							0.000
	[-0.001,-0.001]							[-0.000,0.000]
CHI_ORD	-0.059***	-0.060***						-0.148***
	[-0.085,-0.034]	[-0.085,-0.034]						[-0.171,-0.125]
CHI_SEX	-0.001	-0.003						0.212***
	[-0.078,0.076]	[-0.080,0.074]						[0.139,0.285]
MOT_ANC	0.059***	0.059***	-0.133***	0.047***	0.063***			0.003
	[0.049,0.068]	[0.049,0.068]	[-0.142,-0.125]	[0.039,0.055]	[0.056,0.070]			[-0.008,0.013]
MOT_EDM	0.060***	0.060***	-0.059***	0.040***	0.053***	0.091***	0.027***	0.015
	[0.042,0.079]	[0.042,0.079]	[-0.074,-0.044]	[0.019,0.061]	[0.038,0.067]	[0.070,0.112]	[0.021,0.033]	[-0.005,0.035]
MOT_EDF	0.002	0.002	-0.028***	0.018	0.033***	0.078***	0.019***	0.033***
	[-0.017,0.021]	[-0.017,0.021]	[-0.043,-0.013]	[-0.004,0.040]	[0.019,0.048]	[0.059,0.097]	[0.014,0.025]	[0.015,0.051]
MOT_AWE	2.988***	2.984***	-0.904***	0.237***	0.951***	1.899***	0.615***	
	[2.858,3.117]	[2.854,3.113]	[-0.982,-0.827]	[0.123,0.352]	[0.873,1.030]	[1.796,2.002]	[0.586,0.645]	
MOT_AGE	0.026***	0.027***	-0.002	-0.002	0.002	0.010***	0.000	0.008*
	[0.018,0.035]	[0.018,0.036]	[-0.007,0.004]	[-0.009,0.005]	[-0.002,0.007]	[0.003,0.016]	[-0.002,0.002]	[-0.000,0.017]
HH_RUR	-0.088	-0.091	0.531***	-0.261***	-0.368***	-0.527***	-0.169***	-0.174**
	[-0.211,0.035]	[-0.214,0.033]	[0.430,0.632]	[-0.381,-0.141]	[-0.459,-0.276]	[-0.662,-0.391]	[-0.207,-0.131]	[-0.308,-0.041]
HH_REL (ref: Catholic): Other Christian	-0.117	-0.119	0.036	-0.034	0.001	-0.063	-0.029	-0.113
	[-0.267,0.032]	[-0.268,0.031]	[-0.093,0.164]	[-0.159,0.091]	[-0.112,0.113]	[-0.240,0.113]	[-0.079,0.021]	[-0.292,0.066]
HH_REL (ref: Catholic): Islam	-0.407***	-0.406***	0.150*	-0.073	-0.041	-0.041	-0.033	-0.054
	[-0.594,-0.219]	[-0.593,-0.218]	[-0.009,0.309]	[-0.240,0.094]	[-0.182,0.100]	[-0.258,0.175]	[-0.094,0.029]	[-0.273,0.165]
HH_REL (ref: Catholic): Traditionalist	-0.530**	-0.528**	0.952***	-0.899***	-0.492***	-0.577***	-0.305***	-0.101
	[-0.953,-0.107]	[-0.951,-0.105]	[0.582,1.322]	[-1.369,-0.428]	[-0.864,-0.121]	[-0.985,-0.169]	[-0.421,-0.188]	[-0.492,0.290]
HH_REL (ref: Catholic): Other	-0.430	-0.433	-0.411	0.109	0.395	-0.065	-0.093	0.670
	[-1.507,0.648]	[-1.508,0.641]	[-1.197,0.375]	[-1.200,1.418]	[-0.393,1.182]	[-1.222,1.093]	[-0.417,0.231]	[-0.529,1.868]
HH_ETH (ref: Ekoi): Fulani	-0.669**	-0.681**	0.762***	0.142	-0.693***	-0.693*	-0.300***	0.219
	[-1.279,-0.059]	[-1.292,-0.071]	[0.276,1.248]	[-0.903,1.187]	[-1.167,-0.219]	[-1.442,0.057]	[-0.516,-0.085]	[-0.607,1.046]
HH_ETH (ref: Ekoi): Hausa	-0.527*	-0.537*	0.919***	-0.152	-0.675***	-0.249	-0.149	-0.066
	[-1.124,0.069]	[-1.134,0.061]	[0.440,1.397]	[-1.158,0.854]	[-1.140,-0.210]	[-0.995,0.496]	[-0.363,0.065]	[-0.888,0.757]
HH_ETH (ref: Ekoi): Ibibio	-0.385	-0.388	0.236	0.322	-0.106	-0.354	-0.180	-0.203
	[-1.021,0.251]	[-1.025,0.248]	[-0.298,0.770]	[-0.712,1.356]	[-0.620,0.407]	[-1.185,0.477]	[-0.418,0.057]	[-1.114,0.707]
HH_ETH (ref: Ekoi): Igala	-0.482	-0.492	0.020	0.699	-0.098	-0.410	0.190	0.130
	[-1.206,0.241]	[-1.215,0.232]	[-0.569,0.609]	[-0.330,1.729]	[-0.643,0.448]	[-1.317,0.498]	[-0.070,0.451]	[-0.847,1.108]
HH_ETH (ref: Ekoi): Igbo	-0.167	-0.167	-0.526**	1.189**	-0.333	0.831**	-0.034	0.017
	[-0.751,0.417]	[-0.752,0.418]	[-1.007,-0.045]	[0.223,2.156]	[-0.792,0.125]	[0.082,1.581]	[-0.249,0.181]	[-0.821,0.855]
HH_ETH (ref: Ekoi): Ijaw / Izon	-0.302	-0.306	0.708**	-0.017	-0.599**	-0.842*	-0.163	0.490
	[-0.987,0.384]	[-0.992,0.380]	[0.147,1.269]	[-1.095,1.060]	[-1.143,-0.056]	[-1.688,0.005]	[-0.404,0.078]	[-0.445,1.425]
HH_ETH (ref: Ekoi): Kanuri / Beriberi	-0.488	-0.497	1.038***	-0.045	-0.887***	-0.382	-0.149	-0.082
	[-1.194,0.218]	[-1.203,0.209]	[0.495,1.581]	[-1.336,1.246]	[-1.415,-0.359]	[-1.176,0.412]	[-0.378,0.079]	[-0.945,0.780]
HH_ETH (ref: Ekoi): Tiv	-0.357	-0.373	-0.297	0.992*	0.087	-0.814*	-0.362***	0.209
	[-1.084,0.369]	[-1.100,0.354]	[-0.841,0.248]	[-0.036,2.020]	[-0.447,0.621]	[-1.670,0.042]	[-0.608,-0.116]	[-0.722,1.139]
HH_ETH (ref: Ekoi): Yoruba	-0.230	-0.238	0.291	0.702	-0.428*	1.347***	0.048	0.139
	[-0.826,0.365]	[-0.834,0.358]	[-0.194,0.776]	[-0.273,1.677]	[-0.894,0.038]	[0.589,2.104]	[-0.169,0.266]	[-0.710,0.988]
HH_ETH (ref: Ekoi): Other	-0.393	-0.395	0.372	0.595	-0.317	-0.007	-0.006	-0.023
	[-0.958,0.171]	[-0.960,0.170]	[-0.083,0.828]	[-0.372,1.561]	[-0.761,0.126]	[-0.731,0.717]	[-0.214,0.202]	[-0.831,0.786]

HH_SIZ	-0.016**	-0.016**	0.033***	-0.014*	-0.022***	-0.039***	-0.007***	0.137***
	[-0.030,-0.003]	[-0.030,-0.003]	[0.023,0.043]	[-0.029,0.001]	[-0.032,-0.013]	[-0.052,-0.027]	[-0.011,-0.004]	[0.123,0.152]
HH_WEA	0.141***	0.140***	-0.204***	0.108***	0.131***	0.276***	0.058***	0.008
	[0.120,0.163]	[0.118,0.161]	[-0.221,-0.186]	[0.086,0.130]	[0.115,0.147]	[0.253,0.299]	[0.051,0.064]	[-0.014,0.030]
YEAR (ref: 2003): 2008	0.517***	0.495***	0.040	-0.251**	0.087	-0.670***	-0.108***	0.323***
	[0.235,0.799]	[0.211,0.778]	[-0.156,0.236]	[-0.472,-0.030]	[-0.097,0.270]	[-0.921,-0.420]	[-0.180,-0.037]	[0.097,0.549]
YEAR (ref: 2003): 2013	0.673***	0.635***	-0.042	-0.388***	0.170*	-0.314**	-0.029	0.547***
	[0.394,0.952]	[0.353,0.916]	[-0.237,0.153]	[-0.607,-0.169]	[-0.011,0.351]	[-0.563,-0.065]	[-0.100,0.043]	[0.313,0.780]
YEAR (ref: 2003): 2018	0.800***	0.890***	-0.267***	-0.385***	0.391***	-1.272***	-0.059	0.314***
	[0.512,1.087]	[0.603,1.177]	[-0.468,-0.066]	[-0.617,-0.153]	[0.203,0.578]	[-1.530,-1.013]	[-0.133,0.016]	[0.094,0.535]
Observations	24381	24381	34713	34713	34713	35019	36585	52431
Akaike Information Criterion	18032.806	18053.939	26153.968	17075.688	29973.334	192578.24	111031.373	24062.176
Prob. > X^2	<0.001	<0.001	<0.001	<0.001	<0.001	<0.001	<0.001	<0.001
Hausman test	0.068	0.023	<0.001	<0.001	<0.001	<0.001	<0.001	<0.001

95% confidence intervals in brackets. 774 LGA-level fixed effects estimators omitted from reporting.
* p < 0.10, ** p < 0.05, *** p < 0.01
