# Supplementary material for: Impact and effect mechanisms of mass campaigns in resource-constrained health systems: quasi-experimental evidence from polio eradication in Nigeria
Source: BMJ Glob Health. 2021 Mar 8;6(3):e004248. doi: 10.1136/bmjgh-2020-004248 (PMC7942242; doi:10.1136/bmjgh-2020-004248)
Supplement: Supplementary data [file bmjgh-2020-004248supp008.pdf]

Table 7: Detailed results: Link between SIA exposure and child/maternal health outcomes, stratified by region.

	Non-polio full immunisation		Delivery			Antenatal care		Child survival	
North Central	Full model	Interaction model (EXPxAGE)	At home	At private facility	At public facility	No. of antenatal care visits	No. of tetanus injections	Exposure decomposition	Total exposure
EXP_CHI	0.026*	0.054**							
	[-0.004, 0.056]	[0.009, 0.100]							
EXPxAGE		-0.001**							
		[-0.002, -0.000]							
EXP_PREG			0.003	-0.015	0.002	0.034	0.003		
			[-0.036, 0.042]	[-0.061, 0.031]	[-0.034, 0.038]	[-0.030, 0.097]	[-0.015, 0.021]		
EXP_PREG_nod (date approximation)								-0.019	
								[-0.076, 0.037]	
EXP_CHI_nod (date approximation)								0.030*	
								[-0.001, 0.061]	
EXP_TOT_nod (total exposure, date approximation)									0.018
									[-0.008, 0.044]
CHI_AGE	0.056***	0.017**						-0.003	-0.005
	[0.025, 0.087]	[0.001, 0.033]						[-0.027, 0.022]	[-0.029, 0.018]
CHI_AGE2	-0.001***							0.000	0.000
	[-0.001, -0.000]							[-0.001, 0.000]	[-0.000, 0.000]
CHI_ORD	-0.035	-0.034						-0.157***	-0.158***
	[-0.088, 0.019]	[-0.087, 0.019]						[-0.220, -0.095]	[-0.221, -0.095]
CHI_SEX	-0.077	-0.075						0.217**	0.218**
	[-0.230, 0.077]	[-0.228, 0.079]						[0.031, 0.404]	[0.032, 0.405]
MOT_ANC	0.093***	0.094***	-0.161***	0.061***	0.083***			0.004	0.004
	[0.071, 0.115]	[0.072, 0.115]	[-0.181, -0.142]	[0.043, 0.080]	[0.067, 0.100]			[-0.023, 0.031]	[-0.023, 0.031]
MOT_EDM	0.070***	0.070***	-0.057***	0.060***	0.031**	0.105***	0.028***	0.021	0.021
	[0.035, 0.104]	[0.035, 0.104]	[-0.085, -0.029]	[0.025, 0.096]	[0.004, 0.059]	[0.059, 0.151]	[0.012, 0.043]	[-0.022, 0.063]	[-0.022, 0.063]
MOT_EDF	0.005	0.005	-0.024	0.029	0.023	0.053***	0.008	0.010	0.010
	[-0.032, 0.042]	[-0.031, 0.042]	[-0.053, 0.005]	[-0.008, 0.067]	[-0.005, 0.052]	[0.016, 0.091]	[-0.008, 0.025]	[-0.033, 0.052]	[-0.032, 0.053]
MOT_AWE	2.554***	2.547***	-0.660***	-0.092	0.863***	1.760***	0.614***		
	[2.315, 2.793]	[2.309, 2.786]	[-0.809, -0.510]	[-0.287, 0.102]	[0.709, 1.018]	[1.484, 2.035]	[0.527, 0.701]		
MOT_AGE	0.016*	0.016*	0.005	-0.003	-0.003	0.026***	0.004	0.010	0.010
	[-0.001, 0.034]	[-0.001, 0.034]	[-0.005, 0.015]	[-0.016, 0.009]	[-0.013, 0.006]	[0.010, 0.043]	[-0.002, 0.010]	[-0.011, 0.031]	[-0.011, 0.031]
HH_RUR	-0.035	-0.033	0.750***	-0.405***	-0.425***	-0.559**	-0.096*	-0.079	-0.081
	[-0.257, 0.186]	[-0.255, 0.188]	[0.553, 0.946]	[-0.623, -0.187]	[-0.599, -0.251]	[-1.103, -0.014]	[-0.201, 0.009]	[-0.336, 0.177]	[-0.337, 0.176]
HH_REL (ref: Catholic): Other Christian	-0.105	-0.105	0.104	0.006	-0.106	0.160	0.037	0.164	0.165
	[-0.373, 0.163]	[-0.373, 0.163]	[-0.115, 0.324]	[-0.235, 0.246]	[-0.316, 0.105]	[-0.217, 0.536]	[-0.096, 0.170]	[-0.142, 0.471]	[-0.142, 0.471]
HH_REL (ref: Catholic): Islam	-0.371**	-0.370**	0.095	0.000	-0.094	0.612***	-0.069	0.118	0.117
	[-0.674, -0.069]	[-0.673, -0.068]	[-0.160, 0.350]	[-0.289, 0.290]	[-0.331, 0.143]	[0.149, 1.074]	[-0.232, 0.094]	[-0.220, 0.457]	[-0.222, 0.456]
HH_REL (ref: Catholic): Traditionalist	-0.719	-0.695	0.277	-0.011	-0.612	-0.985***	-0.625***	0.422	0.428
	[-1.837, 0.399]	[-1.811, 0.421]	[-0.415, 0.969]	[-0.861, 0.839]	[-1.424, 0.201]	[-1.728, -0.243]	[-0.918, -0.331]	[-0.523, 1.367]	[-0.518, 1.373]
HH_REL (ref: Catholic): Other	0.000	0.000	-0.391	0.960	-0.172	-0.750	-0.409	0.022	0.015
	[0.000, 0.000]	[0.000, 0.000]	[-1.789, 1.007]	[-0.656, 2.575]	[-1.783, 1.438]	[-2.634, 1.134]	[-1.097, 0.280]	[-2.033, 2.078]	[-2.042, 2.071]
HH_ETH (ref: Ekoi): Fulani	-0.085	-0.065	0.749***	-1.294***	-0.500**	0.000	0.000	0.364	0.370
	[-0.632, 0.462]	[-0.611, 0.481]	[0.286, 1.212]	[-2.219, -0.368]	[-0.939, -0.061]	[0.000, 0.000]	[0.000, 0.000]	[-0.165, 0.892]	[-0.158, 0.898]
HH_ETH (ref: Ekoi): Hausa	-0.492**	-0.489**	0.854***	-0.647***	-0.546***	0.657*	0.305**	0.079	0.078
	[-0.881, -0.104]	[-0.877, -0.101]	[0.538, 1.170]	[-1.117, -0.177]	[-0.850, -0.243]	[-0.026, 1.340]	[0.031, 0.580]	[-0.301, 0.460]	[-0.303, 0.458]
HH_ETH (ref: Ekoi): Ibibio	-0.624	-0.628	-0.658	0.903	-0.511	1.908***	0.364	0.000	0.000
	[-2.729, 1.481]	[-2.732, 1.477]	[-2.862, 1.546]	[-0.485, 2.291]	[-1.878, 0.856]	[0.643, 3.172]	[-0.234, 0.962]	[0.000, 0.000]	[0.000, 0.000]
HH_ETH (ref: Ekoi): Igala	-0.139	-0.141	-0.579***	0.276	0.418***	1.477***	0.848***	0.019	0.015
	[-0.531, 0.253]	[-0.533, 0.251]	[-0.962, -0.195]	[-0.102, 0.653]	[0.102, 0.733]	[0.605, 2.349]	[0.551, 1.145]	[-0.407, 0.445]	[-0.411, 0.441]
HH_ETH (ref: Ekoi): Igbo	-0.163	-0.151	-1.009***	0.672***	0.025	1.672***	0.249*	0.217	0.214
	[-0.689, 0.364]	[-0.677, 0.374]	[-1.546, -0.471]	[0.274, 1.069]	[-0.341, 0.391]	[0.809, 2.535]	[-0.043, 0.541]	[-0.491, 0.926]	[-0.495, 0.923]
HH_ETH (ref: Ekoi): Ijaw / Izon	0.000	0.000	0.000	0.000	0.000	-1.779	0.427**	0.000	0.000
	[0.000, 0.000]	[0.000, 0.000]	[0.000, 0.000]	[0.000, 0.000]	[0.000, 0.000]	[-4.231, 0.674]	[0.080, 0.774]	[0.000, 0.000]	[0.000, 0.000]
HH_ETH (ref: Ekoi): Kanuri / Beriberi	-0.722	-0.707	0.647	0.100	-0.611	2.708**	0.213	-0.902*	-0.888
	[-2.342, 0.898]	[-2.327, 0.913]	[-0.406, 1.701]	[-1.070, 1.270]	[-1.666, 0.444]	[0.535, 4.880]	[-0.284, 0.710]	[-1.967, 0.162]	[-1.952, 0.176]
HH_ETH (ref: Ekoi): Tiv	-0.116	-0.118	-0.808***	0.945***	0.095	0.824**	0.218*	0.088	0.084
	[-0.491, 0.260]	[-0.493, 0.257]	[-1.135, -0.481]	[0.578, 1.312]	[-0.207, 0.398]	[0.013, 1.635]	[-0.009, 0.445]	[-0.253, 0.429]	[-0.258, 0.425]
HH_ETH (ref: Ekoi): Yoruba	0.183	0.191	-0.267	0.600***	-0.310**	2.871***	0.472***	0.301	0.305
	[-0.145, 0.511]	[-0.137, 0.518]	[-0.587, 0.052]	[0.297, 0.904]	[-0.568, -0.051]	[2.117, 3.624]	[0.214, 0.731]	[-0.089, 0.692]	[-0.086, 0.695]
HH_ETH (ref: Ekoi): Other	0.000	0.000	0.000	0.000	0.000	1.335***	0.516***	0.000	0.000
	[0.000, 0.000]	[0.000, 0.000]	[0.000, 0.000]	[0.000, 0.000]	[0.000, 0.000]	[0.666, 2.004]	[0.319, 0.713]	[0.000, 0.000]	[0.000, 0.000]
HH_SIZ	-0.012	-0.012	0.007	0.003	-0.007	-0.067***	-0.014***	0.113***	0.113***
	[-0.037, 0.014]	[-0.038, 0.013]	[-0.012, 0.025]	[-0.020, 0.027]	[-0.026, 0.011]	[-0.098, -0.036]	[-0.022, -0.005]	[0.076, 0.149]	[0.076, 0.150]
	0.150***	0.149***	-0.182***	0.088***	0.116***	0.304***	0.055***	0.015	0.015

HH_WEA	[0.107, 0.193]	[0.106, 0.192]	[-0.218, -0.146]	[0.046, 0.129]	[0.082, 0.149]	[0.225, 0.384]	[0.032, 0.078]	[-0.035, 0.065]	[-0.036, 0.065]
YEAR (ref: 2003): 2008	0.988***	0.989***	-0.331*	-0.344*	0.562***	-1.690***	-0.205	0.183	0.170
	[0.475, 1.501]	[0.474, 1.504]	[-0.691, 0.029]	[-0.742, 0.053]	[0.209, 0.915]	[-2.851, -0.529]	[-0.486, 0.077]	[-0.301, 0.667]	[-0.314, 0.653]
YEAR (ref: 2003): 2013	0.818***	0.837***	-0.433**	-0.216	0.537***	-1.049*	-0.120	0.412	0.406
	[0.311, 1.326]	[0.328, 1.345]	[-0.792, -0.075]	[-0.607, 0.175]	[0.189, 0.886]	[-2.289, 0.191]	[-0.393, 0.153]	[-0.093, 0.917]	[-0.098, 0.911]
YEAR (ref: 2003): 2018	1.134***	1.183***	-0.775***	-0.072	0.787***	-2.272***	-0.304**	0.398	0.376
	[0.609, 1.659]	[0.660, 1.707]	[-1.150, -0.400]	[-0.488, 0.343]	[0.421, 1.153]	[-3.442, -1.101]	[-0.579, -0.030]	[-0.090, 0.886]	[-0.111, 0.863]
Constant	-5.527***	-5.158***	1.901***	-2.371***	-2.459***	1.800***	0.579***	1.877***	1.829***
	[-6.348, -4.707]	[-5.930, -4.386]	[1.358, 2.444]	[-3.004, -1.737]	[-2.986, -1.932]	[0.559, 3.042]	[0.175, 0.983]	[1.097, 2.657]	[1.053, 2.606]
Multilevel variance parameter: Level 1	0.172***	0.172***	0.338***	0.410***	0.186***	0.030	-1.683***	0.000	0.000
	[0.066, 0.279]	[0.066, 0.278]	[0.185, 0.490]	[0.193, 0.628]	[0.090, 0.283]	[-0.159, 0.219]	[-2.031, -1.334]	[-0.000, 0.000]	[-0.000, 0.000]
Multilevel variance parameter: Residual						1.263***	0.254***		
						[1.193, 1.333]	[0.199, 0.309]		
Observations (Level 2)	4342	4342	6071	6071	6071	6103	6293	9028	9028
Observations (Level 1)	113	113	113	113	113	113	113	113	113
Akaike Information Criterion	4152.478	4159.676	6199.652	4691.431	6657.883	32962.923	21185.627	3710.053	3710.113
Prob. > X^2	<0.001	<0.001	<0.001	<0.001	<0.001	<0.001	<0.001	<0.001	<0.001

North East	Non-polio full immunisation		Delivery			Antenatal care		Child survival	
	**Full model**	**Interaction model (EXPxAGE)**	**At home**	**At private facility**	**At public facility**	**No. of antenatal care visits**	**No. of tetanus injections**	**Exposure decompositio n**	**Total exposure**
EXP_CHI	-0.074***	-0.058**							
	[-0.106, -0.042]	[-0.104, -0.013]							
EXPxAGE		0.000							
		[-0.001, 0.001]							
EXP_PREG			-0.025	0.024	0.018	0.015	0.001		
			[-0.067, 0.017]	[-0.103, 0.151]	[-0.024, 0.060]	[-0.026, 0.055]	[-0.017, 0.019]		
EXP_PREG_nod (date approximation)								0.019	
								[-0.022, 0.060]	
EXP_CHI_nod (date approximation)								0.005	
								[-0.020, 0.029]	
EXP_TOT_nod (total exposure, date approximation)									0.008
									[-0.013, 0.030]
CHI_AGE	0.085***	0.054***						-0.014	-0.014
	[0.043, 0.128]	[0.032, 0.077]						[-0.035, 0.008]	[-0.035, 0.007]
CHI_AGE2	-0.001*							0.000	0.000
	[-0.001, 0.000]							[-0.000, 0.000]	[-0.000, 0.000]
CHI_ORD	-0.072**	-0.073**						-0.139***	-0.139***
	[-0.135, -0.010]	[-0.136, -0.011]						[-0.186, -0.093]	[-0.186, -0.093]
CHI_SEX	0.026	0.024						0.271***	0.271***
	[-0.163, 0.215]	[-0.165, 0.213]						[0.121, 0.421]	[0.121, 0.420]
MOT_ANC	0.144***	0.144***	-0.218***	0.134***	0.203***			0.003	0.003
	[0.109, 0.179]	[0.109, 0.179]	[-0.244, -0.192]	[0.069, 0.200]	[0.178, 0.229]			[-0.026, 0.031]	[-0.026, 0.031]
MOT_EDM	0.072***	0.071***	-0.083***	0.120***	0.072***	0.092***	0.030***	-0.009	-0.009
	[0.030, 0.115]	[0.029, 0.114]	[-0.114, -0.051]	[0.030, 0.210]	[0.040, 0.104]	[0.054, 0.130]	[0.016, 0.044]	[-0.050, 0.031]	[-0.049, 0.031]
MOT_EDF	0.006	0.006	-0.028*	0.020	0.031*	0.104***	0.029***	0.038**	0.038**
	[-0.036, 0.048]	[-0.036, 0.047]	[-0.059, 0.003]	[-0.072, 0.112]	[-0.000, 0.062]	[0.071, 0.137]	[0.013, 0.044]	[0.002, 0.074]	[0.003, 0.074]
MOT_AWE	2.870***	2.872***	-0.834***	0.801***	0.806***	1.847***	0.740***		
	[2.563, 3.177]	[2.565, 3.179]	[-0.998, -0.669]	[0.290, 1.311]	[0.640, 0.973]	[1.626, 2.068]	[0.663, 0.818]		
MOT_AGE	0.031***	0.031***	0.007	0.012	-0.009	0.001	0.000	0.008	0.008
	[0.008, 0.055]	[0.007, 0.055]	[-0.004, 0.019]	[-0.021, 0.046]	[-0.021, 0.003]	[-0.009, 0.010]	[-0.003, 0.004]	[-0.010, 0.026]	[-0.010, 0.026]
HH_RUR	-0.159	-0.161	0.601***	0.123	-0.609***	-0.671***	-0.312***	-0.191	-0.190
	[-0.433, 0.115]	[-0.435, 0.114]	[0.388, 0.814]	[-0.501, 0.748]	[-0.817, -0.400]	[-1.014, -0.328]	[-0.443, -0.182]	[-0.432, 0.050]	[-0.430, 0.051]
HH_REL (ref: Catholic): Other Christian	-0.546	-0.558*	-0.366	0.534	0.338	0.285	0.114	-0.898**	-0.895**
	[-1.203, 0.112]	[-1.214, 0.098]	[-0.866, 0.133]	[-0.444, 1.512]	[-0.160, 0.836]	[-0.279, 0.848]	[-0.088, 0.315]	[-1.663, -0.132]	[-1.661, -0.129]
HH_REL (ref: Catholic): Islam	-1.072***	-1.088***	-0.018	0.105	0.051	0.073	0.050	-0.776**	-0.772**
	[-1.741, -0.402]	[-1.756, -0.420]	[-0.531, 0.495]	[-0.941, 1.151]	[-0.460, 0.562]	[-0.518, 0.663]	[-0.159, 0.260]	[-1.548, -0.004]	[-1.544, -0.000]
HH_REL (ref: Catholic): Traditionalist	-1.618**	-1.630**	0.789	0.000	-0.609	-0.676*	-0.263	-0.562	-0.560
	[-3.129, -0.107]	[-3.136, -0.124]	[-0.463, 2.042]	[0.000, 0.000]	[-1.842, 0.624]	[-1.376, 0.024]	[-0.583, 0.056]	[-1.751, 0.628]	[-1.749, 0.629]
HH_REL (ref: Catholic): Other	0.000	0.000	0.000	0.000	0.000	-2.056***	-0.787***	0.000	0.000
	[0.000, 0.000]	[0.000, 0.000]	[0.000, 0.000]	[0.000, 0.000]	[0.000, 0.000]	[-2.585, -1.526]	[-0.978, -0.596]	[0.000, 0.000]	[0.000, 0.000]
HH_ETH (ref: Ekoi): Fulani	-0.057	-0.065	-0.039	0.172	0.026	0.000	0.000	0.172	0.172
	[-0.358, 0.245]	[-0.366, 0.236]	[-0.265, 0.188]	[-0.518, 0.861]	[-0.201, 0.253]	[0.000, 0.000]	[0.000, 0.000]	[-0.049, 0.393]	[-0.049, 0.393]
HH_ETH (ref: Ekoi): Hausa	-0.078	-0.082	0.209*	-0.302	-0.168	0.552***	0.128**	0.005	0.004
	[-0.382, 0.226]	[-0.387, 0.222]	[-0.019, 0.438]	[-1.060, 0.455]	[-0.396, 0.059]	[0.197, 0.907]	[0.025, 0.231]	[-0.224, 0.234]	[-0.224, 0.233]
HH_ETH (ref: Ekoi): Ibibio	0.000	0.000	0.000	0.000	0.000	-1.698***	-0.297***	0.000	0.000
	[0.000, 0.000]	[0.000, 0.000]	[0.000, 0.000]	[0.000, 0.000]	[0.000, 0.000]	[-2.025, -1.371]	[-0.447, -0.146]	[0.000, 0.000]	[0.000, 0.000]
HH_ETH (ref: Ekoi): Igala	0.000	0.000	-0.981	2.001	0.221	0.707	0.077	-1.173	-1.184
	[0.000, 0.000]	[0.000, 0.000]	[-3.004, 1.041]	[-1.253, 5.254]	[-1.710, 2.152]	[-1.352, 2.765]	[-0.733, 0.888]	[-3.300, 0.954]	[-3.311, 0.943]
HH_ETH (ref: Ekoi): Igbo	0.487	0.501	0.000	1.367**	0.292	3.466***	0.444**	-0.922	-0.925
	[-1.189, 2.162]	[-1.172, 2.174]	[0.000, 0.000]	[0.048, 2.687]	[-0.831, 1.415]	[1.887, 5.045]	[0.091, 0.796]	[-2.188, 0.343]	[-2.191, 0.341]
HH_ETH (ref: Ekoi): Ijaw / Izon									
HH_ETH (ref: Ekoi): Kanuri / Beriberi	-0.220	-0.218	0.454***	-0.625	-0.405**	-0.059	-0.021	0.121	0.123
	[-0.684, 0.245]	[-0.683, 0.246]	[0.124, 0.784]	[-1.938, 0.688]	[-0.732, -0.078]	[-0.373, 0.255]	[-0.149, 0.107]	[-0.183, 0.425]	[-0.181, 0.426]
HH_ETH (ref: Ekoi): Tiv	-0.200	-0.236	-1.446***	-0.044	1.613***	-0.345	-0.123	0.065	0.069
	[-1.146, 0.746]	[-1.182, 0.710]	[-2.017, -0.874]	[-1.268, 1.180]	[1.035, 2.191]	[-1.423, 0.733]	[-0.585, 0.340]	[-0.716, 0.847]	[-0.712, 0.851]
HH_ETH (ref: Ekoi): Yoruba	0.351	0.367	-0.990	1.845**	0.112	0.855	0.000	0.000	0.000
	[-1.116, 1.818]	[-1.095, 1.829]	[-2.250, 0.270]	[0.224, 3.467]	[-1.022, 1.245]	[-1.072, 2.783]	[-0.583, 0.584]	[0.000, 0.000]	[0.000, 0.000]

HH_ETH (ref: Ekoi): Other	0.000	0.000	0.000	0.000	0.000	0.415***	0.191***	0.000	0.000
	[0.000, 0.000]	[0.000, 0.000]	[0.000, 0.000]	[0.000, 0.000]	[0.000, 0.000]	[0.151, 0.679]	[0.085, 0.298]	[0.000, 0.000]	[0.000, 0.000]
HH_SIZ	-0.022*	-0.021*	0.032***	-0.032	-0.028***	-0.015*	-0.007	0.096***	0.096***
	[-0.047, 0.003]	[-0.047, 0.004]	[0.013, 0.051]	[-0.090, 0.027]	[-0.046, -0.009]	[-0.033, 0.002]	[-0.016, 0.002]	[0.071, 0.121]	[0.071, 0.121]
HH_WEA	0.103***	0.103***	-0.237***	0.040	0.230***	0.240***	0.071***	0.033	0.033
	[0.050, 0.157]	[0.049, 0.156]	[-0.279, -0.196]	[-0.073, 0.154]	[0.188, 0.271]	[0.185, 0.295]	[0.051, 0.091]	[-0.015, 0.081]	[-0.015, 0.081]
YEAR (ref: 2003): 2008	0.788*	0.799*	0.726***	-1.900***	-0.354	0.217	0.109	0.097	0.108
	[-0.031, 1.608]	[-0.024, 1.622]	[0.299, 1.153]	[-2.812, -0.988]	[-0.790, 0.083]	[-0.214, 0.649]	[-0.068, 0.286]	[-0.318, 0.512]	[-0.305, 0.521]
YEAR (ref: 2003): 2013	1.338***	1.358***	0.396*	-1.756***	-0.054	0.217	0.224**	0.184	0.193
	[0.525, 2.151]	[0.542, 2.174]	[-0.026, 0.818]	[-2.679, -0.833]	[-0.484, 0.377]	[-0.198, 0.632]	[0.026, 0.421]	[-0.247, 0.615]	[-0.237, 0.623]
YEAR (ref: 2003): 2018	1.692***	1.741***	0.091	-1.457***	0.258	-0.034	0.308***	0.115	0.138
	[0.865, 2.518]	[0.915, 2.568]	[-0.352, 0.534]	[-2.441, -0.473]	[-0.195, 0.711]	[-0.467, 0.400]	[0.110, 0.505]	[-0.303, 0.532]	[-0.273, 0.548]
Constant	-5.962***	-5.653***	2.646***	-5.294***	-2.948***	1.088***	0.410***	3.024***	3.046***
	[-7.134, -4.790]	[-6.771, -4.535]	[1.930, 3.362]	[-7.033, -3.555]	[-3.666, -2.230]	[0.398, 1.778]	[0.106, 0.714]	[2.074, 3.974]	[2.099, 3.993]
Multilevel variance parameter: Level 1	0.158**	0.163***	0.317***	1.656***	0.237***	-0.479***	-1.597***	0.053*	0.052*
	[0.037, 0.278]	[0.040, 0.286]	[0.160, 0.475]	[0.508, 2.804]	[0.100, 0.374]	[-0.643, -0.316]	[-1.924, -1.270]	[-0.007, 0.113]	[-0.008, 0.112]
Multilevel variance parameter: Residual						0.924***	0.079***		
						[0.864, 0.985]	[0.035, 0.123]		
Observations (Level 2)	5017	5017	7350	7304	7370	7387	7479	11269	11269
Observations (Level 1)	100	100	100	100	100	100	100	101	101
Akaike Information Criterion	2854.824	2857.651	5075.901	966.734	5023.467	34819.738	22572.587	5492.777	5491.124
Prob. > X^2	<0.001	<0.001	<0.001	<0.001	<0.001	<0.001	<0.001	<0.001	<0.001

North West	Non-polio full immunisation		Delivery			Antenatal care		Child survival	
	Full model	Interaction model (EXPxAGE)	At home	At private facility	At public facility	No. of antenatal care visits	No. of tetanus injections	Exposure decomposition	Total exposure
EXP_CHI	-0.076**	-0.034							
	[-0.151, -0.002]	[-0.110, 0.042]							
EXPxAGE		-0.001*							
		[-0.002, 0.000]							
EXP_PREG			-0.015	-0.059	0.025	0.025	0.002		
			[-0.067, 0.037]	[-0.197, 0.080]	[-0.028, 0.078]	[-0.005, 0.055]	[-0.013, 0.017]		
EXP_PREG_nod (date approximation)								-0.008	
								[-0.051, 0.035]	
EXP_CHI_nod (date approximation)								0.025	
								[-0.011, 0.060]	
EXP_TOT_nod (total exposure, date approximation)									0.013
									[-0.019, 0.045]
CHI_AGE	0.105***	0.065***						-0.047***	-0.043***
	[0.038, 0.172]	[0.017, 0.114]						[-0.072, -0.021]	[-0.068, -0.018]
CHI_AGE2	-0.001**							0.000*	0.000**
	[-0.001, -0.000]							[-0.000, 0.001]	[0.000, 0.001]
CHI_ORD	-0.063*	-0.063*						-0.162***	-0.162***
	[-0.129, 0.002]	[-0.129, 0.002]						[-0.200, -0.124]	[-0.200, -0.124]
CHI_SEX	0.045	0.045						0.126**	0.125**
	[-0.154, 0.245]	[-0.154, 0.245]						[0.010, 0.241]	[0.010, 0.241]
MOT_ANC	0.063***	0.063***	-0.203***	0.221***	0.188***			0.015	0.015
	[0.026, 0.099]	[0.027, 0.099]	[-0.228, -0.178]	[0.147, 0.296]	[0.162, 0.213]			[-0.009, 0.040]	[-0.009, 0.040]
MOT_EDM	0.068***	0.067***	-0.058***	0.011	0.062***	0.127***	0.036***	0.013	0.013
	[0.021, 0.114]	[0.021, 0.114]	[-0.091, -0.025]	[-0.099, 0.120]	[0.028, 0.095]	[0.091, 0.163]	[0.024, 0.049]	[-0.021, 0.048]	[-0.021, 0.048]
MOT_EDF	0.029	0.029	-0.007	-0.043	0.017	0.106***	0.034***	0.043***	0.043***
	[-0.013, 0.071]	[-0.013, 0.071]	[-0.039, 0.024]	[-0.158, 0.071]	[-0.015, 0.048]	[0.083, 0.128]	[0.026, 0.043]	[0.016, 0.070]	[0.015, 0.070]
MOT_AWE	3.547***	3.543***	-0.696***	0.676**	0.688***	1.471***	0.505***		
	[3.265, 3.828]	[3.262, 3.824]	[-0.864, -0.528]	[0.030, 1.323]	[0.517, 0.858]	[1.270, 1.673]	[0.428, 0.582]		
MOT_AGE	0.037***	0.037***	0.007	0.003	-0.009	0.004	-0.002*	0.029***	0.029***
	[0.012, 0.062]	[0.012, 0.062]	[-0.004, 0.019]	[-0.036, 0.043]	[-0.021, 0.003]	[-0.003, 0.011]	[-0.005, 0.000]	[0.014, 0.044]	[0.014, 0.044]
HH_RUR	-0.099	-0.101	0.777***	-0.793**	-0.765***	-0.776***	-0.260***	-0.131	-0.133
	[-0.415, 0.218]	[-0.417, 0.215]	[0.572, 0.982]	[-1.465, -0.120]	[-0.975, -0.555]	[-1.122, -0.429]	[-0.382, -0.139]	[-0.317, 0.055]	[-0.319, 0.054]
HH_REL (ref: Catholic): Other Christian	0.451	0.441	-0.394	-0.061	0.411	0.504	0.005	0.183	0.180
	[-0.256, 1.157]	[-0.266, 1.147]	[-0.948, 0.160]	[-0.966, 0.845]	[-0.133, 0.955]	[-0.148, 1.156]	[-0.186, 0.195]	[-0.565, 0.930]	[-0.567, 0.927]
HH_REL (ref: Catholic): Islam	-0.326	-0.332	0.722**	-1.716***	-0.383	-0.201	-0.190	0.150	0.145
	[-1.138, 0.487]	[-1.144, 0.479]	[0.116, 1.327]	[-2.973, -0.459]	[-0.989, 0.224]	[-0.859, 0.457]	[-0.421, 0.041]	[-0.549, 0.850]	[-0.554, 0.844]
HH_REL (ref: Catholic): Traditionalist	-0.677	-0.669	0.942	0.236	-0.913	-0.536	-0.253**	0.132	0.131
	[-2.263, 0.910]	[-2.251, 0.913]	[-0.244, 2.127]	[-2.038, 2.511]	[-2.224, 0.398]	[-1.263, 0.192]	[-0.496, -0.010]	[-0.787, 1.050]	[-0.787, 1.049]
HH_REL (ref: Catholic): Other	0.000	0.000	0.000	0.000	0.000	1.210	-0.414***	-1.208	-1.195
	[0.000, 0.000]	[0.000, 0.000]	[0.000, 0.000]	[0.000, 0.000]	[0.000, 0.000]	[-0.710, 3.129]	[-0.636, -0.192]	[-3.688, 1.271]	[-3.677, 1.286]
HH_ETH (ref: Ekoi): Fulani	0.087	0.089	0.537**	-0.375	-0.552**	0.389***	0.208***	2.211	2.191
	[-0.590, 0.763]	[-0.587, 0.766]	[0.041, 1.032]	[-1.834, 1.084]	[-1.053, -0.050]	[0.232, 0.546]	[0.138, 0.278]	[-0.597, 5.018]	[-0.615, 4.996]
HH_ETH (ref: Ekoi): Hausa	0.339	0.338	0.474**	-0.792	-0.401**	0.735***	0.333***	1.858	1.841
	[-0.180, 0.859]	[-0.181, 0.857]	[0.096, 0.851]	[-1.816, 0.233]	[-0.782, -0.021]	[0.581, 0.889]	[0.272, 0.394]	[-0.943, 4.659]	[-0.959, 4.641]
HH_ETH (ref: Ekoi): Ibibio	0.000	0.000	0.000	0.000	0.000	0.980***	-0.492	0.000	0.000
	[0.000, 0.000]	[0.000, 0.000]	[0.000, 0.000]	[0.000, 0.000]	[0.000, 0.000]	[0.667, 1.292]	[-2.642, 1.658]	[0.000, 0.000]	[0.000, 0.000]
HH_ETH (ref: Ekoi): Igala	0.000	0.000	0.000	0.000	0.000	3.909***	2.169***	0.000	0.000
	[0.000, 0.000]	[0.000, 0.000]	[0.000, 0.000]	[0.000, 0.000]	[0.000, 0.000]	[3.548, 4.269]	[2.042, 2.296]	[0.000, 0.000]	[0.000, 0.000]
HH_ETH (ref: Ekoi): Igbo	0.216	0.209	-1.134*	-0.479	0.412	1.275**	0.422***	0.000	0.000
	[-0.866, 1.298]	[-0.872, 1.290]	[-2.357, 0.088]	[-1.729, 0.772]	[-0.482, 1.306]	[0.105, 2.444]	[0.130, 0.714]	[0.000, 0.000]	[0.000, 0.000]
HH_ETH (ref: Ekoi): Ijaw / Izon									
HH_ETH (ref: Ekoi): Kanuri / Beriberi	0.626	0.641	1.715**	0.000	-1.428*	1.051***	0.828***	2.261	2.241
	[-0.630, 1.882]	[-0.614, 1.895]	[0.170, 3.259]	[0.000, 0.000]	[-2.963, 0.107]	[0.453, 1.649]	[0.349, 1.307]	[-0.649, 5.170]	[-0.667, 5.149]
HH_ETH (ref: Ekoi): Tiv	0.000	0.000	0.000	1.472	0.197	0.694	-0.069	0.000	0.000
	[0.000, 0.000]	[0.000, 0.000]	[0.000, 0.000]	[-1.265, 4.208]	[-2.515, 2.909]	[-0.510, 1.898]	[-0.478, 0.339]	[0.000, 0.000]	[0.000, 0.000]
HH_ETH (ref: Ekoi): Yoruba	0.609	0.610	-0.492	0.489	0.230	0.606	0.475***	2.118	2.107
	[-0.739, 1.958]	[-0.736, 1.957]	[-1.475, 0.491]	[-0.939, 1.918]	[-0.676, 1.136]	[-0.445, 1.656]	[0.162, 0.787]	[-1.034, 5.270]	[-1.043, 5.258]
	0.000	0.000	0.000	0.000	0.000	0.760***	0.386***	2.275	2.257

HH_ETH (ref: Ekoi): Other	[0.000, 0.000]	[0.000, 0.000]	[0.000, 0.000]	[0.000, 0.000]	[0.000, 0.000]	[0.412, 1.107]	[0.256, 0.516]	[-0.546, 5.096]	[-0.563, 5.076]
HH_SIZ	-0.009	-0.009	0.044***	-0.012	-0.038***	-0.016*	-0.006*	0.122***	0.122***
	[-0.037, 0.020]	[-0.037, 0.020]	[0.022, 0.065]	[-0.081, 0.058]	[-0.060, -0.017]	[-0.033, 0.001]	[-0.013, 0.001]	[0.101, 0.143]	[0.101, 0.143]
HH_WEA	0.133***	0.133***	-0.203***	0.286***	0.170***	0.221***	0.063***	-0.005	-0.005
	[0.080, 0.186]	[0.080, 0.186]	[-0.243, -0.163]	[0.166, 0.405]	[0.130, 0.209]	[0.183, 0.259]	[0.048, 0.079]	[-0.042, 0.031]	[-0.042, 0.031]
YEAR (ref: 2003): 2008	1.387***	1.287***	0.109	-0.886*	0.023	-0.710***	-0.120*	0.276	0.270
	[0.505, 2.270]	[0.402, 2.172]	[-0.313, 0.531]	[-1.808, 0.035]	[-0.417, 0.462]	[-1.208, -0.212]	[-0.257, 0.018]	[-0.075, 0.627]	[-0.081, 0.621]
YEAR (ref: 2003): 2013	1.739***	1.575***	-0.181	-1.844***	0.376*	-0.523*	0.032	0.289	0.309
	[0.753, 2.726]	[0.593, 2.557]	[-0.602, 0.239]	[-2.814, -0.874]	[-0.062, 0.813]	[-1.053, 0.006]	[-0.126, 0.190]	[-0.146, 0.723]	[-0.127, 0.745]
YEAR (ref: 2003): 2018	1.087**	1.170***	-0.450**	0.043	0.476**	-0.116	0.268***	0.272	0.207
	[0.216, 1.959]	[0.304, 2.035]	[-0.872, -0.028]	[-0.846, 0.932]	[0.037, 0.915]	[-0.623, 0.391]	[0.113, 0.424]	[-0.054, 0.599]	[-0.106, 0.520]
Constant	-8.155***	-7.870***	2.044***	-3.822***	-2.524***	1.132**	0.415**	-0.185	-0.229
	[-9.559, -6.752]	[-9.212, -6.529]	[1.300, 2.788]	[-5.669, -1.975]	[-3.287, -1.760]	[0.205, 2.059]	[0.095, 0.736]	[-3.118, 2.747]	[-3.160, 2.701]
Multilevel variance parameter: Level 1	0.708***	0.705***	0.134***	0.290	0.143***	-0.338***	-1.448***	0.025	0.026
	[0.391, 1.025]	[0.389, 1.020]	[0.038, 0.230]	[-0.208, 0.789]	[0.041, 0.244]	[-0.492, -0.183]	[-1.629, -1.267]	[-0.012, 0.062]	[-0.012, 0.063]
Multilevel variance parameter: Residual						0.845***	-0.043*		
						[0.798, 0.892]	[-0.094, 0.007]		
Observations (Level 2)	7249	7249	10429	10383	10432	10447	10663	16552	16552
Observations (Level 1)	166	166	166	166	166	166	166	166	166
Akaike Information Criterion	2869.01	2870.86	5101.199	687.393	5117.543	47639.029	29619.064	8734.342	8734.38
Prob. > X^2	<0.001	<0.001	<0.001	<0.001	<0.001	<0.001	<0.001	<0.001	<0.001

South East	Non-polio full immunisation		Delivery			Antenatal care		Child survival	
	**Full model**	**Interaction model (EXPxAGE)**	**At home**	**At private facility**	**At public facility**	**No. of antenatal care visits**	**No. of tetanus injections**	**Exposure decomposition**	**Total exposure**
EXP_CHI	0.092<br>[-0.035, 0.220]	0.238**<br>[0.056, 0.420]							
EXPxAGE		-0.005***<br>[-0.008, -0.002]							
EXP_PREG			0.036<br>[-0.062, 0.135]	0.020<br>[-0.060, 0.099]	-0.049<br>[-0.129, 0.030]	0.155**<br>[0.001, 0.308]	0.010<br>[-0.021, 0.042]		
EXP_PREG_nod (date approximation)								0.036<br>[-0.116, 0.189]	
EXP_CHI_nod (date approximation)								-0.043<br>[-0.171, 0.085]	
EXP_TOT_nod (total exposure, date approximation)									-0.013<br>[-0.126, 0.099]
CHI_AGE	0.112***<br>[0.069, 0.155]	0.048***<br>[0.019, 0.078]						-0.017<br>[-0.056, 0.022]	-0.013<br>[-0.052, 0.025]
CHI_AGE2	-0.002***<br>[-0.002, -0.001]							0.000<br>[-0.000, 0.001]	0.000<br>[-0.000, 0.001]
CHI_ORD	-0.037<br>[-0.100, 0.026]	-0.036<br>[-0.099, 0.027]						-0.122***<br>[-0.195, -0.048]	-0.122***<br>[-0.195, -0.048]
CHI_SEX	-0.149<br>[-0.362, 0.063]	-0.148<br>[-0.360, 0.065]						0.386***<br>[0.127, 0.645]	0.383***<br>[0.124, 0.642]
MOT_ANC	0.030***<br>[0.007, 0.053]	0.030***<br>[0.007, 0.053]	-0.104***<br>[-0.131, -0.077]	0.037***<br>[0.020, 0.055]	0.021**<br>[0.003, 0.038]			-0.008<br>[-0.033, 0.017]	-0.007<br>[-0.032, 0.018]
MOT_EDM	0.055*<br>[-0.004, 0.115]	0.055*<br>[-0.004, 0.114]	-0.075**<br>[-0.132, -0.018]	-0.027<br>[-0.075, 0.021]	0.099***<br>[0.049, 0.148]	0.056<br>[-0.061, 0.173]	0.040***<br>[0.020, 0.061]	-0.001<br>[-0.071, 0.069]	-0.001<br>[-0.071, 0.069]
MOT_EDF	0.016<br>[-0.046, 0.079]	0.016<br>[-0.047, 0.078]	-0.106***<br>[-0.164, -0.048]	0.055**<br>[0.003, 0.106]	0.036<br>[-0.016, 0.088]	0.008<br>[-0.095, 0.111]	-0.004<br>[-0.032, 0.024]	0.024<br>[-0.046, 0.095]	0.026<br>[-0.045, 0.096]
MOT_AWE	2.623***<br>[2.236, 3.011]	2.613***<br>[2.226, 3.000]	-0.974***<br>[-1.244, -0.703]	0.199<br>[-0.055, 0.452]	0.802***<br>[0.520, 1.083]	1.372***<br>[0.702, 2.042]	0.448***<br>[0.274, 0.621]		
MOT_AGE	0.013<br>[-0.010, 0.036]	0.013<br>[-0.011, 0.036]	-0.020**<br>[-0.037, -0.002]	-0.001<br>[-0.015, 0.013]	0.012*<br>[-0.002, 0.026]	-0.001<br>[-0.024, 0.022]	0.002<br>[-0.003, 0.007]	-0.019<br>[-0.044, 0.007]	-0.019<br>[-0.044, 0.007]
HH_RUR	-0.094<br>[-0.343, 0.155]	-0.102<br>[-0.351, 0.147]	-0.003<br>[-0.271, 0.264]	-0.129<br>[-0.333, 0.075]	0.181*<br>[-0.020, 0.382]	-0.018<br>[-0.493, 0.457]	-0.060<br>[-0.141, 0.020]	-0.306**<br>[-0.571, -0.041]	-0.305**<br>[-0.570, -0.041]
HH_REL (ref: Catholic): Other Christian	-0.110<br>[-0.338, 0.118]	-0.103<br>[-0.330, 0.125]	0.016<br>[-0.221, 0.254]	-0.189**<br>[-0.366, -0.012]	0.206**<br>[0.028, 0.384]	-0.235<br>[-0.572, 0.103]	-0.099**<br>[-0.175, -0.023]	-0.316**<br>[-0.590, -0.041]	-0.312**<br>[-0.587, -0.038]
HH_REL (ref: Catholic): Islam	-0.055<br>[-2.549, 2.440]	-0.031<br>[-2.503, 2.442]	-0.726<br>[-3.253, 1.801]	1.069<br>[-0.661, 2.798]	-0.588<br>[-2.638, 1.463]	0.705<br>[-2.529, 3.938]	-0.146<br>[-0.858, 0.567]	0.000<br>[0.000, 0.000]	0.000<br>[0.000, 0.000]
HH_REL (ref: Catholic): Traditionalist	-0.235<br>[-0.870, 0.400]	-0.223<br>[-0.859, 0.412]	1.728***<br>[1.049, 2.407]	-1.829***<br>[-2.694, -0.964]	-0.951**<br>[-1.693, -0.209]	-1.223**<br>[-2.193, -0.254]	-0.326**<br>[-0.646, -0.005]	-0.303<br>[-1.031, 0.424]	-0.299<br>[-1.027, 0.429]
HH_REL (ref: Catholic): Other	0.000<br>[0.000, 0.000]	0.000<br>[0.000, 0.000]	0.000<br>[0.000, 0.000]	0.000<br>[0.000, 0.000]	0.000<br>[0.000, 0.000]	16.125***<br>[15.152, 17.099]	1.493***<br>[1.304, 1.683]	-1.997<br>[-4.770, 0.776]	-2.025<br>[-4.787, 0.737]
HH_ETH (ref: Ekoi): Fulani	0.000<br>[0.000, 0.000]	0.000<br>[0.000, 0.000]	0.000<br>[0.000, 0.000]	0.000<br>[0.000, 0.000]	0.000<br>[0.000, 0.000]	0.000<br>[0.000, 0.000]	0.000<br>[0.000, 0.000]	0.000<br>[0.000, 0.000]	0.000<br>[0.000, 0.000]
HH_ETH (ref: Ekoi): Hausa	-1.150<br>[-2.979, 0.679]	-1.069<br>[-2.890, 0.753]	-0.604<br>[-3.250, 2.042]	0.283<br>[-1.011, 1.577]	-0.489<br>[-1.841, 0.863]	3.793***<br>[2.817, 4.769]	0.286*<br>[-0.006, 0.578]	0.370<br>[-1.248, 1.989]	0.389<br>[-1.227, 2.006]
HH_ETH (ref: Ekoi): Ibibio	-0.356<br>[-2.952, 2.240]	-0.310<br>[-2.905, 2.285]	0.975<br>[-1.143, 3.094]	-1.174<br>[-3.019, 0.671]	0.507<br>[-1.176, 2.190]	0.393<br>[-1.936, 2.722]	0.100<br>[-0.733, 0.933]	0.296<br>[-2.029, 2.622]	0.303<br>[-2.023, 2.629]
HH_ETH (ref: Ekoi): Igala	-0.604<br>[-3.173, 1.964]	-0.637<br>[-3.211, 1.936]	-0.394<br>[-2.689, 1.901]	-2.032<br>[-4.466, 0.402]	1.572*<br>[-0.222, 3.366]	3.192***<br>[1.463, 4.921]	0.983<br>[-0.706, 2.673]	0.282<br>[-1.560, 2.124]	0.279<br>[-1.562, 2.121]
HH_ETH (ref: Ekoi): Igbo	-0.887<br>[-2.193, 0.419]	-0.856<br>[-2.161, 0.449]	-0.040<br>[-1.256, 1.177]	-0.183<br>[-1.007, 0.640]	0.120<br>[-0.754, 0.994]	3.875***<br>[3.160, 4.590]	0.400***<br>[0.282, 0.518]	0.828<br>[-0.257, 1.914]	0.839<br>[-0.246, 1.924]
HH_ETH (ref: Ekoi): Ijaw / Izon	0.000<br>[0.000, 0.000]	0.000<br>[0.000, 0.000]	0.000<br>[0.000, 0.000]	0.000<br>[0.000, 0.000]	0.000<br>[0.000, 0.000]	-0.404<br>[-1.516, 0.707]	-1.627***<br>[-1.814, -1.440]	0.000<br>[0.000, 0.000]	0.000<br>[0.000, 0.000]
HH_ETH (ref: Ekoi): Kanuri / Beriberi									
HH_ETH (ref: Ekoi): Tiv	0.000<br>[0.000, 0.000]	0.000<br>[0.000, 0.000]	0.000<br>[0.000, 0.000]	0.000<br>[0.000, 0.000]	0.000<br>[0.000, 0.000]	-3.047***<br>[-3.747, -2.348]	-1.582***<br>[-1.708, -1.456]	0.000<br>[0.000, 0.000]	0.000<br>[0.000, 0.000]
HH_ETH (ref: Ekoi): Yoruba	0.000<br>[0.000, 0.000]	0.000<br>[0.000, 0.000]	0.220<br>[-2.478, 2.919]	-1.742*<br>[-3.529, 0.045]	1.350<br>[-0.360, 3.060]	2.502**<br>[0.025, 4.978]	0.491<br>[-0.231, 1.212]	0.000<br>[0.000, 0.000]	0.000<br>[0.000, 0.000]
HH_ETH (ref: Ekoi): Other	0.000	0.000	0.000	0.000	0.000	4.086***	0.676***	0.000	0.000

HH_ETH (ref: Ekol): Other	[0.000, 0.000]	[0.000, 0.000]	[0.000, 0.000]	[0.000, 0.000]	[0.000, 0.000]	[1.888, 6.284]	[0.389, 0.964]	[0.000, 0.000]	[0.000, 0.000]
HH_SIZ	-0.012	-0.013	0.023	-0.002	-0.012	-0.101***	-0.006	0.206***	0.206***
	[-0.052, 0.028]	[-0.053, 0.027]	[-0.017, 0.064]	[-0.035, 0.030]	[-0.045, 0.021]	[-0.166, -0.037]	[-0.018, 0.007]	[0.132, 0.280]	[0.132, 0.280]
HH_WEA	0.165***	0.164***	-0.214***	0.152***	-0.016	0.297***	0.046***	0.034	0.034
	[0.114, 0.217]	[0.113, 0.215]	[-0.268, -0.161]	[0.111, 0.192]	[-0.055, 0.024]	[0.201, 0.393]	[0.029, 0.064]	[-0.025, 0.093]	[-0.025, 0.093]
YEAR (ref: 2003): 2008	0.876***	0.878***	-0.072	0.063	-0.165	-0.647	-0.070	0.265	0.227
	[0.211, 1.541]	[0.214, 1.542]	[-0.638, 0.494]	[-0.404, 0.530]	[-0.641, 0.312]	[-1.778, 0.484]	[-0.286, 0.147]	[-0.444, 0.973]	[-0.477, 0.930]
YEAR (ref: 2003): 2013	0.906***	0.914***	-0.075	-0.150	0.092	0.659	0.113	0.234	0.166
	[0.250, 1.563]	[0.258, 1.571]	[-0.629, 0.479]	[-0.602, 0.302]	[-0.367, 0.551]	[-0.404, 1.722]	[-0.119, 0.345]	[-0.475, 0.943]	[-0.529, 0.861]
YEAR (ref: 2003): 2018	1.159***	1.301***	-0.147	-0.237	0.217	-0.653	0.027	0.468	0.462
	[0.408, 1.910]	[0.534, 2.067]	[-0.738, 0.444]	[-0.718, 0.244]	[-0.268, 0.702]	[-1.764, 0.458]	[-0.217, 0.270]	[-0.359, 1.295]	[-0.365, 1.289]
Constant	-4.957***	-4.578***	2.123***	-1.095**	-2.661***	2.038**	1.009***	1.657**	1.685**
	[-6.650, -3.263]	[-6.238, -2.917]	[0.628, 3.618]	[-2.177, -0.013]	[-3.796, -1.526]	[0.300, 3.777]	[0.663, 1.355]	[0.114, 3.199]	[0.144, 3.226]
Multilevel variance parameter: Level 1	0.169**	0.169**	0.750***	0.355***	0.238***	0.613***	-1.893***	0.000	0.000
	[0.034, 0.304]	[0.033, 0.304]	[0.346, 1.154]	[0.183, 0.528]	[0.105, 0.371]	[0.435, 0.791]	[-2.192, -1.594]	[-0.000, 0.000]	[-0.000, 0.000]
Multilevel variance parameter: Residual						1.510***	-0.008		
						[1.439, 1.582]	[-0.073, 0.056]		
Observations (Level 2)	2036	2036	2900	2900	2900	2949	3229	4362	4362
Observations (Level 1)	85	85	86	86	123	86	87	87	87
Akaike Information Criterion	2198.177	2202.549	2361.019	3572.969	3513.076	17468.034	9205.156	1926.233	1925.154
Prob. > X^2	<0.001	<0.001	<0.001	<0.001	<0.001	<0.001	<0.001	<0.001	<0.001

South South	Non-polio full immunisation		Delivery			Antenatal care		Child survival	
	Full model	Interaction model (EXPxAGE)	At home	At private facility	At public facility	No. of antenatal care visits	No. of tetanus injections	Exposure decomposition	Total exposure
EXP_CHI	0.065	0.171**							
	[-0.042, 0.171]	[0.001, 0.341]							
EXPxAGE		-0.003***							
		[-0.006, -0.001]							
EXP_PREG			-0.023	0.038	0.014	0.072	0.037*		
			[-0.102, 0.056]	[-0.074, 0.150]	[-0.060, 0.088]	[-0.055, 0.198]	[-0.000, 0.074]		
EXP_PREG_nod (date approximation)								-0.056	
								[-0.206, 0.094]	
EXP_CHI_nod (date approximation)								-0.005	
								[-0.139, 0.128]	
EXP_TOT_nod (total exposure, date approximation)									-0.026
									[-0.142, 0.091]
CHI_AGE	0.073***	0.028**						0.023	0.021
	[0.035, 0.110]	[0.003, 0.053]						[-0.015, 0.061]	[-0.016, 0.059]
CHI_AGE2	-0.001***							0.000	0.000
	[-0.002, -0.000]							[-0.001, 0.000]	[-0.001, 0.000]
CHI_ORD	-0.073**	-0.074**						-0.239***	-0.239***
	[-0.135, -0.010]	[-0.137, -0.011]						[-0.318, -0.160]	[-0.318, -0.160]
CHI_SEX	0.176*	0.173*						0.317**	0.317**
	[-0.016, 0.368]	[-0.019, 0.365]						[0.054, 0.580]	[0.054, 0.580]
MOT_ANC	0.039***	0.039***	-0.143***	0.072***	0.083***			0.017	0.017
	[0.019, 0.060]	[0.019, 0.060]	[-0.162, -0.124]	[0.050, 0.093]	[0.067, 0.100]			[-0.011, 0.046]	[-0.011, 0.046]
MOT_EDM	0.050*	0.050*	-0.023	0.028	0.015	0.075**	0.031***	0.033	0.033
	[-0.000, 0.101]	[-0.001, 0.101]	[-0.069, 0.023]	[-0.038, 0.094]	[-0.028, 0.058]	[0.005, 0.146]	[0.010, 0.051]	[-0.032, 0.097]	[-0.032, 0.097]
MOT_EDF	0.010	0.010	-0.005	0.029	-0.007	0.094*	-0.011	-0.036	-0.036
	[-0.046, 0.066]	[-0.046, 0.065]	[-0.055, 0.044]	[-0.044, 0.101]	[-0.054, 0.040]	[-0.000, 0.188]	[-0.032, 0.010]	[-0.113, 0.040]	[-0.113, 0.040]
MOT_AWE	2.921***	2.908***	-1.262***	0.825***	1.080***	2.596***	0.657***		
	[2.577, 3.265]	[2.564, 3.251]	[-1.510, -1.015]	[0.411, 1.239]	[0.830, 1.330]	[2.197, 2.995]	[0.539, 0.775]		
MOT_AGE	0.026**	0.026**	-0.030***	0.007	0.023***	0.019	0.001	-0.003	-0.003
	[0.005, 0.047]	[0.005, 0.048]	[-0.044, -0.016]	[-0.013, 0.027]	[0.010, 0.036]	[-0.006, 0.045]	[-0.005, 0.007]	[-0.031, 0.025]	[-0.031, 0.025]
HH_RUR	-0.040	-0.047	0.715***	-0.564***	-0.322***	-1.067***	-0.065	-0.405**	-0.405**
	[-0.280, 0.200]	[-0.288, 0.193]	[0.476, 0.953]	[-0.874, -0.253]	[-0.543, -0.100]	[-1.594, -0.539]	[-0.192, 0.062]	[-0.762, -0.048]	[-0.762, -0.049]
HH_REL (ref: Catholic): Other Christian	-0.391**	-0.396**	0.312**	0.237	-0.446***	-0.375	-0.106*	0.054	0.054
	[-0.741, -0.042]	[-0.746, -0.046]	[0.027, 0.598]	[-0.166, 0.641]	[-0.710, -0.182]	[-1.003, 0.253]	[-0.226, 0.015]	[-0.403, 0.510]	[-0.402, 0.510]
HH_REL (ref: Catholic): Islam	0.009	0.006	-0.084	0.063	0.157	0.019	0.169	0.786	0.787
	[-0.761, 0.779]	[-0.765, 0.776]	[-0.771, 0.603]	[-0.702, 0.829]	[-0.457, 0.771]	[-1.728, 1.765]	[-0.139, 0.477]	[-0.598, 2.169]	[-0.597, 2.171]
HH_REL (ref: Catholic): Traditionalist	-0.746	-0.727	0.634	-0.113	-0.469	0.748	-0.339	-0.562	-0.557
	[-1.750, 0.258]	[-1.732, 0.277]	[-0.311, 1.579]	[-1.255, 1.030]	[-1.335, 0.397]	[-0.852, 2.347]	[-0.789, 0.112]	[-1.539, 0.415]	[-1.534, 0.420]
HH_REL (ref: Catholic): Other	-0.300	-0.294	-0.562	0.000	0.518	-0.793	-0.237	0.000	0.000
	[-1.524, 0.924]	[-1.520, 0.932]	[-1.606, 0.482]	[0.000, 0.000]	[-0.484, 1.519]	[-2.518, 0.933]	[-1.005, 0.532]	[0.000, 0.000]	[0.000, 0.000]
HH_ETH (ref: Ekoi): Fulani									
HH_ETH (ref: Ekoi): Hausa	-1.438	-1.440	0.033	0.177	0.135	1.450	-0.187	-1.042	-1.025
	[-3.691, 0.815]	[-3.692, 0.813]	[-1.779, 1.845]	[-2.288, 2.643]	[-1.520, 1.790]	[-2.747, 5.646]	[-0.686, 0.312]	[-3.402, 1.317]	[-3.384, 1.334]
HH_ETH (ref: Ekoi): Ibibio	-0.923***	-0.920***	0.244	0.183	-0.129	0.598	-0.003	-0.496	-0.494
	[-1.445, -0.401]	[-1.444, -0.397]	[-0.267, 0.754]	[-0.805, 1.171]	[-0.625, 0.367]	[-0.506, 1.701]	[-0.274, 0.267]	[-1.258, 0.265]	[-1.255, 0.267]
HH_ETH (ref: Ekoi): Igala	-0.758	-0.740	0.050	1.096	-0.256	0.828	-0.337	-0.964	-0.963
	[-3.413, 1.898]	[-3.351, 1.871]	[-1.849, 1.949]	[-1.306, 3.499]	[-1.947, 1.436]	[-1.132, 2.787]	[-1.153, 0.479]	[-3.153, 1.225]	[-3.151, 1.224]
HH_ETH (ref: Ekoi): Igbo	-0.492*	-0.488*	-0.025	1.214***	-0.488**	1.838***	0.088	-0.304	-0.300
	[-1.051, 0.067]	[-1.049, 0.073]	[-0.539, 0.488]	[0.297, 2.130]	[-0.975, -0.000]	[0.562, 3.114]	[-0.165, 0.342]	[-1.113, 0.506]	[-1.109, 0.509]
HH_ETH (ref: Ekoi): Ijaw / Izon	-0.640**	-0.639**	0.804***	-0.043	-0.679***	0.095	-0.018	-0.213	-0.212
	[-1.138, -0.143]	[-1.140, -0.139]	[0.290, 1.317]	[-1.023, 0.938]	[-1.178, -0.180]	[-1.029, 1.219]	[-0.265, 0.229]	[-0.951, 0.526]	[-0.950, 0.526]
HH_ETH (ref: Ekoi): Kanuri / Beriberi	0.000	0.000	0.000	0.000	0.000			0.000	0.000
	[0.000, 0.000]	[0.000, 0.000]	[0.000, 0.000]	[0.000, 0.000]	[0.000, 0.000]			[0.000, 0.000]	[0.000, 0.000]
HH_ETH (ref: Ekoi): Tiv	0.000	0.000	0.000	0.000	0.000	1.582**	-1.433***	0.000	0.000
	[0.000, 0.000]	[0.000, 0.000]	[0.000, 0.000]	[0.000, 0.000]	[0.000, 0.000]	[0.257, 2.906]	[-1.688, -1.177]	[0.000, 0.000]	[0.000, 0.000]
HH_ETH (ref: Ekoi): Yoruba	-2.193***	-2.185***	-0.089	2.236***	-1.470***	1.253	0.005	0.131	0.137
	[-3.155, -1.231]	[-3.148, -1.222]	[-0.970, 0.791]	[1.101, 3.371]	[-2.336, -0.604]	[-0.524, 3.030]	[-0.370, 0.380]	[-1.496, 1.757]	[-1.491, 1.765]
	-0.887***	-0.885***	0.126	0.953**	-0.384*	1.037*	0.063	-0.202	-0.201

HH_ETH (ref: Ekoi): Other	[-1.352, -0.423]	[-1.352, -0.419]	[-0.319, 0.571]	[0.066, 1.840]	[-0.814, 0.046]	[-0.022, 2.095]	[-0.169, 0.294]	[-0.905, 0.500]	[-0.903, 0.501]
HH_SIZ	-0.016	-0.017	0.058***	-0.043	-0.030*	-0.002	-0.016*	0.314***	0.315***
	[-0.060, 0.027]	[-0.060, 0.027]	[0.020, 0.095]	[-0.095, 0.010]	[-0.065, 0.005]	[-0.067, 0.064]	[-0.033, 0.002]	[0.236, 0.393]	[0.236, 0.394]
HH_WEA	0.120***	0.119***	-0.180***	0.139***	0.103***	0.409***	0.066***	-0.041	-0.041
	[0.074, 0.167]	[0.072, 0.166]	[-0.223, -0.137]	[0.079, 0.199]	[0.063, 0.144]	[0.316, 0.502]	[0.043, 0.088]	[-0.105, 0.022]	[-0.104, 0.023]
YEAR (ref: 2003): 2008	0.898**	0.906**	0.499*	-0.197	-0.311	-0.350	-0.306***	1.135***	1.150***
	[0.146, 1.650]	[0.153, 1.658]	[-0.031, 1.029]	[-0.819, 0.425]	[-0.800, 0.179]	[-1.507, 0.807]	[-0.519, -0.093]	[0.505, 1.765]	[0.522, 1.779]
YEAR (ref: 2003): 2013	1.469***	1.461***	0.489*	-0.851***	-0.036	-1.238**	-0.284**	1.092***	1.138***
	[0.726, 2.212]	[0.716, 2.207]	[-0.034, 1.013]	[-1.468, -0.234]	[-0.517, 0.446]	[-2.391, -0.084]	[-0.503, -0.064]	[0.480, 1.704]	[0.543, 1.733]
YEAR (ref: 2003): 2018	1.745***	1.874***	0.674**	-0.759**	-0.153	-1.744***	-0.372***	1.288***	1.287***
	[0.917, 2.572]	[1.022, 2.726]	[0.117, 1.231]	[-1.427, -0.091]	[-0.669, 0.363]	[-2.894, -0.594]	[-0.601, -0.144]	[0.519, 2.058]	[0.518, 2.056]
Constant	-5.197***	-4.898***	2.076***	-4.455***	-2.101***	2.123**	1.270***	1.540**	1.500**
	[-6.400, -3.994]	[-6.061, -3.736]	[1.189, 2.964]	[-5.818, -3.092]	[-2.943, -1.259]	[0.111, 4.135]	[0.854, 1.686]	[0.273, 2.808]	[0.238, 2.761]
Multilevel variance parameter: Level 1	0.024	0.027	0.389***	0.640***	0.365***	0.216**	-1.507***	0.057	0.057
	[-0.040, 0.087]	[-0.038, 0.092]	[0.190, 0.587]	[0.282, 0.997]	[0.193, 0.538]	[0.007, 0.426]	[-1.738, -1.276]	[-0.072, 0.187]	[-0.072, 0.186]
Multilevel variance parameter: Residual						1.554***	0.190***		
						[1.495, 1.614]	[0.136, 0.244]		
Observations (Level 2)	2514	2514	3552	3528	3552	3620	3975	4979	4979
Observations (Level 1)	111	111	112	112	112	112	112	113	113
Akaike Information Criterion	2613.206	2616.454	3449.379	2094.164	3846.725	21688.628	12916.658	1912.315	1910.708
Prob. > X^2	<0.001	<0.001	<0.001	<0.001	<0.001	<0.001	<0.001	<0.001	<0.001

South West	Non-polio full immunisation		Delivery			Antenatal care		Child survival	
	**Full model**	**Interaction model (EXPxAGE)**	**At home**	**At private facility**	**At public facility**	**No. of antenatal care visits**	**No. of tetanus injections**	**Exposure decomposition**	**Total exposure**
EXP_CHI	-0.060	-0.019							
	[-0.144, 0.024]	[-0.138, 0.101]							
EXPxAGE		-0.002*							
		[-0.004, 0.000]							
EXP_PREG			0.055	-0.003	-0.033	-0.069	0.034***		
			[-0.013, 0.122]	[-0.059, 0.052]	[-0.087, 0.022]	[-0.206, 0.069]	[0.009, 0.058]		
EXP_PREG_nod (date approximation)								-0.021	
								[-0.145, 0.102]	
EXP_CHI_nod (date approximation)								0.018	
								[-0.103, 0.139]	
EXP_TOT_nod (total exposure, date approximation)									-0.001
									[-0.103, 0.101]
CHI_AGE	0.083***	0.056***						0.035*	0.035*
	[0.050, 0.117]	[0.035, 0.077]						[-0.001, 0.071]	[-0.001, 0.071]
CHI_AGE2	-0.001**							0.000	0.000
	[-0.001, -0.000]							[-0.001, 0.000]	[-0.001, 0.000]
CHI_ORD	-0.087**	-0.087**						-0.369***	-0.367***
	[-0.156, -0.017]	[-0.157, -0.018]						[-0.468, -0.271]	[-0.466, -0.269]
CHI_SEX	-0.006	-0.002						0.213	0.212
	[-0.182, 0.171]	[-0.179, 0.174]						[-0.061, 0.486]	[-0.061, 0.486]
MOT_ANC	0.039***	0.039***	-0.064***	0.034***	0.011*			-0.023**	-0.022**
	[0.024, 0.054]	[0.024, 0.054]	[-0.078, -0.049]	[0.023, 0.045]	[-0.000, 0.022]			[-0.045, -0.001]	[-0.044, -0.000]
MOT_EDM	0.042*	0.042*	-0.040*	0.054***	-0.013	0.068	0.002	0.012	0.012
	[-0.004, 0.088]	[-0.004, 0.088]	[-0.082, 0.003]	[0.016, 0.091]	[-0.049, 0.023]	[-0.024, 0.160]	[-0.015, 0.019]	[-0.058, 0.082]	[-0.058, 0.082]
MOT_EDF	-0.031	-0.031	-0.015	-0.001	0.018	0.034	0.008	0.003	0.002
	[-0.080, 0.018]	[-0.080, 0.018]	[-0.059, 0.029]	[-0.041, 0.038]	[-0.019, 0.055]	[-0.077, 0.146]	[-0.012, 0.028]	[-0.070, 0.075]	[-0.070, 0.074]
MOT_AWE	2.962***	2.953***	-1.082***	0.072	0.973***	2.383***	0.582***		
	[2.589, 3.336]	[2.580, 3.327]	[-1.294, -0.870]	[-0.142, 0.286]	[0.756, 1.190]	[1.878, 2.889]	[0.488, 0.675]		
MOT_AGE	0.031***	0.031***	-0.006	-0.005	0.011*	0.012	0.002	-0.012	-0.012
	[0.011, 0.051]	[0.011, 0.051]	[-0.020, 0.008]	[-0.018, 0.007]	[-0.001, 0.023]	[-0.016, 0.041]	[-0.003, 0.006]	[-0.041, 0.018]	[-0.042, 0.017]
HH_RUR	-0.242*	-0.243*	0.028	-0.455***	0.333***	0.423	-0.006	0.038	0.044
	[-0.489, 0.006]	[-0.490, 0.005]	[-0.217, 0.273]	[-0.668, -0.242]	[0.129, 0.537]	[-0.286, 1.133]	[-0.098, 0.086]	[-0.304, 0.380]	[-0.297, 0.385]
HH_REL (ref: Catholic): Other Christian	-0.095	-0.098	-0.232	0.171	-0.032	0.380	0.053	-0.180	-0.180
	[-0.593, 0.403]	[-0.596, 0.400]	[-0.672, 0.207]	[-0.186, 0.529]	[-0.385, 0.321]	[-0.733, 1.493]	[-0.078, 0.185]	[-0.859, 0.500]	[-0.859, 0.499]
HH_REL (ref: Catholic): Islam	-0.402	-0.402	-0.261	0.213	-0.042	0.431	0.098	0.080	0.082
	[-0.919, 0.115]	[-0.918, 0.114]	[-0.718, 0.195]	[-0.164, 0.589]	[-0.412, 0.328]	[-0.780, 1.642]	[-0.040, 0.235]	[-0.642, 0.803]	[-0.640, 0.804]
HH_REL (ref: Catholic): Traditionalist	-0.218	-0.228	0.404	-0.846	0.350	1.704	-0.393*	0.124	0.112
	[-1.501, 1.066]	[-1.508, 1.051]	[-0.674, 1.481]	[-2.038, 0.345]	[-0.641, 1.341]	[-3.022, 6.429]	[-0.803, 0.017]	[-2.049, 2.298]	[-2.061, 2.285]
HH_REL (ref: Catholic): Other	0.994	0.992	0.337	0.000	1.628	0.461	1.825	0.000	0.000
	[-3.233, 5.221]	[-3.182, 5.167]	[-2.751, 3.425]	[0.000, 0.000]	[-1.684, 4.939]	[-12.702, 13.625]	[-2.055, 5.705]	[0.000, 0.000]	[0.000, 0.000]
HH_ETH (ref: Ekoi): Fulani	-0.720	-0.713	1.513***	-0.523	-2.137***	0.000	0.000	0.993	1.003
	[-1.789, 0.349]	[-1.784, 0.359]	[0.758, 2.268]	[-1.340, 0.294]	[-3.144, -1.131]	[0.000, 0.000]	[0.000, 0.000]	[-1.064, 3.050]	[-1.053, 3.060]
HH_ETH (ref: Ekoi): Hausa	-0.224	-0.227	0.613*	-0.986***	0.418	2.791**	0.594**	0.383	0.381
	[-1.123, 0.675]	[-1.125, 0.671]	[-0.096, 1.322]	[-1.735, -0.237]	[-0.268, 1.105]	[0.122, 5.461]	[0.121, 1.067]	[-0.869, 1.634]	[-0.869, 1.631]
HH_ETH (ref: Ekoi): Ibibio	1.109	1.115	-0.264	0.026	0.257	3.338**	0.499**	-0.060	-0.068
	[-0.248, 2.466]	[-0.237, 2.468]	[-1.248, 0.720]	[-0.791, 0.844]	[-0.618, 1.132]	[0.715, 5.961]	[0.045, 0.952]	[-1.552, 1.433]	[-1.560, 1.423]
HH_ETH (ref: Ekoi): Igala	0.685	0.694	-0.676	0.647	-0.081	3.470***	0.776***	0.000	0.000
	[-0.655, 2.024]	[-0.645, 2.032]	[-1.738, 0.385]	[-0.253, 1.547]	[-1.019, 0.857]	[1.030, 5.910]	[0.247, 1.305]	[0.000, 0.000]	[0.000, 0.000]
HH_ETH (ref: Ekoi): Igbo	0.521**	0.531**	-1.501***	0.786***	0.009	4.918***	0.908***	-0.024	-0.020
	[0.014, 1.028]	[0.025, 1.038]	[-2.066, -0.936]	[0.422, 1.150]	[-0.366, 0.384]	[2.881, 6.955]	[0.502, 1.315]	[-0.650, 0.603]	[-0.647, 0.606]
HH_ETH (ref: Ekoi): Ijaw / Izon	-0.625	-0.638	0.804	-0.273	-1.925**	2.087*	0.107	0.497	0.505
	[-2.141, 0.891]	[-2.160, 0.884]	[-0.205, 1.812]	[-1.215, 0.668]	[-3.431, -0.419]	[-0.170, 4.344]	[-0.322, 0.536]	[-0.809, 1.804]	[-0.801, 1.811]
HH_ETH (ref: Ekoi): Kanuri / Beriberi	2.769*	2.780*	1.245	0.000	1.406	2.783	0.080	0.000	0.000
	[-0.166, 5.704]	[-0.149, 5.710]	[-1.500, 3.990]	[0.000, 0.000]	[-1.171, 3.984]	[-2.223, 7.790]	[-1.800, 1.960]	[0.000, 0.000]	[0.000, 0.000]
HH_ETH (ref: Ekoi): Tiv	1.769*	1.775*	-0.566	-0.238	0.530	1.717	0.661*	-0.015	-0.011
	[-0.032, 3.569]	[-0.025, 3.574]	[-1.650, 0.518]	[-1.545, 1.068]	[-0.532, 1.592]	[-1.581, 5.014]	[-0.123, 1.445]	[-2.100, 2.070]	[-2.096, 2.074]
HH_ETH (ref: Ekoi): Yoruba	0.289*	0.294*	-0.244*	-0.118	0.341***	5.612***	0.944***	0.438**	0.440**
	[-0.008, 0.587]	[-0.004, 0.591]	[-0.505, 0.016]	[-0.361, 0.126]	[0.102, 0.580]	[3.766, 7.457]	[0.562, 1.325]	[0.030, 0.846]	[0.032, 0.848]

HH_ETH (ref: Ekoi): Other	0.000	0.000	0.000	0.000	0.000	4.747***	0.845***	0.000	0.000
	[0.000, 0.000]	[0.000, 0.000]	[0.000, 0.000]	[0.000, 0.000]	[0.000, 0.000]	[2.737, 6.757]	[0.439, 1.250]	[0.000, 0.000]	[0.000, 0.000]
HH_SIZ	-0.011	-0.011	0.031	-0.020	-0.008	-0.136***	0.002	0.499***	0.498***
	[-0.058, 0.036]	[-0.059, 0.036]	[-0.010, 0.071]	[-0.056, 0.016]	[-0.042, 0.026]	[-0.238, -0.035]	[-0.014, 0.018]	[0.399, 0.599]	[0.398, 0.598]
HH_WEA	0.142***	0.142***	-0.154***	0.066***	0.059***	0.298***	0.040***	-0.012	-0.011
	[0.090, 0.195]	[0.089, 0.195]	[-0.203, -0.105]	[0.024, 0.108]	[0.019, 0.099]	[0.193, 0.403]	[0.022, 0.058]	[-0.088, 0.064]	[-0.087, 0.064]
YEAR (ref: 2003): 2008	-0.257	-0.252	-0.027	-0.356*	0.338	-1.254**	-0.181**	-0.084	-0.065
	[-0.891, 0.378]	[-0.889, 0.385]	[-0.516, 0.461]	[-0.754, 0.041]	[-0.086, 0.763]	[-2.220, -0.288]	[-0.337, -0.024]	[-0.845, 0.677]	[-0.823, 0.694]
YEAR (ref: 2003): 2013	-0.749**	-0.726**	0.236	-0.495**	0.258	-0.078	-0.261***	0.140	0.174
	[-1.390, -0.108]	[-1.368, -0.084]	[-0.256, 0.728]	[-0.891, -0.100]	[-0.165, 0.681]	[-1.055, 0.898]	[-0.415, -0.107]	[-0.628, 0.908]	[-0.584, 0.933]
YEAR (ref: 2003): 2018	-0.694**	-0.656*	-0.072	-0.716***	0.709***	-3.705***	-0.425***	0.159	0.149
	[-1.384, -0.003]	[-1.349, 0.037]	[-0.595, 0.452]	[-1.140, -0.292]	[0.265, 1.153]	[-4.735, -2.674]	[-0.602, -0.247]	[-0.675, 0.993]	[-0.684, 0.982]
Constant	-4.520***	-4.319***	1.438***	-1.088***	-2.422***	3.280***	0.345	1.871***	1.849***
	[-5.602, -3.439]	[-5.373, -3.265]	[0.624, 2.252]	[-1.786, -0.390]	[-3.131, -1.714]	[0.838, 5.722]	[-0.119, 0.808]	[0.540, 3.202]	[0.520, 3.178]
Multilevel variance parameter: Level 1	0.255***	0.257***	0.479***	0.357***	0.543***	0.483***	-1.886***	0.027	0.026
	[0.097, 0.414]	[0.098, 0.416]	[0.264, 0.694]	[0.207, 0.506]	[0.342, 0.744]	[0.285, 0.681]	[-2.158, -1.614]	[-0.106, 0.161]	[-0.107, 0.158]
Multilevel variance parameter: Residual						1.786***	0.023		
						[1.756, 1.816]	[-0.039, 0.086]		
Level 1 Observations (child)	3196	3196	4373	4368	4373	4513	4946	6002	6002
Level 2 Observations (LGA)	121	121	121	121	121	121	121	121	121
Akaike Information Criterion	3244.008	3246.792	3803.166	5074.963	5338.697	29126.36	14392.094	1819.029	1817.343
Prob. > X^2	<0.001	<0.001	<0.001	<0.001	<0.001	<0.001	<0.001	<0.001	<0.001

95% confidence intervals in brackets

* p < 0.10, ** p < 0.05, *** p < 0.01
