# Supplementary material for: Impact and effect mechanisms of mass campaigns in resource-constrained health systems: quasi-experimental evidence from polio eradication in Nigeria
Source: BMJ Glob Health. 2021 Mar 8;6(3):e004248. doi: 10.1136/bmjgh-2020-004248 (PMC7942242; doi:10.1136/bmjgh-2020-004248)
Supplement: Supplementary data [file bmjgh-2020-004248supp009.pdf]

Table 8: Intraclass correlation on LGA level in aggregate two-level models.

Model	Intraclass correlation
Routine childhood immunisation	8.0%
Routine childhood immunisation (EXPxAGE interaction)	8.0%
Place of delivery: home	12.0%
Place of delivery: private facility	18.1%
Place of delivery: public facility	12.3%
No. of antenatal care visits	21.8%
No. of tetanus toxoid injections	4.6%
Child survival (exposure decomposition)	1.2%
Child survival (total exposure)	1.2%
Routine childhood immunisation	8.0%

Notes. Intraclass correlations for main results reported in Section 4.2, based on two-level logistic regression with LGA random effect. LGA = local government area.
