# Supplementary material for: Impact and effect mechanisms of mass campaigns in resource-constrained health systems: quasi-experimental evidence from polio eradication in Nigeria
Source: BMJ Glob Health. 2021 Mar 8;6(3):e004248. doi: 10.1136/bmjgh-2020-004248 (PMC7942242; doi:10.1136/bmjgh-2020-004248)
Supplement: Supplementary data [file bmjgh-2020-004248supp010.pdf]

Contents

Figure 1: Overview of SIA coverage of Nigerian LGAs, 2000-2017
Table 1: Variable Summary
Table 2: Model overview
Table 3: Main results: Link between SIA exposure and routine childhood immunisation uptake
Table 4: Main results: Link between SIA exposure and maternal health service uptake
Table 5: Main results: Link between SIA exposure and child survival
Table 6: Relationship between health system performance and SIA frequency across regions in survey sample.
Table 7: Detailed results: Link between SIA exposure and child/maternal health outcomes, stratified by region.
Table 8: Intraclass correlation on LGA level in aggregate two-level models.
Table 3a: Detailed results: Link between SIA exposure and routine childhood immunisation uptake
Table 3b: Robustness check (3-level models): Link between SIA exposure and routine childhood immunisation uptake
Table 4a: Detailed results: Link between SIA exposure and maternal health service uptake
Table 4b: Robustness check (3-level models): Link between SIA exposure and maternal health service uptake
Table 5a: Detailed results: Link between SIA exposure and child survival
Table 5b: Robustness check (3-level models): Link between SIA exposure and child survival
Table 6a: Additional robustness checks: exclusion of 2018 survey round to compensate for mop-up variability
Table 6b: Additional robustness checks: year-wise stratification to detect time trends
Table 6c: Additional robustness checks: exclusion of measles vaccine from full immunisation outcome
Table 6d: Additional robustness checks: decomposition of SIA exposure into different campaign types
Table 6e: Additional robustness checks: fixed effects models

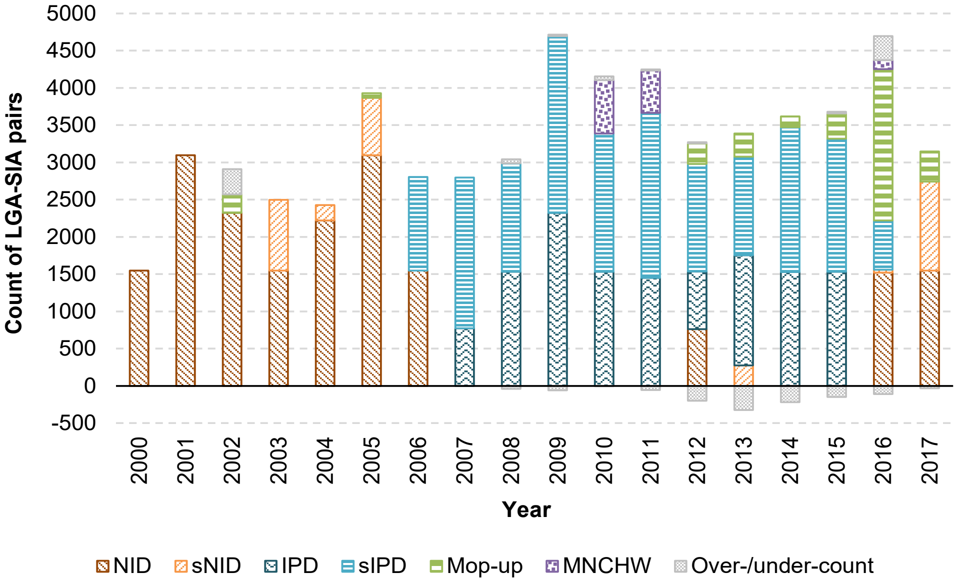

Figure 1. Overview of SIA coverage of Nigerian LGAs, 2000-2017

Source: Authors.

Notes: n = 59,127 LGA-SIA pairs (a total of 145 SIAs and 744 LGAs, with 45-120 SIAs per LGA between 2000 and 2017). NID = National Immunisation Day; sNID = sub-National Immunsiation Day; IPD = Immunisation-Plus Day; sIPD = sub-national Immunisation-Plus Day; MNCHW = Maternal, Neonatal, and Child Health Week; SIA = Supplementary Immunisation Activity; LGA = Local Government Area. "Mop-up" campaigns include revaccination and outbreak response campaigns. Over-/under-count refers to ability to match LGAs in accordance with SIA register of the World Health Organization, with over-count being displayed as negative values (i.e. representing excess LGA count).

Table 1: Variable Summary

Variable Name	Variable Description		N	Mean	Std. Dev.	Min	Max	Unique Values
**Outcome Variables: Childhood vaccination**								
**VAC**	Full vaccination (excl. polio & hepatitis B) [1 = yes]		69,376	0.27	0.44	0	1	2
**VAC_norep**	Full vaccination (excl. polio & hepatitis B, health card only) [1		18,792	0.44	0.5	0	1	2
**Outcome Variables: Pregnancy health service utilisation**								
**MOT_ANC**	Number of antenatal visits during pregnancy		55,790	4.57	5.07	0	29	30
**MOT_TET**	Number of tetanus toxoid injections before birth		57,962	1.31	1.32	0	9	10
**MOT_DEL_home**	Place of delivery: home [1 = yes]		87,532	0.65	0.48	0	1	2
**MOT_DEL_priv**	Place of delivery: private healthcare provider [1 = yes]		87,532	0.12	0.32	0	1	2
**MOT_DEL_pub**	Place of delivery: public healthcare provider [1 = yes]		87,532	0.24	0.42	0	1	2
**Outcome Variables: Child survival**								
**CHI_ALIVE**	Child is alive [1 = alive]		88,881	0.9	0.3	0	1	2
**Key Independent Variables: SIA Exposure**								
**EXP_CHI**	SIA exposure since birth (exact date)		58,453	11.11	9.71	0	42	43
**EXP_CHI_nod**	SIA exposure since birth (approx. date)		88,881	11.34	9.85	0	42	43
**EXP_CHI_RI**	SIA exposure since birth (exact date, RI period)		58,453	3.89	2.33	0	11	13
**EXP_CHI_RI_nod**	SIA exposure since birth (approx. date, RI period)		88,881	3.73	2.44	0	10	13
**EXP_CHI_FU**	SIA exposure since birth (exact date, follow-up period)		58,453	7.5	8.42	0	37	38
**EXP_CHI_FU_nod**	SIA exposure since birth (approx. date, follow-up period)		88,881	7.77	8.53	0	37	38
**EXP_PREG**	SIA exposure during pregnancy (exact date)		58,453	3.67	2.01	0	9	12
**EXP_PREG_nod**	SIA exposure during pregnancy (approximate date)		88,881	3.83	2.12	0	9	12
**EXP_TOT_nod**	Total SIA exposure pre- & post-birth (approximate date)		88,881	15.2	10.76	0	48	49
**Other Control Variables**								
**CHI_AGE**	Age of child (months)		88,881	28.29	17.35	0	60	61
**CHI_ORD**	Birth order of child		88,881	4.01	2.6	1	18	18
**CHI_SEX**	Sex of child [1 = female]		88,881	0.49	0.5	0	1	2
**MOT_AWE**	Child has health card [1 = yes]		68,957	0.52	0.5	0	1	2
**MOT_AGE**	Age of mother at birth (years)		88,881	27.1	6.9	12	49	38
**MOT_EDM**	Mother's highest year of education		88,842	2.43	2.64	0	10	10
**MOT_EDF**	Husband/partner's highest year of education		84,741	3.01	2.7	0	9	10
	Mother's religion	Catholic		0.08	0.28	0	1	
	(categorical variable)	Other Christian		0.3	0.46	0	1	
**HH_REL**		Islam	88,565	0.6	0.49	0	1	5
		Traditionalist		0.01	0.1	0	1	
		Other		0	0.05	0	1	
	Mother's ethnicity	Ekoi		0.01	0.08	0	1	
	(categorical variable)	Fulani		0.09	0.29	0	1	
		Hausa		0.32	0.47	0	1	
		Ibibio		0.01	0.12	0	1	
		Igala		0.01	0.1	0	1	
**HH_ETH**		Igbo	88,646	0.11	0.31	0	1	11
		Ijaw / Izon		0.03	0.17	0	1	
		Kanuri / Beriberi		0.02	0.15	0	1	
		Tiv		0.02	0.14	0	1	
		Yoruba		0.1	0.3	0	1	
		Other		0.27	0.44	0	1	
**HH_SIZ**	Number of household members		88,881	7.13	3.68	1	43	37
**HH_WEA**	Household asset wealth (out of 12 assets)		88,881	3.62	2.5	0	12	13
**INF_RUR**	Rural/urban residence [1 = rural]		88,881	0.69	0.46	0	1	2
	Survey year	2003		0.02	0.15	0	1	
	(categorical variable)	2008		0.29	0.45	0	1	
**YEAR**		2013	88,881	0.35	0.48	0	1	4
		2018		0.34	0.47	0	1	
**SIA intensity**	LGA above [2] or below [1] median total no. of SIAs 2000-2017		88,881	1.66	0.47	1	2	2

Table 2: Model overview

		Childhood vaccination (Table 1)		Pregnancy health service utilisation (Table 2)				Child survival (Table 3)	
	Outcome variables:	VAC		MOT_ANC<br>MOT_TET		MOT_DEL_home<br>MOT_DEL_priv<br>MOT_DEL_pub		CHI_ALIVE	
	Functional form:	Logistic regression		Linear regression		Logistic regression		Logistic regression	
	Analysis:	Main results	Robust-ness checks	Main results	Robust-ness checks	Main results	Robust-ness checks	Main results	Robust-ness checks
Key independent variables	EXP_CHI								
	EXP_CHI_RI								
	EXP_CHI_FU								
	EXP_CHI_nod								
	EXP_CHI_RI_nod								
	EXP_CHI_FU_nod								
	EXP_PREG								
	EXP_PREG_nod								
	EXP_TOT_nod								
	EXPxYR								
	EXPxAGE								
	EXPxAGExYEAR								
Other control variables	CHI_AGE								
	CHI_AGE^2								
	CHI_ORD								
	CHI_SEX								
	MOT_ANC								
	MOT_AWE								
	MOT_AGE								
	MOT_EDM								
	MOT_EDF								
	HH_REL								
	HH_ETH								
	HH_SIZ								
	HH_WEA								
	INF_RUR								
	YEAR								
	LGA random effect								
	State random effect								
	Fixed effects								
	Decomposition of SIAs								
	Stratification by region								
	Stratification by survey round								
	Exclusion of 2018 round								
	Non-polio/mealses RI outcome								
	Alternative reporting (health card only)								

Table 3: Main results: Link between SIA exposure and routine childhood immunisation uptake

Dependent variable: non-polio full immunisation status	Full model	Interaction model (EXPxAGE)	Exposure decomposition
EXP_CHI	-0.024*** [-0.036, -0.012]	0.012 [-0.009, 0.034]	
EXP_CHI_RI			-0.024* [-0.048, 0.000]
EXP_CHI_FU			-0.024*** [-0.037, -0.010]
EXPxAGE		-0.001*** [-0.001, -0.001]	
EXPxYR [yr = 2008]			
EXPxYR [yr = 2013]			
EXPxYR [yr = 2018]			
AGExYR [yr = 2008]			
AGExYR [yr = 2013]			
AGExYR [yr = 2018]			
EXPxAGExYR [yr = 2008]			
EXPxAGExYR [yr = 2013]			
EXPxAGExYR [yr = 2018]			
CHI_AGE	0.078*** [0.063, 0.093]	0.038*** [0.031, 0.044]	0.078*** [0.063, 0.093]
CHI_AGE2	-0.001*** [-0.001, -0.001]		-0.001*** [-0.001, -0.001]
Level 1 Observations (child)	24381	24381	24381
Level 2 Observations (LGA)	684	684	684
Akaike Information Criterion	18030.298	18055.959	18032.469
Prob. > X^2	<0.001	<0.001	<0.001

95% confidence intervals in brackets
* p < 0.10, ** p < 0.05, *** p < 0.01

Table 4: Main results: Link between SIA exposure and maternal health service uptake

Dependent variables: various indicators of maternal healthcare access (see right)	Delivery			Antenatal care	
	At home	At private facility	At public facility	No. of antenatal care visits	No. of tetanus injections
EXP_PREG	0.007<br>[-0.013, 0.027]	-0.065***<br>[-0.093, -0.036]	0.010<br>[-0.009, 0.029]	-0.025*<br>[-0.054, 0.003]	0.001<br>[-0.008, 0.009]
Level 1 Observations (child)	34713	34713	34713	35019	36585
Level 2 Observations (LGA)	686	686	686	686	687
Akaike Information Criterion	26422.845	17601.384	30318.818	193640.288	111227.001
Prob. > X^2	<0.001	<0.001	<0.001	<0.001	<0.001

95% confidence intervals in brackets

* p < 0.10, ** p < 0.05, *** p < 0.01

Table 5: Main results: Link between SIA exposure and child survival

Dependent variable: Child survival	Exposure decomposition	Total exposure
EXP_PREG_nod (date approximation)	-0.012<br>[-0.034, 0.010]	
EXP_CHI_nod (date approximation)	-0.019***<br>[-0.029, -0.009]	
EXP_TOT_nod (total exposure, date approximation)		-0.018***<br>[-0.027, -0.009]
Level 1 Observations (child)	52431	52431
Level 2 Observations (LGA)	689	689
Akaike Information Criterion	23684.534	23682.853
Prob. > X^2	<0.001	<0.001

95% confidence intervals in brackets

* p < 0.10, ** p < 0.05, *** p < 0.01

Table 6: Relationship between health system performance and SIA frequency across regions in survey sample.

Indicator	Region					
	North Central	North East	North West	South East	South South	South West
Average total number of SIAs at LGA level	75.81	100.17	113.94	47.07	47.38	50.61
Full immunisation status (incl. polio)[a]	21.9%	11.7%	8.3%	40.6%	31.5%	33.4%
Child exposure to SIAs	9.11	13.01	15.06	4.78	4.71	5.34
Mother's exposure to SIAs in pregnancy	3.28	4.42	5.07	1.89	1.87	2.07
Mother's average no. of ANC visits	4.68	2.78	2.3	8.16	6.35	10.75
Average place of delivery: home	49.5%	80.1%	87.2%	17.8%	50.4%	23.2%
Average place of delivery: private facility	16.5%	1.5%	1.5%	45.5%	15.6%	29.3%
Average place of delivery: public facility	34.0%	18.4%	11.3%	36.8%	34.0%	47.4%
Average survival of children under 5 years	91.8%	89.6%	87.4%	91.9%	93.1%	94.1%
Mothers' average years of education	3.05	1.49	1.16	4.57	4.42	4.25
Average household wealth index	3.88	2.54	2.93	4.96	4.75	5.1

Notes. Derived from DHS data. Unweighted statistics aggregated firstly at LGA and secondly at regional level, comprising full sample of children born between Oct 2000 and Dec 2017. ANC = antenatal care; DHS = Demographic and Health Survey; LGA = local government area; SIA = Supplementary Immunisation Activity.

[a.] Full immunisation status for children aged between 10 and 60 months, including at least 1 dose of BCG, 3 doses of DPT, 1 dose of measles vaccine, and three doses of oral polio vaccine.

Table 7: Detailed results: Link between SIA exposure and child/maternal health outcomes, stratified by region.

North Central	Non-polio full immunisation: Full model	Non-polio full immunisation: Interaction model (EXPxAGE)	Delivery: At home	Delivery: At private facility	Delivery: At public facility	Antenatal care: No. of antenatal care visits	Antenatal care: No. of tetanus injections	Child survival: Exposure decompositio n	Child survival: Total exposure
EXP_CHI	0.026* [-0.004, 0.056]	0.054** [0.009, 0.100]							
EXPxAGE		-0.001** [-0.002, -0.000]							
EXP_PREG			0.003 [-0.036, 0.042]	-0.015 [-0.061, 0.031]	0.002 [-0.034, 0.038]	0.034 [-0.030, 0.097]	0.003 [-0.015, 0.021]		
EXP_PREG_nod (date approximation)								-0.019 [-0.076, 0.037]	
EXP_CHI_nod (date approximation)								0.030* [-0.001, 0.061]	
EXP_TOT_nod (total exposure, date approximation)									0.018 [-0.008, 0.044]
CHI_AGE	0.056*** [0.025, 0.087]	0.017** [0.001, 0.033]						-0.003 [-0.027, 0.022]	-0.005 [-0.029, 0.018]
CHI_AGE2	-0.001*** [-0.001, -0.000]							0.000 [-0.001, 0.000]	0.000 [-0.000, 0.000]
CHI_ORD	-0.035 [-0.088, 0.019]	-0.034 [-0.087, 0.019]						-0.157*** [-0.220, -0.095]	-0.158*** [-0.221, -0.095]
CHI_SEX	-0.077 [-0.230, 0.077]	-0.075 [-0.228, 0.079]						0.217** [0.031, 0.404]	0.218** [0.032, 0.405]
MOT_ANC	0.093*** [0.071, 0.115]	0.094*** [0.072, 0.115]	-0.161*** [-0.181, -0.142]	0.061*** [0.043, 0.080]	0.083*** [0.067, 0.100]			0.004 [-0.023, 0.031]	0.004 [-0.023, 0.031]
MOT_EDM	0.070*** [0.035, 0.104]	0.070*** [0.035, 0.104]	-0.057*** [-0.085, -0.029]	0.060*** [0.025, 0.096]	0.031** [0.004, 0.059]	0.105*** [0.059, 0.151]	0.028*** [0.012, 0.043]	0.021 [-0.022, 0.063]	0.021 [-0.022, 0.063]
MOT_EDF	0.005 [-0.032, 0.042]	0.005 [-0.031, 0.042]	-0.024 [-0.053, 0.005]	0.029 [-0.008, 0.067]	0.023 [-0.005, 0.052]	0.053*** [0.016, 0.091]	0.008 [-0.008, 0.025]	0.010 [-0.033, 0.052]	0.010 [-0.032, 0.053]
MOT_AWE	2.554*** [2.315, 2.793]	2.547*** [2.309, 2.786]	-0.660*** [-0.809, -0.510]	-0.092 [-0.287, 0.102]	0.863*** [0.709, 1.018]	1.760*** [1.484, 2.035]	0.614*** [0.527, 0.701]		
MOT_AGE	0.016* [-0.001, 0.034]	0.016* [-0.001, 0.034]	0.005 [-0.005, 0.015]	-0.003 [-0.016, 0.009]	-0.003 [-0.013, 0.006]	0.026*** [0.010, 0.043]	0.004 [-0.002, 0.010]	0.010 [-0.011, 0.031]	0.010 [-0.011, 0.031]
HH_RUR	-0.035 [-0.257, 0.186]	-0.033 [-0.255, 0.188]	0.750*** [0.553, 0.946]	-0.405*** [-0.623, -0.187]	-0.425*** [-0.599, -0.251]	-0.559** [-1.103, -0.014]	-0.096* [-0.201, 0.009]	-0.079 [-0.336, 0.177]	-0.081 [-0.337, 0.176]
HH_REL (ref: Catholic): Other Christian	-0.105 [-0.373, 0.163]	-0.105 [-0.373, 0.163]	0.104 [-0.115, 0.324]	0.006 [-0.235, 0.246]	-0.106 [-0.316, 0.105]	0.160 [-0.217, 0.536]	0.037 [-0.096, 0.170]	0.164 [-0.142, 0.471]	0.165 [-0.142, 0.471]
HH_REL (ref: Catholic): Islam	-0.371** [-0.674, -0.069]	-0.370** [-0.673, -0.068]	0.095 [-0.160, 0.350]	0.000 [-0.289, 0.290]	-0.094 [-0.331, 0.143]	0.612*** [0.149, 1.074]	-0.069 [-0.232, 0.094]	0.118 [-0.220, 0.457]	0.117 [-0.222, 0.456]
HH_REL (ref: Catholic): Traditionalist	-0.719 [-1.837, 0.399]	-0.695 [-1.811, 0.421]	0.277 [-0.415, 0.969]	-0.011 [-0.861, 0.839]	-0.612 [-1.424, 0.201]	-0.985*** [-1.728, -0.243]	-0.625*** [-0.918, -0.331]	0.422 [-0.523, 1.367]	0.428 [-0.518, 1.373]
HH_REL (ref: Catholic): Other	0.000 [0.000, 0.000]	0.000 [0.000, 0.000]	-0.391 [-1.789, 1.007]	0.960 [-0.656, 2.575]	-0.172 [-1.783, 1.438]	-0.750 [-2.634, 1.134]	-0.409 [-1.097, 0.280]	0.022 [-2.033, 2.078]	0.015 [-2.042, 2.071]
HH_ETH (ref: Ekoi): Fulani	-0.085 [-0.632, 0.462]	-0.065 [-0.611, 0.481]	0.749*** [0.286, 1.212]	-1.294*** [-2.219, -0.368]	-0.500** [-0.939, -0.061]	0.000 [0.000, 0.000]	0.000 [0.000, 0.000]	0.364 [-0.165, 0.892]	0.370 [-0.158, 0.898]
HH_ETH (ref: Ekoi): Hausa	-0.492** [-0.881, -0.104]	-0.489** [-0.877, -0.101]	0.854*** [0.538, 1.170]	-0.647*** [-1.117, -0.177]	-0.546*** [-0.850, -0.243]	0.657* [-0.026, 1.340]	0.305** [0.031, 0.580]	0.079 [-0.301, 0.460]	0.078 [-0.303, 0.458]
HH_ETH (ref: Ekoi): Ibibio	-0.624 [-2.729, 1.481]	-0.628 [-2.732, 1.477]	-0.658 [-2.862, 1.546]	0.903 [-0.485, 2.291]	-0.511 [-1.878, 0.856]	1.908*** [0.643, 3.172]	0.364 [-0.234, 0.962]	0.000 [0.000, 0.000]	0.000 [0.000, 0.000]
HH_ETH (ref: Ekoi): Igala	-0.139 [-0.531, 0.253]	-0.141 [-0.533, 0.251]	-0.579*** [-0.962, -0.195]	0.276 [-0.102, 0.653]	0.418*** [0.102, 0.733]	1.477*** [0.605, 2.349]	0.848*** [0.551, 1.145]	0.019 [-0.407, 0.445]	0.015 [-0.411, 0.441]
HH_ETH (ref: Ekoi): Igbo	-0.163 [-0.689, 0.364]	-0.151 [-0.677, 0.374]	-1.009*** [-1.546, -0.471]	0.672*** [0.274, 1.069]	0.025 [-0.341, 0.391]	1.672*** [0.809, 2.535]	0.249* [-0.043, 0.541]	0.217 [-0.491, 0.926]	0.214 [-0.495, 0.923]
HH_ETH (ref: Ekoi): Ijaw / Izon	0.000 [0.000, 0.000]	0.000 [0.000, 0.000]	0.000 [0.000, 0.000]	0.000 [0.000, 0.000]	0.000 [0.000, 0.000]	-1.779 [-4.231, 0.674]	0.427** [0.080, 0.774]	0.000 [0.000, 0.000]	0.000 [0.000, 0.000]
HH_ETH (ref: Ekoi): Kanuri / Beriberi	-0.722 [-2.342, 0.898]	-0.707 [-2.327, 0.913]	0.647 [-0.406, 1.701]	0.100 [-1.070, 1.270]	-0.611 [-1.666, 0.444]	2.708** [0.535, 4.880]	0.213 [-0.284, 0.710]	-0.902* [-1.967, 0.162]	-0.888 [-1.952, 0.176]
HH_ETH (ref: Ekoi): Tiv	-0.116 [-0.491, 0.260]	-0.118 [-0.493, 0.257]	-0.808*** [-1.135, -0.481]	0.945*** [0.578, 1.312]	0.095 [-0.207, 0.398]	0.824** [0.013, 1.635]	0.218* [-0.009, 0.445]	0.088 [-0.253, 0.429]	0.084 [-0.258, 0.425]
HH_ETH (ref: Ekoi): Yoruba	0.183 [-0.145, 0.511]	0.191 [-0.137, 0.518]	-0.267 [-0.587, 0.052]	0.600*** [0.297, 0.904]	-0.310** [-0.568, -0.051]	2.871*** [2.117, 3.624]	0.472*** [0.214, 0.731]	0.301 [-0.089, 0.692]	0.305 [-0.086, 0.695]
HH_ETH (ref: Ekoi): Other	0.000 [0.000, 0.000]	0.000 [0.000, 0.000]	0.000 [0.000, 0.000]	0.000 [0.000, 0.000]	0.000 [0.000, 0.000]	1.335*** [0.666, 2.004]	0.516*** [0.319, 0.713]	0.000 [0.000, 0.000]	0.000 [0.000, 0.000]
HH_SIZ	-0.012 [-0.037, 0.014]	-0.012 [-0.038, 0.013]	0.007 [-0.012, 0.025]	0.003 [-0.020, 0.027]	-0.007 [-0.026, 0.011]	-0.067*** [-0.098, -0.036]	-0.014*** [-0.022, -0.005]	0.113*** [0.076, 0.149]	0.113*** [0.076, 0.150]
	0.150***	0.149***	-0.182***	0.088***	0.116***	0.304***	0.055***	0.015	0.015

HH_WEA	[0.107, 0.193]	[0.106, 0.192]	[-0.218, -0.146]	[0.046, 0.129]	[0.082, 0.149]	[0.225, 0.384]	[0.032, 0.078]	[-0.035, 0.065]	[-0.036, 0.065]
YEAR (ref: 2003): 2008	0.988***	0.989***	-0.331*	-0.344*	0.562***	-1.690***	-0.205	0.183	0.170
	[0.475, 1.501]	[0.474, 1.504]	[-0.691, 0.029]	[-0.742, 0.053]	[0.209, 0.915]	[-2.851, -0.529]	[-0.486, 0.077]	[-0.301, 0.667]	[-0.314, 0.653]
YEAR (ref: 2003): 2013	0.818***	0.837***	-0.433**	-0.216	0.537***	-1.049*	-0.120	0.412	0.406
	[0.311, 1.326]	[0.328, 1.345]	[-0.792, -0.075]	[-0.607, 0.175]	[0.189, 0.886]	[-2.289, 0.191]	[-0.393, 0.153]	[-0.093, 0.917]	[-0.098, 0.911]
YEAR (ref: 2003): 2018	1.134***	1.183***	-0.775***	-0.072	0.787***	-2.272***	-0.304**	0.398	0.376
	[0.609, 1.659]	[0.660, 1.707]	[-1.150, -0.400]	[-0.488, 0.343]	[0.421, 1.153]	[-3.442, -1.101]	[-0.579, -0.030]	[-0.090, 0.886]	[-0.111, 0.863]
Constant	-5.527***	-5.158***	1.901***	-2.371***	-2.459***	1.800***	0.579***	1.877***	1.829***
	[-6.348, -4.707]	[-5.930, -4.386]	[1.358, 2.444]	[-3.004, -1.737]	[-2.986, -1.932]	[0.559, 3.042]	[0.175, 0.983]	[1.097, 2.657]	[1.053, 2.606]
Multilevel variance parameter: Level 1	0.172***	0.172***	0.338***	0.410***	0.186***	0.030	-1.683***	0.000	0.000
	[0.066, 0.279]	[0.066, 0.278]	[0.185, 0.490]	[0.193, 0.628]	[0.090, 0.283]	[-0.159, 0.219]	[-2.031, -1.334]	[-0.000, 0.000]	[-0.000, 0.000]
Multilevel variance parameter: Residual						1.263***	0.254***		
						[1.193, 1.333]	[0.199, 0.309]		
Observations (Level 2)	4342	4342	6071	6071	6071	6103	6293	9028	9028
Observations (Level 1)	113	113	113	113	113	113	113	113	113
Akaike Information Criterion	4152.478	4159.676	6199.652	4691.431	6657.883	32962.923	21185.627	3710.053	3710.113
Prob. > X^2	<0.001	<0.001	<0.001	<0.001	<0.001	<0.001	<0.001	<0.001	<0.001

North East	Non-polio full immunisation		Delivery			Antenatal care		Child survival	
	**Full model**	**Interaction model (EXPxAGE)**	**At home**	**At private facility**	**At public facility**	**No. of antenatal care visits**	**No. of tetanus injections**	**Exposure decompositio n**	**Total exposure**
EXP_CHI	-0.074***	-0.058**							
	[-0.106, -0.042]	[-0.104, -0.013]							
EXPxAGE		0.000							
		[-0.001, 0.001]							
EXP_PREG			-0.025	0.024	0.018	0.015	0.001		
			[-0.067, 0.017]	[-0.103, 0.151]	[-0.024, 0.060]	[-0.026, 0.055]	[-0.017, 0.019]		
EXP_PREG_nod (date approximation)								0.019	
								[-0.022, 0.060]	
EXP_CHI_nod (date approximation)								0.005	
								[-0.020, 0.029]	
EXP_TOT_nod (total exposure, date approximation)									0.008
									[-0.013, 0.030]
CHI_AGE	0.085***	0.054***						-0.014	-0.014
	[0.043, 0.128]	[0.032, 0.077]						[-0.035, 0.008]	[-0.035, 0.007]
CHI_AGE2	-0.001*							0.000	0.000
	[-0.001, 0.000]							[-0.000, 0.000]	[-0.000, 0.000]
CHI_ORD	-0.072**	-0.073**						-0.139***	-0.139***
	[-0.135, -0.010]	[-0.136, -0.011]						[-0.186, -0.093]	[-0.186, -0.093]
CHI_SEX	0.026	0.024						0.271***	0.271***
	[-0.163, 0.215]	[-0.165, 0.213]						[0.121, 0.421]	[0.121, 0.420]
MOT_ANC	0.144***	0.144***	-0.218***	0.134***	0.203***			0.003	0.003
	[0.109, 0.179]	[0.109, 0.179]	[-0.244, -0.192]	[0.069, 0.200]	[0.178, 0.229]			[-0.026, 0.031]	[-0.026, 0.031]
MOT_EDM	0.072***	0.071***	-0.083***	0.120***	0.072***	0.092***	0.030***	-0.009	-0.009
	[0.030, 0.115]	[0.029, 0.114]	[-0.114, -0.051]	[0.030, 0.210]	[0.040, 0.104]	[0.054, 0.130]	[0.016, 0.044]	[-0.050, 0.031]	[-0.049, 0.031]
MOT_EDF	0.006	0.006	-0.028*	0.020	0.031*	0.104***	0.029***	0.038**	0.038**
	[-0.036, 0.048]	[-0.036, 0.047]	[-0.059, 0.003]	[-0.072, 0.112]	[-0.000, 0.062]	[0.071, 0.137]	[0.013, 0.044]	[0.002, 0.074]	[0.003, 0.074]
MOT_AWE	2.870***	2.872***	-0.834***	0.801***	0.806***	1.847***	0.740***		
	[2.563, 3.177]	[2.565, 3.179]	[-0.998, -0.669]	[0.290, 1.311]	[0.640, 0.973]	[1.626, 2.068]	[0.663, 0.818]		
MOT_AGE	0.031***	0.031***	0.007	0.012	-0.009	0.001	0.000	0.008	0.008
	[0.008, 0.055]	[0.007, 0.055]	[-0.004, 0.019]	[-0.021, 0.046]	[-0.021, 0.003]	[-0.009, 0.010]	[-0.003, 0.004]	[-0.010, 0.026]	[-0.010, 0.026]
HH_RUR	-0.159	-0.161	0.601***	0.123	-0.609***	-0.671***	-0.312***	-0.191	-0.190
	[-0.433, 0.115]	[-0.435, 0.114]	[0.388, 0.814]	[-0.501, 0.748]	[-0.817, -0.400]	[-1.014, -0.328]	[-0.443, -0.182]	[-0.432, 0.050]	[-0.430, 0.051]
HH_REL (ref: Catholic): Other Christian	-0.546	-0.558*	-0.366	0.534	0.338	0.285	0.114	-0.898**	-0.895**
	[-1.203, 0.112]	[-1.214, 0.098]	[-0.866, 0.133]	[-0.444, 1.512]	[-0.160, 0.836]	[-0.279, 0.848]	[-0.088, 0.315]	[-1.663, -0.132]	[-1.661, -0.129]
HH_REL (ref: Catholic): Islam	-1.072***	-1.088***	-0.018	0.105	0.051	0.073	0.050	-0.776**	-0.772**
	[-1.741, -0.402]	[-1.756, -0.420]	[-0.531, 0.495]	[-0.941, 1.151]	[-0.460, 0.562]	[-0.518, 0.663]	[-0.159, 0.260]	[-1.548, -0.004]	[-1.544, -0.000]
HH_REL (ref: Catholic): Traditionalist	-1.618**	-1.630**	0.789	0.000	-0.609	-0.676*	-0.263	-0.562	-0.560
	[-3.129, -0.107]	[-3.136, -0.124]	[-0.463, 2.042]	[0.000, 0.000]	[-1.842, 0.624]	[-1.376, 0.024]	[-0.583, 0.056]	[-1.751, 0.628]	[-1.749, 0.629]
HH_REL (ref: Catholic): Other	0.000	0.000	0.000	0.000	0.000	-2.056***	-0.787***	0.000	0.000
	[0.000, 0.000]	[0.000, 0.000]	[0.000, 0.000]	[0.000, 0.000]	[0.000, 0.000]	[-2.585, -1.526]	[-0.978, -0.596]	[0.000, 0.000]	[0.000, 0.000]
HH_ETH (ref: Ekoi): Fulani	-0.057	-0.065	-0.039	0.172	0.026	0.000	0.000	0.172	0.172
	[-0.358, 0.245]	[-0.366, 0.236]	[-0.265, 0.188]	[-0.518, 0.861]	[-0.201, 0.253]	[0.000, 0.000]	[0.000, 0.000]	[-0.049, 0.393]	[-0.049, 0.393]
HH_ETH (ref: Ekoi): Hausa	-0.078	-0.082	0.209*	-0.302	-0.168	0.552***	0.128**	0.005	0.004
	[-0.382, 0.226]	[-0.387, 0.222]	[-0.019, 0.438]	[-1.060, 0.455]	[-0.396, 0.059]	[0.197, 0.907]	[0.025, 0.231]	[-0.224, 0.234]	[-0.224, 0.233]
HH_ETH (ref: Ekoi): Ibibio	0.000	0.000	0.000	0.000	0.000	-1.698***	-0.297***	0.000	0.000
	[0.000, 0.000]	[0.000, 0.000]	[0.000, 0.000]	[0.000, 0.000]	[0.000, 0.000]	[-2.025, -1.371]	[-0.447, -0.146]	[0.000, 0.000]	[0.000, 0.000]
HH_ETH (ref: Ekoi): Igala	0.000	0.000	-0.981	2.001	0.221	0.707	0.077	-1.173	-1.184
	[0.000, 0.000]	[0.000, 0.000]	[-3.004, 1.041]	[-1.253, 5.254]	[-1.710, 2.152]	[-1.352, 2.765]	[-0.733, 0.888]	[-3.300, 0.954]	[-3.311, 0.943]
HH_ETH (ref: Ekoi): Igbo	0.487	0.501	0.000	1.367**	0.292	3.466***	0.444**	-0.922	-0.925
	[-1.189, 2.162]	[-1.172, 2.174]	[0.000, 0.000]	[0.048, 2.687]	[-0.831, 1.415]	[1.887, 5.045]	[0.091, 0.796]	[-2.188, 0.343]	[-2.191, 0.341]
HH_ETH (ref: Ekoi): Ijaw / Izon									
HH_ETH (ref: Ekoi): Kanuri / Beriberi	-0.220	-0.218	0.454***	-0.625	-0.405**	-0.059	-0.021	0.121	0.123
	[-0.684, 0.245]	[-0.683, 0.246]	[0.124, 0.784]	[-1.938, 0.688]	[-0.732, -0.078]	[-0.373, 0.255]	[-0.149, 0.107]	[-0.183, 0.425]	[-0.181, 0.426]
HH_ETH (ref: Ekoi): Tiv	-0.200	-0.236	-1.446***	-0.044	1.613***	-0.345	-0.123	0.065	0.069
	[-1.146, 0.746]	[-1.182, 0.710]	[-2.017, -0.874]	[-1.268, 1.180]	[1.035, 2.191]	[-1.423, 0.733]	[-0.585, 0.340]	[-0.716, 0.847]	[-0.712, 0.851]
HH_ETH (ref: Ekoi): Yoruba	0.351	0.367	-0.990	1.845**	0.112	0.855	0.000	0.000	0.000
	[-1.116, 1.818]	[-1.095, 1.829]	[-2.250, 0.270]	[0.224, 3.467]	[-1.022, 1.245]	[-1.072, 2.783]	[-0.583, 0.584]	[0.000, 0.000]	[0.000, 0.000]

HH_ETH (ref: Ekoi): Other	0.000	0.000	0.000	0.000	0.000	0.415***	0.191***	0.000	0.000
	[0.000, 0.000]	[0.000, 0.000]	[0.000, 0.000]	[0.000, 0.000]	[0.000, 0.000]	[0.151, 0.679]	[0.085, 0.298]	[0.000, 0.000]	[0.000, 0.000]
HH_SIZ	-0.022*	-0.021*	0.032***	-0.032	-0.028***	-0.015*	-0.007	0.096***	0.096***
	[-0.047, 0.003]	[-0.047, 0.004]	[0.013, 0.051]	[-0.090, 0.027]	[-0.046, -0.009]	[-0.033, 0.002]	[-0.016, 0.002]	[0.071, 0.121]	[0.071, 0.121]
HH_WEA	0.103***	0.103***	-0.237***	0.040	0.230***	0.240***	0.071***	0.033	0.033
	[0.050, 0.157]	[0.049, 0.156]	[-0.279, -0.196]	[-0.073, 0.154]	[0.188, 0.271]	[0.185, 0.295]	[0.051, 0.091]	[-0.015, 0.081]	[-0.015, 0.081]
YEAR (ref: 2003): 2008	0.788*	0.799*	0.726***	-1.900***	-0.354	0.217	0.109	0.097	0.108
	[-0.031, 1.608]	[-0.024, 1.622]	[0.299, 1.153]	[-2.812, -0.988]	[-0.790, 0.083]	[-0.214, 0.649]	[-0.068, 0.286]	[-0.318, 0.512]	[-0.305, 0.521]
YEAR (ref: 2003): 2013	1.338***	1.358***	0.396*	-1.756***	-0.054	0.217	0.224**	0.184	0.193
	[0.525, 2.151]	[0.542, 2.174]	[-0.026, 0.818]	[-2.679, -0.833]	[-0.484, 0.377]	[-0.198, 0.632]	[0.026, 0.421]	[-0.247, 0.615]	[-0.237, 0.623]
YEAR (ref: 2003): 2018	1.692***	1.741***	0.091	-1.457***	0.258	-0.034	0.308***	0.115	0.138
	[0.865, 2.518]	[0.915, 2.568]	[-0.352, 0.534]	[-2.441, -0.473]	[-0.195, 0.711]	[-0.467, 0.400]	[0.110, 0.505]	[-0.303, 0.532]	[-0.273, 0.548]
Constant	-5.962***	-5.653***	2.646***	-5.294***	-2.948***	1.088***	0.410***	3.024***	3.046***
	[-7.134, -4.790]	[-6.771, -4.535]	[1.930, 3.362]	[-7.033, -3.555]	[-3.666, -2.230]	[0.398, 1.778]	[0.106, 0.714]	[2.074, 3.974]	[2.099, 3.993]
Multilevel variance parameter: Level 1	0.158**	0.163***	0.317***	1.656***	0.237***	-0.479***	-1.597***	0.053*	0.052*
	[0.037, 0.278]	[0.040, 0.286]	[0.160, 0.475]	[0.508, 2.804]	[0.100, 0.374]	[-0.643, -0.316]	[-1.924, -1.270]	[-0.007, 0.113]	[-0.008, 0.112]
Multilevel variance parameter: Residual						0.924***	0.079***		
						[0.864, 0.985]	[0.035, 0.123]		
Observations (Level 2)	5017	5017	7350	7304	7370	7387	7479	11269	11269
Observations (Level 1)	100	100	100	100	100	100	100	101	101
Akaike Information Criterion	2854.824	2857.651	5075.901	966.734	5023.467	34819.738	22572.587	5492.777	5491.124
Prob. > X^2	<0.001	<0.001	<0.001	<0.001	<0.001	<0.001	<0.001	<0.001	<0.001

North West	Non-polio full immunisation		Delivery			Antenatal care		Child survival	
	Full model	Interaction model (EXPxAGE)	At home	At private facility	At public facility	No. of antenatal care visits	No. of tetanus injections	Exposure decompositio n	Total exposure
EXP_CHI	-0.076** [-0.151, -0.002]	-0.034 [-0.110, 0.042]							
EXPxAGE		-0.001* [-0.002, 0.000]							
EXP_PREG			-0.015 [-0.067, 0.037]	-0.059 [-0.197, 0.080]	0.025 [-0.028, 0.078]	0.025 [-0.005, 0.055]	0.002 [-0.013, 0.017]		
EXP_PREG_nod (date approximation)								-0.008 [-0.051, 0.035]	
EXP_CHI_nod (date approximation)								0.025 [-0.011, 0.060]	
EXP_TOT_nod (total exposure, date approximation)									0.013 [-0.019, 0.045]
CHI_AGE	0.105*** [0.038, 0.172]	0.065*** [0.017, 0.114]						-0.047*** [-0.072, -0.021]	-0.043*** [-0.068, -0.018]
CHI_AGE2	-0.001** [-0.001, -0.000]							0.000* [-0.000, 0.001]	0.000** [0.000, 0.001]
CHI_ORD	-0.063* [-0.129, 0.002]	-0.063* [-0.129, 0.002]						-0.162*** [-0.200, -0.124]	-0.162*** [-0.200, -0.124]
CHI_SEX	0.045 [-0.154, 0.245]	0.045 [-0.154, 0.245]						0.126** [0.010, 0.241]	0.125** [0.010, 0.241]
MOT_ANC	0.063*** [0.026, 0.099]	0.063*** [0.027, 0.099]	-0.203*** [-0.228, -0.178]	0.221*** [0.147, 0.296]	0.188*** [0.162, 0.213]			0.015 [-0.009, 0.040]	0.015 [-0.009, 0.040]
MOT_EDM	0.068*** [0.021, 0.114]	0.067*** [0.021, 0.114]	-0.058*** [-0.091, -0.025]	0.011 [-0.099, 0.120]	0.062*** [0.028, 0.095]	0.127*** [0.091, 0.163]	0.036*** [0.024, 0.049]	0.013 [-0.021, 0.048]	0.013 [-0.021, 0.048]
MOT_EDF	0.029 [-0.013, 0.071]	0.029 [-0.013, 0.071]	-0.007 [-0.039, 0.024]	-0.043 [-0.158, 0.071]	0.017 [-0.015, 0.048]	0.106*** [0.083, 0.128]	0.034*** [0.026, 0.043]	0.043*** [0.016, 0.070]	0.043*** [0.015, 0.070]
MOT_AWE	3.547*** [3.265, 3.828]	3.543*** [3.262, 3.824]	-0.696*** [-0.864, -0.528]	0.676** [0.030, 1.323]	0.688*** [0.517, 0.858]	1.471*** [1.270, 1.673]	0.505*** [0.428, 0.582]		
MOT_AGE	0.037*** [0.012, 0.062]	0.037*** [0.012, 0.062]	0.007 [-0.004, 0.019]	0.003 [-0.036, 0.043]	-0.009 [-0.021, 0.003]	0.004 [-0.003, 0.011]	-0.002* [-0.005, 0.000]	0.029*** [0.014, 0.044]	0.029*** [0.014, 0.044]
HH_RUR	-0.099 [-0.415, 0.218]	-0.101 [-0.417, 0.215]	0.777*** [0.572, 0.982]	-0.793** [-1.465, -0.120]	-0.765*** [-0.975, -0.555]	-0.776*** [-1.122, -0.429]	-0.260*** [-0.382, -0.139]	-0.131 [-0.317, 0.055]	-0.133 [-0.319, 0.054]
HH_REL (ref: Catholic): Other Christian	0.451 [-0.256, 1.157]	0.441 [-0.266, 1.147]	-0.394 [-0.948, 0.160]	-0.061 [-0.966, 0.845]	0.411 [-0.133, 0.955]	0.504 [-0.148, 1.156]	0.005 [-0.186, 0.195]	0.183 [-0.565, 0.930]	0.180 [-0.567, 0.927]
HH_REL (ref: Catholic): Islam	-0.326 [-1.138, 0.487]	-0.332 [-1.144, 0.479]	0.722** [0.116, 1.327]	-1.716*** [-2.973, -0.459]	-0.383 [-0.989, 0.224]	-0.201 [-0.859, 0.457]	-0.190 [-0.421, 0.041]	0.150 [-0.549, 0.850]	0.145 [-0.554, 0.844]
HH_REL (ref: Catholic): Traditionalist	-0.677 [-2.263, 0.910]	-0.669 [-2.251, 0.913]	0.942 [-0.244, 2.127]	0.236 [-2.038, 2.511]	-0.913 [-2.224, 0.398]	-0.536 [-1.263, 0.192]	-0.253** [-0.496, -0.010]	0.132 [-0.787, 1.050]	0.131 [-0.787, 1.049]
HH_REL (ref: Catholic): Other	0.000 [0.000, 0.000]	0.000 [0.000, 0.000]	0.000 [0.000, 0.000]	0.000 [0.000, 0.000]	0.000 [0.000, 0.000]	1.210 [-0.710, 3.129]	-0.414*** [-0.636, -0.192]	-1.208 [-3.688, 1.271]	-1.195 [-3.677, 1.286]
HH_ETH (ref: Ekoi): Fulani	0.087 [-0.590, 0.763]	0.089 [-0.587, 0.766]	0.537** [0.041, 1.032]	-0.375 [-1.834, 1.084]	-0.552** [-1.053, -0.050]	0.389*** [0.232, 0.546]	0.208*** [0.138, 0.278]	2.211 [-0.597, 5.018]	2.191 [-0.615, 4.996]
HH_ETH (ref: Ekoi): Hausa	0.339 [-0.180, 0.859]	0.338 [-0.181, 0.857]	0.474** [0.096, 0.851]	-0.792 [-1.816, 0.233]	-0.401** [-0.782, -0.021]	0.735*** [0.581, 0.889]	0.333*** [0.272, 0.394]	1.858 [-0.943, 4.659]	1.841 [-0.959, 4.641]
HH_ETH (ref: Ekoi): Ibibio	0.000 [0.000, 0.000]	0.000 [0.000, 0.000]	0.000 [0.000, 0.000]	0.000 [0.000, 0.000]	0.000 [0.000, 0.000]	0.980*** [0.667, 1.292]	-0.492 [-2.642, 1.658]	0.000 [0.000, 0.000]	0.000 [0.000, 0.000]
HH_ETH (ref: Ekoi): Igala	0.000 [0.000, 0.000]	0.000 [0.000, 0.000]	0.000 [0.000, 0.000]	0.000 [0.000, 0.000]	0.000 [0.000, 0.000]	3.909*** [3.548, 4.269]	2.169*** [2.042, 2.296]	0.000 [0.000, 0.000]	0.000 [0.000, 0.000]
HH_ETH (ref: Ekoi): Igbo	0.216 [-0.866, 1.298]	0.209 [-0.872, 1.290]	-1.134* [-2.357, 0.088]	-0.479 [-1.729, 0.772]	0.412 [-0.482, 1.306]	1.275** [0.105, 2.444]	0.422*** [0.130, 0.714]	0.000 [0.000, 0.000]	0.000 [0.000, 0.000]
HH_ETH (ref: Ekoi): Ijaw / Izon									
HH_ETH (ref: Ekoi): Kanuri / Beriberi	0.626 [-0.630, 1.882]	0.641 [-0.614, 1.895]	1.715** [0.170, 3.259]	0.000 [0.000, 0.000]	-1.428* [-2.963, 0.107]	1.051*** [0.453, 1.649]	0.828*** [0.349, 1.307]	2.261 [-0.649, 5.170]	2.241 [-0.667, 5.149]
HH_ETH (ref: Ekoi): Tiv	0.000 [0.000, 0.000]	0.000 [0.000, 0.000]	0.000 [0.000, 0.000]	1.472 [-1.265, 4.208]	0.197 [-2.515, 2.909]	0.694 [-0.510, 1.898]	-0.069 [-0.478, 0.339]	0.000 [0.000, 0.000]	0.000 [0.000, 0.000]
HH_ETH (ref: Ekoi): Yoruba	0.609 [-0.739, 1.958]	0.610 [-0.736, 1.957]	-0.492 [-1.475, 0.491]	0.489 [-0.939, 1.918]	0.230 [-0.676, 1.136]	0.606 [-0.445, 1.656]	0.475*** [0.162, 0.787]	2.118 [-1.034, 5.270]	2.107 [-1.043, 5.258]
	0.000	0.000	0.000	0.000	0.000	0.760***	0.386***	2.275	2.257

HH_ETH (ref: Ekoi): Other	[0.000, 0.000]	[0.000, 0.000]	[0.000, 0.000]	[0.000, 0.000]	[0.000, 0.000]	[0.412, 1.107]	[0.256, 0.516]	[-0.546, 5.096]	[-0.563, 5.076]
HH_SIZ	-0.009	-0.009	0.044***	-0.012	-0.038***	-0.016*	-0.006*	0.122***	0.122***
	[-0.037, 0.020]	[-0.037, 0.020]	[0.022, 0.065]	[-0.081, 0.058]	[-0.060, -0.017]	[-0.033, 0.001]	[-0.013, 0.001]	[0.101, 0.143]	[0.101, 0.143]
HH_WEA	0.133***	0.133***	-0.203***	0.286***	0.170***	0.221***	0.063***	-0.005	-0.005
	[0.080, 0.186]	[0.080, 0.186]	[-0.243, -0.163]	[0.166, 0.405]	[0.130, 0.209]	[0.183, 0.259]	[0.048, 0.079]	[-0.042, 0.031]	[-0.042, 0.031]
YEAR (ref: 2003): 2008	1.387***	1.287***	0.109	-0.886*	0.023	-0.710***	-0.120*	0.276	0.270
	[0.505, 2.270]	[0.402, 2.172]	[-0.313, 0.531]	[-1.808, 0.035]	[-0.417, 0.462]	[-1.208, -0.212]	[-0.257, 0.018]	[-0.075, 0.627]	[-0.081, 0.621]
YEAR (ref: 2003): 2013	1.739***	1.575***	-0.181	-1.844***	0.376*	-0.523*	0.032	0.289	0.309
	[0.753, 2.726]	[0.593, 2.557]	[-0.602, 0.239]	[-2.814, -0.874]	[-0.062, 0.813]	[-1.053, 0.006]	[-0.126, 0.190]	[-0.146, 0.723]	[-0.127, 0.745]
YEAR (ref: 2003): 2018	1.087**	1.170***	-0.450**	0.043	0.476**	-0.116	0.268***	0.272	0.207
	[0.216, 1.959]	[0.304, 2.035]	[-0.872, -0.028]	[-0.846, 0.932]	[0.037, 0.915]	[-0.623, 0.391]	[0.113, 0.424]	[-0.054, 0.599]	[-0.106, 0.520]
Constant	-8.155***	-7.870***	2.044***	-3.822***	-2.524***	1.132**	0.415**	-0.185	-0.229
	[-9.559, -6.752]	[-9.212, -6.529]	[1.300, 2.788]	[-5.669, -1.975]	[-3.287, -1.760]	[0.205, 2.059]	[0.095, 0.736]	[-3.118, 2.747]	[-3.160, 2.701]
Multilevel variance parameter: Level 1	0.708***	0.705***	0.134***	0.290	0.143***	-0.338***	-1.448***	0.025	0.026
	[0.391, 1.025]	[0.389, 1.020]	[0.038, 0.230]	[-0.208, 0.789]	[0.041, 0.244]	[-0.492, -0.183]	[-1.629, -1.267]	[-0.012, 0.062]	[-0.012, 0.063]
Multilevel variance parameter: Residual						0.845***	-0.043*		
						[0.798, 0.892]	[-0.094, 0.007]		
Observations (Level 2)	7249	7249	10429	10383	10432	10447	10663	16552	16552
Observations (Level 1)	166	166	166	166	166	166	166	166	166
Akaike Information Criterion	2869.01	2870.86	5101.199	687.393	5117.543	47639.029	29619.064	8734.342	8734.38
Prob. > X^2	<0.001	<0.001	<0.001	<0.001	<0.001	<0.001	<0.001	<0.001	<0.001

South East	Non-polio full immunisation		Delivery			Antenatal care		Child survival	
	Full model	Interaction model (EXPxAGE)	At home	At private facility	At public facility	No. of antenatal care visits	No. of tetanus injections	Exposure decompositio n	Total exposure
EXP_CHI	0.092	0.238**							
	[-0.035, 0.220]	[0.056, 0.420]							
EXPxAGE		-0.005***							
		[-0.008, -0.002]							
EXP_PREG			0.036	0.020	-0.049	0.155**	0.010		
			[-0.062, 0.135]	[-0.060, 0.099]	[-0.129, 0.030]	[0.001, 0.308]	[-0.021, 0.042]		
EXP_PREG_nod (date approximation)								0.036	
								[-0.116, 0.189]	
EXP_CHI_nod (date approximation)								-0.043	
								[-0.171, 0.085]	
EXP_TOT_nod (total exposure, date approximation)									-0.013
									[-0.126, 0.099]
CHI_AGE	0.112***	0.048***						-0.017	-0.013
	[0.069, 0.155]	[0.019, 0.078]						[-0.056, 0.022]	[-0.052, 0.025]
CHI_AGE2	-0.002***							0.000	0.000
	[-0.002, -0.001]							[-0.000, 0.001]	[-0.000, 0.001]
CHI_ORD	-0.037	-0.036						-0.122***	-0.122***
	[-0.100, 0.026]	[-0.099, 0.027]						[-0.195, -0.048]	[-0.195, -0.048]
CHI_SEX	-0.149	-0.148						0.386***	0.383***
	[-0.362, 0.063]	[-0.360, 0.065]						[0.127, 0.645]	[0.124, 0.642]
MOT_ANC	0.030***	0.030***	-0.104***	0.037***	0.021**			-0.008	-0.007
	[0.007, 0.053]	[0.007, 0.053]	[-0.131, -0.077]	[0.020, 0.055]	[0.003, 0.038]			[-0.033, 0.017]	[-0.032, 0.018]
MOT_EDM	0.055*	0.055*	-0.075**	-0.027	0.099***	0.056	0.040***	-0.001	-0.001
	[-0.004, 0.115]	[-0.004, 0.114]	[-0.132, -0.018]	[-0.075, 0.021]	[0.049, 0.148]	[-0.061, 0.173]	[0.020, 0.061]	[-0.071, 0.069]	[-0.071, 0.069]
MOT_EDF	0.016	0.016	-0.106***	0.055**	0.036	0.008	-0.004	0.024	0.026
	[-0.046, 0.079]	[-0.047, 0.078]	[-0.164, -0.048]	[0.003, 0.106]	[-0.016, 0.088]	[-0.095, 0.111]	[-0.032, 0.024]	[-0.046, 0.095]	[-0.045, 0.096]
MOT_AWE	2.623***	2.613***	-0.974***	0.199	0.802***	1.372***	0.448***		
	[2.236, 3.011]	[2.226, 3.000]	[-1.244, -0.703]	[-0.055, 0.452]	[0.520, 1.083]	[0.702, 2.042]	[0.274, 0.621]		
MOT_AGE	0.013	0.013	-0.020**	-0.001	0.012*	-0.001	0.002	-0.019	-0.019
	[-0.010, 0.036]	[-0.011, 0.036]	[-0.037, -0.002]	[-0.015, 0.013]	[-0.002, 0.026]	[-0.024, 0.022]	[-0.003, 0.007]	[-0.044, 0.007]	[-0.044, 0.007]
HH_RUR	-0.094	-0.102	-0.003	-0.129	0.181*	-0.018	-0.060	-0.306**	-0.305**
	[-0.343, 0.155]	[-0.351, 0.147]	[-0.271, 0.264]	[-0.333, 0.075]	[-0.020, 0.382]	[-0.493, 0.457]	[-0.141, 0.020]	[-0.571, -0.041]	[-0.570, -0.041]
HH_REL (ref: Catholic): Other Christian	-0.110	-0.103	0.016	-0.189**	0.206**	-0.235	-0.099**	-0.316**	-0.312**
	[-0.338, 0.118]	[-0.330, 0.125]	[-0.221, 0.254]	[-0.366, -0.012]	[0.028, 0.384]	[-0.572, 0.103]	[-0.175, -0.023]	[-0.590, -0.041]	[-0.587, -0.038]
HH_REL (ref: Catholic): Islam	-0.055	-0.031	-0.726	1.069	-0.588	0.705	-0.146	0.000	0.000
	[-2.549, 2.440]	[-2.503, 2.442]	[-3.253, 1.801]	[-0.661, 2.798]	[-2.638, 1.463]	[-2.529, 3.938]	[-0.858, 0.567]	[0.000, 0.000]	[0.000, 0.000]
HH_REL (ref: Catholic): Traditionalist	-0.235	-0.223	1.728***	-1.829***	-0.951**	-1.223**	-0.326**	-0.303	-0.299
	[-0.870, 0.400]	[-0.859, 0.412]	[1.049, 2.407]	[-2.694, -0.964]	[-1.693, -0.209]	[-2.193, -0.254]	[-0.646, -0.005]	[-1.031, 0.424]	[-1.027, 0.429]
HH_REL (ref: Catholic): Other	0.000	0.000	0.000	0.000	0.000	16.125***	1.493***	-1.997	-2.025
	[0.000, 0.000]	[0.000, 0.000]	[0.000, 0.000]	[0.000, 0.000]	[0.000, 0.000]	[15.152, 17.099]	[1.304, 1.683]	[-4.770, 0.776]	[-4.787, 0.737]
HH_ETH (ref: Ekoi): Fulani	0.000	0.000	0.000	0.000	0.000	0.000	0.000	0.000	0.000
	[0.000, 0.000]	[0.000, 0.000]	[0.000, 0.000]	[0.000, 0.000]	[0.000, 0.000]	[0.000, 0.000]	[0.000, 0.000]	[0.000, 0.000]	[0.000, 0.000]
HH_ETH (ref: Ekoi): Hausa	-1.150	-1.069	-0.604	0.283	-0.489	3.793***	0.286*	0.370	0.389
	[-2.979, 0.679]	[-2.890, 0.753]	[-3.250, 2.042]	[-1.011, 1.577]	[-1.841, 0.863]	[2.817, 4.769]	[-0.006, 0.578]	[-1.248, 1.989]	[-1.227, 2.006]
HH_ETH (ref: Ekoi): Ibibio	-0.356	-0.310	0.975	-1.174	0.507	0.393	0.100	0.296	0.303
	[-2.952, 2.240]	[-2.905, 2.285]	[-1.143, 3.094]	[-3.019, 0.671]	[-1.176, 2.190]	[-1.936, 2.722]	[-0.733, 0.933]	[-2.029, 2.622]	[-2.023, 2.629]
HH_ETH (ref: Ekoi): Igala	-0.604	-0.637	-0.394	-2.032	1.572*	3.192***	0.983	0.282	0.279
	[-3.173, 1.964]	[-3.211, 1.936]	[-2.689, 1.901]	[-4.466, 0.402]	[-0.222, 3.366]	[1.463, 4.921]	[-0.706, 2.673]	[-1.560, 2.124]	[-1.562, 2.121]
HH_ETH (ref: Ekoi): Igbo	-0.887	-0.856	-0.040	-0.183	0.120	3.875***	0.400***	0.828	0.839
	[-2.193, 0.419]	[-2.161, 0.449]	[-1.256, 1.177]	[-1.007, 0.640]	[-0.754, 0.994]	[3.160, 4.590]	[0.282, 0.518]	[-0.257, 1.914]	[-0.246, 1.924]
HH_ETH (ref: Ekoi): Ijaw / Izon	0.000	0.000	0.000	0.000	0.000	-0.404	-1.627***	0.000	0.000
	[0.000, 0.000]	[0.000, 0.000]	[0.000, 0.000]	[0.000, 0.000]	[0.000, 0.000]	[-1.516, 0.707]	[-1.814, -1.440]	[0.000, 0.000]	[0.000, 0.000]
HH_ETH (ref: Ekoi): Kanuri / Beriberi									
HH_ETH (ref: Ekoi): Tiv	0.000	0.000	0.000	0.000	0.000	-3.047***	-1.582***	0.000	0.000
	[0.000, 0.000]	[0.000, 0.000]	[0.000, 0.000]	[0.000, 0.000]	[0.000, 0.000]	[-3.747, -2.348]	[-1.708, -1.456]	[0.000, 0.000]	[0.000, 0.000]
HH_ETH (ref: Ekoi): Yoruba	0.000	0.000	0.220	-1.742*	1.350	2.502**	0.491	0.000	0.000
	[0.000, 0.000]	[0.000, 0.000]	[-2.478, 2.919]	[-3.529, 0.045]	[-0.360, 3.060]	[0.025, 4.978]	[-0.231, 1.212]	[0.000, 0.000]	[0.000, 0.000]
HH_ETH (ref: Ekoi): Other	0.000	0.000	0.000	0.000	0.000	4.086***	0.676***	0.000	0.000

HH_ETH (ref: Ekoi): Other	[0.000, 0.000]	[0.000, 0.000]	[0.000, 0.000]	[0.000, 0.000]	[0.000, 0.000]	[1.888, 6.284]	[0.389, 0.964]	[0.000, 0.000]	[0.000, 0.000]
HH_SIZ	-0.012	-0.013	0.023	-0.002	-0.012	-0.101***	-0.006	0.206***	0.206***
	[-0.052, 0.028]	[-0.053, 0.027]	[-0.017, 0.064]	[-0.035, 0.030]	[-0.045, 0.021]	[-0.166, -0.037]	[-0.018, 0.007]	[0.132, 0.280]	[0.132, 0.280]
HH_WEA	0.165***	0.164***	-0.214***	0.152***	-0.016	0.297***	0.046***	0.034	0.034
	[0.114, 0.217]	[0.113, 0.215]	[-0.268, -0.161]	[0.111, 0.192]	[-0.055, 0.024]	[0.201, 0.393]	[0.029, 0.064]	[-0.025, 0.093]	[-0.025, 0.093]
YEAR (ref: 2003): 2008	0.876***	0.878***	-0.072	0.063	-0.165	-0.647	-0.070	0.265	0.227
	[0.211, 1.541]	[0.214, 1.542]	[-0.638, 0.494]	[-0.404, 0.530]	[-0.641, 0.312]	[-1.778, 0.484]	[-0.286, 0.147]	[-0.444, 0.973]	[-0.477, 0.930]
YEAR (ref: 2003): 2013	0.906***	0.914***	-0.075	-0.150	0.092	0.659	0.113	0.234	0.166
	[0.250, 1.563]	[0.258, 1.571]	[-0.629, 0.479]	[-0.602, 0.302]	[-0.367, 0.551]	[-0.404, 1.722]	[-0.119, 0.345]	[-0.475, 0.943]	[-0.529, 0.861]
YEAR (ref: 2003): 2018	1.159***	1.301***	-0.147	-0.237	0.217	-0.653	0.027	0.468	0.462
	[0.408, 1.910]	[0.534, 2.067]	[-0.738, 0.444]	[-0.718, 0.244]	[-0.268, 0.702]	[-1.764, 0.458]	[-0.217, 0.270]	[-0.359, 1.295]	[-0.365, 1.289]
Constant	-4.957***	-4.578***	2.123***	-1.095**	-2.661***	2.038**	1.009***	1.657**	1.685**
	[-6.650, -3.263]	[-6.238, -2.917]	[0.628, 3.618]	[-2.177, -0.013]	[-3.796, -1.526]	[0.300, 3.777]	[0.663, 1.355]	[0.114, 3.199]	[0.144, 3.226]
Multilevel variance parameter: Level 1	0.169**	0.169**	0.750***	0.355***	0.238***	0.613***	-1.893***	0.000	0.000
	[0.034, 0.304]	[0.033, 0.304]	[0.346, 1.154]	[0.183, 0.528]	[0.105, 0.371]	[0.435, 0.791]	[-2.192, -1.594]	[-0.000, 0.000]	[-0.000, 0.000]
Multilevel variance parameter: Residual						1.510***	-0.008		
						[1.439, 1.582]	[-0.073, 0.056]		
Observations (Level 2)	2036	2036	2900	2900	2900	2949	3229	4362	4362
Observations (Level 1)	85	85	86	86	123	86	87	87	87
Akaike Information Criterion	2198.177	2202.549	2361.019	3572.969	3513.076	17468.034	9205.156	1926.233	1925.154
Prob. > X^2	<0.001	<0.001	<0.001	<0.001	<0.001	<0.001	<0.001	<0.001	<0.001

South South	Non-polio full immunisation		Delivery			Antenatal care		Child survival	
	Full model	Interaction model (EXPxAGE)	At home	At private facility	At public facility	No. of antenatal care visits	No. of tetanus injections	Exposure decomposition	Total exposure
EXP_CHI	0.065	0.171**							
	[-0.042, 0.171]	[0.001, 0.341]							
EXPxAGE		-0.003***							
		[-0.006, -0.001]							
EXP_PREG			-0.023	0.038	0.014	0.072	0.037*		
			[-0.102, 0.056]	[-0.074, 0.150]	[-0.060, 0.088]	[-0.055, 0.198]	[-0.000, 0.074]		
EXP_PREG_nod (date approximation)								-0.056	
								[-0.206, 0.094]	
EXP_CHI_nod (date approximation)								-0.005	
								[-0.139, 0.128]	
EXP_TOT_nod (total exposure, date approximation)									-0.026
									[-0.142, 0.091]
CHI_AGE	0.073***	0.028**						0.023	0.021
	[0.035, 0.110]	[0.003, 0.053]						[-0.015, 0.061]	[-0.016, 0.059]
CHI_AGE2	-0.001***							0.000	0.000
	[-0.002, -0.000]							[-0.001, 0.000]	[-0.001, 0.000]
CHI_ORD	-0.073**	-0.074**						-0.239***	-0.239***
	[-0.135, -0.010]	[-0.137, -0.011]						[-0.318, -0.160]	[-0.318, -0.160]
CHI_SEX	0.176*	0.173*						0.317**	0.317**
	[-0.016, 0.368]	[-0.019, 0.365]						[0.054, 0.580]	[0.054, 0.580]
MOT_ANC	0.039***	0.039***	-0.143***	0.072***	0.083***			0.017	0.017
	[0.019, 0.060]	[0.019, 0.060]	[-0.162, -0.124]	[0.050, 0.093]	[0.067, 0.100]			[-0.011, 0.046]	[-0.011, 0.046]
MOT_EDM	0.050*	0.050*	-0.023	0.028	0.015	0.075**	0.031***	0.033	0.033
	[-0.000, 0.101]	[-0.001, 0.101]	[-0.069, 0.023]	[-0.038, 0.094]	[-0.028, 0.058]	[0.005, 0.146]	[0.010, 0.051]	[-0.032, 0.097]	[-0.032, 0.097]
MOT_EDF	0.010	0.010	-0.005	0.029	-0.007	0.094*	-0.011	-0.036	-0.036
	[-0.046, 0.066]	[-0.046, 0.065]	[-0.055, 0.044]	[-0.044, 0.101]	[-0.054, 0.040]	[-0.000, 0.188]	[-0.032, 0.010]	[-0.113, 0.040]	[-0.113, 0.040]
MOT_AWE	2.921***	2.908***	-1.262***	0.825***	1.080***	2.596***	0.657***		
	[2.577, 3.265]	[2.564, 3.251]	[-1.510, -1.015]	[0.411, 1.239]	[0.830, 1.330]	[2.197, 2.995]	[0.539, 0.775]		
MOT_AGE	0.026**	0.026**	-0.030***	0.007	0.023***	0.019	0.001	-0.003	-0.003
	[0.005, 0.047]	[0.005, 0.048]	[-0.044, -0.016]	[-0.013, 0.027]	[0.010, 0.036]	[-0.006, 0.045]	[-0.005, 0.007]	[-0.031, 0.025]	[-0.031, 0.025]
HH_RUR	-0.040	-0.047	0.715***	-0.564***	-0.322***	-1.067***	-0.065	-0.405**	-0.405**
	[-0.280, 0.200]	[-0.288, 0.193]	[0.476, 0.953]	[-0.874, -0.253]	[-0.543, -0.100]	[-1.594, -0.539]	[-0.192, 0.062]	[-0.762, -0.048]	[-0.762, -0.049]
HH_REL (ref: Catholic): Other Christian	-0.391**	-0.396**	0.312**	0.237	-0.446***	-0.375	-0.106*	0.054	0.054
	[-0.741, -0.042]	[-0.746, -0.046]	[0.027, 0.598]	[-0.166, 0.641]	[-0.710, -0.182]	[-1.003, 0.253]	[-0.226, 0.015]	[-0.403, 0.510]	[-0.402, 0.510]
HH_REL (ref: Catholic): Islam	0.009	0.006	-0.084	0.063	0.157	0.019	0.169	0.786	0.787
	[-0.761, 0.779]	[-0.765, 0.776]	[-0.771, 0.603]	[-0.702, 0.829]	[-0.457, 0.771]	[-1.728, 1.765]	[-0.139, 0.477]	[-0.598, 2.169]	[-0.597, 2.171]
HH_REL (ref: Catholic): Traditionalist	-0.746	-0.727	0.634	-0.113	-0.469	0.748	-0.339	-0.562	-0.557
	[-1.750, 0.258]	[-1.732, 0.277]	[-0.311, 1.579]	[-1.255, 1.030]	[-1.335, 0.397]	[-0.852, 2.347]	[-0.789, 0.112]	[-1.539, 0.415]	[-1.534, 0.420]
HH_REL (ref: Catholic): Other	-0.300	-0.294	-0.562	0.000	0.518	-0.793	-0.237	0.000	0.000
	[-1.524, 0.924]	[-1.520, 0.932]	[-1.606, 0.482]	[0.000, 0.000]	[-0.484, 1.519]	[-2.518, 0.933]	[-1.005, 0.532]	[0.000, 0.000]	[0.000, 0.000]
HH_ETH (ref: Ekoi): Fulani									
HH_ETH (ref: Ekoi): Hausa	-1.438	-1.440	0.033	0.177	0.135	1.450	-0.187	-1.042	-1.025
	[-3.691, 0.815]	[-3.692, 0.813]	[-1.779, 1.845]	[-2.288, 2.643]	[-1.520, 1.790]	[-2.747, 5.646]	[-0.686, 0.312]	[-3.402, 1.317]	[-3.384, 1.334]
HH_ETH (ref: Ekoi): Ibibio	-0.923***	-0.920***	0.244	0.183	-0.129	0.598	-0.003	-0.496	-0.494
	[-1.445, -0.401]	[-1.444, -0.397]	[-0.267, 0.754]	[-0.805, 1.171]	[-0.625, 0.367]	[-0.506, 1.701]	[-0.274, 0.267]	[-1.258, 0.265]	[-1.255, 0.267]
HH_ETH (ref: Ekoi): Igala	-0.758	-0.740	0.050	1.096	-0.256	0.828	-0.337	-0.964	-0.963
	[-3.413, 1.898]	[-3.351, 1.871]	[-1.849, 1.949]	[-1.306, 3.499]	[-1.947, 1.436]	[-1.132, 2.787]	[-1.153, 0.479]	[-3.153, 1.225]	[-3.151, 1.224]
HH_ETH (ref: Ekoi): Igbo	-0.492*	-0.488*	-0.025	1.214***	-0.488**	1.838***	0.088	-0.304	-0.300
	[-1.051, 0.067]	[-1.049, 0.073]	[-0.539, 0.488]	[0.297, 2.130]	[-0.975, -0.000]	[0.562, 3.114]	[-0.165, 0.342]	[-1.113, 0.506]	[-1.109, 0.509]
HH_ETH (ref: Ekoi): Ijaw / Izon	-0.640**	-0.639**	0.804***	-0.043	-0.679***	0.095	-0.018	-0.213	-0.212
	[-1.138, -0.143]	[-1.140, -0.139]	[0.290, 1.317]	[-1.023, 0.938]	[-1.178, -0.180]	[-1.029, 1.219]	[-0.265, 0.229]	[-0.951, 0.526]	[-0.950, 0.526]
HH_ETH (ref: Ekoi): Kanuri / Beriberi	0.000	0.000	0.000	0.000	0.000			0.000	0.000
	[0.000, 0.000]	[0.000, 0.000]	[0.000, 0.000]	[0.000, 0.000]	[0.000, 0.000]			[0.000, 0.000]	[0.000, 0.000]
HH_ETH (ref: Ekoi): Tiv	0.000	0.000	0.000	0.000	0.000	1.582**	-1.433***	0.000	0.000
	[0.000, 0.000]	[0.000, 0.000]	[0.000, 0.000]	[0.000, 0.000]	[0.000, 0.000]	[0.257, 2.906]	[-1.688, -1.177]	[0.000, 0.000]	[0.000, 0.000]
HH_ETH (ref: Ekoi): Yoruba	-2.193***	-2.185***	-0.089	2.236***	-1.470***	1.253	0.005	0.131	0.137
	[-3.155, -1.231]	[-3.148, -1.222]	[-0.970, 0.791]	[1.101, 3.371]	[-2.336, -0.604]	[-0.524, 3.030]	[-0.370, 0.380]	[-1.496, 1.757]	[-1.491, 1.765]
	-0.887***	-0.885***	0.126	0.953**	-0.384*	1.037*	0.063	-0.202	-0.201

HH_ETH (ref: Ekoi): Other	[-1.352, -0.423]	[-1.352, -0.419]	[-0.319, 0.571]	[0.066, 1.840]	[-0.814, 0.046]	[-0.022, 2.095]	[-0.169, 0.294]	[-0.905, 0.500]	[-0.903, 0.501]
HH_SIZ	-0.016	-0.017	0.058***	-0.043	-0.030*	-0.002	-0.016*	0.314***	0.315***
	[-0.060, 0.027]	[-0.060, 0.027]	[0.020, 0.095]	[-0.095, 0.010]	[-0.065, 0.005]	[-0.067, 0.064]	[-0.033, 0.002]	[0.236, 0.393]	[0.236, 0.394]
HH_WEA	0.120***	0.119***	-0.180***	0.139***	0.103***	0.409***	0.066***	-0.041	-0.041
	[0.074, 0.167]	[0.072, 0.166]	[-0.223, -0.137]	[0.079, 0.199]	[0.063, 0.144]	[0.316, 0.502]	[0.043, 0.088]	[-0.105, 0.022]	[-0.104, 0.023]
YEAR (ref: 2003): 2008	0.898**	0.906**	0.499*	-0.197	-0.311	-0.350	-0.306***	1.135***	1.150***
	[0.146, 1.650]	[0.153, 1.658]	[-0.031, 1.029]	[-0.819, 0.425]	[-0.800, 0.179]	[-1.507, 0.807]	[-0.519, -0.093]	[0.505, 1.765]	[0.522, 1.779]
YEAR (ref: 2003): 2013	1.469***	1.461***	0.489*	-0.851***	-0.036	-1.238**	-0.284**	1.092***	1.138***
	[0.726, 2.212]	[0.716, 2.207]	[-0.034, 1.013]	[-1.468, -0.234]	[-0.517, 0.446]	[-2.391, -0.084]	[-0.503, -0.064]	[0.480, 1.704]	[0.543, 1.733]
YEAR (ref: 2003): 2018	1.745***	1.874***	0.674**	-0.759**	-0.153	-1.744***	-0.372***	1.288***	1.287***
	[0.917, 2.572]	[1.022, 2.726]	[0.117, 1.231]	[-1.427, -0.091]	[-0.669, 0.363]	[-2.894, -0.594]	[-0.601, -0.144]	[0.519, 2.058]	[0.518, 2.056]
Constant	-5.197***	-4.898***	2.076***	-4.455***	-2.101***	2.123**	1.270***	1.540**	1.500**
	[-6.400, -3.994]	[-6.061, -3.736]	[1.189, 2.964]	[-5.818, -3.092]	[-2.943, -1.259]	[0.111, 4.135]	[0.854, 1.686]	[0.273, 2.808]	[0.238, 2.761]
Multilevel variance parameter: Level 1	0.024	0.027	0.389***	0.640***	0.365***	0.216**	-1.507***	0.057	0.057
	[-0.040, 0.087]	[-0.038, 0.092]	[0.190, 0.587]	[0.282, 0.997]	[0.193, 0.538]	[0.007, 0.426]	[-1.738, -1.276]	[-0.072, 0.187]	[-0.072, 0.186]
Multilevel variance parameter: Residual						1.554***	0.190***		
						[1.495, 1.614]	[0.136, 0.244]		
Observations (Level 2)	2514	2514	3552	3528	3552	3620	3975	4979	4979
Observations (Level 1)	111	111	112	112	112	112	112	113	113
Akaike Information Criterion	2613.206	2616.454	3449.379	2094.164	3846.725	21688.628	12916.658	1912.315	1910.708
Prob. > X^2	<0.001	<0.001	<0.001	<0.001	<0.001	<0.001	<0.001	<0.001	<0.001

South West	Non-polio full immunisation		Delivery			Antenatal care		Child survival	
	**Full model**	**Interaction model (EXPxAGE)**	**At home**	**At private facility**	**At public facility**	**No. of antenatal care visits**	**No. of tetanus injections**	**Exposure decomposition**	**Total exposure**
EXP_CHI	-0.060<br>[-0.144, 0.024]	-0.019<br>[-0.138, 0.101]							
EXPxAGE		-0.002*<br>[-0.004, 0.000]							
EXP_PREG			0.055<br>[-0.013, 0.122]	-0.003<br>[-0.059, 0.052]	-0.033<br>[-0.087, 0.022]	-0.069<br>[-0.206, 0.069]	0.034***<br>[0.009, 0.058]		
EXP_PREG_nod (date approximation)								-0.021<br>[-0.145, 0.102]	
EXP_CHI_nod (date approximation)								0.018<br>[-0.103, 0.139]	
EXP_TOT_nod (total exposure, date approximation)									-0.001<br>[-0.103, 0.101]
CHI_AGE	0.083***<br>[0.050, 0.117]	0.056***<br>[0.035, 0.077]						0.035*<br>[-0.001, 0.071]	0.035*<br>[-0.001, 0.071]
CHI_AGE2	-0.001**<br>[-0.001, -0.000]							0.000<br>[-0.001, 0.000]	0.000<br>[-0.001, 0.000]
CHI_ORD	-0.087**<br>[-0.156, -0.017]	-0.087**<br>[-0.157, -0.018]						-0.369***<br>[-0.468, -0.271]	-0.367***<br>[-0.466, -0.269]
CHI_SEX	-0.006<br>[-0.182, 0.171]	-0.002<br>[-0.179, 0.174]						0.213<br>[-0.061, 0.486]	0.212<br>[-0.061, 0.486]
MOT_ANC	0.039***<br>[0.024, 0.054]	0.039***<br>[0.024, 0.054]	-0.064***<br>[-0.078, -0.049]	0.034***<br>[0.023, 0.045]	0.011*<br>[-0.000, 0.022]			-0.023**<br>[-0.045, -0.001]	-0.022**<br>[-0.044, -0.000]
MOT_EDM	0.042*<br>[-0.004, 0.088]	0.042*<br>[-0.004, 0.088]	-0.040*<br>[-0.082, 0.003]	0.054***<br>[0.016, 0.091]	-0.013<br>[-0.049, 0.023]	0.068<br>[-0.024, 0.160]	0.002<br>[-0.015, 0.019]	0.012<br>[-0.058, 0.082]	0.012<br>[-0.058, 0.082]
MOT_EDF	-0.031<br>[-0.080, 0.018]	-0.031<br>[-0.080, 0.018]	-0.015<br>[-0.059, 0.029]	-0.001<br>[-0.041, 0.038]	0.018<br>[-0.019, 0.055]	0.034<br>[-0.077, 0.146]	0.008<br>[-0.012, 0.028]	0.003<br>[-0.070, 0.075]	0.002<br>[-0.070, 0.074]
MOT_AWE	2.962***<br>[2.589, 3.336]	2.953***<br>[2.580, 3.327]	-1.082***<br>[-1.294, -0.870]	0.072<br>[-0.142, 0.286]	0.973***<br>[0.756, 1.190]	2.383***<br>[1.878, 2.889]	0.582***<br>[0.488, 0.675]		
MOT_AGE	0.031***<br>[0.011, 0.051]	0.031***<br>[0.011, 0.051]	-0.006<br>[-0.020, 0.008]	-0.005<br>[-0.018, 0.007]	0.011*<br>[-0.001, 0.023]	0.012<br>[-0.016, 0.041]	0.002<br>[-0.003, 0.006]	-0.012<br>[-0.041, 0.018]	-0.012<br>[-0.042, 0.017]
HH_RUR	-0.242*<br>[-0.489, 0.006]	-0.243*<br>[-0.490, 0.005]	0.028<br>[-0.217, 0.273]	-0.455***<br>[-0.668, -0.242]	0.333***<br>[0.129, 0.537]	0.423<br>[-0.286, 1.133]	-0.006<br>[-0.098, 0.086]	0.038<br>[-0.304, 0.380]	0.044<br>[-0.297, 0.385]
HH_REL (ref: Catholic): Other Christian	-0.095<br>[-0.593, 0.403]	-0.098<br>[-0.596, 0.400]	-0.232<br>[-0.672, 0.207]	0.171<br>[-0.186, 0.529]	-0.032<br>[-0.385, 0.321]	0.380<br>[-0.733, 1.493]	0.053<br>[-0.078, 0.185]	-0.180<br>[-0.859, 0.500]	-0.180<br>[-0.859, 0.499]
HH_REL (ref: Catholic): Islam	-0.402<br>[-0.919, 0.115]	-0.402<br>[-0.918, 0.114]	-0.261<br>[-0.718, 0.195]	0.213<br>[-0.164, 0.589]	-0.042<br>[-0.412, 0.328]	0.431<br>[-0.780, 1.642]	0.098<br>[-0.040, 0.235]	0.080<br>[-0.642, 0.803]	0.082<br>[-0.640, 0.804]
HH_REL (ref: Catholic): Traditionalist	-0.218<br>[-1.501, 1.066]	-0.228<br>[-1.508, 1.051]	0.404<br>[-0.674, 1.481]	-0.846<br>[-2.038, 0.345]	0.350<br>[-0.641, 1.341]	1.704<br>[-3.022, 6.429]	-0.393*<br>[-0.803, 0.017]	0.124<br>[-2.049, 2.298]	0.112<br>[-2.061, 2.285]
HH_REL (ref: Catholic): Other	0.994<br>[-3.233, 5.221]	0.992<br>[-3.182, 5.167]	0.337<br>[-2.751, 3.425]	0.000<br>[0.000, 0.000]	1.628<br>[-1.684, 4.939]	0.461<br>[-12.702, 13.625]	1.825<br>[-2.055, 5.705]	0.000<br>[0.000, 0.000]	0.000<br>[0.000, 0.000]
HH_ETH (ref: Ekoi): Fulani	-0.720<br>[-1.789, 0.349]	-0.713<br>[-1.784, 0.359]	1.513***<br>[0.758, 2.268]	-0.523<br>[-1.340, 0.294]	-2.137***<br>[-3.144, -1.131]	0.000<br>[0.000, 0.000]	0.000<br>[0.000, 0.000]	0.993<br>[-1.064, 3.050]	1.003<br>[-1.053, 3.060]
HH_ETH (ref: Ekoi): Hausa	-0.224<br>[-1.123, 0.675]	-0.227<br>[-1.125, 0.671]	0.613*<br>[-0.096, 1.322]	-0.986***<br>[-1.735, -0.237]	0.418<br>[-0.268, 1.105]	2.791**<br>[0.122, 5.461]	0.594**<br>[0.121, 1.067]	0.383<br>[-0.869, 1.634]	0.381<br>[-0.869, 1.631]
HH_ETH (ref: Ekoi): Ibibio	1.109<br>[-0.248, 2.466]	1.115<br>[-0.237, 2.468]	-0.264<br>[-1.248, 0.720]	0.026<br>[-0.791, 0.844]	0.257<br>[-0.618, 1.132]	3.338**<br>[0.715, 5.961]	0.499**<br>[0.045, 0.952]	-0.060<br>[-1.552, 1.433]	-0.068<br>[-1.560, 1.423]
HH_ETH (ref: Ekoi): Igala	0.685<br>[-0.655, 2.024]	0.694<br>[-0.645, 2.032]	-0.676<br>[-1.738, 0.385]	0.647<br>[-0.253, 1.547]	-0.081<br>[-1.019, 0.857]	3.470***<br>[1.030, 5.910]	0.776***<br>[0.247, 1.305]	0.000<br>[0.000, 0.000]	0.000<br>[0.000, 0.000]
HH_ETH (ref: Ekoi): Igbo	0.521**<br>[0.014, 1.028]	0.531**<br>[0.025, 1.038]	-1.501***<br>[-2.066, -0.936]	0.786***<br>[0.422, 1.150]	0.009<br>[-0.366, 0.384]	4.918***<br>[2.881, 6.955]	0.908***<br>[0.502, 1.315]	-0.024<br>[-0.650, 0.603]	-0.020<br>[-0.647, 0.606]
HH_ETH (ref: Ekoi): Ijaw / Izon	-0.625<br>[-2.141, 0.891]	-0.638<br>[-2.160, 0.884]	0.804<br>[-0.205, 1.812]	-0.273<br>[-1.215, 0.668]	-1.925**<br>[-3.431, -0.419]	2.087*<br>[-0.170, 4.344]	0.107<br>[-0.322, 0.536]	0.497<br>[-0.809, 1.804]	0.505<br>[-0.801, 1.811]
HH_ETH (ref: Ekoi): Kanuri / Beriberi	2.769*<br>[-0.166, 5.704]	2.780*<br>[-0.149, 5.710]	1.245<br>[-1.500, 3.990]	0.000<br>[0.000, 0.000]	1.406<br>[-1.171, 3.984]	2.783<br>[-2.223, 7.790]	0.080<br>[-1.800, 1.960]	0.000<br>[0.000, 0.000]	0.000<br>[0.000, 0.000]
HH_ETH (ref: Ekoi): Tiv	1.769*<br>[-0.032, 3.569]	1.775*<br>[-0.025, 3.574]	-0.566<br>[-1.650, 0.518]	-0.238<br>[-1.545, 1.068]	0.530<br>[-0.532, 1.592]	1.717<br>[-1.581, 5.014]	0.661*<br>[-0.123, 1.445]	-0.015<br>[-2.100, 2.070]	-0.011<br>[-2.096, 2.074]
HH_ETH (ref: Ekoi): Yoruba	0.289*<br>[-0.008, 0.587]	0.294*<br>[-0.004, 0.591]	-0.244*<br>[-0.505, 0.016]	-0.118<br>[-0.361, 0.126]	0.341***<br>[0.102, 0.580]	5.612***<br>[3.766, 7.457]	0.944***<br>[0.562, 1.325]	0.438**<br>[0.030, 0.846]	0.440**<br>[0.032, 0.848]

HH_ETH (ref: Ekoi): Other	0.000	0.000	0.000	0.000	0.000	4.747***	0.845***	0.000	0.000
	[0.000, 0.000]	[0.000, 0.000]	[0.000, 0.000]	[0.000, 0.000]	[0.000, 0.000]	[2.737, 6.757]	[0.439, 1.250]	[0.000, 0.000]	[0.000, 0.000]
HH_SIZ	-0.011	-0.011	0.031	-0.020	-0.008	-0.136***	0.002	0.499***	0.498***
	[-0.058, 0.036]	[-0.059, 0.036]	[-0.010, 0.071]	[-0.056, 0.016]	[-0.042, 0.026]	[-0.238, -0.035]	[-0.014, 0.018]	[0.399, 0.599]	[0.398, 0.598]
HH_WEA	0.142***	0.142***	-0.154***	0.066***	0.059***	0.298***	0.040***	-0.012	-0.011
	[0.090, 0.195]	[0.089, 0.195]	[-0.203, -0.105]	[0.024, 0.108]	[0.019, 0.099]	[0.193, 0.403]	[0.022, 0.058]	[-0.088, 0.064]	[-0.087, 0.064]
YEAR (ref: 2003): 2008	-0.257	-0.252	-0.027	-0.356*	0.338	-1.254**	-0.181**	-0.084	-0.065
	[-0.891, 0.378]	[-0.889, 0.385]	[-0.516, 0.461]	[-0.754, 0.041]	[-0.086, 0.763]	[-2.220, -0.288]	[-0.337, -0.024]	[-0.845, 0.677]	[-0.823, 0.694]
YEAR (ref: 2003): 2013	-0.749**	-0.726**	0.236	-0.495**	0.258	-0.078	-0.261***	0.140	0.174
	[-1.390, -0.108]	[-1.368, -0.084]	[-0.256, 0.728]	[-0.891, -0.100]	[-0.165, 0.681]	[-1.055, 0.898]	[-0.415, -0.107]	[-0.628, 0.908]	[-0.584, 0.933]
YEAR (ref: 2003): 2018	-0.694**	-0.656*	-0.072	-0.716***	0.709***	-3.705***	-0.425***	0.159	0.149
	[-1.384, -0.003]	[-1.349, 0.037]	[-0.595, 0.452]	[-1.140, -0.292]	[0.265, 1.153]	[-4.735, -2.674]	[-0.602, -0.247]	[-0.675, 0.993]	[-0.684, 0.982]
Constant	-4.520***	-4.319***	1.438***	-1.088***	-2.422***	3.280***	0.345	1.871***	1.849***
	[-5.602, -3.439]	[-5.373, -3.265]	[0.624, 2.252]	[-1.786, -0.390]	[-3.131, -1.714]	[0.838, 5.722]	[-0.119, 0.808]	[0.540, 3.202]	[0.520, 3.178]
Multilevel variance parameter: Level 1	0.255***	0.257***	0.479***	0.357***	0.543***	0.483***	-1.886***	0.027	0.026
	[0.097, 0.414]	[0.098, 0.416]	[0.264, 0.694]	[0.207, 0.506]	[0.342, 0.744]	[0.285, 0.681]	[-2.158, -1.614]	[-0.106, 0.161]	[-0.107, 0.158]
Multilevel variance parameter: Residual						1.786***	0.023		
						[1.756, 1.816]	[-0.039, 0.086]		
Level 1 Observations (child)	3196	3196	4373	4368	4373	4513	4946	6002	6002
Level 2 Observations (LGA)	121	121	121	121	121	121	121	121	121
Akaike Information Criterion	3244.008	3246.792	3803.166	5074.963	5338.697	29126.36	14392.094	1819.029	1817.343
Prob. > X^2	<0.001	<0.001	<0.001	<0.001	<0.001	<0.001	<0.001	<0.001	<0.001

95% confidence intervals in brackets

* p < 0.10, ** p < 0.05, *** p < 0.01

Table 8: Intraclass correlation on LGA level in aggregate two-level models.

Model	Intraclass correlation
Routine childhood immunisation	8.0%
Routine childhood immunisation (EXPxAGE interaction)	8.0%
Place of delivery: home	12.0%
Place of delivery: private facility	18.1%
Place of delivery: public facility	12.3%
No. of antenatal care visits	21.8%
No. of tetanus toxoid injections	4.6%
Child survival (exposure decomposition)	1.2%
Child survival (total exposure)	1.2%
Routine childhood immunisation	8.0%

Notes. Intraclass correlations for main results reported in Section 4.2, based on two-level logistic regression with LGA random effect. LGA = local government area.

Table 3a: Detailed results: Link between SIA exposure and routine childhood immunisation uptake

	Main results					Robustness checks: reporting			
				Year-Interaction models		Full model		Interaction model (EXPxAGE)	
Dependent variable: non-polio full immunisation status	Full model	Interaction model (EXPxAGE)	Exposure decomposition	(EXPxYR)	(EXPxAGExYR)	Only health card reported vaccines (VAC_norep)	Date approximation (EXP_CHI_nod)	Only health card reported vaccines (VAC_norep)	Date approximation (EXP_CHI_nod)
EXP_CHI	-0.024***	0.012		-0.053	-0.551	-0.002	-0.025***	0.036**	0.004
	[-0.036, -0.012]	[-0.009, 0.034]		[-0.217, 0.112]	[-1.460, 0.359]	[-0.021, 0.018]	[-0.036, -0.015]	[0.002, 0.070]	[-0.014, 0.022]
EXP_CHI_RI			-0.024*						
			[-0.048, 0.000]						
EXP_CHI_FU			-0.024***						
			[-0.037, -0.010]						
EXPxAGE		-0.001***			0.020			-0.001***	-0.001***
		[-0.001, -0.001]			[-0.032, 0.072]			[-0.002, -0.000]	[-0.001, -0.000]
EXPxYR [yr = 2008]				0.010	0.531				
				[-0.154, 0.174]	[-0.379, 1.440]				
EXPxYR [yr = 2013]				0.027	0.570				
				[-0.137, 0.190]	[-0.339, 1.479]				
EXPxYR [yr = 2018]				0.041	0.711				
				[-0.124, 0.205]	[-0.203, 1.626]				
AGExYR [yr = 2008]					-0.062				
					[-0.251, 0.126]				
AGExYR [yr = 2013]					-0.058				
					[-0.246, 0.130]				
AGExYR [yr = 2018]					-0.060				
					[-0.249, 0.129]				
EXPxAGExYR [yr = 2008]					-0.020				
					[-0.072, 0.032]				
EXPxAGExYR [yr = 2013]					-0.021				
					[-0.073, 0.031]				
EXPxAGExYR [yr = 2018]					-0.026				
					[-0.078, 0.026]				
CHI_AGE	0.078***	0.038***	0.078***	0.033***	0.098	0.069***	0.070***	0.033***	0.036***
	[0.063, 0.093]	[0.031, 0.044]	[0.063, 0.093]	[0.027, 0.039]	[-0.090, 0.286]	[0.046, 0.091]	[0.057, 0.083]	[0.023, 0.043]	[0.030, 0.042]
CHI_AGE2	-0.001***		-0.001***			-0.001***	-0.001***		
	[-0.001, -0.001]		[-0.001, -0.001]			[-0.001, -0.000]	[-0.001, -0.000]		
CHI_ORD	-0.060***	-0.061***	-0.061***	-0.061***	-0.062***	-0.083***	-0.076***	-0.083***	-0.077***
	[-0.085, -0.036]	[-0.086, -0.037]	[-0.085, -0.036]	[-0.085, -0.036]	[-0.086, -0.037]	[-0.121, -0.046]	[-0.098, -0.055]	[-0.121, -0.046]	[-0.099, -0.055]
CHI_SEX	-0.003	-0.004	-0.003	-0.007	-0.006	-0.036	0.014	-0.037	0.013
	[-0.078, 0.071]	[-0.079, 0.070]	[-0.078, 0.071]	[-0.081, 0.068]	[-0.081, 0.069]	[-0.146, 0.074]	[-0.050, 0.079]	[-0.147, 0.073]	[-0.051, 0.078]
MOT_ANC	0.055***	0.056***	0.055***	0.055***	0.055***	0.037***	0.059***	0.037***	0.059***
	[0.046, 0.064]	[0.047, 0.064]	[0.046, 0.064]	[0.046, 0.064]	[0.047, 0.064]	[0.025, 0.049]	[0.051, 0.067]	[0.025, 0.050]	[0.051, 0.067]
MOT_EDM	0.061***	0.061***	0.061***	0.061***	0.062***	0.050***	0.057***	0.049***	0.057***
	[0.043, 0.079]	[0.044, 0.079]	[0.043, 0.079]	[0.043, 0.079]	[0.044, 0.080]	[0.023, 0.077]	[0.041, 0.072]	[0.022, 0.076]	[0.041, 0.072]
MOT_EDF	0.010	0.010	0.010	0.009	0.010	-0.021	0.008	-0.021	0.008
	[-0.008, 0.028]	[-0.008, 0.028]	[-0.008, 0.028]	[-0.009, 0.028]	[-0.009, 0.028]	[-0.049, 0.007]	[-0.008, 0.024]	[-0.049, 0.008]	[-0.008, 0.024]
MOT_AWE	2.972***	2.969***	2.972***	2.957***	2.965***	0.000	2.990***	0.000	2.986***
	[2.848, 3.096]	[2.846, 3.093]	[2.848, 3.096]	[2.833, 3.080]	[2.841, 3.089]	[0.000, 0.000]	[2.882, 3.099]	[0.000, 0.000]	[2.878, 3.095]
MOT_AGE	0.026***	0.026***	0.026***	0.026***	0.027***	0.037***	0.031***	0.037***	0.032***
	[0.018, 0.035]	[0.018, 0.035]	[0.018, 0.035]	[0.017, 0.035]	[0.018, 0.035]	[0.024, 0.050]	[0.024, 0.039]	[0.024, 0.050]	[0.024, 0.039]
HH_RUR	-0.087*	-0.090*	-0.087*	-0.093*	-0.098*	-0.182**	-0.094**	-0.184**	-0.097**
	[-0.190, 0.016]	[-0.193, 0.013]	[-0.190, 0.016]	[-0.195, 0.010]	[-0.201, 0.005]	[-0.324, -0.039]	[-0.186, -0.002]	[-0.326, -0.042]	[-0.189, -0.005]
HH_REL (ref: Catholic): Other Christian	-0.126*	-0.128*	-0.126*	-0.130*	-0.124*	-0.131	-0.105*	-0.133	-0.107*
	[-0.267, 0.015]	[-0.269, 0.013]	[-0.267, 0.015]	[-0.271, 0.011]	[-0.265, 0.017]	[-0.318, 0.056]	[-0.230, 0.020]	[-0.320, 0.054]	[-0.232, 0.018]
HH_REL (ref: Catholic): Islam	-0.472***	-0.481***	-0.472***	-0.467***	-0.476***	-0.460***	-0.427***	-0.472***	-0.434***
	[-0.644, -0.300]	[-0.653, -0.308]	[-0.645, -0.300]	[-0.639, -0.295]	[-0.649, -0.304]	[-0.697, -0.222]	[-0.580, -0.275]	[-0.709, -0.234]	[-0.586, -0.281]
HH_REL (ref: Catholic): Traditionalist	-0.546***	-0.543***	-0.546***	-0.552***	-0.559***	-0.470	-0.513***	-0.474	-0.508***
	[-0.954, -0.139]	[-0.950, -0.137]	[-0.954, -0.139]	[-0.959, -0.146]	[-0.967, -0.152]	[-1.121, 0.182]	[-0.892, -0.135]	[-1.126, 0.178]	[-0.886, -0.129]
HH_REL (ref: Catholic): Other	-0.228	-0.222	-0.229	-0.207	-0.185	-0.956	-0.158	-0.957	-0.160
	[-1.246, 0.789]	[-1.236, 0.792]	[-1.246, 0.789]	[-1.220, 0.805]	[-1.198, 0.827]	[-2.528, 0.616]	[-0.927, 0.611]	[-2.528, 0.614]	[-0.927, 0.608]
HH_ETH (ref: Ekoi): Fulani	-0.930***	-0.958***	-0.931***	-0.866***	-0.900***	-0.782**	-0.961***	-0.799**	-0.983***
	[-1.438, -0.422]	[-1.467, -0.450]	[-1.438, -0.423]	[-1.374, -0.358]	[-1.411, -0.390]	[-1.430, -0.133]	[-1.423, -0.499]	[-1.448, -0.149]	[-1.445, -0.521]
HH_ETH (ref: Ekoi): Hausa	-0.851***	-0.892***	-0.852***	-0.777***	-0.828***	-0.983***	-0.771***	-1.018***	-0.798***
	[-1.337, -0.365]	[-1.379, -0.404]	[-1.339, -0.365]	[-1.263, -0.290]	[-1.318, -0.339]	[-1.585, -0.382]	[-1.217, -0.325]	[-1.621, -0.414]	[-1.245, -0.352]
HH_ETH (ref: Ekoi): Ibibio	-0.636**	-0.629**	-0.636**	-0.589**	-0.579**	-0.704**	-0.622**	-0.696**	-0.620**
	[-1.171, -0.101]	[-1.165, -0.094]	[-1.171, -0.101]	[-1.123, -0.055]	[-1.115, -0.044]	[-1.360, -0.047]	[-1.117, -0.126]	[-1.354, -0.038]	[-1.115, -0.124]
HH_ETH (ref: Ekoi): Igala	-0.660**	-0.659**	-0.660**	-0.610**	-0.600**	-0.540	-0.519*	-0.532	-0.521*
	[-1.245, -0.075]	[-1.245, -0.073]	[-1.246, -0.075]	[-1.194, -0.026]	[-1.186, -0.014]	[-1.326, 0.246]	[-1.058, 0.020]	[-1.320, 0.255]	[-1.061, 0.018]
HH_ETH (ref: Ekoi): Igbo	-0.453*	-0.449*	-0.453*	-0.409*	-0.404*	-0.804***	-0.428*	-0.804***	-0.427*
	[-0.922, 0.016]	[-0.919, 0.020]	[-0.922, 0.016]	[-0.876, 0.059]	[-0.874, 0.065]	[-1.362, -0.245]	[-0.864, 0.009]	[-1.364, -0.245]	[-0.864, 0.009]
HH_ETH (ref: Ekoi): Ijaw / Izon	-0.489*	-0.487*	-0.489*	-0.456*	-0.443	-1.058***	-0.358	-1.051***	-0.357
	[-1.029, 0.052]	[-1.029, 0.054]	[-1.029, 0.052]	[-0.995, 0.083]	[-0.984, 0.098]	[-1.737, -0.378]	[-0.859, 0.143]	[-1.731, -0.370]	[-0.858, 0.145]
HH_ETH (ref: Ekoi): Kanuri / Beriberi	-0.994***	-1.025***	-0.994***	-0.900***	-0.966***	-1.482***	-0.949***	-1.518***	-0.972***
	[-1.598, -0.389]	[-1.630, -0.420]	[-1.599, -0.390]	[-1.504, -0.295]	[-1.574, -0.359]	[-2.320, -0.643]	[-1.492, -0.405]	[-2.357, -0.679]	[-1.515, -0.428]
HH_ETH (ref: Ekoi): Tiv	-0.601**	-0.618**	-0.602**	-0.566**	-0.565**	-1.137***	-0.497*	-1.145***	-0.515**
	[-1.158, -0.045]	[-1.175, -0.060]	[-1.158, -0.045]	[-1.121, -0.010]	[-1.123, -0.007]	[-1.828, -0.445]	[-1.010, 0.017]	[-1.838, -0.452]	[-1.028, -0.001]
HH_ETH (ref: Ekoi): Yoruba	-0.574**	-0.573**	-0.574**	-0.526**	-0.527**	-0.775***	-0.479**	-0.773***	-0.480**
	[-1.051, -0.096]	[-1.051, -0.095]	[-1.051, -0.096]	[-1.002, -0.050]	[-1.006, -0.049]	[-1.351, -0.198]	[-0.923, -0.036]	[-1.351, -0.195]	[-0.924, -0.036]
HH_ETH (ref: Ekoi): Other	-0.620***	-0.632***	-0.620***	-0.558**	-0.577**	-0.802***	-0.542**	-0.810***	-0.553**
	[-1.081, -0.158]	[-1.094, -0.170]	[-1.082, -0.159]	[-1.019, -0.097]	[-1.040, -0.113]	[-1.356, -0.248]	[-0.970, -0.114]	[-1.365, -0.254]	[-0.981, -0.125]
HH_SIZ	-0.013**	-0.013**	-0.013**	-0.013**	-0.013**	-0.023**	-0.006	-0.023**	-0.006
	[-0.026, -0.000]	[-0.026, -0.001]	[-0.026, -0.000]	[-0.026, -0.001]	[-0.026, -0.001]	[-0.042, -0.003]	[-0.016, 0.005]	[-0.042, -0.003]	[-0.016, 0.005]
HH_WEA	0.142***	0.141***	0.142***	0.141***	0.142***	0.119***	0.119***	0.118***	0.119***
	[0.122, 0.162]	[0.121, 0.161]	[0.122, 0.162]	[0.121, 0.161]	[0.123, 0.162]	[0.090, 0.147]	[0.102, 0.136]	[0.089, 0.146]	[0.102, 0.135]
YEAR (ref: 2003): 2008	0.606***	0.604***	0.606***	0.693**	0.944	0.753***	0.712***	0.745***	0.710***
	[0.346, 0.866]	[0.343, 0.865]	[0.346, 0.866]	[0.102, 1.283]	[-1.902, 3.790]	[0.392, 1.113]	[0.470, 0.954]	[0.384, 1.107]	[0.467, 0.953]
YEAR (ref: 2003): 2013	0.749***	0.740***	0.748***	0.703**	0.779	0.873***	0.902***	0.864***	0.894***
	[0.491, 1.006]	[0.482, 0.999]	[0.491, 1.006]	[0.117, 1.289]	[-2.061, 3.618]	[0.518, 1.228]	[0.662, 1.142]	[0.509, 1.220]	[0.653, 1.135]
YEAR (ref: 2003): 2018	0.843***	0.919***	0.844***	0.771**	0.977	1.161***	1.056***	1.222***	1.115***
	[0.578, 1.108]	[0.654, 1.183]	[0.578, 1.109]	[0.177, 1.366]	[-1.879, 3.834]	[0.796, 1.526]	[0.817, 1.295]	[0.858, 1.586]	[0.877, 1.354]
Constant	-5.247***	-4.888***	-5.247***	-4.684***	-5.085***	-1.906***	-5.438***	-1.598***	-5.122***
	[-5.854, -4.640]	[-5.479, -4.296]	[-5.854, -4.640]	[-5.467, -3.902]	[-7.967, -2.202]	[-2.682, -1.130]	[-5.995, -4.880]	[-2.348, -0.849]	[-5.666, -4.578]
Multilevel variance parameter: Level 1	0.285***	0.286***	0.285***	0.280***	0.283***	0.254***	0.337***	0.255***	0.336***
	[0.218, 0.352]	[0.219, 0.353]	[0.218, 0.352]	[0.214, 0.346]	[0.216, 0.349]	[0.161, 0.347]	[0.269, 0.405]	[0.162, 0.348]	[0.268, 0.403]
Level 1 Observations (child)	24381	24381	24381	24381	24381	6514	31805	6514	31805
Level 2 Observations (LGA)	684	684	684	684	684	622	687	622	687
Akaike Information Criterion	18030.298	18055.959	18032.469	18070.207	18050.327	7972.642	23901.729	7980.84	23926.462
Prob. > X^2	<0.001	<0.001	<0.001	<0.001	<0.001	<0.001	<0.001	<0.001	<0.001

95% confidence intervals in brackets

* p < 0.10, ** p < 0.05, *** p < 0.01

Table 3b: Robustness Check (3-Level models): Link between SIA exposure and routine childhood immunisation uptake

Dependent variable: non-polio full immunisation status	Main results					Robustness checks: reporting			
				Year-Interaction models		Full model		Interaction model (EXPxAGE)	
	Full model	Exposure decomposition	Interaction model (EXPxAGE)	(EXPxYR)	(EXPxAGExYR)	Only health card reported vaccines (VAC_norep)	Date approximation (EXP_CHI_nod)	Only health card reported vaccines (VAC_norep)	Date approximation (EXP_CHI_nod)
EXP_CHI	-0.020***<br>[-0.033, -0.006]		0.023*<br>[-0.001, 0.047]	-0.067<br>[-0.231, 0.098]	-0.453<br>[-1.359, 0.453]	0.001<br>[-0.021, 0.022]	-0.022***<br>[-0.034, -0.011]	0.043**<br>[0.007, 0.079]	0.010<br>[-0.009, 0.030]
EXP_CHI_RI		-0.020<br>[-0.046, 0.005]							
EXP_CHI_FU		-0.020***<br>[-0.034, -0.005]							
EXPxAGE			-0.001***<br>[-0.001, -0.001]		0.015<br>[-0.037, 0.066]			-0.001***<br>[-0.002, -0.000]	-0.001***<br>[-0.001, -0.000]
EXPxYR [yr = 2008]				0.028<br>[-0.135, 0.191]	0.449<br>[-0.456, 1.354]				
EXPxYR [yr = 2013]				0.043<br>[-0.120, 0.206]	0.481<br>[-0.424, 1.386]				
EXPxYR [yr = 2018]				0.057<br>[-0.107, 0.220]	0.624<br>[-0.286, 1.534]				
AGExYR [yr = 2008]					-0.071<br>[-0.259, 0.117]				
AGExYR [yr = 2013]					-0.067<br>[-0.255, 0.121]				
AGExYR [yr = 2018]					-0.070<br>[-0.259, 0.118]				
EXPxAGExYR [yr = 2008]					-0.015<br>[-0.067, 0.037]				
EXPxAGExYR [yr = 2013]					-0.016<br>[-0.068, 0.036]				
EXPxAGExYR [yr = 2018]					-0.021<br>[-0.073, 0.032]				
CHI_AGE	0.076***<br>[0.061, 0.091]	0.076***<br>[0.061, 0.091]	0.036***<br>[0.029, 0.042]	0.032***<br>[0.025, 0.038]	0.106<br>[-0.082, 0.294]	0.068***<br>[0.045, 0.091]	0.069***<br>[0.055, 0.082]	0.032***<br>[0.022, 0.043]	0.034***<br>[0.028, 0.040]
CHI_AGE2	-0.001***<br>[-0.001, -0.001]	-0.001***<br>[-0.001, -0.001]				-0.001***<br>[-0.001, -0.000]	-0.001***<br>[-0.001, -0.000]		
CHI_ORD	-0.060***<br>[-0.085, -0.036]	-0.060***<br>[-0.085, -0.036]	-0.061***<br>[-0.086, -0.036]	-0.061***<br>[-0.085, -0.036]	-0.061***<br>[-0.086, -0.037]	-0.082***<br>[-0.120, -0.045]	-0.075***<br>[-0.097, -0.054]	-0.082***<br>[-0.120, -0.045]	-0.076***<br>[-0.098, -0.054]
CHI_SEX	-0.002<br>[-0.076, 0.073]	-0.002<br>[-0.076, 0.073]	-0.003<br>[-0.077, 0.072]	-0.004<br>[-0.079, 0.070]	-0.004<br>[-0.078, 0.070]	-0.040<br>[-0.150, 0.069]	0.014<br>[-0.050, 0.079]	-0.041<br>[-0.151, 0.069]	0.013<br>[-0.052, 0.078]
MOT_ANC	0.057***<br>[0.048, 0.066]	0.057***<br>[0.048, 0.066]	0.057***<br>[0.048, 0.066]	0.056***<br>[0.048, 0.065]	0.057***<br>[0.048, 0.066]	0.038***<br>[0.025, 0.051]	0.060***<br>[0.052, 0.068]	0.038***<br>[0.025, 0.051]	0.060***<br>[0.052, 0.068]
MOT_EDM	0.060***<br>[0.042, 0.078]	0.060***<br>[0.042, 0.078]	0.060***<br>[0.042, 0.078]	0.060***<br>[0.042, 0.078]	0.061***<br>[0.043, 0.079]	0.049***<br>[0.022, 0.076]	0.056***<br>[0.040, 0.071]	0.048***<br>[0.021, 0.075]	0.056***<br>[0.040, 0.071]
MOT_EDF	0.009<br>[-0.010, 0.027]	0.009<br>[-0.010, 0.027]	0.009<br>[-0.010, 0.027]	0.008<br>[-0.010, 0.027]	0.008<br>[-0.010, 0.027]	-0.023<br>[-0.051, 0.005]	0.007<br>[-0.008, 0.023]	-0.023<br>[-0.051, 0.005]	0.007<br>[-0.009, 0.023]
MOT_AWE	2.962***<br>[2.838, 3.086]	2.962***<br>[2.838, 3.086]	2.960***<br>[2.836, 3.084]	2.946***<br>[2.822, 3.070]	2.955***<br>[2.831, 3.079]	0.000<br>[0.000, 0.000]	2.991***<br>[2.882, 3.100]	0.000<br>[0.000, 0.000]	2.987***<br>[2.878, 3.096]
MOT_AGE	0.027***<br>[0.018, 0.035]	0.027***<br>[0.018, 0.035]	0.027***<br>[0.018, 0.036]	0.027***<br>[0.018, 0.035]	0.027***<br>[0.019, 0.036]	0.037***<br>[0.024, 0.050]	0.032***<br>[0.024, 0.039]	0.037***<br>[0.024, 0.050]	0.032***<br>[0.024, 0.039]
HH_RUR	-0.062<br>[-0.165, 0.040]	-0.062<br>[-0.165, 0.040]	-0.065<br>[-0.168, 0.038]	-0.068<br>[-0.170, 0.035]	-0.072<br>[-0.175, 0.031]	-0.176**<br>[-0.320, -0.033]	-0.066<br>[-0.159, 0.026]	-0.180**<br>[-0.323, -0.037]	-0.068<br>[-0.161, 0.024]
HH_REL (ref: Catholic): Other Christian	-0.112<br>[-0.254, 0.030]	-0.112<br>[-0.253, 0.030]	-0.114<br>[-0.256, 0.028]	-0.116<br>[-0.257, 0.025]	-0.109<br>[-0.251, 0.033]	-0.160*<br>[-0.349, 0.029]	-0.093<br>[-0.218, 0.033]	-0.163*<br>[-0.352, 0.026]	-0.094<br>[-0.220, 0.031]
HH_REL (ref: Catholic): Islam	-0.429***<br>[-0.605, -0.254]	-0.429***<br>[-0.605, -0.254]	-0.433***<br>[-0.608, -0.257]	-0.430***<br>[-0.605, -0.255]	-0.432***<br>[-0.607, -0.256]	-0.437***<br>[-0.680, -0.194]	-0.392***<br>[-0.548, -0.237]	-0.447***<br>[-0.690, -0.204]	-0.394***<br>[-0.550, -0.239]
HH_REL (ref: Catholic): Traditionalist	-0.498**<br>[-0.906, -0.091]	-0.498**<br>[-0.906, -0.090]	-0.493**<br>[-0.901, -0.085]	-0.503**<br>[-0.910, -0.096]	-0.509**<br>[-0.918, -0.101]	-0.476<br>[-1.127, 0.176]	-0.459**<br>[-0.840, -0.079]	-0.480<br>[-1.131, 0.172]	-0.453**<br>[-0.833, -0.073]
HH_REL (ref: Catholic): Other	-0.318<br>[-1.344, 0.709]	-0.318<br>[-1.345, 0.708]	-0.316<br>[-1.339, 0.708]	-0.292<br>[-1.312, 0.729]	-0.280<br>[-1.302, 0.742]	-1.100<br>[-2.685, 0.485]	-0.212<br>[-0.986, 0.562]	-1.106<br>[-2.689, 0.478]	-0.215<br>[-0.989, 0.558]
HH_ETH (ref: Ekoi): Fulani	-0.802***<br>[-1.341, -0.262]	-0.802***<br>[-1.342, -0.263]	-0.816***<br>[-1.357, -0.275]	-0.751***<br>[-1.290, -0.212]	-0.772***<br>[-1.314, -0.229]	-0.833**<br>[-1.522, -0.145]	-0.888***<br>[-1.378, -0.399]	-0.845**<br>[-1.535, -0.155]	-0.901***<br>[-1.391, -0.411]
HH_ETH (ref: Ekoi): Hausa	-0.703***<br>[-1.227, -0.179]	-0.703***<br>[-1.227, -0.179]	-0.722***<br>[-1.248, -0.196]	-0.649**<br>[-1.172, -0.125]	-0.677**<br>[-1.205, -0.150]	-1.106***<br>[-1.756, -0.455]	-0.673***<br>[-1.152, -0.195]	-1.131***<br>[-1.783, -0.479]	-0.686***<br>[-1.165, -0.207]
HH_ETH (ref: Ekoi): Ibibio	-0.468<br>[-1.030, 0.095]	-0.468<br>[-1.030, 0.095]	-0.458<br>[-1.022, 0.106]	-0.418<br>[-0.980, 0.144]	-0.419<br>[-0.983, 0.145]	-0.550<br>[-1.258, 0.159]	-0.492*<br>[-1.009, 0.026]	-0.537<br>[-1.247, 0.173]	-0.489*<br>[-1.007, 0.029]
HH_ETH (ref: Ekoi): Igala	-0.637**<br>[-1.266, -0.008]	-0.637**<br>[-1.266, -0.009]	-0.639**<br>[-1.268, -0.009]	-0.595*<br>[-1.222, 0.032]	-0.594*<br>[-1.224, 0.036]	-0.651<br>[-1.495, 0.194]	-0.505*<br>[-1.080, 0.069]	-0.646<br>[-1.492, 0.200]	-0.510*<br>[-1.085, 0.065]
HH_ETH (ref: Ekoi): Igbo	-0.332<br>[-0.849, 0.185]	-0.332<br>[-0.849, 0.185]	-0.324<br>[-0.842, 0.195]	-0.291<br>[-0.806, 0.225]	-0.294<br>[-0.812, 0.225]	-0.737**<br>[-1.358, -0.116]	-0.382<br>[-0.859, 0.095]	-0.732**<br>[-1.354, -0.109]	-0.382<br>[-0.859, 0.095]
HH_ETH (ref: Ekoi): Ijaw / Izon	-0.545*<br>[-1.142, 0.053]	-0.545*<br>[-1.142, 0.053]	-0.548*<br>[-1.147, 0.052]	-0.512*<br>[-1.108, 0.084]	-0.507*<br>[-1.106, 0.092]	-1.211***<br>[-1.974, -0.448]	-0.417<br>[-0.964, 0.130]	-1.202***<br>[-1.966, -0.437]	-0.418<br>[-0.966, 0.129]
HH_ETH (ref: Ekoi): Kanuri / Beriberi	-0.751**<br>[-1.386, -0.116]	-0.751**<br>[-1.387, -0.116]	-0.765**<br>[-1.401, -0.128]	-0.678**<br>[-1.312, -0.043]	-0.729**<br>[-1.367, -0.091]	-1.423***<br>[-2.294, -0.552]	-0.809***<br>[-1.379, -0.240]	-1.450***<br>[-2.322, -0.578]	-0.821***<br>[-1.391, -0.251]
HH_ETH (ref: Ekoi): Tiv	-0.556*<br>[-1.178, 0.067]	-0.556*<br>[-1.178, 0.067]	-0.571*<br>[-1.195, 0.053]	-0.530*<br>[-1.151, 0.091]	-0.526*<br>[-1.150, 0.099]	-0.969**<br>[-1.750, -0.188]	-0.482*<br>[-1.052, 0.088]	-0.974**<br>[-1.757, -0.191]	-0.497*<br>[-1.067, 0.074]
HH_ETH (ref: Ekoi): Yoruba	-0.428<br>[-0.950, 0.094]	-0.428<br>[-0.950, 0.094]	-0.427<br>[-0.950, 0.097]	-0.386<br>[-0.907, 0.135]	-0.394<br>[-0.918, 0.130]	-0.630*<br>[-1.269, 0.009]	-0.371<br>[-0.853, 0.111]	-0.624*<br>[-1.265, 0.016]	-0.373<br>[-0.855, 0.110]
HH_ETH (ref: Ekoi): Other	-0.526**<br>[-1.020, -0.031]	-0.526**<br>[-1.021, -0.032]	-0.530**<br>[-1.026, -0.034]	-0.475*<br>[-0.969, 0.019]	-0.487*<br>[-0.984, 0.010]	-0.828**<br>[-1.426, -0.230]	-0.496**<br>[-0.952, -0.039]	-0.833**<br>[-1.432, -0.233]	-0.501**<br>[-0.958, -0.044]
HH_SIZ	-0.014**<br>[-0.027, -0.001]	-0.014**<br>[-0.027, -0.001]	-0.014**<br>[-0.027, -0.001]	-0.014**<br>[-0.027, -0.001]	-0.014**<br>[-0.027, -0.001]	-0.023**<br>[-0.043, -0.004]	-0.007<br>[-0.017, 0.004]	-0.024**<br>[-0.043, -0.004]	-0.007<br>[-0.017, 0.004]
HH_WEA	0.140***<br>[0.121, 0.160]	0.140***<br>[0.121, 0.160]	0.139***<br>[0.119, 0.159]	0.140***<br>[0.120, 0.159]	0.141***<br>[0.121, 0.161]	0.116***<br>[0.087, 0.145]	0.119***<br>[0.102, 0.136]	0.115***<br>[0.086, 0.144]	0.118***<br>[0.101, 0.135]
YEAR (ref: 2003): 2008	0.636***<br>[0.377, 0.894]	0.636***<br>[0.377, 0.894]	0.625***<br>[0.366, 0.885]	0.662**<br>[0.076, 1.248]	1.088<br>[-1.748, 3.924]	0.789***<br>[0.429, 1.149]	0.729***<br>[0.487, 0.971]	0.774***<br>[0.414, 1.135]	0.723***<br>[0.480, 0.965]
YEAR (ref: 2003): 2013	0.768***<br>[0.511, 1.025]	0.768***<br>[0.511, 1.025]	0.746***<br>[0.487, 1.005]	0.679**<br>[0.097, 1.261]	0.943<br>[-1.887, 3.773]	0.894***<br>[0.538, 1.250]	0.908***<br>[0.667, 1.148]	0.875***<br>[0.518, 1.233]	0.893***<br>[0.651, 1.134]
YEAR (ref: 2003): 2018	0.899***<br>[0.635, 1.163]	0.900***<br>[0.635, 1.164]	0.980***<br>[0.717, 1.243]	0.774**<br>[0.182, 1.367]	1.222<br>[-1.626, 4.069]	1.202***<br>[0.838, 1.567]	1.094***<br>[0.855, 1.333]	1.266***<br>[0.902, 1.629]	1.155***<br>[0.917, 1.393]
Constant	-5.446***<br>[-6.091, -4.801]	-5.445***<br>[-6.090, -4.800]	-5.111***<br>[-5.743, -4.479]	-4.829***<br>[-5.638, -4.021]	-5.451***<br>[-8.332, -2.571]	-1.929***<br>[-2.749, -1.108]	-5.572***<br>[-6.166, -4.979]	-1.635***<br>[-2.430, -0.839]	-5.271***<br>[-5.853, -4.690]
Multilevel variance parameter: Level 1	0.133***<br>[0.054, 0.211]	0.133***<br>[0.054, 0.212]	0.144***<br>[0.058, 0.231]	0.127***<br>[0.052, 0.202]	0.140***<br>[0.056, 0.223]	0.114***<br>[0.036, 0.192]	0.146***<br>[0.064, 0.227]	0.118***<br>[0.038, 0.197]	0.152***<br>[0.067, 0.237]
Multilevel variance parameter: Level 2	0.177***<br>[0.122, 0.233]	0.177***<br>[0.122, 0.233]	0.176***<br>[0.120, 0.231]	0.174***<br>[0.119, 0.229]	0.174***<br>[0.119, 0.229]	0.165***<br>[0.086, 0.244]	0.225***<br>[0.168, 0.283]	0.164***<br>[0.085, 0.243]	0.222***<br>[0.166, 0.279]
Level 1 Observations (child)	24381	24381	24381	24381	24381	6514	31805	6514	31805
Level 2 Observations (LGA)	684	684	684	684	684	622	687	622	687
Level 3 Observations (state)	37	37	37	37	37	37	37	37	37
Akaike Information Criterion	17947.458	17949.558	17969.372	17988.064	17966.35	7940.803	23768.277	7947.817	23790.225
Prob. > X^2	<0.001	<0.001	<0.001	<0.001	<0.001	<0.001	<0.001	<0.001	<0.001

95% confidence intervals in brackets
* p < 0.10, ** p < 0.05, *** p < 0.01

Table 4a: Detailed results: Link between SIA exposure and maternal health service uptake

Dependent variables: various indicators of maternal healthcare access (see right)	Main results					Year-Interaction models					Robustness checks: reporting (date approximation)				
	Delivery			Antenatal care		Delivery			Antenatal care		Delivery			Antenatal care	
	At home	At private facility	At public facility	No. of antenatal care visits	No. of tetanus injections	At home	At private facility	At public facility	No. of antenatal care visits	No. of tetanus injections	At home	At private facility	At public facility	No. of antenatal care visits	No. of tetanus injections
EXP_PREG	0.007<br>[-0.013, 0.027]	-0.065***<br>[-0.093, -0.036]	0.010<br>[-0.009, 0.029]	-0.025*<br>[-0.054, 0.003]	0.001<br>[-0.008, 0.009]	0.102<br>[-0.086, 0.289]	-0.094<br>[-0.332, 0.144]	0.005<br>[-0.177, 0.187]	0.027<br>[-0.239, 0.292]	-0.040<br>[-0.107, 0.028]	-0.004<br>[-0.019, 0.012]	-0.060***<br>[-0.084, -0.036]	0.016**<br>[0.001, 0.031]	-0.013<br>[-0.033, 0.007]	0.001<br>[-0.005, 0.008]
EXPxYR [yr = 2008]						-0.064<br>[-0.253, 0.126]	0.013<br>[-0.230, 0.256]	-0.027<br>[-0.212, 0.157]	-0.065<br>[-0.337, 0.208]	0.009<br>[-0.060, 0.077]					
EXPxYR [yr = 2013]						-0.110<br>[-0.298, 0.078]	0.028<br>[-0.213, 0.268]	0.023<br>[-0.159, 0.206]	-0.130<br>[-0.401, 0.141]	0.037<br>[-0.032, 0.106]					
EXPxYR [yr = 2018]						-0.097<br>[-0.286, 0.092]	0.047<br>[-0.195, 0.289]	0.002<br>[-0.181, 0.185]	0.034<br>[-0.237, 0.304]	0.064*<br>[-0.005, 0.133]					
MOT_ANC	-0.137***<br>[-0.146, -0.129]	0.057***<br>[0.049, 0.065]	0.062***<br>[0.055, 0.068]			-0.137***<br>[-0.146, -0.129]	0.057***<br>[0.049, 0.064]	0.062***<br>[0.055, 0.068]			-0.144***<br>[-0.151, -0.136]	0.060***<br>[0.053, 0.068]	0.063***<br>[0.057, 0.069]		
MOT_EDM	-0.065***<br>[-0.079, -0.050]	0.052***<br>[0.032, 0.073]	0.057***<br>[0.043, 0.071]	0.097***<br>[0.074, 0.120]	0.031***<br>[0.024, 0.038]	-0.065***<br>[-0.079, -0.050]	0.052***<br>[0.032, 0.073]	0.057***<br>[0.043, 0.071]	0.097***<br>[0.074, 0.120]	0.031***<br>[0.024, 0.038]	-0.066***<br>[-0.078, -0.053]	0.051***<br>[0.033, 0.069]	0.061***<br>[0.049, 0.073]	0.095***<br>[0.075, 0.115]	0.031***<br>[0.025, 0.037]
MOT_EDF	-0.030***<br>[-0.045, -0.016]	0.033***<br>[0.012, 0.054]	0.035***<br>[0.020, 0.049]	0.082***<br>[0.063, 0.102]	0.023***<br>[0.017, 0.029]	-0.030***<br>[-0.045, -0.016]	0.033***<br>[0.011, 0.054]	0.035***<br>[0.020, 0.049]	0.082***<br>[0.063, 0.101]	0.023***<br>[0.017, 0.029]	-0.028***<br>[-0.040, -0.015]	0.027***<br>[0.007, 0.046]	0.038***<br>[0.026, 0.050]	0.076***<br>[0.059, 0.092]	0.025***<br>[0.020, 0.031]
MOT_AWE	-0.913***<br>[-0.988, -0.838]	0.284***<br>[0.172, 0.397]	0.978***<br>[0.901, 1.055]	1.946***<br>[1.813, 2.078]	0.643***<br>[0.603, 0.683]	-0.911***<br>[-0.986, -0.835]	0.282***<br>[0.169, 0.394]	0.977***<br>[0.900, 1.054]	1.928***<br>[1.796, 2.060]	0.637***<br>[0.596, 0.677]	-0.903***<br>[-0.968, -0.837]	0.282***<br>[0.181, 0.383]	0.957***<br>[0.890, 1.023]	1.905***<br>[1.791, 2.019]	0.646***<br>[0.611, 0.681]
MOT_AGE	-0.002<br>[-0.007, 0.003]	0.001<br>[-0.005, 0.008]	0.002<br>[-0.002, 0.007]	0.011***<br>[0.005, 0.017]	0.000<br>[-0.001, 0.002]	-0.003<br>[-0.008, 0.002]	0.001<br>[-0.005, 0.008]	0.002<br>[-0.002, 0.007]	0.011***<br>[0.005, 0.017]	0.001<br>[-0.001, 0.002]	0.000<br>[-0.005, 0.004]	0.001<br>[-0.005, 0.007]	0.001<br>[-0.003, 0.005]	0.007**<br>[0.001, 0.012]	0.000<br>[-0.002, 0.001]
HH_RUR	0.538***<br>[0.448, 0.628]	-0.374***<br>[-0.484, -0.264]	-0.325***<br>[-0.408, -0.241]	-0.588***<br>[-0.816, -0.360]	-0.156***<br>[-0.206, -0.105]	0.542***<br>[0.452, 0.632]	-0.376***<br>[-0.486, -0.266]	-0.329***<br>[-0.412, -0.245]	-0.585***<br>[-0.812, -0.359]	-0.159***<br>[-0.209, -0.108]	0.487***<br>[0.407, 0.567]	-0.341***<br>[-0.441, -0.241]	-0.300***<br>[-0.373, -0.226]	-0.579***<br>[-0.779, -0.380]	-0.160***<br>[-0.209, -0.112]
HH_REL (ref: Catholic): Other Christian	0.094<br>[-0.030, 0.217]	-0.097<br>[-0.219, 0.026]	0.012<br>[-0.097, 0.121]	-0.055<br>[-0.277, 0.167]	-0.046*<br>[-0.099, 0.006]	0.092<br>[-0.031, 0.215]	-0.097<br>[-0.219, 0.026]	0.013<br>[-0.096, 0.122]	-0.060<br>[-0.283, 0.163]	-0.047*<br>[-0.100, 0.006]	0.103*<br>[-0.007, 0.213]	-0.150***<br>[-0.259, -0.040]	0.055<br>[-0.042, 0.152]	-0.108<br>[-0.310, 0.094]	-0.040<br>[-0.087, 0.008]
HH_REL (ref: Catholic): Islam	0.225***<br>[0.076, 0.375]	-0.242***<br>[-0.403, -0.081]	-0.025<br>[-0.159, 0.110]	-0.168<br>[-0.467, 0.130]	-0.101***<br>[-0.174, -0.028]	0.225***<br>[0.075, 0.374]	-0.242***<br>[-0.403, -0.081]	-0.026<br>[-0.161, 0.108]	-0.170<br>[-0.469, 0.130]	-0.100***<br>[-0.173, -0.027]	0.231***<br>[0.098, 0.365]	-0.289***<br>[-0.435, -0.144]	0.022<br>[-0.098, 0.142]	-0.234*<br>[-0.501, 0.034]	-0.104***<br>[-0.171, -0.036]
HH_REL (ref: Catholic): Traditionalist	1.004***<br>[0.646, 1.362]	-0.911***<br>[-1.380, -0.442]	-0.475**<br>[-0.838, -0.111]	-0.653***<br>[-1.130, -0.176]	-0.350***<br>[-0.480, -0.219]	1.004***<br>[0.646, 1.362]	-0.913***<br>[-1.382, -0.444]	-0.474**<br>[-0.837, -0.111]	-0.665***<br>[-1.143, -0.187]	-0.354***<br>[-0.485, -0.223]	0.900***<br>[0.569, 1.231]	-0.866***<br>[-1.301, -0.430]	-0.406**<br>[-0.741, -0.070]	-0.619***<br>[-1.087, -0.151]	-0.282***<br>[-0.407, -0.158]
HH_REL (ref: Catholic): Other	-0.257<br>[-1.016, 0.503]	0.019<br>[-1.254, 1.292]	0.387<br>[-0.373, 1.148]	-0.084<br>[-1.535, 1.366]	-0.142<br>[-0.681, 0.397]	-0.265<br>[-1.024, 0.495]	0.028<br>[-1.246, 1.302]	0.388<br>[-0.373, 1.149]	0.020<br>[-1.431, 1.471]	-0.115<br>[-0.655, 0.424]	-0.189<br>[-0.779, 0.401]	0.115<br>[-0.877, 1.107]	0.286<br>[-0.301, 0.874]	-0.783<br>[-1.829, 0.262]	-0.287<br>[-0.662, 0.087]
HH_ETH (ref: Ekoi): Fulani	0.672***<br>[0.239, 1.104]	-0.084<br>[-0.988, 0.821]	-0.765***<br>[-1.190, -0.340]	-0.741<br>[-2.013, 0.531]	-0.349***<br>[-0.591, -0.107]	0.639***<br>[0.206, 1.072]	-0.080<br>[-0.985, 0.825]	-0.731***<br>[-1.156, -0.306]	-0.813<br>[-2.103, 0.477]	-0.324***<br>[-0.566, -0.081]	0.710***<br>[0.314, 1.106]	-0.065<br>[-0.816, 0.687]	-0.808***<br>[-1.192, -0.425]	-0.646<br>[-1.867, 0.576]	-0.331***<br>[-0.565, -0.097]
HH_ETH (ref: Ekoi): Hausa	0.941***<br>[0.521, 1.360]	-0.596<br>[-1.459, 0.266]	-0.820***<br>[-1.231, -0.408]	-0.436<br>[-1.703, 0.830]	-0.254**<br>[-0.493, -0.015]	0.908***<br>[0.488, 1.328]	-0.591<br>[-1.453, 0.271]	-0.787***<br>[-1.199, -0.374]	-0.506<br>[-1.790, 0.778]	-0.228*<br>[-0.468, 0.012]	0.971***<br>[0.584, 1.358]	-0.811**<br>[-1.537, -0.086]	-0.843***<br>[-1.216, -0.469]	-0.319<br>[-1.535, 0.896]	-0.228*<br>[-0.459, 0.003]
HH_ETH (ref: Ekoi): Ibibio	0.378<br>[-0.094, 0.850]	0.562<br>[-0.336, 1.459]	-0.310<br>[-0.771, 0.151]	-0.197<br>[-1.463, 1.070]	-0.102<br>[-0.385, 0.182]	0.350<br>[-0.122, 0.823]	0.565<br>[-0.332, 1.463]	-0.281<br>[-0.742, 0.180]	-0.254<br>[-1.535, 1.027]	-0.081<br>[-0.366, 0.204]	0.538**<br>[0.099, 0.977]	0.290<br>[-0.478, 1.058]	-0.449**<br>[-0.871, -0.027]	-0.011<br>[-1.219, 1.196]	-0.113<br>[-0.385, 0.160]
HH_ETH (ref: Ekoi): Igala	-0.510*<br>[-1.034, 0.015]	1.297***<br>[0.404, 2.190]	0.034<br>[-0.454, 0.522]	0.013<br>[-1.304, 1.329]	0.277*<br>[-0.017, 0.571]	-0.545**<br>[-1.070, -0.020]	1.301***<br>[0.408, 2.195]	0.069<br>[-0.419, 0.557]	-0.066<br>[-1.400, 1.268]	0.301**<br>[0.006, 0.595]	-0.417*<br>[-0.895, 0.061]	0.951**<br>[0.195, 1.707]	0.037<br>[-0.405, 0.478]	0.266<br>[-1.004, 1.535]	0.297**<br>[0.002, 0.591]
HH_ETH (ref: Ekoi): Igbo	-0.788***<br>[-1.203, -0.372]	1.877***<br>[1.046, 2.708]	-0.575***<br>[-0.979, -0.171]	1.258*<br>[-0.025, 2.540]	0.120<br>[-0.113, 0.353]	-0.813***<br>[-1.229, -0.397]	1.881***<br>[1.050, 2.712]	-0.547***<br>[-0.952, -0.143]	1.201*<br>[-0.099, 2.501]	0.141<br>[-0.092, 0.374]	-0.742***<br>[-1.129, -0.354]	1.545***<br>[0.846, 2.244]	-0.556***<br>[-0.925, -0.186]	1.273**<br>[0.037, 2.509]	0.122<br>[-0.104, 0.348]
HH_ETH (ref: Ekoi): Ijaw / Izon	0.887***<br>[0.401, 1.372]	0.122<br>[-0.804, 1.047]	-0.812***<br>[-1.287, -0.337]	-0.683<br>[-2.054, 0.688]	-0.133<br>[-0.397, 0.131]	0.863***<br>[0.377, 1.348]	0.122<br>[-0.803, 1.048]	-0.786***<br>[-1.261, -0.311]	-0.749<br>[-2.134, 0.636]	-0.120<br>[-0.383, 0.143]	0.841***<br>[0.395, 1.287]	-0.004<br>[-0.791, 0.783]	-0.828***<br>[-1.258, -0.397]	-0.583<br>[-1.910, 0.743]	-0.122<br>[-0.377, 0.133]
HH_ETH (ref: Ekoi): Kanuri / Beriberi	0.996***<br>[0.506, 1.487]	-0.539<br>[-1.680, 0.602]	-0.949***<br>[-1.430, -0.469]	-0.503<br>[-1.794, 0.787]	-0.239*<br>[-0.504, 0.026]	0.961***<br>[0.469, 1.453]	-0.533<br>[-1.674, 0.609]	-0.912***<br>[-1.393, -0.430]	-0.591<br>[-1.896, 0.715]	-0.211<br>[-0.477, 0.054]	0.869***<br>[0.427, 1.312]	-0.450<br>[-1.377, 0.477]	-0.859***<br>[-1.288, -0.430]	-0.396<br>[-1.636, 0.843]	-0.241*<br>[-0.492, 0.010]
HH_ETH (ref: Ekoi): Tiv	-0.776***<br>[-1.248, -0.304]	1.720***<br>[0.838, 2.601]	0.133<br>[-0.333, 0.600]	-0.896<br>[-2.238, 0.445]	-0.344**<br>[-0.612, -0.075]	-0.809***<br>[-1.281, -0.337]	1.722***<br>[0.841, 2.604]	0.164<br>[-0.303, 0.631]	-0.968<br>[-2.326, 0.389]	-0.321**<br>[-0.588, -0.054]	-0.651***<br>[-1.089, -0.213]	1.405***<br>[0.657, 2.152]	0.111<br>[-0.313, 0.535]	-0.698<br>[-1.982, 0.586]	-0.290**<br>[-0.541, -0.038]
HH_ETH (ref: Ekoi): Yoruba	-0.160<br>[-0.582, 0.261]	1.295***<br>[0.459, 2.132]	-0.418**<br>[-0.827, -0.009]	2.233***<br>[0.948, 3.518]	0.060<br>[-0.175, 0.295]	-0.189<br>[-0.611, 0.233]	1.300***<br>[0.463, 2.136]	-0.388*<br>[-0.797, 0.022]	2.177***<br>[0.874, 3.480]	0.078<br>[-0.157, 0.313]	-0.116<br>[-0.508, 0.276]	0.960***<br>[0.256, 1.664]	-0.374**<br>[-0.748, -0.000]	2.208***<br>[0.976, 3.439]	0.052<br>[-0.177, 0.281]
HH_ETH (ref: Ekoi): Other	0.206<br>[-0.196, 0.607]	0.905**<br>[0.076, 1.734]	-0.350*<br>[-0.744, 0.044]	0.035<br>[-1.215, 1.284]	-0.018<br>[-0.251, 0.214]	0.174<br>[-0.229, 0.576]	0.909**<br>[0.080, 1.738]	-0.318<br>[-0.713, 0.077]	-0.035<br>[-1.303, 1.232]	0.005<br>[-0.228, 0.238]	0.263<br>[-0.110, 0.636]	0.642*<br>[-0.054, 1.338]	-0.371**<br>[-0.731, -0.011]	0.132<br>[-1.070, 1.333]	-0.006<br>[-0.232, 0.219]
HH_SIZ	0.032***<br>[0.023, 0.042]	-0.017**<br>[-0.032, -0.003]	-0.020***<br>[-0.030, -0.011]	-0.043***<br>[-0.056, -0.030]	-0.006***<br>[-0.010, -0.002]	0.033***<br>[0.023, 0.042]	-0.017**<br>[-0.032, -0.002]	-0.020***<br>[-0.030, -0.011]	-0.043***<br>[-0.057, -0.030]	-0.006***<br>[-0.010, -0.002]	0.035***<br>[0.027, 0.044]	-0.021***<br>[-0.034, -0.008]	-0.023***<br>[-0.031, -0.015]	-0.038***<br>[-0.049, -0.027]	-0.005***<br>[-0.009, -0.002]
HH_WEA	-0.207***<br>[-0.223, -0.190]	0.120***<br>[0.099, 0.141]	0.130***<br>[0.114, 0.145]	0.286***<br>[0.256, 0.317]	0.058***<br>[0.050, 0.067]	-0.207***<br>[-0.224, -0.190]	0.120***<br>[0.099, 0.141]	0.129***<br>[0.114, 0.145]	0.288***<br>[0.258, 0.319]	0.059***<br>[0.051, 0.067]	-0.207***<br>[-0.222, -0.193]	0.128***<br>[0.110, 0.147]	0.129***<br>[0.116, 0.142]	0.261***<br>[0.235, 0.288]	0.053***<br>[0.046, 0.060]
YEAR (ref: 2003): 2008	0.013<br>[-0.168, 0.193]	-0.240**<br>[-0.450, -0.031]	0.079<br>[-0.094, 0.251]	-0.634***<br>[-1.029, -0.238]	-0.100**<br>[-0.188, -0.013]	0.146<br>[-0.348, 0.640]	-0.266<br>[-0.840, 0.309]	0.162<br>[-0.312, 0.636]	-0.459<br>[-1.284, 0.366]	-0.094<br>[-0.286, 0.098]	0.035<br>[-0.130, 0.200]	-0.236**<br>[-0.434, -0.038]	0.064<br>[-0.094, 0.223]	-0.542***<br>[-0.912, -0.172]	-0.089*<br>[-0.180, 0.001]
YEAR (ref: 2003): 2013	-0.025<br>[-0.204, 0.155]	-0.388***<br>[-0.594, -0.183]	0.143*<br>[-0.027, 0.314]	-0.258<br>[-0.668, 0.153]	-0.024<br>[-0.113, 0.065]	0.263<br>[-0.228, 0.755]	-0.449<br>[-1.018, 0.121]	0.064<br>[-0.405, 0.534]	0.186<br>[-0.638, 1.011]	-0.112<br>[-0.303, 0.078]	-0.021<br>[-0.185, 0.142]	-0.355***<br>[-0.549, -0.161]	0.116<br>[-0.039, 0.272]	-0.141<br>[-0.528, 0.246]	-0.001<br>[-0.092, 0.090]
YEAR (ref: 2003): 2018	-0.247***<br>[-0.433, -0.061]	-0.401***<br>[-0.620, -0.182]	0.354***<br>[0.178, 0.531]	-1.242***<br>[-1.651, -0.834]	-0.046<br>[-0.139, 0.048]	-0.011<br>[-0.505, 0.482]	-0.498*<br>[-1.071, 0.074]	0.350<br>[-0.122, 0.821]	-1.370***<br>[-2.184, -0.556]	-0.217**<br>[-0.408, -0.027]	-0.282***<br>[-0.443, -0.120]	-0.412***<br>[-0.608, -0.216]	0.379***<br>[0.225, 0.534]	-0.998***<br>[-1.377, -0.620]	-0.009<br>[-0.103, 0.084]
Constant	2.049***<br>[1.575, 2.524]	-3.635***<br>[-4.523, -2.747]	-2.439***<br>[-2.903, -1.975]	3.562***<br>[2.191, 4.933]	0.987***<br>[0.723, 1.251]	1.851***<br>[1.200, 2.503]	-3.572***<br>[-4.602, -2.542]	-2.455***<br>[-3.088, -1.821]	3.498***<br>[1.962, 5.033]	1.065***<br>[0.745, 1.386]	2.011***<br>[1.575, 2.448]	-3.405***<br>[-4.160, -2.650]	-2.453***<br>[-2.874, -2.032]	3.596***<br>[2.289, 4.904]	0.976***<br>[0.724, 1.228]
Multilevel variance parameter: Level 1	0.449***<br>[0.362, 0.536]	0.726***<br>[0.565, 0.888]	0.462***<br>[0.383, 0.542]	0.683***<br>[0.597, 0.769]	-1.428***<br>[-1.525, -1.330]	0.445***<br>[0.359, 0.531]	0.723***<br>[0.562, 0.884]	0.460***<br>[0.381, 0.539]	0.672***<br>[0.585, 0.759]	-1.456***<br>[-1.554, -1.359]	0.501***<br>[0.414, 0.589]	0.767***<br>[0.603, 0.931]	0.460***<br>[0.387, 0.533]	0.675***<br>[0.595, 0.756]	-1.405***<br>[-1.498, -1.313]
Multilevel variance parameter: Residual				1.322***<br>[1.282, 1.361]	0.090***<br>[0.064, 0.116]				1.321***<br>[1.282, 1.361]	0.090***<br>[0.064, 0.115]				1.281***<br>[1.243, 1.319]	0.085***<br>[0.063, 0.107]
Level 1 Observations (child)	34713	34713	34713	35019	36585	34713	34713	34713	35019	36585	45437	45437	45437	45839	47644
Level 2 Observations (LGA)	686	686	686	686	687	686	686	686	686	687	689	689	689	689	690
Akaike Information Criterion	26422.845	17601.384	30318.818	193640.288	111227.001	26423.667	17606.418	30319.207	193610.157	111190.932	34655.448	21928.17	39661.692	249499.916	144291.105
Prob. > X^2	<0.001	<0.001	<0.001	<0.001	<0.001	<0.001	<0.001	<0.001	<0.001	<0.001	<0.001	<0.001	<0.001	<0.001	<0.001

95% confidence intervals in brackets

* p < 0.10, ** p < 0.05, *** p < 0.01

Table 4b: Robustness check (3-level models): Link between SIA exposure and maternal health service uptake

Dependent variables: various indicators of maternal care and child survival (see right)	Main results					Year-Interaction models					Robustness checks: reporting				
	Delivery			Antenatal care		Delivery			Antenatal care		Delivery			Antenatal care	
	At home	At private facility	At public facility	No. of antenatal care visits	No. of tetanus injections	At home	At private facility	At public facility	No. of antenatal care visits	No. of tetanus injections	At home	At private facility	At public facility	No. of antenatal care visits	No. of tetanus injections
EXP_PREG	-0.014	0.001	0.012	0.025	0.012**	0.056	0.035	0.014	0.076	-0.020	-0.018**	-0.002	0.017**	0.017	0.008*
	[-0.036, 0.007]	[-0.029, 0.030]	[-0.008, 0.031]	[-0.012, 0.063]	[0.003, 0.021]	[-0.130, 0.243]	[-0.207, 0.276]	[-0.169, 0.197]	[-0.178, 0.330]	[-0.096, 0.055]	[-0.035, -0.002]	[-0.026, 0.023]	[0.001, 0.032]	[-0.013, 0.047]	[-0.000, 0.016]
EXPxYR						-0.044	-0.033	-0.036	-0.064	0.000					
[yr = 2008]						[-0.232, 0.145]	[-0.278, 0.212]	[-0.221, 0.149]	[-0.337, 0.209]	[-0.076, 0.076]					
EXPxYR						-0.087	-0.040	0.018	-0.128	0.030					
[yr = 2013]						[-0.274, 0.100]	[-0.282, 0.203]	[-0.165, 0.201]	[-0.388, 0.133]	[-0.047, 0.107]					
EXPxYR						-0.068	-0.026	-0.008	0.034	0.052					
[yr = 2018]						[-0.255, 0.120]	[-0.271, 0.219]	[-0.192, 0.176]	[-0.223, 0.292]	[-0.027, 0.132]					
MOT_ANC	-0.133***	0.048***	0.063***			-0.133***	0.048***	0.063***			-0.139***	0.052***	0.064***		
	[-0.141, -0.124]	[0.040, 0.056]	[0.056, 0.069]			[-0.141, -0.125]	[0.040, 0.056]	[0.056, 0.069]			[-0.147, -0.132]	[0.045, 0.059]	[0.058, 0.070]		
MOT_EDM	-0.062***	0.041***	0.057***	0.091***	0.029***	-0.062***	0.041***	0.057***	0.091***	0.029***	-0.063***	0.040***	0.061***	0.090***	0.029***
	[-0.077, -0.047]	[0.020, 0.061]	[0.043, 0.071]	[0.068, 0.113]	[0.020, 0.038]	[-0.077, -0.047]	[0.020, 0.061]	[0.043, 0.071]	[0.069, 0.113]	[0.020, 0.038]	[-0.076, -0.051]	[0.021, 0.058]	[0.049, 0.073]	[0.070, 0.109]	[0.021, 0.038]
MOT_EDF	-0.029***	0.024**	0.034***	0.081***	0.022***	-0.029***	0.024**	0.034***	0.081***	0.022***	-0.026***	0.017*	0.037***	0.075***	0.024***
	[-0.044, -0.014]	[0.003, 0.046]	[0.020, 0.048]	[0.062, 0.100]	[0.013, 0.030]	[-0.044, -0.014]	[0.003, 0.045]	[0.020, 0.048]	[0.062, 0.100]	[0.013, 0.030]	[-0.039, -0.014]	[-0.003, 0.036]	[0.025, 0.050]	[0.054, 0.096]	[0.017, 0.032]
MOT_AWE	-0.910***	0.232***	0.972***	1.927***	0.633***	-0.909***	0.232***	0.971***	1.908***	0.628***	-0.900***	0.236***	0.948***	1.894***	0.637***
	[-0.986, -0.835]	[0.120, 0.344]	[0.895, 1.049]	[1.682, 2.172]	[0.572, 0.694]	[-0.985, -0.834]	[0.120, 0.344]	[0.894, 1.049]	[1.662, 2.153]	[0.565, 0.690]	[-0.966, -0.834]	[0.136, 0.337]	[0.882, 1.015]	[1.665, 2.123]	[0.586, 0.688]
MOT_AGE	-0.003	-0.001	0.003	0.009***	0.000	-0.003	-0.001	0.003	0.009***	0.000	-0.001	-0.001	0.001	0.005*	0.000
	[-0.008, 0.002]	[-0.008, 0.006]	[-0.002, 0.008]	[0.003, 0.015]	[-0.002, 0.002]	[-0.008, 0.002]	[-0.008, 0.006]	[-0.002, 0.008]	[0.004, 0.015]	[-0.002, 0.002]	[-0.005, 0.004]	[-0.007, 0.005]	[-0.003, 0.005]	[-0.000, 0.011]	[-0.003, 0.002]
HH_RUR	0.528***	-0.345***	-0.335***	-0.514***	-0.142***	0.532***	-0.345***	-0.340***	-0.510***	-0.145***	0.474***	-0.309***	-0.313***	-0.511***	-0.149***
	[0.439, 0.618]	[-0.453, -0.238]	[-0.419, -0.251]	[-0.778, -0.249]	[-0.205, -0.080]	[0.442, 0.622]	[-0.453, -0.237]	[-0.424, -0.256]	[-0.776, -0.244]	[-0.209, -0.082]	[0.395, 0.554]	[-0.405, -0.213]	[-0.387, -0.239]	[-0.730, -0.293]	[-0.207, -0.091]
HH_REL (ref: Catholic): Other Christian	0.031	-0.023	0.014	-0.071	-0.031	0.030	-0.023	0.015	-0.076	-0.031	0.053	-0.085	0.055	-0.116	-0.028
	[-0.093, 0.156]	[-0.145, 0.099]	[-0.095, 0.124]	[-0.288, 0.146]	[-0.084, 0.022]	[-0.095, 0.154]	[-0.146, 0.099]	[-0.094, 0.125]	[-0.295, 0.143]	[-0.084, 0.021]	[-0.059, 0.164]	[-0.194, 0.023]	[-0.043, 0.153]	[-0.336, 0.104]	[-0.075, 0.019]
HH_REL (ref: Catholic): Islam	0.148*	-0.079	-0.021	-0.042	-0.040	0.147*	-0.079	-0.023	-0.045	-0.042	0.161**	-0.129*	0.021	-0.134	-0.054
	[-0.006, 0.301]	[-0.242, 0.084]	[-0.158, 0.116]	[-0.433, 0.349]	[-0.107, 0.027]	[-0.006, 0.301]	[-0.242, 0.084]	[-0.160, 0.114]	[-0.439, 0.348]	[-0.109, 0.025]	[0.024, 0.297]	[-0.275, 0.018]	[-0.101, 0.143]	[-0.491, 0.224]	[-0.126, 0.019]
HH_REL (ref: Catholic): Traditionalist	0.951***	-0.902***	-0.456**	-0.555**	-0.308***	0.951***	-0.904***	-0.455**	-0.567**	-0.313***	0.855***	-0.852***	-0.394**	-0.543**	-0.247***
	[0.590, 1.312]	[-1.367, -0.437]	[-0.819, -0.094]	[-1.011, -0.098]	[-0.423, -0.193]	[0.590, 1.311]	[-1.369, -0.438]	[-0.817, -0.093]	[-1.024, -0.109]	[-0.428, -0.197]	[0.523, 1.187]	[-1.284, -0.420]	[-0.729, -0.059]	[-1.031, -0.054]	[-0.375, -0.119]
HH_REL (ref: Catholic): Other	-0.389	0.218	0.409	0.037	-0.087	-0.389	0.220	0.407	0.149	-0.063	-0.306	0.306	0.305	-0.590	-0.231*
	[-1.143, 0.365]	[-1.057, 1.493]	[-0.350, 1.169]	[-1.052, 1.125]	[-0.512, 0.338]	[-1.143, 0.365]	[-1.056, 1.496]	[-0.353, 1.166]	[-0.923, 1.220]	[-0.476, 0.350]	[-0.894, 0.282]	[-0.687, 1.299]	[-0.284, 0.893]	[-1.351, 0.171]	[-0.486, 0.023]
HH_ETH (ref: Ekoi): Fulani	0.824***	-0.200	-0.744***	-0.608**	-0.280***	0.788***	-0.205	-0.704***	-0.687**	-0.256***	0.841***	-0.160	-0.785***	-0.576**	-0.282***
	[0.369, 1.279]	[-1.163, 0.764]	[-1.189, -0.300]	[-1.110, -0.106]	[-0.405, -0.155]	[0.332, 1.244]	[-1.168, 0.759]	[-1.149, -0.258]	[-1.223, -0.151]	[-0.388, -0.125]	[0.426, 1.256]	[-0.962, 0.642]	[-1.186, -0.384]	[-1.075, -0.078]	[-0.405, -0.158]
HH_ETH (ref: Ekoi): Hausa	0.979***	-0.399	-0.748***	-0.134	-0.115**	0.945***	-0.403	-0.708***	-0.211	-0.092*	0.995***	-0.610	-0.763***	-0.124	-0.124**
	[0.532, 1.426]	[-1.328, 0.531]	[-1.183, -0.313]	[-0.634, 0.366]	[-0.217, -0.013]	[0.497, 1.393]	[-1.332, 0.526]	[-1.144, -0.272]	[-0.731, 0.310]	[-0.198, 0.014]	[0.586, 1.404]	[-1.393, 0.172]	[-1.157, -0.369]	[-0.601, 0.354]	[-0.231, -0.017]
HH_ETH (ref: Ekoi): Ibibio	0.427*	0.030	-0.243	-0.170	-0.099	0.397	0.028	-0.209	-0.228	-0.075	0.596***	-0.198	-0.381*	-0.025	-0.135
	[-0.063, 0.917]	[-0.920, 0.980]	[-0.721, 0.234]	[-0.746, 0.405]	[-0.352, 0.153]	[-0.093, 0.888]	[-0.922, 0.979]	[-0.687, 0.269]	[-0.835, 0.379]	[-0.327, 0.176]	[0.144, 1.047]	[-1.009, 0.612]	[-0.817, 0.055]	[-0.417, 0.367]	[-0.329, 0.058]
HH_ETH (ref: Ekoi): Igala	0.105	0.377	-0.091	-0.114	0.288**	0.068	0.373	-0.048	-0.199	0.312**	0.086	0.108	-0.018	0.101	0.304**
	[-0.446, 0.657]	[-0.572, 1.327]	[-0.604, 0.423]	[-0.505, 0.278]	[0.044, 0.532]	[-0.484, 0.621]	[-0.577, 1.323]	[-0.562, 0.465]	[-0.610, 0.212]	[0.071, 0.554]	[-0.411, 0.582]	[-0.694, 0.909]	[-0.480, 0.444]	[-0.276, 0.477]	[0.033, 0.576]
HH_ETH (ref: Ekoi): Igbo	-0.358	0.818*	-0.431*	0.755***	0.020	-0.388*	0.816*	-0.395*	0.694***	0.044	-0.355*	0.595	-0.390*	0.772***	0.016
	[-0.813, 0.097]	[-0.078, 1.713]	[-0.862, 0.001]	[0.284, 1.225]	[-0.080, 0.120]	[-0.843, 0.068]	[-0.080, 1.711]	[-0.828, 0.037]	[0.188, 1.199]	[-0.052, 0.140]	[-0.774, 0.065]	[-0.159, 1.350]	[-0.783, 0.003]	[0.399, 1.145]	[-0.077, 0.109]
HH_ETH (ref: Ekoi): Ijaw / Izon	0.857***	-0.265	-0.705***	-0.772*	-0.083	0.829***	-0.266	-0.673***	-0.839**	-0.066	0.802***	-0.416	-0.684***	-0.731*	-0.080
	[0.336, 1.378]	[-1.253, 0.723]	[-1.212, -0.199]	[-1.550, 0.007]	[-0.259, 0.093]	[0.308, 1.351]	[-1.254, 0.722]	[-1.180, -0.167]	[-1.640, -0.038]	[-0.248, 0.115]	[0.329, 1.275]	[-1.254, 0.422]	[-1.141, -0.227]	[-1.484, 0.023]	[-0.247, 0.087]
HH_ETH (ref: Ekoi): Kanuri / Beriberi	1.104***	-0.411	-0.925***	-0.316	-0.153**	1.068***	-0.413	-0.882***	-0.410	-0.128*	0.944***	-0.240	-0.813***	-0.276	-0.169***
	[0.593, 1.616]	[-1.629, 0.808]	[-1.424, -0.426]	[-0.771, 0.138]	[-0.276, -0.030]	[0.555, 1.581]	[-1.632, 0.806]	[-1.382, -0.382]	[-0.915, 0.095]	[-0.257, 0.002]	[0.484, 1.404]	[-1.230, 0.751]	[-1.258, -0.367]	[-0.708, 0.156]	[-0.286, -0.052]
HH_ETH (ref: Ekoi): Tiv	-0.140	0.642	-0.010	-0.676***	-0.421***	-0.173	0.639	0.025	-0.750***	-0.399***	-0.082	0.460	-0.040	-0.570**	-0.358***
	[-0.647, 0.367]	[-0.304, 1.588]	[-0.511, 0.491]	[-1.181, -0.171]	[-0.581, -0.260]	[-0.681, 0.334]	[-0.307, 1.586]	[-0.477, 0.526]	[-1.281, -0.219]	[-0.564, -0.234]	[-0.549, 0.384]	[-0.343, 1.262]	[-0.493, 0.413]	[-1.059, -0.080]	[-0.492, -0.224]
HH_ETH (ref: Ekoi): Yoruba	0.331	0.348	-0.448**	1.415***	0.097*	0.298	0.346	-0.411*	1.348***	0.120**	0.327	0.103	-0.394*	1.495***	0.082
	[-0.123, 0.784]	[-0.551, 1.247]	[-0.883, -0.013]	[0.906, 1.924]	[-0.018, 0.212]	[-0.157, 0.752]	[-0.553, 1.245]	[-0.847, 0.025]	[0.827, 1.869]	[0.007, 0.232]	[-0.091, 0.745]	[-0.654, 0.860]	[-0.790, 0.003]	[0.975, 2.014]	[-0.029, 0.192]
HH_ETH (ref: Ekoi): Other	0.424*	0.273	-0.350*	0.085	0.029	0.390*	0.271	-0.312	0.011	0.053	0.434**	0.077	-0.349*	0.138	0.032
	[-0.000, 0.849]	[-0.614, 1.161]	[-0.765, 0.064]	[-0.250, 0.421]	[-0.053, 0.111]	[-0.036, 0.816]	[-0.617, 1.159]	[-0.727, 0.103]	[-0.370, 0.392]	[-0.032, 0.138]	[0.042, 0.826]	[-0.670, 0.823]	[-0.726, 0.028]	[-0.170, 0.445]	[-0.041, 0.105]
HH_SIZ	0.033***	-0.013*	-0.022***	-0.038***	-0.006***	0.033***	-0.013*	-0.022***	-0.039***	-0.006***	0.036***	-0.018***	-0.024***	-0.035***	-0.005***
	[0.023, 0.043]	[-0.028, 0.001]	[-0.031, -0.013]	[-0.054, -0.023]	[-0.010, -0.002]	[0.023, 0.043]	[-0.028, 0.001]	[-0.031, -0.012]	[-0.054, -0.023]	[-0.010, -0.002]	[0.027, 0.044]	[-0.031, -0.005]	[-0.032, -0.016]	[-0.046, -0.023]	[-0.009, -0.001]
HH_WEA	-0.210***	0.120***	0.133***	0.278***	0.057***	-0.210***	0.120***	0.132***	0.280***	0.057***	-0.211***	0.129***	0.132***	0.256***	0.051***
	[-0.227, -0.193]	[0.099, 0.141]	[0.117, 0.148]	[0.241, 0.314]	[0.047, 0.066]	[-0.227, -0.193]	[0.099, 0.141]	[0.117, 0.148]	[0.243, 0.317]	[0.048, 0.067]	[-0.225, -0.196]	[0.110, 0.147]	[0.119, 0.145]	[0.220, 0.291]	[0.044, 0.059]
YEAR (ref: 2003): 2008	0.064	-0.308***	0.077	-0.715***	-0.110**	0.153	-0.236	0.181	-0.538	-0.082	0.081	-0.304***	0.057	-0.625***	-0.099*
	[-0.114, 0.243]	[-0.514, -0.102]	[-0.096, 0.250]	[-1.142, -0.287]	[-0.213, -0.006]	[-0.338, 0.644]	[-0.812, 0.341]	[-0.295, 0.657]	[-1.384, 0.307]	[-0.295, 0.130]	[-0.083, 0.245]	[-0.499, -0.109]	[-0.102, 0.215]	[-1.057, -0.192]	[-0.207, 0.009]
YEAR (ref: 2003): 2013	0.026	-0.475***	0.136	-0.389*	-0.037	0.260	-0.384	0.064	0.051	-0.109	0.017	-0.437***	0.109	-0.254	-0.011
	[-0.152, 0.204]	[-0.678, -0.272]	[-0.035, 0.307]	[-0.827, 0.050]	[-0.150, 0.076]	[-0.228, 0.748]	[-0.956, 0.187]	[-0.408, 0.536]	[-0.779, 0.880]	[-0.306, 0.088]	[-0.146, 0.180]	[-0.629, -0.246]	[-0.047, 0.265]	[-0.686, 0.179]	[-0.130, 0.107]
YEAR (ref: 2003): 2018	-0.189**	-0.468***	0.342***	-1.296***	-0.046	-0.027	-0.408	0.362	-1.422***	-0.186*	-0.232***	-0.456***	0.363***	-1.065***	-0.012
	[-0.374, -0.005]	[-0.684, -0.252]	[0.165, 0.519]	[-1.916, -0.676]	[-0.207, 0.115]	[-0.518, 0.464]	[-0.983, 0.167]	[-0.112, 0.836]	[-2.332, -0.511]	[-0.402, 0.029]	[-0.393, -0.072]	[-0.650, -0.263]	[0.207, 0.518]	[-1.660, -0.471]	[-0.174, 0.150]
Constant	1.852***	-3.405***	-2.482***	3.574***	0.910***	1.715***	-3.476***	-2.522***	3.511***	0.966***	1.834***	-3.202***	-2.497***	3.689***	0.929***
	[1.322, 2.382]	[-4.464, -2.346]	[-2.980, -1.984]	[2.722, 4.426]	[0.764, 1.055]	[1.024, 2.407]	[-4.657, -2.294]	[-3.182, -1.861]	[2.422, 4.599]	[0.730, 1.201]	[1.340, 2.328]	[-4.134, -2.269]	[-2.953, -2.040]	[2.816, 4.561]	[0.774, 1.085]
Multilevel variance parameter: Level 1	0.353***	2.194***	0.151***	0.731***	-1.488***	0.351***	2.191***	0.151***	0.722***	-1.522***	0.380***	2.175***	0.172***	0.698***	-1.534***
	[0.170, 0.536]	[1.041, 3.348]	[0.067, 0.235]	[0.524, 0.938]	[-1.757, -1.219]	[0.169, 0.534]	[1.038, 3.343]	[0.067, 0.236]	[0.515, 0.929]	[-1.797, -1.246]	[0.188, 0.571]	[1.070, 3.279]	[0.080, 0.264]	[0.491, 0.905]	[-1.791, -1.277]
Multilevel variance parameter: Level 2	0.256***	0.255***	0.337***	-0.047	-1.653***	0.254***	0.255***	0.334***	-0.057	-1.679***	0.266***	0.222***	0.321***	-0.073	-1.615***
	[0.197, 0.316]	[0.183, 0.328]	[0.271, 0.403]	[-0.276, 0.181]	[-1.789, -1.517]	[0.195, 0.314]	[0.183, 0.328]	[0.269, 0.400]	[-0.287, 0.173]	[-1.815, -1.542]	[0.211, 0.322]	[0.162, 0.282]	[0.263, 0.378]	[-0.297, 0.151]	[-1.740, -1.490]
Multilevel variance parameter: Residual				1.320***	0.088***				1.319***	0.088***				1.279***	0.083***
				[1.190, 1.449]	[0.040, 0.137]				[1.190, 1.448]	[0.040, 0.137]				[1.150, 1.409]	[0.038, 0.129]
Level 1 Observations (child)	34713	34713	34713	35019	36585	34713	34713	34713	35019	36585	45437	45437	45437	45839	47644
Level 2 Observations (LGA)	757	757	757	757	758	757	757	757	757	758	761	761	761	761	762
Level 3 Observations (state)	37	37	37	37	37	37	37	37	37	37	37	37	37	37	37
Akaike Information Criterion	26186.547	17119.846	30165.878	192900.905	111038.932	26188.211	17125.583	30165.157	192869.798	111007.527	34345.877	21317.031	39462.966	248646.722	144047.955
Prob. > X^2	<0.001	<0.001	<0.001	<0.001	<0.001	<0.001	<0.001	<0.001	<0.001	<0.001	<0.001	<0.001	<0.001	<0.001	<0.001

95% confidence intervals in brackets

* p < 0.10, ** p < 0.05, *** p < 0.01

Table 5a: Detailed results: Link between SIA exposure and child survival

Dependent variable: Child survival	Main results		
	Full model		Year-Interaction
	Exposure decomposition	Total exposure	Child survival (total exposure)
EXP_PREG_nod (date approximation)	-0.012 [-0.034, 0.010]		
EXP_CHI_nod (date approximation)	-0.019*** [-0.029, -0.009]		
EXP_TOT_nod (total exposure, date approximation)		-0.018*** [-0.027, -0.009]	-0.058 [-0.134, 0.019]
EXPxYR [yr = 2008]			0.031 [-0.044, 0.107]
EXPxYR [yr = 2013]			0.046 [-0.030, 0.121]
EXPxYR [yr = 2018]			0.031 [-0.045, 0.106]
CHI_AGE	-0.005 [-0.014, 0.005]	-0.005 [-0.014, 0.005]	-0.002 [-0.012, 0.008]
CHI_AGE2	0.000 [-0.000, 0.000]	0.000 [-0.000, 0.000]	0.000 [-0.000, 0.000]
CHI_ORD	-0.154*** [-0.176, -0.131]	-0.154*** [-0.176, -0.131]	-0.153*** [-0.176, -0.131]
CHI_SEX	0.213*** [0.141, 0.285]	0.213*** [0.141, 0.285]	0.213*** [0.141, 0.285]
MOT_ANC	-0.001 [-0.011, 0.009]	-0.001 [-0.011, 0.009]	-0.001 [-0.011, 0.009]
MOT_EDM	0.013 [-0.006, 0.032]	0.013 [-0.006, 0.032]	0.013 [-0.006, 0.032]
MOT_EDF	0.028*** [0.011, 0.045]	0.028*** [0.011, 0.045]	0.028*** [0.010, 0.045]
MOT_AGE	0.010** [0.002, 0.018]	0.010** [0.002, 0.018]	0.010** [0.001, 0.018]
HH_RUR	-0.182*** [-0.284, -0.079]	-0.181*** [-0.284, -0.079]	-0.184*** [-0.286, -0.081]
HH_REL (ref: Catholic): Other Christian	-0.137 [-0.303, 0.028]	-0.138 [-0.303, 0.028]	-0.136 [-0.302, 0.029]
HH_REL (ref: Catholic): Islam	-0.072 [-0.267, 0.123]	-0.070 [-0.265, 0.124]	-0.065 [-0.260, 0.130]
HH_REL (ref: Catholic): Traditionalist	-0.092 [-0.464, 0.280]	-0.091 [-0.463, 0.281]	-0.099 [-0.471, 0.273]
HH_REL (ref: Catholic): Other	0.584 [-0.590, 1.757]	0.581 [-0.592, 1.755]	0.558 [-0.616, 1.732]
HH_ETH (ref: Ekoi): Fulani	-0.182 [-0.808, 0.444]	-0.178 [-0.803, 0.448]	-0.118 [-0.745, 0.510]
HH_ETH (ref: Ekoi): Hausa	-0.502 [-1.120, 0.116]	-0.497 [-1.114, 0.121]	-0.436 [-1.056, 0.183]
HH_ETH (ref: Ekoi): Ibibio	-0.457 [-1.138, 0.225]	-0.458 [-1.140, 0.223]	-0.422 [-1.104, 0.259]
HH_ETH (ref: Ekoi): Igala	-0.390 [-1.102, 0.322]	-0.389 [-1.101, 0.323]	-0.357 [-1.070, 0.355]
HH_ETH (ref: Ekoi): Igbo	-0.500 [-1.109, 0.109]	-0.501 [-1.110, 0.108]	-0.471 [-1.081, 0.139]
HH_ETH (ref: Ekoi): Ijaw / Izon	-0.242 [-0.896, 0.413]	-0.243 [-0.898, 0.412]	-0.204 [-0.859, 0.451]
HH_ETH (ref: Ekoi): Kanuri / Beriberi	-0.269 [-0.928, 0.389]	-0.264 [-0.922, 0.395]	-0.189 [-0.850, 0.472]
HH_ETH (ref: Ekoi): Tiv	-0.362 [-1.013, 0.290]	-0.359 [-1.010, 0.293]	-0.312 [-0.964, 0.341]
HH_ETH (ref: Ekoi): Yoruba	-0.021 [-0.642, 0.601]	-0.021 [-0.643, 0.600]	0.011 [-0.611, 0.633]
HH_ETH (ref: Ekoi): Other	-0.354 [-0.959, 0.251]	-0.351 [-0.956, 0.254]	-0.298 [-0.905, 0.308]
HH_SIZ	0.132*** [0.117, 0.146]	0.132*** [0.117, 0.146]	0.132*** [0.118, 0.146]
HH_WEA	0.017* [-0.003, 0.037]	0.017 [-0.003, 0.037]	0.016 [-0.004, 0.036]
YEAR (ref: 2003): 2008	0.330*** [0.136, 0.524]	0.334*** [0.141, 0.528]	0.208 [-0.263, 0.680]
YEAR (ref: 2003): 2013	0.489*** [0.290, 0.689]	0.493*** [0.295, 0.692]	0.161 [-0.308, 0.629]
YEAR (ref: 2003): 2018	0.302*** [0.110, 0.494]	0.309*** [0.119, 0.500]	0.164 [-0.304, 0.633]
Constant	2.494*** [1.819, 3.169]	2.502*** [1.827, 3.177]	2.644*** [1.859, 3.428]
Multilevel variance parameter: Level 1	0.041*** [0.014, 0.068]	0.041*** [0.014, 0.068]	0.040*** [0.013, 0.067]
Level 1 Observations (child)	52431	52431	52431
Level 2 Observations (LGA)	689	689	689
Akaike Information Criterion	23684.534	23682.853	23676.199
Prob. > X^2	<0.001	<0.001	<0.001

95% confidence intervals in brackets

* p < 0.10, ** p < 0.05, *** p < 0.01

Table 5b: Robustness check (3-level models): Link between SIA exposure and child survival

Dependent variables: various indicators of maternal care and child survival (see right)	Main results		
	Full model		Year-Interaction
	Exposure decomposition	Total exposure	Child survival (total exposure)
EXP_PREG_nod (date approximation)	-0.013 [-0.035, 0.010]		
EXP_CHI_nod (date approximation)	-0.020*** [-0.031, -0.010]		
EXP_TOT_nod (total exposure, date approximation)		-0.019*** [-0.029, -0.009]	-0.062 [-0.140, 0.015]
EXPxYR [yr = 2008]			0.033 [-0.042, 0.109]
EXPxYR [yr = 2013]			0.048 [-0.027, 0.124]
EXPxYR [yr = 2018]			0.034 [-0.042, 0.110]
CHI_AGE	-0.004 [-0.014, 0.006]	-0.004 [-0.013, 0.006]	-0.001 [-0.011, 0.009]
CHI_AGE2	0.000 [-0.000, 0.000]	0.000 [-0.000, 0.000]	0.000 [-0.000, 0.000]
CHI_ORD	-0.154*** [-0.177, -0.132]	-0.154*** [-0.177, -0.132]	-0.154*** [-0.176, -0.131]
CHI_SEX	0.212*** [0.140, 0.284]	0.212*** [0.140, 0.284]	0.212*** [0.140, 0.284]
MOT_ANC	0.000 [-0.010, 0.010]	0.000 [-0.010, 0.010]	0.000 [-0.010, 0.010]
MOT_EDM	0.014 [-0.005, 0.033]	0.014 [-0.005, 0.033]	0.014 [-0.005, 0.033]
MOT_EDF	0.029*** [0.012, 0.046]	0.029*** [0.012, 0.046]	0.029*** [0.012, 0.046]
MOT_AGE	0.010** [0.002, 0.019]	0.010** [0.002, 0.019]	0.010** [0.002, 0.019]
HH_RUR	-0.176*** [-0.279, -0.073]	-0.176*** [-0.279, -0.073]	-0.178*** [-0.281, -0.075]
HH_REL (ref: Catholic): Other Christian	-0.128 [-0.296, 0.039]	-0.129 [-0.296, 0.038]	-0.127 [-0.295, 0.040]
HH_REL (ref: Catholic): Islam	-0.089 [-0.288, 0.109]	-0.088 [-0.286, 0.110]	-0.083 [-0.282, 0.115]
HH_REL (ref: Catholic): Traditionalist	-0.055 [-0.428, 0.319]	-0.054 [-0.428, 0.320]	-0.060 [-0.434, 0.314]
HH_REL (ref: Catholic): Other	0.639 [-0.535, 1.813]	0.636 [-0.538, 1.809]	0.613 [-0.561, 1.788]
HH_ETH (ref: Ekoi): Fulani	-0.001 [-0.665, 0.663]	0.003 [-0.662, 0.667]	0.071 [-0.595, 0.737]
HH_ETH (ref: Ekoi): Hausa	-0.349 [-1.006, 0.308]	-0.345 [-1.002, 0.312]	-0.276 [-0.935, 0.384]
HH_ETH (ref: Ekoi): Ibibio	-0.347 [-1.064, 0.370]	-0.349 [-1.066, 0.368]	-0.304 [-1.022, 0.415]
HH_ETH (ref: Ekoi): Igala	-0.203 [-0.969, 0.563]	-0.202 [-0.968, 0.564]	-0.153 [-0.921, 0.614]
HH_ETH (ref: Ekoi): Igbo	-0.353 [-1.010, 0.305]	-0.355 [-1.012, 0.303]	-0.316 [-0.975, 0.342]
HH_ETH (ref: Ekoi): Ijaw / Izon	-0.056 [-0.773, 0.660]	-0.058 [-0.775, 0.658]	-0.011 [-0.728, 0.707]
HH_ETH (ref: Ekoi): Kanuri / Beriberi	-0.230 [-0.925, 0.464]	-0.225 [-0.920, 0.469]	-0.146 [-0.843, 0.551]
HH_ETH (ref: Ekoi): Tiv	-0.200 [-0.911, 0.511]	-0.198 [-0.909, 0.513]	-0.142 [-0.855, 0.571]
HH_ETH (ref: Ekoi): Yoruba	0.047 [-0.616, 0.709]	0.046 [-0.617, 0.709]	0.087 [-0.577, 0.751]
HH_ETH (ref: Ekoi): Other	-0.217 [-0.861, 0.426]	-0.215 [-0.859, 0.428]	-0.153 [-0.798, 0.493]
HH_SIZ	0.132*** [0.118, 0.146]	0.132*** [0.118, 0.146]	0.133*** [0.118, 0.147]
HH_WEA	0.014 [-0.006, 0.035]	0.014 [-0.006, 0.034]	0.014 [-0.007, 0.034]
YEAR (ref: 2003): 2008	0.326*** [0.133, 0.519]	0.331*** [0.139, 0.523]	0.204 [-0.266, 0.674]
YEAR (ref: 2003): 2013	0.495*** [0.295, 0.694]	0.500*** [0.301, 0.699]	0.155 [-0.312, 0.623]
YEAR (ref: 2003): 2018	0.297*** [0.106, 0.488]	0.305*** [0.115, 0.494]	0.142 [-0.326, 0.611]
Constant	2.353*** [1.641, 3.065]	2.362*** [1.650, 3.074]	2.505*** [1.689, 3.322]
Multilevel variance parameter: Level 1	0.026** [0.005, 0.047]	0.026** [0.005, 0.047]	0.027** [0.005, 0.048]
Multilevel variance parameter: Level 2	0.020* [-0.004, 0.044]	0.020 [-0.004, 0.044]	0.019 [-0.005, 0.042]
Level 1 Observations (child)	52431	52431	52431
Level 2 Observations (LGA)	761	761	761
Level 3 Observations (state)	37	37	37
Akaike Information Criterion	23665.091	23663.461	23656.424
Prob. > X^2	<0.001	<0.001	<0.001

95% confidence intervals in brackets

* p < 0.10, ** p < 0.05, *** p < 0.01

Table 6a: Additional robustness checks: exclusion of 2018 survey round to compensate for mop-up variability

Dependent variables: various indicators of routine immunisation, maternal care and child survival (see right)	**Non-polio full immunisation status**		**Delivery**			**Antenatal care**		**Child survival**
	Full model	Interaction model	At home	At private facility	At public facility	No. of antenatal care	No. of tetanus injections	Total exposure
EXP_CHI	-0.030***	0.011						
	[-0.044,-0.016]	[-0.015,0.036]						
EXPxAGE		-0.001***						
		[-0.001,-0.000]						
EXP_PREG			0.006	-0.057***	0.004	-0.020	-0.003	
			[-0.020,0.032]	[-0.091,-0.024]	[-0.020,0.029]	[-0.060,0.021]	[-0.014,0.007]	
EXP_TOT_nod (total exposure, date approximation)								-0.013**
								[-0.024,-0.001]
CHI_AGE	0.033***	0.038***						-0.002
	[0.026,0.039]	[0.031,0.045]						[-0.014,0.009]
CHI_AGE2								0.000
								[-0.000,0.000]
CHI_ORD	-0.060***	-0.061***						-0.143***
	[-0.088,-0.033]	[-0.088,-0.034]						[-0.170,-0.116]
CHI_SEX	-0.007	-0.006						0.245***
	[-0.090,0.077]	[-0.089,0.078]						[0.157,0.334]
MOT_ANC	0.053***	0.053***	-0.133***	0.056***	0.061***			0.002
	[0.043,0.062]	[0.044,0.062]	[-0.142,-0.125]	[0.047,0.064]	[0.054,0.068]			[-0.010,0.013]
MOT_EDM	0.069***	0.069***	-0.064***	0.057***	0.053***	0.105***	0.031***	0.018
	[0.049,0.089]	[0.049,0.089]	[-0.081,-0.048]	[0.035,0.080]	[0.038,0.069]	[0.079,0.130]	[0.024,0.038]	[-0.006,0.041]
MOT_EDF	0.006	0.007	-0.028***	0.036***	0.031***	0.090***	0.022***	0.024**
	[-0.014,0.027]	[-0.013,0.028]	[-0.045,-0.012]	[0.012,0.059]	[0.015,0.047]	[0.068,0.112]	[0.015,0.029]	[0.002,0.045]
MOT_AWE	2.955***	2.964***	-0.947***	0.289***	1.021***	1.900***	0.631***	
	[2.821,3.089]	[2.829,3.098]	[-1.029,-0.865]	[0.166,0.411]	[0.936,1.106]	[1.754,2.047]	[0.584,0.677]	
MOT_AGE	0.025***	0.026***	-0.002	0.001	0.002	0.014***	0.002	0.010**
	[0.016,0.035]	[0.016,0.035]	[-0.007,0.004]	[-0.007,0.008]	[-0.003,0.007]	[0.007,0.020]	[-0.000,0.004]	[0.000,0.020]
HH_RUR	-0.074	-0.076	0.599***	-0.402***	-0.364***	-0.568***	-0.168***	-0.266***
	[-0.191,0.044]	[-0.194,0.042]	[0.498,0.701]	[-0.526,-0.278]	[-0.458,-0.270]	[-0.836,-0.301]	[-0.226,-0.111]	[-0.395,-0.137]
HH_REL (ref: Catholic): Other Christian	-0.172**	-0.172**	0.106	-0.042	-0.050	-0.102	-0.037	-0.050
	[-0.329,-0.015]	[-0.330,-0.015]	[-0.029,0.242]	[-0.180,0.096]	[-0.172,0.071]	[-0.348,0.144]	[-0.096,0.022]	[-0.245,0.144]
HH_REL (ref: Catholic): Islam	-0.504***	-0.522***	0.205**	-0.218**	-0.035	-0.177	-0.109***	0.025
	[-0.695,-0.313]	[-0.714,-0.330]	[0.040,0.369]	[-0.397,-0.040]	[-0.184,0.113]	[-0.508,0.154]	[-0.189,-0.029]	[-0.204,0.254]
HH_REL (ref: Catholic): Traditionalist	-0.596***	-0.600***	1.053***	-0.863***	-0.553***	-0.589**	-0.360***	-0.069
	[-1.025,-0.168]	[-1.029,-0.171]	[0.680,1.426]	[-1.347,-0.380]	[-0.936,-0.171]	[-1.087,-0.091]	[-0.498,-0.221]	[-0.457,0.318]
HH_REL (ref: Catholic): Other	-0.733	-0.725	-0.555	0.570	0.388	0.096	0.022	-0.324
	[-2.533,1.067]	[-2.524,1.075]	[-1.634,0.523]	[-0.794,1.933]	[-0.744,1.520]	[-2.104,2.295]	[-0.697,0.741]	[-1.551,0.903]
HH_ETH (ref: Ekoi): Fulani	-1.040***	-1.092***	0.671***	-0.058	-0.744***	-0.764	-0.383***	-0.447
	[-1.596,-0.484]	[-1.650,-0.535]	[0.201,1.141]	[-1.098,0.981]	[-1.207,-0.280]	[-2.268,0.740]	[-0.655,-0.110]	[-1.256,0.362]
HH_ETH (ref: Ekoi): Hausa	-0.926***	-1.012***	0.936***	-0.569	-0.806***	-0.504	-0.293**	-0.832**
	[-1.459,-0.393]	[-1.547,-0.477]	[0.481,1.391]	[-1.557,0.419]	[-1.254,-0.357]	[-2.005,0.996]	[-0.563,-0.023]	[-1.632,-0.032]
HH_ETH (ref: Ekoi): Ibibio	-0.639**	-0.643**	0.296	0.750	-0.263	-0.026	-0.163	-0.777*
	[-1.224,-0.054]	[-1.229,-0.057]	[-0.220,0.811]	[-0.270,1.771]	[-0.767,0.242]	[-1.484,1.433]	[-0.483,0.156]	[-1.650,0.095]
HH_ETH (ref: Ekoi): Igala	-0.699**	-0.704**	-0.532*	1.440***	0.023	-0.135	0.284*	-0.670
	[-1.337,-0.061]	[-1.342,-0.065]	[-1.106,0.043]	[0.423,2.456]	[-0.509,0.556]	[-1.689,1.418]	[-0.046,0.615]	[-1.584,0.243]
HH_ETH (ref: Ekoi): Igbo	-0.542**	-0.546**	-0.852***	2.114***	-0.601***	1.096	0.078	-0.917**
	[-1.050,-0.035]	[-1.054,-0.037]	[-1.301,-0.403]	[1.165,3.064]	[-1.039,-0.162]	[-0.409,2.602]	[-0.184,0.340]	[-1.704,-0.130]
HH_ETH (ref: Ekoi): Ijaw / Izon	-0.654**	-0.651**	0.819***	0.238	-0.751***	-0.915	-0.150	-0.766*
	[-1.242,-0.066]	[-1.240,-0.061]	[0.289,1.350]	[-0.817,1.292]	[-1.271,-0.231]	[-2.502,0.672]	[-0.444,0.145]	[-1.592,0.059]
HH_ETH (ref: Ekoi): Kanuri / Beriberi	-1.146***	-1.232***	1.027***	-0.564	-0.954***	-0.626	-0.286*	-0.628
	[-1.819,-0.474]	[-1.906,-0.557]	[0.493,1.560]	[-1.886,0.759]	[-1.477,-0.431]	[-2.148,0.897]	[-0.579,0.007]	[-1.471,0.215]
	-0.721**	-0.734**	-0.803***	1.933***	0.111	-1.066	-0.392**	-0.883**

HH_ETH (ref: Ekoi): Tiv	[-1.334,-0.108]	[-1.347,-0.120]	[-1.314,-0.292]	[0.928,2.937]	[-0.398,0.621]	[-2.636,0.504]	[-0.696,-0.088]	[-1.710,-0.056]
HH_ETH (ref: Ekoi): Yoruba	-0.650**	-0.660**	-0.172	1.441***	-0.422*	2.185***	0.061	-0.429
	[-1.168,-0.132]	[-1.179,-0.141]	[-0.628,0.283]	[0.485,2.397]	[-0.866,0.022]	[0.668,3.702]	[-0.204,0.327]	[-1.231,0.372]
HH_ETH (ref: Ekoi): Other	-0.727***	-0.759***	0.196	1.015**	-0.322	-0.039	-0.054	-0.723*
	[-1.226,-0.228]	[-1.260,-0.259]	[-0.238,0.630]	[0.066,1.963]	[-0.750,0.105]	[-1.520,1.442]	[-0.317,0.209]	[-1.506,0.060]
HH_SIZ	-0.015**	-0.015**	0.030***	-0.013	-0.019***	-0.049***	-0.008***	0.135***
	[-0.029,-0.001]	[-0.029,-0.001]	[0.019,0.041]	[-0.029,0.003]	[-0.030,-0.009]	[-0.064,-0.035]	[-0.013,-0.004]	[0.117,0.153]
HH_WEA	0.155***	0.156***	-0.213***	0.116***	0.138***	0.318***	0.066***	0.012
	[0.133,0.178]	[0.133,0.179]	[-0.232,-0.194]	[0.092,0.140]	[0.120,0.155]	[0.282,0.353]	[0.057,0.076]	[-0.013,0.038]
YEAR (ref: 2003): 2008	0.667***	0.601***	0.001	-0.246**	0.081	-0.658***	-0.098**	0.320***
	[0.402,0.932]	[0.333,0.869]	[-0.185,0.188]	[-0.462,-0.031]	[-0.097,0.259]	[-1.073,-0.242]	[-0.184,-0.013]	[0.123,0.517]
YEAR (ref: 2003): 2013	0.801***	0.720***	-0.030	-0.406***	0.146	-0.338	-0.036	0.464***
	[0.539,1.064]	[0.453,0.986]	[-0.216,0.156]	[-0.618,-0.194]	[-0.030,0.322]	[-0.769,0.094]	[-0.123,0.052]	[0.258,0.670]
Constant	-4.617***	-4.787***	2.050***	-3.869***	-2.399***	3.518***	1.007***	2.723***
	[-5.246,-3.989]	[-5.423,-4.151]	[1.540,2.561]	[-4.878,-2.861]	[-2.900,-1.897]	[1.918,5.118]	[0.716,1.299]	[1.871,3.576]
Multilevel variance parameter: Level 1	0.349***	0.348***	0.478***	0.789***	0.499***	0.793***	-1.384***	0.041**
	[0.265,0.434]	[0.264,0.432]	[0.381,0.576]	[0.603,0.975]	[0.407,0.590]	[0.696,0.890]	[-1.483,-1.285]	[0.007,0.075]
Multilevel variance parameter: Residual						1.333***	0.092***	
						[1.291,1.376]	[0.064,0.121]	
Level 1 Observations (child)	20752	20752	29480	29480	29480	29691	31177	34500
Level 2 Observations (LGA)	572	572	574	574	574	574	575	576
Akaike Information Criterion	14663.935	14651.865	21880.73	14443.993	24935.19	164998.875	94979.021	15648.893
Prob. > X^2	<0.001	<0.001	<0.001	<0.001	<0.001	<0.001	<0.001	<0.001

95% confidence intervals in brackets
* p < 0.10, ** p < 0.05, *** p < 0.01

Table 6b-1: Additional robustness checks: year-wise stratification to detect time trends - Polio

Dependent variable: non-polio full immunisation status	**Full model**				**Interaction model (EXPxAGE)**			
	2003	2008	2013	2018	2003	2008	2013	2018
EXP_CHI	-0.040	-0.051***	-0.026***	-0.027	-0.086	-0.042	0.027*	0.111*
	[-0.289,0.209]	[-0.079,-0.023]	[-0.043,-0.010]	[-0.065,0.010]	[-1.046,0.874]	[-0.093,0.009]	[-0.003,0.058]	[-0.012,0.233]
EXPxAGE					0.003	0.000	-0.001***	-0.005**
					[-0.051,0.057]	[-0.001,0.001]	[-0.002,-0.001]	[-0.009,-0.001]
CHI_AGE	0.117**	0.035***	0.035***	0.036***	0.109	0.036***	0.043***	0.042***
	[0.017,0.218]	[0.025,0.046]	[0.026,0.043]	[0.016,0.056]	[-0.087,0.305]	[0.024,0.049]	[0.033,0.052]	[0.021,0.062]
CHI_ORD	0.018	-0.037*	-0.076***	-0.069**	0.018	-0.037*	-0.076***	-0.070**
	[-0.153,0.188]	[-0.080,0.007]	[-0.113,-0.039]	[-0.129,-0.008]	[-0.153,0.188]	[-0.081,0.006]	[-0.113,-0.039]	[-0.131,-0.009]
CHI_SEX	0.031	0.010	-0.027	0.030	0.031	0.010	-0.025	0.029
	[-0.464,0.526]	[-0.126,0.145]	[-0.137,0.083]	[-0.146,0.206]	[-0.464,0.526]	[-0.126,0.145]	[-0.135,0.085]	[-0.147,0.205]
MOT_ANC	0.095***	0.061***	0.044***	0.074***	0.094***	0.061***	0.045***	0.075***
	[0.045,0.144]	[0.045,0.077]	[0.032,0.056]	[0.049,0.100]	[0.044,0.144]	[0.045,0.077]	[0.032,0.057]	[0.050,0.101]
MOT_EDM	0.074	0.064***	0.075***	0.041*	0.074	0.064***	0.076***	0.044*
	[-0.036,0.185]	[0.031,0.096]	[0.048,0.101]	[-0.003,0.085]	[-0.036,0.185]	[0.031,0.096]	[0.049,0.103]	[-0.000,0.088]
MOT_EDF	-0.035	0.026	-0.009	0.028	-0.035	0.026	-0.007	0.029
	[-0.159,0.088]	[-0.007,0.059]	[-0.037,0.018]	[-0.015,0.071]	[-0.159,0.088]	[-0.007,0.059]	[-0.035,0.020]	[-0.014,0.072]
MOT_AWE	2.968***	2.453***	3.411***	3.131***	2.971***	2.455***	3.422***	3.132***
	[1.880,4.056]	[2.255,2.650]	[3.217,3.605]	[2.779,3.484]	[1.881,4.061]	[2.257,2.652]	[3.228,3.615]	[2.779,3.484]
MOT_AGE	0.012	0.021***	0.027***	0.033***	0.012	0.021***	0.027***	0.034***
	[-0.046,0.070]	[0.006,0.037]	[0.014,0.040]	[0.012,0.054]	[-0.046,0.070]	[0.006,0.037]	[0.015,0.040]	[0.013,0.055]
HH_RUR	-0.056	-0.031	-0.168**	-0.232*	-0.054	-0.030	-0.176**	-0.235**
	[-0.680,0.569]	[-0.244,0.181]	[-0.321,-0.015]	[-0.467,0.002]	[-0.680,0.571]	[-0.243,0.182]	[-0.329,-0.022]	[-0.469,-0.001]
HH_REL (ref: Catholic): Other Christian	-0.189	-0.269**	-0.099	0.011	-0.188	-0.270**	-0.099	0.018
	[-0.895,0.517]	[-0.532,-0.007]	[-0.310,0.112]	[-0.320,0.342]	[-0.894,0.518]	[-0.532,-0.007]	[-0.311,0.112]	[-0.313,0.349]
HH_REL (ref: Catholic): Islam	-0.258	-0.540***	-0.474***	-0.432**	-0.257	-0.543***	-0.504***	-0.438**
	[-1.035,0.519]	[-0.864,-0.216]	[-0.729,-0.218]	[-0.848,-0.015]	[-1.034,0.520]	[-0.868,-0.219]	[-0.760,-0.248]	[-0.854,-0.022]
HH_REL (ref: Catholic): Traditionalist	-0.325	-0.481	-0.757**	-0.168	-0.322	-0.481	-0.774**	-0.105
	[-2.215,1.565]	[-1.089,0.126]	[-1.411,-0.104]	[-1.807,1.470]	[-2.212,1.568]	[-1.089,0.127]	[-1.428,-0.119]	[-1.755,1.545]
HH_REL (ref: Catholic): Other	0.000	-0.531	0.000	0.162	0.000	-0.526	0.000	0.191
	[0.000,0.000]	[-2.432,1.370]	[0.000,0.000]	[-1.319,1.642]	[0.000,0.000]	[-2.428,1.377]	[0.000,0.000]	[-1.290,1.671]
HH_ETH (ref: Ekoi): Fulani	0.000	-1.277***	0.058	-0.712	0.000	-1.289***	0.034	-0.771
	[0.000,0.000]	[-2.032,-0.522]	[-1.523,1.640]	[-2.074,0.650]	[0.000,0.000]	[-2.046,-0.531]	[-1.549,1.617]	[-2.133,0.591]
HH_ETH (ref: Ekoi): Hausa	-0.297	-1.227***	0.249	-0.635	-0.287	-1.245***	0.170	-0.691
	[-2.094,1.500]	[-1.956,-0.498]	[-1.314,1.812]	[-1.952,0.682]	[-2.098,1.524]	[-1.978,-0.511]	[-1.395,1.734]	[-2.008,0.626]
HH_ETH (ref: Ekoi): Ibibio	0.000	-0.898**	0.426	-0.712	0.000	-0.899**	0.469	-0.721
	[0.000,0.000]	[-1.695,-0.101]	[-1.174,2.027]	[-2.124,0.700]	[0.000,0.000]	[-1.697,-0.101]	[-1.134,2.071]	[-2.133,0.690]
HH_ETH (ref: Ekoi): Igala	-0.403	-1.279***	0.926	-1.062	-0.399	-1.284***	0.987	-1.073
	[-2.844,2.038]	[-2.159,-0.399]	[-0.719,2.571]	[-2.652,0.528]	[-2.844,2.046]	[-2.165,-0.404]	[-0.660,2.633]	[-2.664,0.519]
HH_ETH (ref: Ekoi): Igbo	0.510	-0.827**	0.569	-0.415	0.512	-0.828**	0.611	-0.433
	[-1.416,2.435]	[-1.481,-0.173]	[-0.981,2.120]	[-1.695,0.865]	[-1.418,2.442]	[-1.482,-0.174]	[-0.941,2.164]	[-1.712,0.847]
HH_ETH (ref: Ekoi): Ijaw / Izon	0.219	-1.426***	0.894	0.126	0.212	-1.425***	0.943	0.110
	[-2.879,3.317]	[-2.240,-0.611]	[-0.696,2.484]	[-1.326,1.578]	[-2.892,3.316]	[-2.239,-0.610]	[-0.649,2.535]	[-1.342,1.562]
HH_ETH (ref: Ekoi): Kanuri / Beriberi	0.000	-1.606***	0.072	-0.521	0.000	-1.622***	-0.019	-0.615
	[0.000,0.000]	[-2.589,-0.624]	[-1.584,1.727]	[-2.060,1.018]	[0.000,0.000]	[-2.607,-0.636]	[-1.677,1.640]	[-2.154,0.925]
HH_ETH (ref: Ekoi): Tiv	0.636	-0.966**	0.299	-0.453	0.640	-0.972**	0.347	-0.511
	[-1.631,2.903]	[-1.777,-0.155]	[-1.325,1.924]	[-1.861,0.955]	[-1.632,2.912]	[-1.784,-0.161]	[-1.280,1.973]	[-1.920,0.897]
HH_ETH (ref: Ekoi): Yoruba	1.185	-0.572*	0.193	-0.550	1.185	-0.576*	0.228	-0.566
	[-0.686,3.055]	[-1.252,0.108]	[-1.360,1.746]	[-1.852,0.753]	[-0.689,3.060]	[-1.256,0.105]	[-1.327,1.783]	[-1.868,0.736]
HH_ETH (ref: Ekoi): Other	0.094	-0.999***	0.430	-0.390	0.098	-1.008***	0.438	-0.448
	[-1.703,1.892]	[-1.651,-0.347]	[-1.113,1.973]	[-1.670,0.890]	[-1.705,1.902]	[-1.661,-0.354]	[-1.107,1.982]	[-1.728,0.832]
HH_SIZ	0.005	-0.026**	-0.009	-0.012	0.005	-0.026**	-0.008	-0.012
	[-0.080,0.090]	[-0.049,-0.004]	[-0.027,0.010]	[-0.043,0.019]	[-0.080,0.090]	[-0.049,-0.004]	[-0.027,0.011]	[-0.043,0.018]
HH_WEA	0.083	0.170***	0.156***	0.099***	0.083	0.170***	0.157***	0.101***
	[-0.035,0.201]	[0.130,0.209]	[0.127,0.185]	[0.056,0.142]	[-0.035,0.201]	[0.131,0.209]	[0.127,0.186]	[0.058,0.145]
Constant	-7.031***	-3.237***	-5.320***	-4.308***	-6.896***	-3.286***	-5.704***	-4.474***
	[-9.848,-4.214]	[-4.034,-2.439]	[-6.931,-3.709]	[-5.802,-2.814]	[-10.781,-3.011]	[-4.117,-2.454]	[-7.327,-4.081]	[-5.974,-2.973]
Multilevel variance parameter: Level 1	0.000	0.520***	0.295***	0.398***	0.000	0.520***	0.292***	0.396***
	[-0.000,0.000]	[0.347,0.692]	[0.198,0.392]	[0.200,0.596]	[-0.000,0.000]	[0.347,0.692]	[0.196,0.389]	[0.198,0.593]
Level 1 Observations (child)	648	8163	11924	3629	648	8163	11924	3629
Level 2 Observations (LGA)	138	369	370	504	138	369	370	504
Akaike Information Criterion	450.792	5772.355	8366.43	3393.899	452.782	5774.188	8351.201	3390.504
Prob. > X^2	<0.001	<0.001	<0.001	<0.001	<0.001	<0.001	<0.001	<0.001

95% confidence intervals in brackets
* p < 0.10, ** p < 0.05, *** p < 0.01

Table 6b-2: Additional robustness checks: year-wise stratification to detect time trends - delivery

Dependent variables: various indicators of delivery (see right)	**At home**				**At private facility**				**At public facility**			
	2003	2008	2013	2018	2003	2008	2013	2018	2003	2008	2013	2018
EXP_PREG	0.104<br>[-0.113,0.320]	0.004<br>[-0.042,0.050]	0.019<br>[-0.014,0.051]	0.023<br>[-0.012,0.058]	-0.138<br>[-0.391,0.116]	-0.065**<br>[-0.123,-0.007]	-0.060***<br>[-0.103,-0.018]	-0.108***<br>[-0.160,-0.056]	0.038<br>[-0.176,0.252]	0.005<br>[-0.039,0.048]	-0.005<br>[-0.035,0.026]	0.014<br>[-0.019,0.047]
MOT_ANC	-0.112***<br>[-0.150,-0.075]	-0.155***<br>[-0.171,-0.140]	-0.124***<br>[-0.136,-0.113]	-0.179***<br>[-0.205,-0.152]	0.029*<br>[-0.004,0.062]	0.083***<br>[0.067,0.098]	0.049***<br>[0.039,0.060]	0.069***<br>[0.046,0.092]	0.071***<br>[0.040,0.103]	0.070***<br>[0.057,0.083]	0.054***<br>[0.045,0.063]	0.071***<br>[0.051,0.090]
MOT_EDM	-0.129***<br>[-0.201,-0.058]	-0.056***<br>[-0.083,-0.028]	-0.061***<br>[-0.082,-0.040]	-0.085***<br>[-0.122,-0.047]	0.076*<br>[-0.008,0.159]	0.057***<br>[0.020,0.094]	0.056***<br>[0.026,0.086]	0.047*<br>[-0.004,0.098]	0.102***<br>[0.030,0.174]	0.041***<br>[0.014,0.068]	0.055***<br>[0.034,0.075]	0.088***<br>[0.053,0.124]
MOT_EDF	-0.012<br>[-0.084,0.060]	-0.039***<br>[-0.066,-0.011]	-0.023**<br>[-0.045,-0.002]	-0.041**<br>[-0.078,-0.004]	0.029<br>[-0.061,0.119]	0.027<br>[-0.012,0.065]	0.046***<br>[0.014,0.078]	0.028<br>[-0.027,0.082]	0.009<br>[-0.065,0.083]	0.043***<br>[0.016,0.069]	0.024**<br>[0.004,0.045]	0.051***<br>[0.016,0.087]
MOT_AWE	-0.746***<br>[-1.124,-0.368]	-0.934***<br>[-1.072,-0.795]	-0.982***<br>[-1.092,-0.872]	-0.765***<br>[-0.966,-0.564]	0.229<br>[-0.229,0.688]	0.327***<br>[0.128,0.526]	0.259***<br>[0.088,0.430]	0.238<br>[-0.061,0.537]	0.848***<br>[0.446,1.249]	0.990***<br>[0.846,1.133]	1.075***<br>[0.962,1.188]	0.809***<br>[0.609,1.010]
MOT_AGE	-0.013<br>[-0.039,0.013]	-0.001<br>[-0.010,0.008]	-0.001<br>[-0.009,0.006]	-0.006<br>[-0.019,0.007]	-0.005<br>[-0.035,0.026]	0.002<br>[-0.010,0.015]	-0.001<br>[-0.010,0.009]	0.002<br>[-0.014,0.019]	0.018<br>[-0.008,0.044]	0.000<br>[-0.009,0.009]	0.003<br>[-0.004,0.009]	0.005<br>[-0.007,0.016]
HH_RUR	0.476**<br>[0.034,0.919]	0.641***<br>[0.448,0.835]	0.709***<br>[0.572,0.846]	0.450***<br>[0.227,0.672]	-0.086<br>[-0.548,0.375]	-0.329***<br>[-0.570,-0.089]	-0.592***<br>[-0.758,-0.426]	-0.431***<br>[-0.686,-0.176]	-0.526**<br>[-0.960,-0.092]	-0.436***<br>[-0.623,-0.248]	-0.356***<br>[-0.479,-0.233]	-0.241**<br>[-0.443,-0.039]
HH_REL (ref: Catholic): Other Christian	-0.228<br>[-0.756,0.299]	0.055<br>[-0.176,0.285]	0.199**<br>[0.008,0.390]	0.176<br>[-0.132,0.485]	0.514**<br>[0.018,1.010]	0.266**<br>[0.019,0.512]	-0.283***<br>[-0.467,-0.099]	-0.391***<br>[-0.669,-0.113]	-0.370<br>[-0.876,0.136]	-0.278**<br>[-0.493,-0.063]	0.090<br>[-0.073,0.254]	0.203<br>[-0.054,0.461]
HH_REL (ref: Catholic): Islam	0.347<br>[-0.211,0.905]	-0.047<br>[-0.333,0.239]	0.411***<br>[0.182,0.640]	0.416**<br>[0.030,0.802]	0.171<br>[-0.382,0.724]	0.270*<br>[-0.049,0.589]	-0.558***<br>[-0.798,-0.319]	-0.348*<br>[-0.737,0.042]	-0.467*<br>[-1.002,0.068]	-0.191<br>[-0.454,0.073]	0.066<br>[-0.134,0.267]	-0.052<br>[-0.388,0.284]
HH_REL (ref: Catholic): Traditionalist	2.659**<br>[0.237,5.081]	1.275***<br>[0.678,1.872]	0.673**<br>[0.148,1.198]	0.535<br>[-0.915,1.985]	0.000<br>[0.000,0.000]	-0.624*<br>[-1.326,0.077]	-0.779**<br>[-1.488,-0.070]	-1.217<br>[-3.410,0.976]	-1.795<br>[-4.091,0.500]	-1.044***<br>[-1.706,-0.383]	-0.099<br>[-0.608,0.410]	0.332<br>[-1.031,1.696]
HH_REL (ref: Catholic): Other	0.000<br>[0.000,0.000]	-1.175<br>[-2.618,0.269]	0.003<br>[-2.187,2.194]	-0.456<br>[-1.750,0.838]	0.000<br>[0.000,0.000]	1.580**<br>[0.067,3.094]	0.000<br>[0.000,0.000]	0.000<br>[0.000,0.000]	0.000<br>[0.000,0.000]	0.330<br>[-1.066,1.726]	0.489<br>[-1.708,2.685]	1.074*<br>[-0.143,2.291]
HH_ETH (ref: Ekoi): Fulani	0.000<br>[0.000,0.000]	1.075***<br>[0.394,1.756]	0.369<br>[-0.914,1.651]	0.357<br>[-0.785,1.498]	0.000<br>[0.000,0.000]	-0.040<br>[-1.341,1.262]	-0.898<br>[-3.171,1.375]	-0.153<br>[-1.943,1.638]	0.000<br>[0.000,0.000]	-1.106***<br>[-1.798,-0.414]	-0.403<br>[-1.644,0.839]	-0.625<br>[-1.722,0.471]
HH_ETH (ref: Ekoi): Hausa	0.924**<br>[0.152,1.696]	1.371***<br>[0.719,2.023]	0.590<br>[-0.682,1.862]	0.759<br>[-0.351,1.869]	-1.297<br>[-2.864,0.271]	-1.110*<br>[-2.374,0.154]	-0.953<br>[-3.152,1.247]	-0.675<br>[-2.384,1.035]	-0.617<br>[-1.391,0.158]	-1.075***<br>[-1.736,-0.415]	-0.514<br>[-1.745,0.717]	-0.770<br>[-1.834,0.295]
HH_ETH (ref: Ekoi): Ibibio	-0.948<br>[-2.910,1.014]	0.178<br>[-0.575,0.931]	0.484<br>[-0.832,1.800]	0.610<br>[-0.601,1.821]	0.744<br>[-1.904,3.391]	0.846<br>[-0.451,2.142]	-0.114<br>[-2.351,2.123]	-0.010<br>[-1.848,1.828]	1.351<br>[-0.631,3.333]	-0.198<br>[-0.943,0.546]	-0.248<br>[-1.521,1.024]	-0.492<br>[-1.656,0.673]
HH_ETH (ref: Ekoi): Igala	-1.208<br>[-2.662,0.247]	-0.841*<br>[-1.703,0.020]	-0.481<br>[-1.845,0.882]	-0.705<br>[-2.034,0.624]	1.997**<br>[0.298,3.695]	1.364**<br>[0.074,2.654]	0.878<br>[-1.350,3.106]	1.585*<br>[-0.224,3.394]	0.324<br>[-1.053,1.702]	0.288<br>[-0.516,1.093]	-0.001<br>[-1.297,1.296]	-0.234<br>[-1.495,1.027]
HH_ETH (ref: Ekoi): Igbo	-0.910*<br>[-1.907,0.088]	-1.046***<br>[-1.669,-0.424]	-0.853<br>[-2.124,0.418]	-0.730<br>[-1.824,0.364]	2.503***<br>[1.109,3.896]	2.415***<br>[1.271,3.559]	1.396<br>[-0.776,3.568]	1.515*<br>[-0.123,3.153]	-0.728<br>[-1.717,0.261]	-0.660**<br>[-1.289,-0.031]	-0.459<br>[-1.685,0.767]	-0.554<br>[-1.596,0.487]
HH_ETH (ref: Ekoi): Ijaw / Izon	0.012<br>[-1.949,1.973]	0.996**<br>[0.218,1.775]	0.881<br>[-0.435,2.196]	1.401**<br>[0.175,2.628]	0.257<br>[-2.349,2.862]	0.102<br>[-1.229,1.434]	-0.448<br>[-2.713,1.817]	-0.270<br>[-2.136,1.597]	0.377<br>[-1.539,2.292]	-0.640<br>[-1.437,0.157]	-0.825<br>[-2.099,0.448]	-1.319**<br>[-2.502,-0.135]
HH_ETH (ref: Ekoi): Kanuri / Beriberi	0.656<br>[-0.672,1.983]	1.330***<br>[0.557,2.102]	0.663<br>[-0.674,1.999]	0.511<br>[-0.775,1.797]	0.000<br>[0.000,0.000]	-0.880<br>[-2.734,0.975]	-0.741<br>[-3.228,1.745]	-0.448<br>[-2.686,1.790]	0.131<br>[-1.139,1.402]	-1.285***<br>[-2.060,-0.510]	-0.555<br>[-1.848,0.738]	-0.644<br>[-1.890,0.602]
HH_ETH (ref: Ekoi): Tiv	-1.588**<br>[-3.085,-0.091]	-0.853**<br>[-1.559,-0.146]	-0.951<br>[-2.279,0.378]	-1.013*<br>[-2.218,0.191]	2.493***<br>[0.804,4.181]	2.198***<br>[0.960,3.437]	1.302<br>[-0.924,3.527]	1.892**<br>[0.164,3.621]	-0.041<br>[-1.477,1.395]	-0.025<br>[-0.756,0.706]	0.358<br>[-0.931,1.647]	0.062<br>[-1.090,1.215]
HH_ETH (ref: Ekoi): Yoruba	-0.659<br>[-1.615,0.297]	-0.370<br>[-1.011,0.271]	-0.137<br>[-1.409,1.134]	-0.332<br>[-1.443,0.779]	1.967***<br>[0.601,3.333]	1.501**<br>[0.341,2.661]	0.855<br>[-1.318,3.029]	1.004<br>[-0.650,2.657]	-0.462<br>[-1.400,0.476]	-0.288<br>[-0.933,0.357]	-0.408<br>[-1.636,0.820]	-0.334<br>[-1.390,0.722]
HH_ETH (ref: Ekoi): Other	-0.001<br>[-0.779,0.777]	0.307<br>[-0.299,0.913]	0.015<br>[-1.243,1.274]	-0.015<br>[-1.092,1.062]	1.369**<br>[0.075,2.663]	0.946<br>[-0.200,2.092]	0.373<br>[-1.795,2.541]	0.781<br>[-0.858,2.421]	-0.441<br>[-1.240,0.359]	-0.414<br>[-1.033,0.204]	-0.089<br>[-1.308,1.129]	-0.337<br>[-1.370,0.695]
HH_SIZ	0.030<br>[-0.018,0.078]	0.022**<br>[0.004,0.040]	0.038***<br>[0.023,0.052]	0.044***<br>[0.019,0.069]	-0.043<br>[-0.104,0.018]	-0.006<br>[-0.032,0.020]	-0.021*<br>[-0.044,0.002]	-0.035*<br>[-0.072,0.002]	0.003<br>[-0.045,0.051]	-0.014<br>[-0.032,0.004]	-0.026***<br>[-0.039,-0.012]	-0.021*<br>[-0.045,0.002]
HH_WEA	-0.247***<br>[-0.334,-0.160]	-0.264***<br>[-0.299,-0.230]	-0.186***<br>[-0.210,-0.161]	-0.183***<br>[-0.222,-0.145]	0.177***<br>[0.090,0.264]	0.143***<br>[0.101,0.186]	0.106***<br>[0.075,0.137]	0.150***<br>[0.103,0.196]	0.082**<br>[0.003,0.160]	0.174***<br>[0.142,0.206]	0.118***<br>[0.096,0.140]	0.086***<br>[0.051,0.121]
Constant	2.344***<br>[0.986,3.702]	2.386***<br>[1.683,3.089]	1.702**<br>[0.396,3.008]	1.973***<br>[0.767,3.180]	-3.864***<br>[-5.696,-2.032]	-4.914***<br>[-6.157,-3.670]	-3.104***<br>[-5.323,-0.886]	-3.415***<br>[-5.192,-1.639]	-2.637***<br>[-3.998,-1.277]	-2.205***<br>[-2.916,-1.493]	-2.397***<br>[-3.658,-1.136]	-2.225***<br>[-3.372,-1.079]
Multilevel variance parameter: Level 1	0.733***<br>[0.226,1.240]	0.652***<br>[0.466,0.839]	0.490***<br>[0.368,0.612]	0.619***<br>[0.402,0.835]	0.439*<br>[-0.014,0.892]	1.004***<br>[0.672,1.335]	0.814***<br>[0.584,1.045]	0.606***<br>[0.351,0.861]	0.846***<br>[0.332,1.360]	0.761***<br>[0.570,0.952]	0.473***<br>[0.364,0.581]	0.639***<br>[0.442,0.836]
Level 1 Observations (child)	1395	11609	16475	5233	1346	11609	16467	5211	1395	11609	16475	5233
Level 2 Observations (LGA)	153	370	370	510	153	370	370	510	153	370	370	510
Akaike Information Criterion	1147.831	7880.9	12731.565	4519.37	901.306	5217.905	8321.506	3169.365	1222.345	8804.345	14843.344	5370.273
Prob. > X^2	<0.001	<0.001	<0.001	<0.001	<0.001	<0.001	<0.001	<0.001	<0.001	<0.001	<0.001	<0.001

95% confidence intervals in brackets

* p < 0.10, ** p < 0.05, *** p < 0.01

Table 6b-3: Additional robustness checks: year-wise stratification to detect time trends: antenatal care and child survival

Dependent variables: various indicators of maternal care and child survival (see right)	Antenatal care								Child survival			
	No. of antenatal care visits				No. of tetanus injections				Total exposure			
	2003	2008	2013	2018	2003	2008	2013	2018	2003	2008	2013	2018
EXP_PREG	0.015<br>[-0.239,0.270]	-0.051<br>[-0.113,0.010]	-0.024<br>[-0.074,0.025]	-0.054***<br>[-0.086,-0.022]	0.014<br>[-0.056,0.084]	-0.012<br>[-0.030,0.006]	-0.005<br>[-0.018,0.008]	0.006<br>[-0.008,0.019]				
EXP_TOT_nod (total exposure, date approximation)									-0.087<br>[-0.267,0.092]	-0.038***<br>[-0.061,-0.015]	-0.006<br>[-0.021,0.009]	-0.037***<br>[-0.052,-0.021]
CHI_AGE									-0.122*<br>[-0.249,0.004]	0.007<br>[-0.011,0.026]	0.001<br>[-0.016,0.017]	-0.003<br>[-0.019,0.014]
CHI_AGE2									0.005*<br>[-0.001,0.011]	0.000<br>[-0.000,0.000]	0.000<br>[-0.000,0.000]	0.000*<br>[-0.000,0.000]
CHI_ORD									-0.159***<br>[-0.276,-0.043]	-0.142***<br>[-0.183,-0.102]	-0.138***<br>[-0.178,-0.099]	-0.171***<br>[-0.212,-0.131]
CHI_SEX									0.182<br>[-0.173,0.537]	0.283***<br>[0.149,0.418]	0.224***<br>[0.098,0.350]	0.151**<br>[0.027,0.275]
MOT_ANC									0.043*<br>[-0.007,0.093]	-0.009<br>[-0.027,0.010]	0.006<br>[-0.010,0.022]	-0.005<br>[-0.025,0.015]
MOT_EDM	0.122**<br>[0.000,0.244]	0.123***<br>[0.084,0.162]	0.088***<br>[0.055,0.121]	0.074***<br>[0.024,0.123]	0.047***<br>[0.016,0.078]	0.033***<br>[0.023,0.043]	0.028***<br>[0.017,0.038]	0.035***<br>[0.018,0.051]	0.066<br>[-0.026,0.158]	0.007<br>[-0.028,0.043]	0.023<br>[-0.010,0.057]	0.003<br>[-0.030,0.036]
MOT_EDF	0.094<br>[-0.028,0.216]	0.075***<br>[0.040,0.109]	0.093***<br>[0.064,0.121]	0.025<br>[-0.021,0.072]	0.010<br>[-0.018,0.038]	0.020***<br>[0.010,0.031]	0.026***<br>[0.016,0.036]	0.023***<br>[0.009,0.038]	0.092**<br>[0.009,0.176]	0.035**<br>[0.003,0.068]	0.003<br>[-0.028,0.034]	0.035**<br>[0.006,0.064]
MOT_AWE	2.141***<br>[1.525,2.757]	1.721***<br>[1.483,1.959]	1.955***<br>[1.752,2.158]	1.553***<br>[1.328,1.779]	0.750***<br>[0.563,0.937]	0.582***<br>[0.511,0.654]	0.640***<br>[0.579,0.700]	0.576***<br>[0.505,0.647]				
MOT_AGE	0.035**<br>[0.003,0.067]	0.005<br>[-0.005,0.015]	0.016***<br>[0.008,0.025]	0.006<br>[-0.009,0.022]	0.007<br>[-0.003,0.017]	0.002<br>[-0.001,0.005]	0.001<br>[-0.002,0.004]	-0.005**<br>[-0.009,-0.000]	0.041*<br>[-0.006,0.088]	0.013*<br>[-0.002,0.028]	0.003<br>[-0.012,0.017]	0.007<br>[-0.008,0.022]
HH_RUR	-0.486<br>[-1.276,0.305]	-0.456*<br>[-0.941,0.030]	-0.726***<br>[-1.070,-0.381]	-0.717***<br>[-1.048,-0.387]	-0.115<br>[-0.253,0.023]	-0.199***<br>[-0.300,-0.098]	-0.171***<br>[-0.248,-0.095]	-0.117***<br>[-0.205,-0.029]	-0.067<br>[-0.560,0.427]	-0.236**<br>[-0.439,-0.033]	-0.365***<br>[-0.550,-0.181]	-0.049<br>[-0.219,0.121]
HH_REL (ref: Catholic): Other Christian	0.320<br>[-0.579,1.220]	-0.156<br>[-0.492,0.179]	-0.057<br>[-0.424,0.310]	0.309<br>[-0.086,0.704]	0.197<br>[-0.048,0.443]	-0.051<br>[-0.143,0.040]	-0.049<br>[-0.133,0.034]	-0.085<br>[-0.190,0.021]	0.083<br>[-0.578,0.745]	0.041<br>[-0.263,0.345]	-0.247<br>[-0.546,0.052]	-0.412**<br>[-0.739,-0.086]
HH_REL (ref: Catholic): Islam	0.058<br>[-1.035,1.151]	-0.153<br>[-0.614,0.308]	-0.203<br>[-0.690,0.284]	-0.025<br>[-0.545,0.495]	0.004<br>[-0.227,0.236]	-0.135**<br>[-0.259,-0.010]	-0.125**<br>[-0.231,-0.019]	-0.083<br>[-0.228,0.061]	0.682**<br>[0.021,1.344]	0.195<br>[-0.167,0.557]	-0.303*<br>[-0.657,0.052]	-0.369*<br>[-0.748,0.009]
HH_REL (ref: Catholic): Traditionalist	-1.539***<br>[-2.633,-0.445]	-0.418<br>[-1.022,0.186]	-0.582<br>[-1.401,0.238]	-1.368**<br>[-2.474,-0.262]	0.075<br>[-0.669,0.820]	-0.440***<br>[-0.623,-0.257]	-0.357***<br>[-0.585,-0.128]	-0.213<br>[-0.621,0.194]	-0.160<br>[-1.352,1.031]	-0.059<br>[-0.591,0.472]	-0.187<br>[-0.876,0.501]	1.121<br>[-0.914,3.155]
HH_REL (ref: Catholic): Other	-4.444***<br>[-5.625,-3.264]	1.812<br>[-0.963,4.587]	-1.774***<br>[-2.603,-0.946]	0.433<br>[-1.236,2.102]	-0.814***<br>[-1.099,-0.529]	0.288<br>[-0.732,1.307]	-0.541<br>[-1.258,0.176]	-0.017<br>[-0.660,0.627]	0.000<br>[0.000,0.000]	-0.835<br>[-2.150,0.481]	0.000<br>[0.000,0.000]	0.000<br>[0.000,0.000]
HH_ETH (ref: Ekoi): Fulani	0.000<br>[0.000,0.000]	-0.658<br>[-2.000,0.683]	0.567<br>[-2.267,3.401]	-0.490<br>[-2.077,1.096]	0.000<br>[0.000,0.000]	-0.541***<br>[-0.881,-0.201]	-0.336***<br>[-0.521,-0.152]	-0.281**<br>[-0.542,-0.021]	0.000<br>[0.000,0.000]	-0.356<br>[-1.217,0.505]	0.302*<br>[-0.012,0.617]	0.472<br>[-0.516,1.461]
HH_ETH (ref: Ekoi): Hausa	0.074<br>[-0.470,0.619]	-0.617<br>[-1.948,0.714]	1.027<br>[-1.798,3.852]	0.037<br>[-1.542,1.616]	0.062<br>[-0.137,0.260]	-0.539***<br>[-0.875,-0.202]	-0.179**<br>[-0.355,-0.003]	-0.164<br>[-0.419,0.090]	-0.577<br>[-1.324,0.169]	-0.598<br>[-1.448,0.252]	-0.163<br>[-0.389,0.064]	0.263<br>[-0.710,1.235]
HH_ETH (ref: Ekoi): Ibibio	1.244<br>[-1.726,4.213]	1.024<br>[-0.267,2.314]	0.716<br>[-2.289,3.721]	0.064<br>[-1.630,1.758]	0.364<br>[-0.397,1.126]	-0.255<br>[-0.619,0.110]	-0.199<br>[-0.444,0.047]	0.119<br>[-0.247,0.485]	-2.494***<br>[-4.324,-0.664]	-0.137<br>[-1.235,0.961]	-0.204<br>[-0.758,0.351]	0.185<br>[-0.906,1.275]
HH_ETH (ref: Ekoi): Igala	3.934***<br>[2.295,5.573]	0.215<br>[-1.301,1.731]	0.941<br>[-1.957,3.838]	0.854<br>[-0.935,2.642]	2.076***<br>[1.699,2.453]	0.024<br>[-0.375,0.424]	0.189<br>[-0.112,0.491]	0.193<br>[-0.241,0.628]	-1.161<br>[-2.970,0.648]	-0.713<br>[-1.709,0.282]	0.528<br>[-0.403,1.459]	0.114<br>[-1.020,1.247]
HH_ETH (ref: Ekoi): Igbo	2.564***<br>[1.360,3.768]	1.493**<br>[0.169,2.818]	2.530*<br>[-0.338,5.398]	2.459***<br>[0.844,4.073]	0.657***<br>[0.299,1.015]	-0.077<br>[-0.404,0.250]	0.103<br>[-0.043,0.249]	0.301***<br>[0.072,0.530]	-0.783<br>[-1.910,0.345]	-0.654<br>[-1.467,0.159]	-0.409***<br>[-0.695,-0.122]	0.289<br>[-0.675,1.252]
HH_ETH (ref: Ekoi): Ijaw / Izon	1.149<br>[-1.180,3.478]	-0.127<br>[-1.742,1.488]	0.263<br>[-2.656,3.183]	-0.052<br>[-2.106,2.002]	0.201<br>[-0.240,0.641]	-0.184<br>[-0.601,0.233]	-0.198*<br>[-0.422,0.025]	-0.183<br>[-0.541,0.174]	-1.101<br>[-3.020,0.818]	-0.575<br>[-1.472,0.323]	-0.179<br>[-0.592,0.234]	1.357**<br>[0.109,2.605]
HH_ETH (ref: Ekoi): Kanuri / Beriberi	1.003**<br>[0.009,1.996]	-0.914<br>[-2.320,0.491]	1.163<br>[-1.701,4.028]	-0.290<br>[-1.924,1.345]	0.223<br>[-0.507,0.953]	-0.534***<br>[-0.911,-0.157]	-0.157<br>[-0.405,0.091]	-0.234<br>[-0.611,0.143]	-0.102<br>[-1.519,1.315]	-0.529<br>[-1.434,0.377]	0.231<br>[-0.391,0.854]	0.571<br>[-0.493,1.636]
HH_ETH (ref: Ekoi): Tiv	-0.068<br>[-1.797,1.661]	-0.978<br>[-2.399,0.443]	0.077<br>[-2.858,3.012]	0.280<br>[-1.475,2.036]	0.326<br>[-0.395,1.047]	-0.565***<br>[-0.929,-0.202]	-0.375**<br>[-0.669,-0.082]	-0.197<br>[-0.461,0.068]	-0.327<br>[-1.976,1.322]	-0.847*<br>[-1.710,0.016]	-0.142<br>[-0.698,0.415]	0.856<br>[-0.246,1.959]
HH_ETH (ref: Ekoi): Yoruba	5.942***<br>[4.397,7.488]	2.849***<br>[1.460,4.238]	3.553**<br>[0.695,6.412]	3.056***<br>[1.428,4.685]	0.767***<br>[0.436,1.098]	-0.071<br>[-0.401,0.258]	0.049<br>[-0.110,0.208]	-0.027<br>[-0.263,0.208]	-1.234*<br>[-2.477,0.009]	-0.103<br>[-0.951,0.745]	0.168<br>[-0.141,0.476]	0.808<br>[-0.178,1.794]
HH_ETH (ref: Ekoi): Other	1.491***<br>[0.544,2.437]	0.149<br>[-1.149,1.447]	1.326<br>[-1.484,4.137]	0.679<br>[-0.884,2.242]	0.556***<br>[0.259,0.852]	-0.211<br>[-0.536,0.114]	-0.040<br>[-0.186,0.106]	0.059<br>[-0.168,0.285]	-0.829*<br>[-1.679,0.021]	-0.570<br>[-1.378,0.238]	0.000<br>[0.000,0.000]	0.466<br>[-0.489,1.421]
HH_SIZ	-0.070**<br>[-0.140,-0.001]	-0.031***<br>[-0.052,-0.010]	-0.066***<br>[-0.086,-0.047]	-0.036**<br>[-0.064,-0.008]	-0.009<br>[-0.024,0.006]	-0.003<br>[-0.009,0.002]	-0.014***<br>[-0.020,-0.007]	-0.001<br>[-0.011,0.009]	0.059*<br>[-0.001,0.118]	0.134***<br>[0.107,0.161]	0.153***<br>[0.127,0.180]	0.133***<br>[0.108,0.157]
HH_WEA	0.348***<br>[0.212,0.484]	0.356***<br>[0.306,0.405]	0.297***<br>[0.250,0.345]	0.221***<br>[0.171,0.272]	0.076***<br>[0.045,0.108]	0.073***<br>[0.058,0.087]	0.063***<br>[0.050,0.075]	0.037***<br>[0.021,0.053]	0.109**<br>[0.000,0.217]	-0.018<br>[-0.060,0.024]	0.018<br>[-0.016,0.053]	0.015<br>[-0.017,0.048]
Constant	0.814<br>[-0.905,2.534]	2.695***<br>[1.257,4.132]	2.145<br>[-0.805,5.095]	2.444***<br>[0.707,4.182]	0.054<br>[-0.379,0.487]	1.125***<br>[0.765,1.485]	1.010***<br>[0.806,1.215]	1.074***<br>[0.766,1.383]	2.191***<br>[0.564,3.818]	2.852***<br>[1.933,3.771]	2.839***<br>[2.295,3.383]	2.454***<br>[1.373,3.536]
Multilevel variance parameter: Level 1	0.446***<br>[0.139,0.754]	0.674***<br>[0.535,0.812]	0.881***<br>[0.773,0.988]	0.382***<br>[0.215,0.548]	-1.847***<br>[-2.590,-1.104]	-1.260***<br>[-1.389,-1.131]	-1.339***<br>[-1.463,-1.216]	-1.244***<br>[-1.420,-1.068]	0.269<br>[-0.119,0.658]	0.035<br>[-0.022,0.092]	0.079**<br>[0.013,0.145]	0.066**<br>[0.005,0.127]
Multilevel variance parameter: Residual	1.395***<br>[1.266,1.523]	1.270***<br>[1.213,1.327]	1.350***<br>[1.298,1.402]	1.173***<br>[1.122,1.225]	0.126***<br>[0.031,0.222]	0.041*<br>[-0.005,0.086]	0.114***<br>[0.078,0.150]	0.036*<br>[-0.006,0.079]				
Level 1 Observations (child)	1400	11781	16510	5328	1437	12654	17086	5408	1921	14275	18276	17857
Level 2 Observations (LGA)	153	370	370	511	154	370	370	512	158	370	370	515
Akaike Information Criterion	8042.517	64196.451	92468.14	28199.742	4511.606	37408.855	52854.597	16072.681	1014.205	6786.461	7863.049	8003.619
Prob. > X^2	<0.001	<0.001	<0.001	<0.001	<0.001	<0.001	<0.001	<0.001	<0.001	<0.001	<0.001	<0.001

95% confidence intervals in brackets
* p < 0.10, ** p < 0.05, *** p < 0.01

Table 6c: Additional robustness checks: exclusion of measles vaccine from full immunisation outcome

Dependent variable: non-polio/-measles full immunisation status	Full model	Interaction model (EXPxAGE)	**Full model (by region)** North Central	North East	North West	South East	South South	South West	**Interaction model (EXPxAGE) (by region)** North Central	North East	North West	South East	South South	South West
EXP_CHI	-0.020***	-0.032***	0.028*	-0.061***	-0.045	0.051	-0.019	-0.064	0.025	-0.065***	-0.041	0.057	-0.063	-0.085
	[-0.033,-0.008]	[-0.054,-0.010]	[-0.003,0.059]	[-0.094,-0.028]	[-0.118,0.029]	[-0.100,0.202]	[-0.131,0.093]	[-0.154,0.026]	[-0.022,0.072]	[-0.110,-0.019]	[-0.116,0.034]	[-0.157,0.271]	[-0.245,0.118]	[-0.213,0.043]
EXPxAGE		0.000							0.000	0.000	0.000	-0.001	0.001	0.000
		[-0.000,0.001]							[-0.001,0.001]	[-0.001,0.001]	[-0.001,0.001]	[-0.004,0.003]	[-0.002,0.003]	[-0.002,0.002]
CHI_AGE	0.026***	0.016***	0.009	0.049**	0.051	0.022	0.008	0.041**	-0.003	0.034***	0.033	0.007	0.011	0.032***
	[0.010,0.042]	[0.009,0.023]	[-0.023,0.041]	[0.008,0.091]	[-0.015,0.117]	[-0.029,0.072]	[-0.031,0.048]	[0.005,0.078]	[-0.019,0.014]	[0.011,0.056]	[-0.015,0.081]	[-0.028,0.042]	[-0.015,0.038]	[0.009,0.055]
CHI_AGE2	0.000		0.000	0.000	0.000	0.000	0.000	0.000						
	[-0.000,0.000]		[-0.001,0.000]	[-0.001,0.000]	[-0.001,0.000]	[-0.001,0.001]	[-0.001,0.001]	[-0.001,0.000]						
CHI_ORD	-0.046***	-0.046***	-0.018	-0.081***	-0.038	0.003	-0.071**	-0.056	-0.018	-0.082***	-0.038	0.004	-0.071**	-0.056
	[-0.071,-0.020]	[-0.071,-0.020]	[-0.072,0.036]	[-0.142,-0.020]	[-0.102,0.026]	[-0.072,0.078]	[-0.136,-0.005]	[-0.131,0.020]	[-0.072,0.037]	[-0.143,-0.021]	[-0.101,0.026]	[-0.071,0.079]	[-0.137,-0.005]	[-0.131,0.019]
CHI_SEX	-0.006	-0.006	-0.050	-0.038	0.048	-0.026	0.158	-0.073	-0.048	-0.039	0.045	-0.026	0.158	-0.072
	[-0.084,0.072]	[-0.084,0.072]	[-0.208,0.108]	[-0.222,0.146]	[-0.149,0.244]	[-0.279,0.228]	[-0.045,0.362]	[-0.266,0.121]	[-0.206,0.110]	[-0.223,0.144]	[-0.151,0.242]	[-0.280,0.228]	[-0.045,0.362]	[-0.266,0.122]
MOT_ANC	0.061***	0.061***	0.100***	0.135***	0.070***	0.027*	0.044***	0.041***	0.100***	0.135***	0.071***	0.027*	0.044***	0.041***
	[0.052,0.071]	[0.052,0.071]	[0.077,0.123]	[0.101,0.169]	[0.035,0.106]	[-0.000,0.055]	[0.022,0.066]	[0.025,0.057]	[0.077,0.123]	[0.101,0.169]	[0.035,0.106]	[-0.001,0.054]	[0.023,0.066]	[0.025,0.058]
MOT_EDM	0.056***	0.056***	0.054***	0.049**	0.073***	0.073**	0.046*	0.044*	0.054***	0.049**	0.073***	0.074**	0.045*	0.044*
	[0.037,0.074]	[0.037,0.074]	[0.019,0.090]	[0.008,0.091]	[0.027,0.119]	[0.005,0.142]	[-0.007,0.098]	[-0.006,0.094]	[0.019,0.089]	[0.007,0.091]	[0.027,0.118]	[0.006,0.143]	[-0.008,0.098]	[-0.006,0.094]
MOT_EDF	0.024**	0.024**	0.035*	0.014	0.044**	0.014	0.024	-0.033	0.035*	0.014	0.044**	0.013	0.024	-0.034
	[0.006,0.043]	[0.006,0.043]	[-0.002,0.072]	[-0.026,0.055]	[0.002,0.085]	[-0.057,0.085]	[-0.034,0.082]	[-0.086,0.020]	[-0.002,0.072]	[-0.026,0.055]	[0.003,0.085]	[-0.058,0.084]	[-0.034,0.082]	[-0.086,0.019]
MOT_AWE	3.124***	3.121***	2.731***	2.920***	3.684***	3.059***	2.965***	3.248***	2.730***	2.920***	3.683***	3.053***	2.964***	3.240***
	[3.008,3.240]	[3.005,3.237]	[2.505,2.956]	[2.644,3.196]	[3.415,3.953]	[2.682,3.436]	[2.649,3.281]	[2.892,3.604]	[2.505,2.955]	[2.644,3.196]	[3.414,3.952]	[2.677,3.430]	[2.648,3.280]	[2.885,3.595]
MOT_AGE	0.021***	0.021***	0.011	0.031***	0.033***	0.016	0.023**	0.015	0.011	0.031***	0.033***	0.015	0.023**	0.015
	[0.012,0.030]	[0.012,0.030]	[-0.007,0.029]	[0.008,0.054]	[0.008,0.058]	[-0.012,0.043]	[0.001,0.045]	[-0.007,0.036]	[-0.007,0.029]	[0.008,0.054]	[0.008,0.057]	[-0.012,0.043]	[0.001,0.046]	[-0.007,0.037]
HH_RUR	-0.150***	-0.150***	-0.045	-0.214	-0.172	-0.073	-0.037	-0.377***	-0.044	-0.214	-0.173	-0.072	-0.036	-0.376***
	[-0.260,-0.040]	[-0.259,-0.040]	[-0.279,0.188]	[-0.490,0.063]	[-0.484,0.140]	[-0.374,0.228]	[-0.299,0.226]	[-0.645,-0.109]	[-0.278,0.190]	[-0.491,0.062]	[-0.485,0.138]	[-0.374,0.229]	[-0.298,0.226]	[-0.644,-0.108]
HH_REL (ref: Catholic): Other Christian	-0.187**	-0.188**	-0.111	-0.611*	0.389	-0.263*	-0.345*	-0.132	-0.112	-0.616*	0.387	-0.261*	-0.350*	-0.132
	[-0.343,-0.031]	[-0.344,-0.032]	[-0.387,0.164]	[-1.272,0.051]	[-0.330,1.108]	[-0.542,0.016]	[-0.726,0.036]	[-0.710,0.447]	[-0.387,0.163]	[-1.276,0.044]	[-0.332,1.106]	[-0.540,0.018]	[-0.730,0.031]	[-0.710,0.447]
HH_REL (ref: Catholic): Islam	-0.575***	-0.571***	-0.448***	-1.070***	-0.348	-0.342	-0.101	-0.464	-0.446***	-1.080***	-0.348	-0.336	-0.103	-0.463
	[-0.761,-0.389]	[-0.757,-0.385]	[-0.759,-0.137]	[-1.744,-0.396]	[-1.166,0.470]	[-3.017,2.333]	[-0.916,0.714]	[-1.059,0.131]	[-0.758,-0.135]	[-1.752,-0.407]	[-1.166,0.469]	[-3.009,2.336]	[-0.917,0.712]	[-1.057,0.132]
HH_REL (ref: Catholic): Traditionalist	-0.757***	-0.756***	-0.964*	-1.116*	-0.812	-0.562	-0.866*	-0.025	-0.953*	-1.136*	-0.789	-0.557	-0.872*	-0.044
	[-1.166,-0.347]	[-1.165,-0.346]	[-2.082,0.155]	[-2.434,0.202]	[-2.396,0.773]	[-1.235,0.110]	[-1.896,0.163]	[-1.378,1.327]	[-2.070,0.163]	[-2.452,0.180]	[-2.370,0.792]	[-1.230,0.115]	[-1.903,0.158]	[-1.393,1.305]
HH_REL (ref: Catholic): Other	-0.098	-0.106	-0.138	0.000	0.000	0.000	-0.301	0.447	-0.137	0.000	0.000	0.000	-0.306	0.436
	[-1.149,0.952]	[-1.156,0.943]	[-2.341,2.065]	[0.000,0.000]	[0.000,0.000]	[0.000,0.000]	[-1.596,0.994]	[-3.479,4.372]	[-2.338,2.065]	[0.000,0.000]	[0.000,0.000]	[0.000,0.000]	[-1.600,0.989]	[-3.479,4.350]
HH_ETH (ref: Ekoi): Fulani	-0.847***	-0.833***	-0.261	-0.138	0.316	0.000		-0.881	-0.252	-0.142	0.319	0.000		-0.863
	[-1.388,-0.305]	[-1.375,-0.292]	[-0.817,0.295]	[-0.430,0.154]	[-0.343,0.975]	[0.000,0.000]		[-1.937,0.175]	[-0.807,0.303]	[-0.434,0.150]	[-0.339,0.978]	[0.000,0.000]		[-1.918,0.193]
HH_ETH (ref: Ekoi): Hausa	-0.804***	-0.784***	-0.461**	-0.137	0.410	-0.677	-0.163	-0.110	-0.459**	-0.137	0.412	-0.660	-0.164	-0.113
	[-1.328,-0.281]	[-1.307,-0.260]	[-0.849,-0.073]	[-0.433,0.158]	[-0.106,0.925]	[-3.000,1.646]	[-2.964,2.638]	[-1.047,0.828]	[-0.848,-0.071]	[-0.432,0.159]	[-0.103,0.928]	[-2.980,1.660]	[-2.970,2.643]	[-1.050,0.825]
HH_ETH (ref: Ekoi): Ibibio	-0.582**	-0.582**	-1.077	0.000	0.000	-0.764	-0.963***	0.727	-1.082	0.000	0.000	-0.748	-0.960***	0.734
	[-1.158,-0.006]	[-1.158,-0.006]	[-3.132,0.979]	[0.000,0.000]	[0.000,0.000]	[-3.466,1.937]	[-1.527,-0.399]	[-0.674,2.128]	[-3.140,0.977]	[0.000,0.000]	[0.000,0.000]	[-3.447,1.951]	[-1.522,-0.397]	[-0.667,2.134]
HH_ETH (ref: Ekoi): Igala	-0.733**	-0.731**	-0.423**	0.000	0.000	-1.131	-1.242	1.297	-0.428**	0.000	0.000	-1.134	-1.257	1.306
	[-1.359,-0.106]	[-1.358,-0.105]	[-0.832,-0.014]	[0.000,0.000]	[0.000,0.000]	[-3.896,1.635]	[-3.919,1.435]	[-0.300,2.894]	[-0.837,-0.019]	[0.000,0.000]	[0.000,0.000]	[-3.897,1.630]	[-3.956,1.441]	[-0.293,2.905]
HH_ETH (ref: Ekoi): Igbo	-0.031	-0.031	-0.312	0.195	0.011	-0.421	-0.199	0.848***	-0.305	0.206	0.013	-0.411	-0.198	0.850***
	[-0.543,0.480]	[-0.542,0.481]	[-0.873,0.248]	[-1.479,1.868]	[-1.068,1.090]	[-1.915,1.074]	[-0.831,0.433]	[0.231,1.465]	[-0.865,0.255]	[-1.466,1.877]	[-1.065,1.091]	[-1.904,1.081]	[-0.829,0.433]	[0.233,1.466]
HH_ETH (ref: Ekoi): Ijaw / Izon	-0.418	-0.419	0.000			0.000	-0.772***	-1.020	0.000			0.000	-0.767***	-1.030
	[-1.001,0.166]	[-1.002,0.165]	[0.000,0.000]			[0.000,0.000]	[-1.306,-0.238]	[-2.616,0.577]	[0.000,0.000]			[0.000,0.000]	[-1.300,-0.234]	[-2.626,0.567]
HH_ETH (ref: Ekoi): Kanuri / Beriberi	-0.940***	-0.915***	-0.903	-0.278	0.270		0.000	2.526*	-0.891	-0.272	0.279		0.000	2.558*
	[-1.569,-0.312]	[-1.544,-0.286]	[-2.583,0.778]	[-0.722,0.166]	[-0.982,1.522]		[0.000,0.000]	[-0.454,5.506]	[-2.572,0.791]	[-0.716,0.172]	[-0.973,1.530]		[0.000,0.000]	[-0.428,5.545]
HH_ETH (ref: Ekoi): Tiv	-0.529*	-0.525*	-0.187	-0.336	0.000	0.000	0.000	1.480	-0.189	-0.360	0.000	0.000	0.000	1.493
	[-1.122,0.064]	[-1.118,0.068]	[-0.574,0.200]	[-1.274,0.603]	[0.000,0.000]	[0.000,0.000]	[0.000,0.000]	[-0.382,3.342]	[-0.576,0.198]	[-1.298,0.579]	[0.000,0.000]	[0.000,0.000]	[0.000,0.000]	[-0.363,3.349]
HH_ETH (ref: Ekoi): Yoruba	-0.398	-0.395	0.162	-0.053	0.949	0.000	-2.229***	0.313*	0.163	-0.042	0.945	0.000	-2.222***	0.313*
	[-0.916,0.120]	[-0.913,0.123]	[-0.192,0.516]	[-1.510,1.403]	[-0.456,2.355]	[0.000,0.000]	[-3.204,-1.254]	[-0.007,0.633]	[-0.191,0.517]	[-1.498,1.414]	[-0.452,2.341]	[0.000,0.000]	[-3.196,-1.248]	[-0.007,0.633]
HH_ETH (ref: Ekoi): Other	-0.499*	-0.490*	0.000	0.000	0.000	0.000	-0.852***	0.000	0.000	0.000	0.000	0.000	-0.850***	0.000
	[-0.999,0.001]	[-0.989,0.010]	[0.000,0.000]	[0.000,0.000]	[0.000,0.000]	[0.000,0.000]	[-1.356,-0.347]	[0.000,0.000]	[0.000,0.000]	[0.000,0.000]	[0.000,0.000]	[0.000,0.000]	[-1.353,-0.346]	[0.000,0.000]
HH_SIZ	-0.014**	-0.014**	-0.012	-0.021*	-0.010	-0.037	-0.013	0.004	-0.013	-0.021*	-0.009	-0.037	-0.013	0.004
	[-0.027,-0.001]	[-0.027,-0.001]	[-0.038,0.014]	[-0.046,0.003]	[-0.038,0.019]	[-0.083,0.009]	[-0.059,0.033]	[-0.047,0.055]	[-0.038,0.013]	[-0.046,0.003]	[-0.037,0.019]	[-0.083,0.008]	[-0.059,0.033]	[-0.047,0.055]
HH_WEA	0.132***	0.132***	0.140***	0.112***	0.120***	0.152***	0.107***	0.121***	0.139***	0.112***	0.120***	0.152***	0.107***	0.122***
	[0.111,0.153]	[0.111,0.153]	[0.095,0.184]	[0.059,0.165]	[0.067,0.173]	[0.090,0.214]	[0.057,0.157]	[0.064,0.179]	[0.095,0.184]	[0.059,0.165]	[0.067,0.173]	[0.090,0.214]	[0.057,0.157]	[0.065,0.179]
YEAR (ref: 2003): 2008	0.784***	0.812***	1.026***	0.798**	1.350***	1.354***	0.961***	-0.273	1.044***	0.826**	1.352***	1.355***	0.952***	-0.237
	[0.524,1.044]	[0.551,1.073]	[0.528,1.525]	[0.030,1.567]	[0.502,2.198]	[0.679,2.029]	[0.237,1.685]	[-0.981,0.436]	[0.543,1.545]	[0.054,1.598]	[0.501,2.203]	[0.680,2.029]	[0.228,1.676]	[-0.948,0.473]
YEAR (ref: 2003): 2013	1.032***	1.064***	0.870***	1.389***	1.696***	1.767***	1.833***	-0.659*	0.894***	1.419***	1.673***	1.787***	1.859***	-0.617*
	[0.773,1.290]	[0.804,1.324]	[0.375,1.365]	[0.625,2.153]	[0.745,2.647]	[1.092,2.442]	[1.113,2.552]	[-1.375,0.056]	[0.397,1.391]	[0.652,2.186]	[0.725,2.621]	[1.111,2.463]	[1.137,2.582]	[-1.333,0.099]
YEAR (ref: 2003): 2018	1.237***	1.252***	1.389***	1.753***	1.447***	2.088***	1.779***	-0.572	1.410***	1.789***	1.490***	2.084***	1.711***	-0.585
	[0.970,1.505]	[0.985,1.518]	[0.874,1.904]	[0.978,2.528]	[0.609,2.285]	[1.281,2.896]	[0.959,2.599]	[-1.341,0.197]	[0.896,1.923]	[1.014,2.563]	[0.659,2.322]	[1.257,2.911]	[0.864,2.559]	[-1.356,0.186]
Constant	-4.445***	-4.303***	-4.563***	-5.058***	-7.592***	-4.376***	-3.903***	-3.203***	-4.425***	-4.860***	-7.380***	-4.239***	-3.851***	-3.074***
	[-5.084,-3.806]	[-4.926,-3.680]	[-5.376,-3.750]	[-6.190,-3.926]	[-8.962,-6.223]	[-6.286,-2.466]	[-5.111,-2.694]	[-4.375,-2.032]	[-5.190,-3.660]	[-5.941,-3.779]	[-8.692,-6.067]	[-6.113,-2.365]	[-5.017,-2.685]	[-4.216,-1.932]
Multilevel variance parameter: Level 1	0.354***	0.353***	0.213***	0.250***	0.695***	0.305***	0.045	0.296***	0.214***	0.252***	0.692***	0.304***	0.043	0.293***
	[0.274,0.434]	[0.273,0.433]	[0.085,0.342]	[0.097,0.402]	[0.394,0.996]	[0.085,0.525]	[-0.049,0.138]	[0.118,0.475]	[0.085,0.342]	[0.098,0.405]	[0.392,0.992]	[0.084,0.524]	[-0.050,0.136]	[0.116,0.471]
Level 1 Observations (child)	24367	24367	4346	5015	7248	2034	2511	3195	4346	5015	7248	2034	2511	3195
Level 2 Observations (LGA)	684	684	113	100	166	85	111	121	113	100	166	85	111	121
Akaike Information Criterion	17045.336	17044.81	4014.257	3048.336	2966.083	1700.836	2421.769	2826.662	4014.885	3048.749	2966.498	1701.332	2421.402	2826.912
Prob. > X^2	<0.001	<0.001	<0.001	<0.001	<0.001	<0.001	<0.001	<0.001	<0.001	<0.001	<0.001	<0.001	<0.001	<0.001

95% confidence intervals in brackets
* p < 0.10, ** p < 0.05, *** p < 0.01

Table 6d: Additional robustness checks: decomposition of SIA exposure into different campaign types

Dependent variables: various indicators of routine immunisation, maternal care and child survival (see right)	**Non-polio full immu-nisation (full model)**	**Delivery**			**Antenatal care**		**Child survival (total exposure)**	**Non-polio full immunisation status (by region)**					
								**Full model**					
		At home	At private facility	At public facility	No. of antenatal care visits	No. of tetanus injections		North Central	North East	North West	South East	South South	South West
EXP_CHI (NIDs and sNIDs)	-0.037**<br>[-0.070,-0.004]							0.008<br>[-0.063,0.080]	-0.151***<br>[-0.233,-0.069]	-0.056<br>[-0.191,0.079]	0.038<br>[-0.125,0.202]	0.118*<br>[-0.021,0.256]	-0.103*<br>[-0.216,0.011]
EXP_CHI (IPDs and sIPDs)	-0.025***<br>[-0.037,-0.012]							0.043**<br>[0.006,0.080]	-0.093***<br>[-0.128,-0.058]	-0.078*<br>[-0.167,0.010]	0.119<br>[-0.029,0.268]	0.189***<br>[0.061,0.317]	-0.072<br>[-0.165,0.021]
EXP_CHI (mop-up campaigns)	-0.007<br>[-0.048,0.035]							-0.011<br>[-0.103,0.082]	0.027<br>[-0.048,0.101]	-0.139**<br>[-0.247,-0.030]	0.000<br>[0.000,0.000]	0.000<br>[0.000,0.000]	0.303<br>[-0.418,1.024]
EXP_CHI (MNCHW)	-0.047<br>[-0.146,0.052]							-0.175<br>[-0.393,0.044]	-0.102<br>[-0.359,0.155]	0.117<br>[-0.197,0.431]	-0.034<br>[-0.340,0.272]	-0.348***<br>[-0.610,-0.085]	-0.030<br>[-0.267,0.206]
EXP_PREG (NIDs and sNIDs)		0.014<br>[-0.018,0.046]	-0.063***<br>[-0.104,-0.021]	0.003<br>[-0.027,0.033]	-0.043*<br>[-0.086,0.000]	0.002<br>[-0.010,0.013]							
EXP_PREG (IPDs and sIPDs)		0.004<br>[-0.021,0.029]	-0.093***<br>[-0.127,-0.058]	0.020*<br>[-0.003,0.043]	-0.059***<br>[-0.095,-0.023]	-0.007<br>[-0.017,0.003]							
EXP_PREG (mop-up campaigns)		-0.012<br>[-0.049,0.025]	-0.021<br>[-0.093,0.051]	0.017<br>[-0.019,0.053]	0.022<br>[-0.015,0.058]	0.013*<br>[-0.002,0.029]							
EXP_PREG (MNCHW)		0.088**<br>[0.009,0.167]	0.024<br>[-0.066,0.114]	-0.075**<br>[-0.146,-0.004]	0.111*<br>[-0.009,0.230]	0.027*<br>[-0.002,0.057]							
EXP_TOT_nod (NIDs and sNIDs)							-0.035***<br>[-0.057,-0.014]						
EXP_TOT_nod (IPDs and sIPDs)							-0.014***<br>[-0.024,-0.004]						
EXP_TOT_nod (mop-up campaigns)							-0.018<br>[-0.041,0.005]						
EXP_TOT_nod (MNCHW)							0.040<br>[-0.052,0.133]						
CHI_AGE	0.079***<br>[0.062,0.095]						-0.004<br>[-0.015,0.007]	0.072***<br>[0.038,0.107]	0.082***<br>[0.036,0.129]	0.109***<br>[0.034,0.183]	0.121***<br>[0.075,0.168]	0.100***<br>[0.059,0.142]	0.081***<br>[0.043,0.119]
CHI_AGE2	-0.001***<br>[-0.001,-0.001]						0.000<br>[-0.000,0.000]	-0.001***<br>[-0.002,-0.000]	0.000<br>[-0.001,0.000]	-0.001**<br>[-0.002,-0.000]	-0.002***<br>[-0.003,-0.001]	-0.002***<br>[-0.002,-0.001]	-0.001**<br>[-0.001,-0.000]
CHI_ORD	-0.060***<br>[-0.085,-0.036]						-0.154***<br>[-0.176,-0.131]	-0.034<br>[-0.088,0.019]	-0.071**<br>[-0.133,-0.008]	-0.063*<br>[-0.129,0.003]	-0.036<br>[-0.099,0.027]	-0.074**<br>[-0.137,-0.011]	-0.087**<br>[-0.157,-0.017]
CHI_SEX	-0.004<br>[-0.078,0.071]						0.212***<br>[0.140,0.284]	-0.075<br>[-0.229,0.079]	0.022<br>[-0.168,0.211]	0.045<br>[-0.155,0.245]	-0.147<br>[-0.359,0.066]	0.196**<br>[0.003,0.390]	-0.006<br>[-0.183,0.170]
MOT_ANC	0.055***<br>[0.046,0.064]	-0.138***<br>[-0.146,-0.129]	0.057***<br>[0.049,0.064]	0.062***<br>[0.055,0.069]			-0.001<br>[-0.011,0.009]	0.094***<br>[0.072,0.115]	0.145***<br>[0.110,0.180]	0.064***<br>[0.028,0.101]	0.031***<br>[0.008,0.054]	0.041***<br>[0.020,0.061]	0.039***<br>[0.024,0.054]
MOT_EDM	0.061***<br>[0.043,0.079]	-0.065***<br>[-0.079,-0.050]	0.052***<br>[0.032,0.072]	0.057***<br>[0.043,0.071]	0.097***<br>[0.074,0.120]	0.031***<br>[0.024,0.038]	0.013<br>[-0.006,0.032]	0.070***<br>[0.035,0.105]	0.072***<br>[0.030,0.114]	0.067***<br>[0.021,0.114]	0.054*<br>[-0.005,0.114]	0.053**<br>[0.002,0.104]	0.043*<br>[-0.003,0.089]
MOT_EDF	0.010<br>[-0.008,0.028]	-0.030***<br>[-0.045,-0.016]	0.033***<br>[0.011,0.054]	0.035***<br>[0.020,0.049]	0.083***<br>[0.063,0.102]	0.023***<br>[0.017,0.029]	0.028***<br>[0.010,0.045]	0.006<br>[-0.030,0.043]	0.006<br>[-0.036,0.048]	0.029<br>[-0.013,0.071]	0.016<br>[-0.046,0.079]	0.007<br>[-0.050,0.063]	-0.030<br>[-0.079,0.019]
MOT_AWE	2.972***<br>[2.848,3.096]	-0.916***<br>[-0.991,-0.840]	0.275***<br>[0.162,0.387]	0.984***<br>[0.907,1.061]	1.937***<br>[1.804,2.069]	0.640***<br>[0.600,0.680]		2.561***<br>[2.321,2.800]	2.882***<br>[2.574,3.189]	3.549***<br>[3.267,3.831]	2.629***<br>[2.241,3.018]	2.927***<br>[2.580,3.273]	2.959***<br>[2.586,3.333]
MOT_AGE	0.026***<br>[0.018,0.035]	-0.002<br>[-0.007,0.003]	0.001<br>[-0.005,0.008]	0.002<br>[-0.002,0.007]	0.011***<br>[0.005,0.018]	0.000<br>[-0.001,0.002]	0.010**<br>[0.001,0.018]	0.017*<br>[-0.001,0.034]	0.031**<br>[0.007,0.054]	0.037***<br>[0.012,0.062]	0.013<br>[-0.011,0.036]	0.027**<br>[0.006,0.048]	0.031***<br>[0.011,0.051]
HH_RUR	-0.087*<br>[-0.190,0.016]	0.538***<br>[0.448,0.628]	-0.375***<br>[-0.485,-0.265]	-0.325***<br>[-0.408,-0.241]	-0.591***<br>[-0.818,-0.364]	-0.156***<br>[-0.206,-0.106]	-0.179***<br>[-0.282,-0.077]	-0.038<br>[-0.259,0.184]	-0.155<br>[-0.429,0.119]	-0.093<br>[-0.410,0.224]	-0.092<br>[-0.342,0.157]	-0.038<br>[-0.280,0.203]	-0.239*<br>[-0.486,0.008]
HH_REL (ref: Catholic): Other Christian	-0.126*<br>[-0.267,0.015]	0.095<br>[-0.029,0.218]	-0.095<br>[-0.217,0.027]	0.011<br>[-0.098,0.120]	-0.055<br>[-0.277,0.167]	-0.047*<br>[-0.099,0.006]	-0.137<br>[-0.303,0.028]	-0.105<br>[-0.374,0.164]	-0.562*<br>[-1.218,0.094]	0.465<br>[-0.245,1.175]	-0.109<br>[-0.337,0.119]	-0.409**<br>[-0.760,-0.059]	-0.093<br>[-0.592,0.405]
HH_REL (ref: Catholic): Islam	-0.472***<br>[-0.644,-0.300]	0.229***<br>[0.079,0.379]	-0.238***<br>[-0.399,-0.077]	-0.028<br>[-0.163,0.106]	-0.167<br>[-0.465,0.132]	-0.100***<br>[-0.172,-0.027]	-0.069<br>[-0.263,0.126]	-0.379**<br>[-0.682,-0.077]	-1.077***<br>[-1.745,-0.409]	-0.301<br>[-1.116,0.514]	-0.073<br>[-2.570,2.424]	0.020<br>[-0.754,0.793]	-0.403<br>[-0.920,0.114]
HH_REL (ref: Catholic): Traditionalist	-0.548***<br>[-0.955,-0.141]	1.008***<br>[0.650,1.366]	-0.914***<br>[-1.383,-0.445]	-0.478***<br>[-0.842,-0.115]	-0.650***<br>[-1.127,-0.173]	-0.349***<br>[-0.480,-0.218]	-0.091<br>[-0.463,0.281]	-0.697<br>[-1.815,0.421]	-1.628**<br>[-3.137,-0.120]	-0.649<br>[-2.242,0.944]	-0.246<br>[-0.881,0.389]	-0.724<br>[-1.736,0.288]	-0.190<br>[-1.479,1.099]
HH_REL (ref: Catholic): Other	-0.224<br>[-1.244,0.795]	-0.259<br>[-1.018,0.500]	0.021<br>[-1.253,1.294]	0.383<br>[-0.377,1.144]	-0.057<br>[-1.495,1.382]	-0.135<br>[-0.672,0.401]	0.573<br>[-0.601,1.747]	0.000<br>[0.000,0.000]	0.000<br>[0.000,0.000]	0.000<br>[0.000,0.000]	0.000<br>[0.000,0.000]	-0.301<br>[-1.532,0.929]	0.946<br>[-3.331,5.223]
HH_ETH (ref: Ekoi): Fulani	-0.926***<br>[-1.433,-0.418]	0.673***<br>[0.240,1.105]	-0.070<br>[-0.974,0.834]	-0.768***<br>[-1.192,-0.343]	-0.745<br>[-2.016,0.527]	-0.347***<br>[-0.587,-0.107]	-0.186<br>[-0.813,0.440]	-0.085<br>[-0.631,0.460]	-0.060<br>[-0.361,0.242]	0.110<br>[-0.568,0.787]	0.000<br>[0.000,0.000]		-0.692<br>[-1.755,0.371]
HH_ETH (ref: Ekoi): Hausa	-0.847***<br>[-1.334,-0.361]	0.945***<br>[0.525,1.364]	-0.572<br>[-1.433,0.290]	-0.828***<br>[-1.239,-0.416]	-0.437<br>[-1.703,0.829]	-0.250**<br>[-0.487,-0.013]	-0.504<br>[-1.122,0.115]	-0.483**<br>[-0.872,-0.095]	-0.073<br>[-0.378,0.231]	0.361<br>[-0.160,0.882]	-1.194<br>[-3.028,0.639]	-1.657<br>[-3.919,0.605]	-0.229<br>[-1.129,0.670]
HH_ETH (ref: Ekoi): Ibibio	-0.633**<br>[-1.168,-0.098]	0.372<br>[-0.101,0.844]	0.555<br>[-0.341,1.451]	-0.301<br>[-0.762,0.160]	-0.212<br>[-1.478,1.055]	-0.103<br>[-0.385,0.178]	-0.477<br>[-1.159,0.204]	-0.665<br>[-2.776,1.446]	0.000<br>[0.000,0.000]	0.000<br>[0.000,0.000]	-0.358<br>[-2.952,2.236]	-0.902***<br>[-1.429,-0.374]	1.125<br>[-0.229,2.479]
HH_ETH (ref: Ekoi): Igala	-0.657**<br>[-1.243,-0.071]	-0.513*<br>[-1.038,0.012]	1.298***<br>[0.405,2.190]	0.040<br>[-0.448,0.528]	0.004<br>[-1.313,1.322]	0.276*<br>[-0.016,0.568]	-0.400<br>[-1.112,0.313]	-0.132<br>[-0.525,0.260]	0.000<br>[0.000,0.000]	0.000<br>[0.000,0.000]	-0.644<br>[-3.219,1.932]	-0.762<br>[-3.465,1.941]	0.698<br>[-0.640,2.036]
HH_ETH (ref: Ekoi): Igbo	-0.450*<br>[-0.919,0.019]	-0.795***<br>[-1.211,-0.379]	1.881***<br>[1.051,2.711]	-0.568***<br>[-0.972,-0.164]	1.248*<br>[-0.035,2.531]	0.120<br>[-0.111,0.351]	-0.528*<br>[-1.138,0.081]	-0.163<br>[-0.690,0.364]	0.534<br>[-1.143,2.211]	0.274<br>[-0.817,1.364]	-0.896<br>[-2.200,0.408]	-0.502*<br>[-1.066,0.062]	0.524**<br>[0.017,1.032]
HH_ETH (ref: Ekoi): Ijaw / Izon	-0.486*<br>[-1.027,0.055]	0.883***<br>[0.397,1.369]	0.118<br>[-0.807,1.042]	-0.806***<br>[-1.282,-0.331]	-0.696<br>[-2.066,0.675]	-0.134<br>[-0.396,0.127]	-0.260<br>[-0.915,0.395]	0.000<br>[0.000,0.000]			0.000<br>[0.000,0.000]	-0.656**<br>[-1.158,-0.153]	-0.636<br>[-2.153,0.882]
HH_ETH (ref: Ekoi): Kanuri / Beriberi	-0.987***<br>[-1.591,-0.382]	1.001***<br>[0.511,1.492]	-0.518<br>[-1.659,0.622]	-0.956***<br>[-1.436,-0.476]	-0.512<br>[-1.801,0.777]	-0.237*<br>[-0.500,0.026]	-0.271<br>[-0.930,0.388]	-0.661<br>[-2.271,0.948]	-0.178<br>[-0.644,0.287]	0.639<br>[-0.618,1.895]		0.000<br>[0.000,0.000]	2.778*<br>[-0.171,5.727]
HH_ETH (ref: Ekoi): Tiv	-0.601**<br>[-1.158,-0.045]	-0.775***<br>[-1.247,-0.303]	1.727***<br>[0.847,2.608]	0.134<br>[-0.333,0.601]	-0.904<br>[-2.245,0.437]	-0.343**<br>[-0.609,-0.077]	-0.365<br>[-1.017,0.287]	-0.104<br>[-0.480,0.272]	-0.251<br>[-1.192,0.690]	0.000<br>[0.000,0.000]	0.000<br>[0.000,0.000]	0.000<br>[0.000,0.000]	1.786*<br>[-0.016,3.588]
HH_ETH (ref: Ekoi): Yoruba	-0.570**<br>[-1.048,-0.093]	-0.165<br>[-0.586,0.257]	1.300***<br>[0.465,2.136]	-0.413**<br>[-0.822,-0.004]	2.235***<br>[0.950,3.519]	0.059<br>[-0.174,0.291]	-0.048<br>[-0.669,0.574]	0.187<br>[-0.142,0.516]	0.329<br>[-1.144,1.801]	0.649<br>[-0.692,1.989]	0.000<br>[0.000,0.000]	-2.187***<br>[-3.154,-1.221]	0.292*<br>[-0.006,0.590]
HH_ETH (ref: Ekoi): Other	-0.616***<br>[-1.077,-0.154]	0.206<br>[-0.196,0.607]	0.912**<br>[0.083,1.740]	-0.349*<br>[-0.744,0.045]	0.030<br>[-1.220,1.279]	-0.017<br>[-0.247,0.213]	-0.361<br>[-0.967,0.245]	0.000<br>[0.000,0.000]	0.000<br>[0.000,0.000]	0.000<br>[0.000,0.000]	0.000<br>[0.000,0.000]	-0.887***<br>[-1.357,-0.417]	0.000<br>[0.000,0.000]
HH_SIZ	-0.013**<br>[-0.026,-0.000]	0.033***<br>[0.023,0.042]	-0.017**<br>[-0.032,-0.003]	-0.020***<br>[-0.030,-0.011]	-0.043***<br>[-0.056,-0.030]	-0.006***<br>[-0.010,-0.002]	0.132***<br>[0.118,0.146]	-0.011<br>[-0.036,0.015]	-0.021*<br>[-0.047,0.004]	-0.008<br>[-0.036,0.021]	-0.011<br>[-0.051,0.029]	-0.019<br>[-0.063,0.025]	-0.011<br>[-0.058,0.036]
HH_WEA	0.142***<br>[0.122,0.162]	-0.206***<br>[-0.223,-0.189]	0.120***<br>[0.099,0.141]	0.129***<br>[0.114,0.145]	0.287***<br>[0.256,0.318]	0.059***<br>[0.050,0.067]	0.016<br>[-0.004,0.036]	0.149***<br>[0.106,0.192]	0.102***<br>[0.048,0.155]	0.132***<br>[0.078,0.185]	0.165***<br>[0.113,0.216]	0.121***<br>[0.074,0.168]	0.142***<br>[0.090,0.195]
YEAR (ref: 2003): 2008	0.578***<br>[0.302,0.854]	0.021<br>[-0.164,0.206]	-0.203*<br>[-0.416,0.009]	0.056<br>[-0.120,0.232]	-0.569***<br>[-0.967,-0.170]	-0.080*<br>[-0.169,0.008]	0.268**<br>[0.062,0.473]	0.808***<br>[0.258,1.358]	0.797*<br>[-0.064,1.659]	1.411***<br>[0.469,2.352]	0.666*<br>[-0.076,1.407]	0.675*<br>[-0.121,1.472]	-0.271<br>[-0.966,0.425]
YEAR (ref: 2003): 2013	0.726***<br>[0.457,0.996]	-0.033<br>[-0.226,0.159]	-0.354***<br>[-0.576,-0.132]	0.131<br>[-0.051,0.312]	-0.217<br>[-0.635,0.201]	-0.005<br>[-0.097,0.088]	0.330***<br>[0.110,0.550]	0.760***<br>[0.231,1.289]	1.279***<br>[0.434,2.125]	1.883***<br>[0.837,2.928]	0.724**<br>[0.003,1.445]	1.410***<br>[0.626,2.195]	-0.820**<br>[-1.492,-0.148]
YEAR (ref: 2003): 2018	0.808***<br>[0.532,1.083]	-0.228**<br>[-0.417,-0.038]	-0.412***<br>[-0.637,-0.188]	0.342***<br>[0.162,0.522]	-1.275***<br>[-1.685,-0.866]	-0.051<br>[-0.145,0.043]	0.282***<br>[0.075,0.488]	1.141***<br>[0.583,1.699]	1.521***<br>[0.685,2.356]	1.159**<br>[0.248,2.070]	1.034**<br>[0.246,1.823]	1.843***<br>[0.981,2.705]	-0.801**<br>[-1.517,-0.086]
Constant	-5.234***<br>[-5.846,-4.623]	2.038***<br>[1.561,2.515]	-3.645***<br>[-4.533,-2.756]	-2.427***<br>[-2.893,-1.961]	3.586***<br>[2.215,4.958]	0.980***<br>[0.718,1.243]	2.596***<br>[1.919,3.273]	-5.701***<br>[-6.558,-4.844]	-5.778***<br>[-6.960,-4.596]	-8.231***<br>[-9.639,-6.823]	-4.939***<br>[-6.647,-3.231]	-5.672***<br>[-6.916,-4.428]	-4.425***<br>[-5.521,-3.330]
Multilevel variance parameter: Level 1	0.285***<br>[0.218,0.352]	0.451***<br>[0.364,0.538]	0.710***<br>[0.552,0.868]	0.463***<br>[0.384,0.543]	0.672***<br>[0.585,0.759]	-1.437***<br>[-1.535,-1.340]	0.041***<br>[0.013,0.068]	0.171***<br>[0.065,0.277]	0.146**<br>[0.032,0.261]	0.705***<br>[0.388,1.021]	0.168**<br>[0.034,0.303]	0.029<br>[-0.038,0.097]	0.243***<br>[0.088,0.398]
Multilevel variance parameter: Residual					1.322***<br>[1.282,1.361]	0.090***<br>[0.064,0.116]							
Level 1 Observations (child)	24381	34713	34713	34713	35019	36585	52431	4342	5017	7249	2036	2514	3196
Level 2 Observations (LGA)	684	686	686	686	686	687	689	113	100	166	85	111	121
Akaike Information Criterion	18035.652	26423.512	17597.951	30317.164	193631.04	111221.63	23679.445	4154.682	2852.628	2871.344	2200.745	2604.453	3247.815
Prob. > X^2	<0.001	<0.001	<0.001	<0.001	<0.001	<0.001	<0.001	<0.001	<0.001	<0.001	<0.001	<0.001	<0.001

95% confidence intervals in brackets. NID = National Immunisation Day; sNID = sub-National Immunsiation Day; IPD = Immunisation-Plus Day; sIPD = sub-national Immunisation-Plus Day; MNCHW = Maternal, Neonatal, and Child Health Week; SIA = Supplementary Immunisation Activity.

* p < 0.10, ** p < 0.05, *** p < 0.01

Table 6e: Additional robustness checks: fixed effects models

Dependent variables: various indicators of routine immunisation, maternal care and child survival (see right)	**Non-polio full immunisation**		**Delivery**			**Antenatal care**		**Child**
	Full model	Interaction model (EXPxAGE)	At home	At private facility	At public facility	No. of antenatal care visits	No. of tetanus injections	Total exposure
EXP_CHI	-0.020**	0.034**						
	[-0.036,-0.004]	[0.006,0.062]						
EXPxAGE		-0.001***						
		[-0.002,-0.001]						
EXP_PREG			-0.016	0.007	0.011	0.023	0.012***	
			[-0.038,0.006]	[-0.023,0.037]	[-0.009,0.032]	[-0.005,0.052]	[0.004,0.021]	
EXP_TOT_nod (total exposure, date approximation)								-0.029***
								[-0.041,-0.017]
CHI_AGE	0.080***	0.036***						0.001
	[0.064,0.095]	[0.028,0.043]						[-0.010,0.011]
CHI_AGE2	-0.001***							0.000
	[-0.001,-0.001]							[-0.000,0.000]
CHI_ORD	-0.059***	-0.060***						-0.148***
	[-0.085,-0.034]	[-0.085,-0.034]						[-0.171,-0.125]
CHI_SEX	-0.001	-0.003						0.212***
	[-0.078,0.076]	[-0.080,0.074]						[0.139,0.285]
MOT_ANC	0.059***	0.059***	-0.133***	0.047***	0.063***			0.003
	[0.049,0.068]	[0.049,0.068]	[-0.142,-0.125]	[0.039,0.055]	[0.056,0.070]			[-0.008,0.013]
MOT_EDM	0.060***	0.060***	-0.059***	0.040***	0.053***	0.091***	0.027***	0.015
	[0.042,0.079]	[0.042,0.079]	[-0.074,-0.044]	[0.019,0.061]	[0.038,0.067]	[0.070,0.112]	[0.021,0.033]	[-0.005,0.035]
MOT_EDF	0.002	0.002	-0.028***	0.018	0.033***	0.078***	0.019***	0.033***
	[-0.017,0.021]	[-0.017,0.021]	[-0.043,-0.013]	[-0.004,0.040]	[0.019,0.048]	[0.059,0.097]	[0.014,0.025]	[0.015,0.051]
MOT_AWE	2.988***	2.984***	-0.904***	0.237***	0.951***	1.899***	0.615***	
	[2.858,3.117]	[2.854,3.113]	[-0.982,-0.827]	[0.123,0.352]	[0.873,1.030]	[1.796,2.002]	[0.586,0.645]	
MOT_AGE	0.026***	0.027***	-0.002	-0.002	0.002	0.010***	0.000	0.008*
	[0.018,0.035]	[0.018,0.036]	[-0.007,0.004]	[-0.009,0.005]	[-0.002,0.007]	[0.003,0.016]	[-0.002,0.002]	[-0.000,0.017]
HH_RUR	-0.088	-0.091	0.531***	-0.261***	-0.368***	-0.527***	-0.169***	-0.174**
	[-0.211,0.035]	[-0.214,0.033]	[0.430,0.632]	[-0.381,-0.141]	[-0.459,-0.276]	[-0.662,-0.391]	[-0.207,-0.131]	[-0.308,-0.041]
HH_REL (ref: Catholic): Other Christian	-0.117	-0.119	0.036	-0.034	0.001	-0.063	-0.029	-0.113
	[-0.267,0.032]	[-0.268,0.031]	[-0.093,0.164]	[-0.159,0.091]	[-0.112,0.113]	[-0.240,0.113]	[-0.079,0.021]	[-0.292,0.066]
HH_REL (ref: Catholic): Islam	-0.407***	-0.406***	0.150*	-0.073	-0.041	-0.041	-0.033	-0.054
	[-0.594,-0.219]	[-0.593,-0.218]	[-0.009,0.309]	[-0.240,0.094]	[-0.182,0.100]	[-0.258,0.175]	[-0.094,0.029]	[-0.273,0.165]
HH_REL (ref: Catholic): Traditionalist	-0.530**	-0.528**	0.952***	-0.899***	-0.492***	-0.577***	-0.305***	-0.101
	[-0.953,-0.107]	[-0.951,-0.105]	[0.582,1.322]	[-1.369,-0.428]	[-0.864,-0.121]	[-0.985,-0.169]	[-0.421,-0.188]	[-0.492,0.290]
HH_REL (ref: Catholic): Other	-0.430	-0.433	-0.411	0.109	0.395	-0.065	-0.093	0.670
	[-1.507,0.648]	[-1.508,0.641]	[-1.197,0.375]	[-1.200,1.418]	[-0.393,1.182]	[-1.222,1.093]	[-0.417,0.231]	[-0.529,1.868]
HH_ETH (ref: Ekoi): Fulani	-0.669**	-0.681**	0.762***	0.142	-0.693***	-0.693*	-0.300***	0.219
	[-1.279,-0.059]	[-1.292,-0.071]	[0.276,1.248]	[-0.903,1.187]	[-1.167,-0.219]	[-1.442,0.057]	[-0.516,-0.085]	[-0.607,1.046]
HH_ETH (ref: Ekoi): Hausa	-0.527*	-0.537*	0.919***	-0.152	-0.675***	-0.249	-0.149	-0.066
	[-1.124,0.069]	[-1.134,0.061]	[0.440,1.397]	[-1.158,0.854]	[-1.140,-0.210]	[-0.995,0.496]	[-0.363,0.065]	[-0.888,0.757]
HH_ETH (ref: Ekoi): Ibibio	-0.385	-0.388	0.236	0.322	-0.106	-0.354	-0.180	-0.203
	[-1.021,0.251]	[-1.025,0.248]	[-0.298,0.770]	[-0.712,1.356]	[-0.620,0.407]	[-1.185,0.477]	[-0.418,0.057]	[-1.114,0.707]
HH_ETH (ref: Ekoi): Igala	-0.482	-0.492	0.020	0.699	-0.098	-0.410	0.190	0.130
	[-1.206,0.241]	[-1.215,0.232]	[-0.569,0.609]	[-0.330,1.729]	[-0.643,0.448]	[-1.317,0.498]	[-0.070,0.451]	[-0.847,1.108]
HH_ETH (ref: Ekoi): Igbo	-0.167	-0.167	-0.526**	1.189**	-0.333	0.831**	-0.034	0.017
	[-0.751,0.417]	[-0.752,0.418]	[-1.007,-0.045]	[0.223,2.156]	[-0.792,0.125]	[0.082,1.581]	[-0.249,0.181]	[-0.821,0.855]
HH_ETH (ref: Ekoi): Ijaw / Izon	-0.302	-0.306	0.708**	-0.017	-0.599**	-0.842*	-0.163	0.490
	[-0.987,0.384]	[-0.992,0.380]	[0.147,1.269]	[-1.095,1.060]	[-1.143,-0.056]	[-1.688,0.005]	[-0.404,0.078]	[-0.445,1.425]
HH_ETH (ref: Ekoi): Kanuri / Beriberi	-0.488	-0.497	1.038***	-0.045	-0.887***	-0.382	-0.149	-0.082
	[-1.194,0.218]	[-1.203,0.209]	[0.495,1.581]	[-1.336,1.246]	[-1.415,-0.359]	[-1.176,0.412]	[-0.378,0.079]	[-0.945,0.780]
HH_ETH (ref: Ekoi): Tiv	-0.357	-0.373	-0.297	0.992*	0.087	-0.814*	-0.362***	0.209
	[-1.084,0.369]	[-1.100,0.354]	[-0.841,0.248]	[-0.036,2.020]	[-0.447,0.621]	[-1.670,0.042]	[-0.608,-0.116]	[-0.722,1.139]
HH_ETH (ref: Ekoi): Yoruba	-0.230	-0.238	0.291	0.702	-0.428*	1.347***	0.048	0.139
	[-0.826,0.365]	[-0.834,0.358]	[-0.194,0.776]	[-0.273,1.677]	[-0.894,0.038]	[0.589,2.104]	[-0.169,0.266]	[-0.710,0.988]
HH_ETH (ref: Ekoi): Other	-0.393	-0.395	0.372	0.595	-0.317	-0.007	-0.006	-0.023
	[-0.958,0.171]	[-0.960,0.170]	[-0.083,0.828]	[-0.372,1.561]	[-0.761,0.126]	[-0.731,0.717]	[-0.214,0.202]	[-0.831,0.786]

HH_SIZ	-0.016**	-0.016**	0.033***	-0.014*	-0.022***	-0.039***	-0.007***	0.137***
	[-0.030,-0.003]	[-0.030,-0.003]	[0.023,0.043]	[-0.029,0.001]	[-0.032,-0.013]	[-0.052,-0.027]	[-0.011,-0.004]	[0.123,0.152]
HH_WEA	0.141***	0.140***	-0.204***	0.108***	0.131***	0.276***	0.058***	0.008
	[0.120,0.163]	[0.118,0.161]	[-0.221,-0.186]	[0.086,0.130]	[0.115,0.147]	[0.253,0.299]	[0.051,0.064]	[-0.014,0.030]
YEAR (ref: 2003): 2008	0.517***	0.495***	0.040	-0.251**	0.087	-0.670***	-0.108***	0.323***
	[0.235,0.799]	[0.211,0.778]	[-0.156,0.236]	[-0.472,-0.030]	[-0.097,0.270]	[-0.921,-0.420]	[-0.180,-0.037]	[0.097,0.549]
YEAR (ref: 2003): 2013	0.673***	0.635***	-0.042	-0.388***	0.170*	-0.314**	-0.029	0.547***
	[0.394,0.952]	[0.353,0.916]	[-0.237,0.153]	[-0.607,-0.169]	[-0.011,0.351]	[-0.563,-0.065]	[-0.100,0.043]	[0.313,0.780]
YEAR (ref: 2003): 2018	0.800***	0.890***	-0.267***	-0.385***	0.391***	-1.272***	-0.059	0.314***
	[0.512,1.087]	[0.603,1.177]	[-0.468,-0.066]	[-0.617,-0.153]	[0.203,0.578]	[-1.530,-1.013]	[-0.133,0.016]	[0.094,0.535]
Observations	24381	24381	34713	34713	34713	35019	36585	52431
Akaike Information Criterion	18032.806	18053.939	26153.968	17075.688	29973.334	192578.24	111031.373	24062.176
Prob. > X^2	<0.001	<0.001	<0.001	<0.001	<0.001	<0.001	<0.001	<0.001
Hausman test	0.068	0.023	<0.001	<0.001	<0.001	<0.001	<0.001	<0.001

95% confidence intervals in brackets. 774 LGA-level fixed effects estimators omitted from reporting.
* p < 0.10, ** p < 0.05, *** p < 0.01
